# Synthesis and Photoacoustic Characterization of Amphiphilic Ni(II) Phthalocyanines as Calorimetric References

**DOI:** 10.1002/cphc.70561

**Published:** 2026-11-05

**Authors:** Sidharth Thulaseedharan Nair Sailaja, Niek M. M. Aarts, Iván Maisuls, Alexander Hepp, Andreas Faust, Cristian A. Strassert

**Affiliations:** ^1^ Institut für Anorganische und Analytische Chemie Universität Münster Münster Germany; ^2^ CeNTech, SoN, CiMIC, Universität Münster Münster Germany; ^3^ European Institute for Molecular Imaging Münster Germany; ^4^ Department of Nuclear Medicine University Hospital Münster Münster Germany

**Keywords:** aggregation, amphiphiles, Ni(II) phthalocyanines, photoacoustic spectroscopy, transient absorption spectroscopy

## Abstract

This work describes the synthesis and photophysical characterization of a series comprised by four nonionic yet amphiphilic octa‐substituted Ni(II) phthalocyanines (NiPc) with peripheral or proximal decoration. The combination of MALDI–TOF mass spectrometry, ^1^H‐NMR, ^13^C‐NMR, and UV–vis spectroscopy enabled an unambiguous structural elucidation. Notably, these NiPcs are soluble in both polar organic solvents and aqueous media. Since these complexes exhibit near‐infrared absorption (*λ*_max_ = 650–800 nm), are non‐emissive, and display remarkable chemical stability, a new experimental setup was developed to investigate their photoacoustic efficiency by combining transient absorption spectroscopy and laser‐induced photoacoustic calorimetry. This approach revealed that all complexes are equally suited and stable as photoacoustic references in both aqueous and organic media.

## Graphical abstract

A series comprised by four nonionic yet amphiphilic octa‐substituted Ni(II) phthalocyanines is introduced. These NiPcs are soluble in both polar organic solvents and aqueous media. A new experimental setup was developed to investigate their photoacoustic efficiency by combining transient absorption and photoacoustic spectroscopy. This approach revealed that all complexes are equally suited and stable as photoacoustic references in both aqueous and organic media.

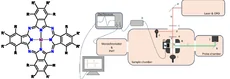

## 1 Introduction

In recent decades, increasing attention has been devoted to phthalocyanines (Pc) owing to their multifaceted properties and inherent stability. The literature reports numerous articles and reviews exploring their potential applications in diverse areas, including chemical sensing [1], catalysis [2], photochemistry [3, 4], electrochemistry [5], nonlinear optics [6], photodynamic therapy (PDT) [7, 8], and bioimaging [9], among many others. By appropriately selecting the metal centers and substituents, the photophysical and photochemical properties of Pc can be fine‐tuned on demand. A major drawback, however, lies in the fact that unsubstituted metallophthalocyaninates exhibit very limited solubility in common organic solvents and aqueous media. This drawback is attributed to the strong *π–π* interactions between stacked monomers that promote the formation of dimers and higher aggregates [1, 10]. However, the solubility can be enhanced by introducing suitable substituents at the peripheral (*β*) or proximal (*α*) positions of the macrocycle. Typically, ionic substituents have been employed to improve the water solubility of Pcs [11]. These can be attached either directly to the phthalonitrile precursors or at the axial coordination site of the central atom [12]. Common examples include anionic groups such as sulfonates [13], carboxylates [14, 15], and phosphorus‐based moieties [16, 17], as well as cationic substituents such as quaternary ammonium [18] or pyridinium units [19, 20]. In contrast, nonionic yet water‐soluble Pcs have been obtained through the introduction of hydrophilic groups such as carbohydrates, poly(ethylene glycol), or polyhydroxylated substituents [12]. Nevertheless, most hydrophilic coordination compounds tend to form aggregates under aqueous conditions, resulting in the quenching of excited states and impairing their photosensitizing activity, thus rendering the molecules photodynamically inactive. To overcome these drawbacks, various design strategies have been explored such as the incorporation of bulky ionic groups at peripheral positions [21] or long alkylsulfanyl or alkoxy chains at proximal sites [1, 22]. Among these, Zn(II) phthalocyanines (ZnPc) with diverse substitution patterns represent the majority of reported systems [1, 23, 24]. Conversely, studies on water‐soluble Ni(II) phthalocyanines (NiPc) remain scarce. Most reported examples are either cationic or anionic and therefore soluble only in water but insoluble in organic solvents [25, 26]. This limited solubility not only hampers their purification and spectroscopic characterization but also restricts their applicability in areas such as homogeneous catalysis and other technologically relevant fields [27]. Motivated by these features, we herein report on our newly designed and synthesized universally soluble Ni(II) phthalocyanines with well‐defined substituents, combining structural versatility with strong near‐infrared (NIR) absorption and efficient nonradiative energy conversion, making them suitable for photothermal and optoacoustic investigations.

In this context, laser‐induced photoacoustic spectroscopy (PAS) as well as laser‐induced photoacoustic calorimetry (PAC) both constitute powerful tools to study light‐to‐heat conversion in molecular systems. Originally discovered by Alexander Graham Bell [28, 29], the photoacoustic effect was further developed by Rosencwaig and has since found application ranging from thermodynamic and photophysical studies to phototherapy and biomedical imaging [30, 31, 32, 33, 34, 35, 36, 37, 38]. The effect arises from local pressure changes (acoustic pressure waves and PA signals) generated by the thermoelastic expansion of a sample upon absorption of pulsed electromagnetic radiation, which produces local heating through nonradiative relaxation [39, 40, 41, 42]. These pressure waves can be detected using a microphone or a piezoelectric transducer. Complementary to PAS, transient absorption spectroscopy (TAS) is widely employed to investigate excited‐state dynamics, where a pump laser excites the sample and transient absorbance changes induced by a probe beam reveal population evolution following excitation [43].

Phthalocyanines have been extensively studied as sensitizers for PDT, photothermal therapy, and contrast agents for photoacoustic imaging due to their strong absorption in the NIR region, which enables efficient tissue penetration [9, 44, 45]. However, to the best of our knowledge, their use as reference compounds for PAS in both aqueous and organic media has not been reported. Given that NiPcs are non‐emissive and rapidly relax to the ground state due to the low ligand field splitting of the metal center, they are particularly suitable as photocalorimetric references.

Therefore, we aimed to develop NiPcs with strong NIR absorption and isomeric purity, obtained through an unambiguous synthetic route, while maintaining solubility in both water and polar organic solvents. Furthermore, we introduce a combined experimental setup that integrates PAS and TAS by reengineering a commercially available transient absorption spectrometer. This configuration allows orthogonal monitoring of relaxation pathways involving non‐emissive states without long‐lived intermediates acting as energy‐storing species, ensuring the full conversion of absorbed energy into prompt heat. In summary, this work presents the synthesis of four highly soluble NiPcs together with an integrated PAS–TAS setup, enabling an in‐depth investigation of their photothermal properties supported by comprehensive structural characterization.

## 2 Results and Discussion

### 2.1 Synthesis, Structural, and Photophysical Characterization of NiPcs

We performed a de novo synthesis and full structural characterization of four new octa‐substituted Ni(II) phthalocyanines bearing either 2‐(2‐ethoxy)ethoxyl or 2‐(2‐(2‐sulfanylethoxy)ethoxy)ethoxyl chains in the proximal (*α*) (**NiPc‐1** and **NiPc‐3**) or peripheral (*β*) positions (**NiPc‐2** and **NiPc‐4**). As shown in Scheme 1, the targeted octa‐substituted NiPcs were obtained via cyclotetramerization of the corresponding phthalonitrile precursors with Ni(OAc)_2_ in the presence of 1,8‐‍diazabicycloundec‐7‐ene (DBU) as a catalyst in refluxing isoamyl alcohol (*i*‐amOH).

**SCHEME 1:**
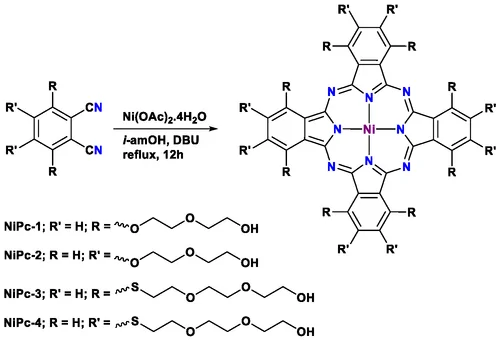
Synthesis of NiPcs. Further experimental details, including reaction yields and characterization data, can be found in the ESI.

The obtained phthalocyanines were purified by size‐exclusion chromatography (Sephadex LH‐20) using methanol (detailed synthetic procedures are provided in the ESI). The electronic absorption spectra of the nonionic NiPcs were recorded in both water and dimethyl sulfoxide (DMSO), displaying the characteristic Q‐ and B‐bands (Figure 1). The B‐band, or Soret band, corresponds to the transition from S_0_ to a higher singlet state (π–π*’ character), whereas the Q‐band arises from the ground state (S_0_) to the lowest doubly degenerate excited state (S_1/2_, *π–π* ^∗^ character) [46].

**FIGURE 1:**
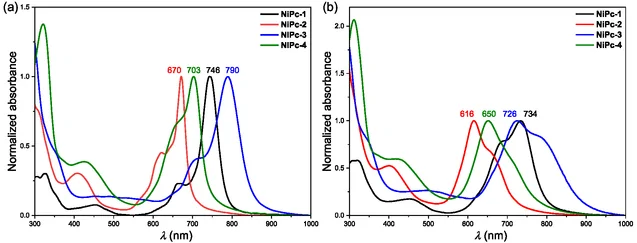
UV–vis absorption spectra normalized to the corresponding Q‐band of the NiPcs: (a) in DMSO and (b) in water. For more details regarding UV–vis spectra at different concentrations, see Section S5.

As previously reported for Zn(II) phthalocyanines [1, 11, 22, 23], the Q‐bands appeared between 670–790 nm in DMSO and 610–740 nm in water, while the B‐band was centered at around 350–370 nm in both solvents. The UV–vis absorption data are shown in Table S1. It is well documented that *α*‐substitution of phthalocyanines destabilizes the HOMO of the macrocycle, resulting in a bathochromic shift of the Q‐band compared to the *β*‐substituted analogues [1, 23]. Consistently, **NiPc‐1** exhibits a Q‐band redshifted by ~76 nm relative to **NiPc‐2**, and **NiPc‐3** appears ~87 nm redshifted compared to **NiPc‐4**.

Additionally, the electron‐donating sulfanyl linkers induce further bathochromic shifts in the sulfur‐containing phthalocyanines (**NiPc‐3** and **NiPc‐4**) compared to their oxygen‐bridged counterparts (**NiPc‐1** and **NiPc‐2**). Consequently, **NiPc‐3**, with sulfur atoms in the *α*‐positions, shows the most pronounced redshift among the series, in agreement with previous studies [47, 48]. Furthermore, the Q‐bands of sulfur‐bearing phthalocyanines are substantially broadened relative to the oxygen‐containing analogues. This broadening can be attributed to a higher tendency for aggregation, likely due to stronger van der Waals interactions associated with the electron‐richer sulfur atoms, as reported elsewhere [32].

Although the synthesized NiPcs displayed excellent solubility in water, their absorption spectra in an aqueous medium provided clear evidence of aggregation [1, 11]. Compared with the spectra in DMSO, the Q‐band maxima of the complexes underwent hypsochromic shifts upon dissolution in water, indicative of *H*‐type aggregation. Specifically, the Q‐band shifted from 670 to 616 nm for **NiPc‐2**, from 790 to 726 nm for **NiPc‐3**, and from 703 to 650 nm for **NiPc‐4**. In contrast, the spectrum of **NiPc‐1** showed only a minor shift (746–734 nm), suggesting a weaker degree of aggregation relative to the other complexes.

Except for **NiPc‐1** and **NiPc‐3**, which exist predominantly as monomers in DMSO, the remaining complexes form stable, non‐dissociating *H*‐aggregates, as indicated by their molar absorption coefficients and spectral profiles (Figures S74–S89). The molar absorption coefficients of all NiPcs are typical for phthalocyanines and comparable to those reported for related derivatives [25, 26, 49, 50, 51]. As expected, none of the NiPcs exhibited fluorescence in the NIR region, consistent with efficient radiationless deactivation to the ground state following photoexcitation in both solvents.

In addition to their optical characterization, the structural features of the synthesized NiPcs were confirmed by mass spectrometry and nuclear magnetic resonance (NMR) spectroscopy. Due to the intrinsic aggregation tendency of phthalocyanines, obtaining well‐resolved ^1^H‐ and ^13^C‐NMR spectra is typically challenging, and most related compounds reported in the literature have been only partially characterized by NMR. Remarkably, the good solubility and high symmetry of the present octa‐substituted NiPcs enabled the acquisition of well‐resolved ^1^H‐NMR spectra (Section S3). In line with previous studies [52], the proximally *α*‐substituted species (**NiPc‐1** and **NiPc‐3**) displayed a reduced tendency to aggregate compared with their *β*‐substituted counterparts (**NiPc‐2** and **NiPc‐4**).

The ^1^H‐ and ^13^C‐NMR spectra of **NiPc‐1** and **NiPc‐3** were fully assigned using 2D NMR techniques (Figures S40–S60). Their ^1^H‐‍NMR spectra exhibited a single peak in the aromatic region and well‐defined aliphatic resonances, consistent with the proposed structures. Conversely, the room‐temperature ^1^H‐NMR spectra of **NiPc‐2** and **NiPc‐4** were relatively broad, reflecting aggregation at the concentrations used for NMR experiments. To overcome this limitation, variable temperature ^1^H‐NMR experiments were performed up to 350 K (Figures S50 and S60 for **NiPc‐2** and **NiPc‐4**, respectively). Upon heating, sharper and more intense signals were obtained, allowing their assignment by comparison with the corresponding *α*‐substituted analogues. The proposed structures were further corroborated by mass spectrometry of both the coordination compounds and their phthalonitrile precursors. Matrix‐assisted laser desorption ionization–time of flight (MALDI–TOF) spectra of the NiPcs and electrospray ionization mass spectrometry data for the phthalonitrile derivatives showed excellent agreement between the calculated and observed isotopic patterns (see Figures S61–S73).

### 2.2 Laser‐Induced Photoacoustic Calorimetry

Both PAS and PAC rely on the generation of an acoustic pressure wave upon absorption of a light pulse, followed by radiationless relaxation and thermalization of the excited species within the surrounding medium (along with a concomitant thermoelastic expansion) [30]. For molecules in solution, photon absorption produces vibronically excited states that relax to the ground state through radiative and radiationless pathways. The latter leads to local heat release (*H*), which depends on the excitation pulse energy (*E*) according to

(1) $$H=\alpha \left(1-10^{-A}\right)E$$

where *α* is the fraction of absorbed energy converted into prompt heat and *A* is the absorbance of the sample at the excitation wavelength [53]. The heat generated induces a transient temperature rise (ΔT) in the excitation volume (*V*_exc_)

(2) $$H=C_{p}V_{\mathrm{exc}}\rho \text{Δ}T$$

with *C*_p_ being the heat capacity and *ρ* the solvent density. The sudden temperature increase results in a local pressure change (Δp) described by

(3) $$\text{Δ}p=\gamma_{v}\text{Δ}T$$

where *γ*_v_ is the thermal pressure coefficient. The resulting acoustic wave is detected by a piezoelectric transducer, which converts pressure into a voltage signal (*S*). Combining the above expressions yields

(4) $$S\propto \text{Δ}p=B\frac{\gamma_{v}}{C_{p}V_{\mathrm{exc}}\rho }H$$

where *B* is an instrumental constant accounting for the transducer characteristics and acoustic coupling. Substituting Equation (1) into Equation (4) leads to the final relation between the excitation energy and the amplitude of the first photoacoustic wave (ΔS)

(5) $$\text{Δ}S=\alpha K(1-10^{-A})E$$

where *K* encompasses all experimental constants [54]. Since *K* is unknown, a calorimetric reference compound (with known *α*_r_) is used for calibration under the same conditions as the sample is measured. The value of *α* for a given sample (*α*_s_) can then be determined from the slopes of the linear fit of ΔS versus *E* for both the sample and the reference. If *α*_r_ = 1, then

(6) $$\frac{\frac{d\text{Δ}S_{s}}{dE_{s}}}{\frac{d\text{Δ}S_{r}}{dE_{r}}}=\;\frac{\alpha_{s}K(1-10^{-A_{s}})}{\alpha_{r}K(1-10^{-A_{r}})}\;=\alpha_{s}\frac{1-10^{-A_{s}}}{1-10^{-A_{r}}}$$

where *A*_s_ and *A*_r_ are the absorbances of sample and reference, respectively. Thus, *α*_s_ can be determined directly from experimental data, without requiring explicit knowledge of *K* [53].

### 2.3 Methodology

TAS measurements were performed using a LP980 spectrometer (Edinburgh Instruments) equipped with a Q‐switched Nd:YAG laser (Ekspla NT342B) producing 4–6 ns pump pulses at 355 nm (third harmonic). An integrated optical parametric oscillator (OPO) provided tunable excitation from 410–710 nm (signal beam, 0.1 nm increments) and from 710 to 2600 nm (idler beam, 1 nm increments). The probe beam, generated by a Xe lamp, intersected the pump pulse at 90° inside the sample chamber and was detected with a Czerny–Turner‐type monochromator (325 nm focal length, 1800 grooves/mm, 500 nm blaze) coupled to a photomultiplier tube (PMT, 200–870 nm range). The resulting transient signals were captured by a Tektronix MDO3022 oscilloscope, yielding a temporal resolution of approximately 5 ns. All data acquisition and synchronization were controlled by the L900 software provided by Edinburgh Instruments.

To measure PAS with this setup, several adaptations were implemented (Figure 2). The setup was modified to include two power sensors (Coherent J‐10 MB‐LE and J‐25 MB‐HE) positioned before and after the sample to measure the incident and transmitted laser power, respectively, allowing an accurate determination of the absorbed energy per pulse. The original sample holder was replaced by a custom‐designed device capable of accommodating a 1 MHz piezoelectric transducer for detecting the photoacoustic signal. The holder incorporated precise temperature and stirring control (Quantum Northwest TC‐1) to ensure stable and reproducible experimental conditions. In addition, slits or pinholes can be mounted in the sample holder to modify the shape of the incident laser pulse [55]. In this setup, both the slit position and the cuvette are mechanically fixed within the sample holder, preventing displacement during measurements and ensuring a stable and reproducible excitation and acoustic detection geometry. A thin layer of coupling grease (vaseline‐ or paraffin‐based) was applied between the transducer and the cuvette to enhance acoustic coupling and mechanical contact [56]. The electric output from the transducer was amplified (using an Evident 5682 preamplifier) and recorded on a separate oscilloscope channel. In order to acquire the data, the L900 software was adapted to process and store both data streams, thus integrating PAS into the existing TAS workflow without requiring external synchronization or hardware.

**FIGURE 2:**
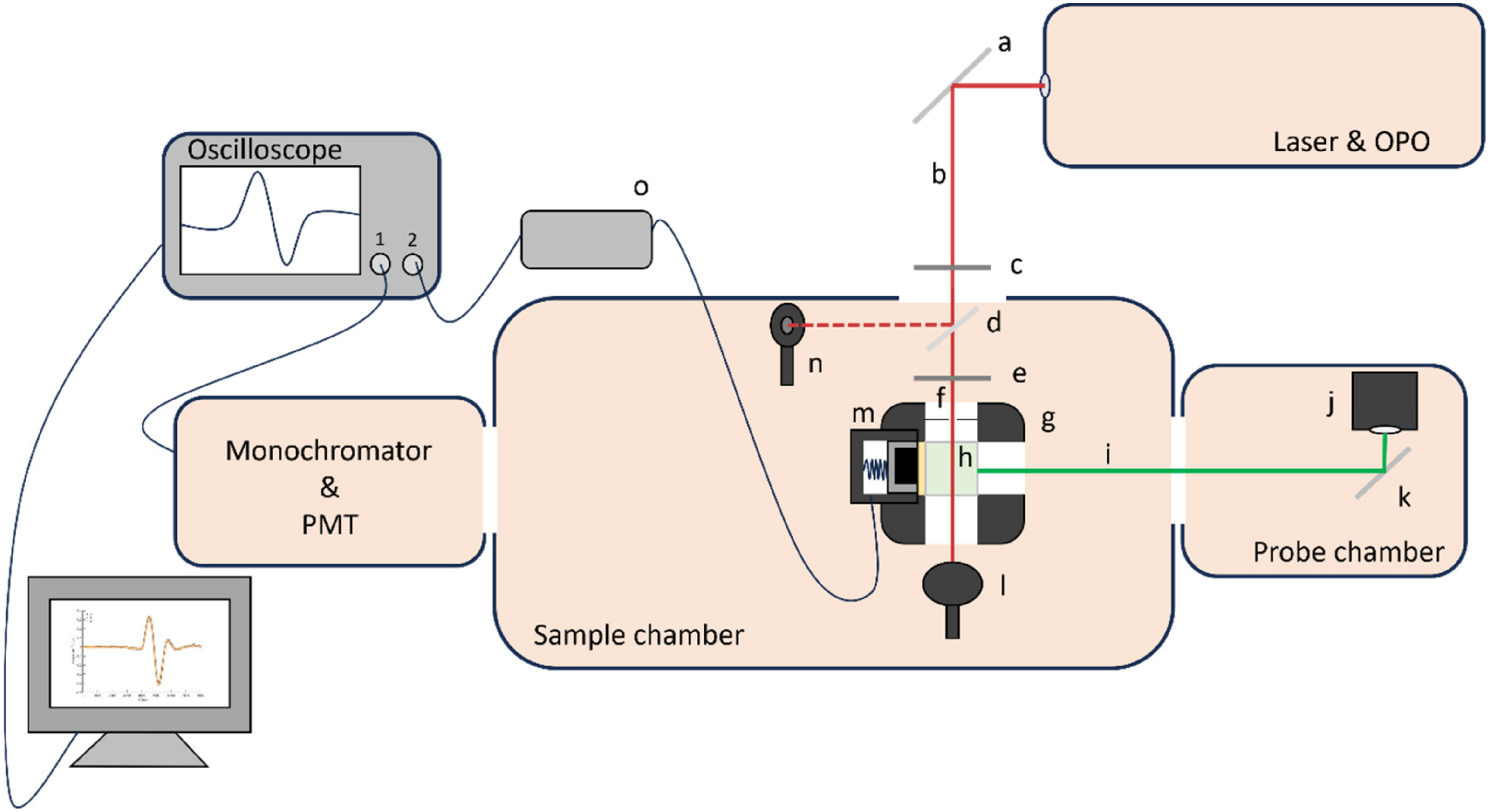
Schematic overview of the PAS and TAS setup: (a) mirror; (b) pump beam path; (c) first ND filter; (d) beam splitter; (e) second ND filter; (f) vertical slit; (g) custom sample holder; (h) sample cuvette; (i) probe beam path; (j) Xe arc lamp; (k) probe beam steering mirror; (l) power sensor after sample; (m) removable custom holder with piezoelectric transducer; (n) power sensor before sample; (o) preamplifier. For the measurement of TAS, piezoelectric transducer is removed and the probe light is detected by the monochromator and the PMT.

For the determination of *α*_s_, the incident laser power (85 mJ) was varied using absorptive neutral density (ND) filters (OD 0.1–0.7, Thorlabs, NE200B series) or by employing the OPO’s internal attenuator for tunable wavelengths between 410 and 710 nm. For the calorimetric measurements performed in this study, a vertical slit (0.5 cm width) was used to define the laser beam profile at the sample and ensure a reproducible excitation volume [42].

Photoacoustic signals of the NiPcs were recorded at *λ*_exc_ = 355 nm in both DMSO and water. Orthohydroxybenzophenone (OHBP) and new coccine (NC) were employed as calorimetric reference compounds in DMSO and water, respectively [55, 57]. The concentration of sample and reference solutions was adjusted to achieve absorbances between 0.1 and 0.2 at the excitation wavelength.

For each compound, two independent measurements were recorded at six different laser pulse energies, yielding 12 pressure transients for linear regression. Each transient was averaged over five laser shots to enhance the signal‐to‐noise ratio. The use of five accumulations was verified by comparison with measurements acquired using higher numbers of laser shots, showing no changes in the extracted signal features (see Figures S114 and S115). The resulting waveforms were processed using a Savitzky–Golay filter (50‐point window) to extract the signal amplitude (Δ*S*) from the peak‐to‐peak difference [58]. The slopes from ΔS versus *E* plots were used to determine the fraction of absorbed energy released as prompt heat (*α*_s_) according to Equation (6), with uncertainties estimated as twice the standard deviation (2*σ*_s_) of the linear fit. Validation against extensive averaging (64 shots) using NC as a reference confirmed the robustness of the smoothing and fitting procedure (see Figures S114 and S115). All data analysis was performed in MATLAB 2019b [59].

### 2.4 Photoacoustic Response of Ni(II) Phthalocyanines

The absorbance‐normalized acoustic transients of the reference and **NiPc‐4** in DMSO upon 355 nm excitation are shown in Figure 3, as a representative example for all samples. Immediately after the laser pulse, a small signal attributed to light scattering at the cuvette walls is detected, as previously reported by Schmid et al. [54, 56] After approximately 4 µs, the first pressure transient appears, lasting for about 2 µs. The identical shape and timing of the signals for both the reference and **NiPc‐4** confirm that the photoacoustic response originates solely from prompt thermalization of the absorbed energy. Based on the highly rigid structure of the phthalocyanines reported in previous bibliography and in our experimental data (vide infra), the possible structural (nonthermal) volume changes can be neglected while validating Equations (1)–(6) [32, 45, 60, 61]. Figure 3 exemplarily displays the dependence of ΔS on the absorbance‐normalized excitation energy (*E*) for the sample as well as for the reference (detected before and after measurement to verify consistency). A complete set of data, including results in water and DMSO, is found in the ESI.

**FIGURE 3:**
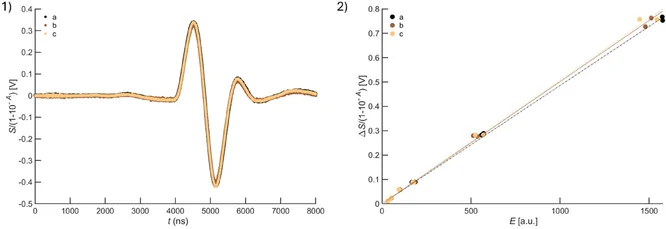
(1) Instrumentally averaged photoacoustic signal (*S*) in DMSO of the OHBP reference measured before the sample (a), of the sample **NiPc‐4** (b), and of OHBP acquired after the sample measurement (c). (2) Normalized ΔS in DMSO measured as a function of relative absorbed energy of the OHBP reference measured before the sample (a), of the sample **NiPc‐4** (b) and of OHBP acquired after the sample (c). Solid and dashed lines are the least‐squares linear fits through the data points of (a–c). The inset is the calculated *α*_s_ by dividing the slopes of the linear fits of (a) and (b).

The derived *α*_s_ values (*λ*_exc_ = 355 nm) for all NiPcs in DMSO and water are shown in Table 1. In DMSO, all the NiPcs exhibit an *α*_s_ ≈ 1 within the 95% confidence interval, demonstrating complete prompt heat release irrespective of *α*‐ or *β*‐substitution. This behavior is consistent with previous reports on Ni(II) phthalocyanines [62, 63, 64].

**TABLE 1:** *α*_s_ determined at *λ*_exc_ = 355 nm for NiPc‐1–4 in DMSO and water. The *α*_s_ in water is obtained from the average of two measurements with different fluences.

***α***_**s**_ of NiPc/solvent	NiPc‐1	NiPc‐2	NiPc‐3	NiPc‐4
DMSO	1.03 ± 0.03	1.01 ± 0.04	0.98 ± 0.02	1.03 ± 0.03
Water	1.04 ± 0.05	0.96 ± 0.08	0.93 ± 0.06	1.02 ± 0.05

For the NiPcs investigated in the present study, the absence of radiative decay and the efficient non‐radiative relaxation confirmed by TAS (vide infra) support the expectation that *α*_s_ should remain essentially independent of the excitation wavelength, including excitation into the lower energy absorption bands (i.e., the Q‐‍bands), suggesting that these compounds may represent promising candidates for photocalorimetric reference materials under far‐red/NIR excitation conditions.

Notably, the pressure waveforms show no evidence of delayed components or shape distortions relative to the reference signals, indicating that no longer‐lived excited states contribute to the photoacoustic response. This holds particularly true in aqueous media, where the temperature dependence of *K* is more pronounced, yet no significant deviations were observed. Therefore, under the present experimental conditions and at the working temperature in liquid aqueous solution, the photoacoustic response remains consistent with a predominantly thermal origin, with no significant contribution from nonthermal volume changes (e.g., photoinduced isomerization or decomposition). Moreover, the identical photoacoustic behavior observed between the NiPc samples and the calorimetric reference in both DMSO and aqueous solution further supports this conclusion, particularly when considering the lack of time shifts between samples and references.

### 2.5 Transient Absorption Studies

To further verify the ultrafast nonradiative relaxation of the NiPcs and the lack of transient or permanent photoproducts acting as energy sinks, TAS was performed in both DMSO and water. The measurements were designed to detect ground‐state bleaching, which would indicate the presence of longer‐lived excited species. However, no ground‐state depletion was observed for any of the complexes when exciting at *λ*_ex_ = 355 nm and probed across the Q‐ and B‐bands (see ESI). Importantly, no residual changes in the baseline were observed after excitation, indicating that the transient signal fully returns to the initial ground‐state level and that no long‐lived photoproducts or persistent excited‐state species accumulate under the experimental conditions. Given the temporal resolution of the instrumentation employed in this study (limited to lifetimes longer than ≈10 ns due to the pump pulse width), these results indicate that complete ground‐state recovery occurs within this time frame. Hence, the excited‐state lifetimes of the NiPcs are shorter than 10 ns, confirming that all absorbed energy is released as prompt heat, analogous to the behavior of the reference compounds [55, 65].

Additional measurements were carried out by exciting within the Q‐band and probing at both Q‐ and B‐band wavelengths. Once again, no ground‐state depletion was detected (see Section S8), confirming that the relaxation dynamics are independent of the excitation wavelength. Combined with the complete absence of fluorescence or phosphorescence upon Q‐band excitation, these results demonstrate that all NiPcs undergo rapid, radiationless deactivation and behave as quasi‐ideal photoacoustic calorimetric references.

## 3 Conclusion

In summary, we have developed and validated a straightforward and reliable methodology for the synthesis of a series of water‐ and solvent‐soluble octa‐substituted Ni(II) phthalocyanines, specifically tailored as PAS reference compounds. Their preparation, purification, and comprehensive structural characterization were achieved through a combination of MALDI–TOF mass spectrometry, NMR, and UV–vis spectroscopies, confirming their high purity and well‐defined substitution patterns.

Importantly, we introduced a reengineered and custom‐adapted spectroscopy platform that integrates PAS and TAS within a single instrument, enabling the simultaneous assessment of photothermal and excited‐state relaxation dynamics with a transient absorption spectrometer. The results unambiguously demonstrate that all NiPcs exhibit complete and prompt conversion of absorbed energy into heat (*α *≈ 1) in both aqueous and organic solvents without evidence of long‐lived excited states or emissive decay channels.

These findings establish Ni(II) phthalocyanines as stable, non‐emissive, and photocalorimetrically reliable standards for PAS, suitable for use across a broad spectral range from the UV to the NIR. Most importantly, they can be employed in a broad range of solvents, including water. Beyond their role as reference materials, their photostability and tunable absorption properties also position them as promising candidates for future optoacoustic and photothermal applications.

## Funding

This study was supported by the Deutsche Forschungsgemeinschaft (INST 211/1033‐1 FUGG; DFG EXC1003, CRC 1450‐431,460,824), Deutscher Akademischer Austauschdienst (doctoral fellowship for STNS), and Erasmus+ (Erasmus+ grant for NMMA).

## Conflicts of Interest

The authors declare no conflicts of interest.

## Supporting information

Supplementary Material

## Data Availability

The data that support the findings of this study are available in the Supporting Information of this article.
