# Supplementary material for: Synthesis and Photoacoustic Characterization of Amphiphilic Ni(II) Phthalocyanines as Calorimetric References
Source: Chemphyschem. 2026 Nov 5;27(21):e70561. doi: 10.1002/cphc.70561 (PMC13735987; doi:10.1002/cphc.70561)
Supplement: Supplementary file 1 [file CPHC-27-e70561-s001.pdf]

Synthesis and Photoacoustic Characterization of Amphiphilic Ni(II) Phthalocyanines as Calorimetric References

Sidharth Thulaseedharan Nair Sailaja[‡1,2], Niek M. M. Aarts[‡1,2], Iván Maisuls[1,2], Alexander Hepp[1], Andreas Faust[3,4] and Cristian A. Strassert*[1,2]

[1]Institut für Anorganische und Analytische Chemie, Universität Münster, Corrensstraße 28/30, 48149 Münster, Germany
[2]CeNTech, SoN, CiMIC, Universität Münster, Heisenbergstraße 11, 48149 Münster, Germany
[3]European Institute for Molecular Imaging, Röntgenstraße 16, 48149 Münster, Germany
[4]Department of Nuclear Medicine, University Hospital Münster, Albert-Schweitzer-Campus 1, 48149 Münster, Germany

email: Cristian A. Strassert* ca.s@uni-muenster.de
* corresponding author
‡ Equal contributors

Table of contents

Section S1: Materials and methods **3**

S1.1. Materials **3**

S1.2. NMR and mass spectrometry **3**

S1.3. Absorption and fluorescence spectroscopic techniques **3**

Section S2: Synthesis and characterization **4**

Section S3: NMR spectra **13**

Section S4: Mass spectra **43**

Section S5: UV-vis absorption studies of NiPcs **48**

Section S6: Experimental setup of TAS and PAS **57**

Section S7: Photoacoustic spectra and linear fits **58**

Section S8: Comparison between mechanical averaging and Savitsky-Golay smoothening **69**

Section S9: Transient absorption spectroscopy data **70**

Section S10: References **84**

Section S1: Materials and methods

All chemicals were purchased with the maximum quality available from Sigma Aldrich or TCI Chemicals and used without further purification. Reactions were carried out using dried solvents (99.9% purity) under argon atmosphere. They were monitored by thin-layer chromatography (TLC), which was performed on 0.2 mm Macherey-Nagel ALUGRAM precoated silica gel aluminum sheets. Spots were visualized by an UV-handlamp (254 and 365 nm). Silica gel 60 (0.063 – 0.200 mm, Merck) was used for column chromatography, if not otherwise stated.

NMR spectra were obtained at the Institut für Anorganische und Analytische Chemie (Universität Münster), using a Bruker Avance Neo 500 and a Bruker Avance III 400. All measurements were performed at room temperature unless mentioned otherwise. The ^{1}H-NMR and ^{13}C-NMR chemical shifts (δ) of the signals are given in parts per million and are referenced to the residual proton signal in the deuterated solvent DCM-d_2 (^{1}H: 5.32 ppm / ^{13}C: 54.0 ppm), DMF-d_7 (^{1}H: 8.03 ppm, 2.92 ppm, 2.75 ppm / ^{13}C: 163.15 ppm, 34.89 ppm, 29.76 ppm) or dimethyl sulfoxide-d_6 (^{1}H: 2.50 ppm / ^{13}C: 39.52 ppm). The signal multiplicities are abbreviated as follows: s, singlet; d, doublet; t, triplet; q, quartet; br, broad; m, multiplet.
Exact mass (EM) determination by mass spectrometry (MS) was carried out at the Organisch-Chemisches Institut (Universität Münster) using a LTQ Orbitap LTQ XL (Thermo-Fisher Scientific, Bremen) with electron spray injection (ESI). MALDI-TOF mass spectra were taken on Autoflex Speed MALDI-TOF spectrometer with 2-[(2*E*)-3-(4-*tert*-butylphenyl)-2-methylprop-2-enylidene]malonitrile (DCTB) as the matrix. Absorption spectra were measured using quartz (Hellma®) square cuvettes on a Shimadzu UV-3600i Plus UV-Vis-NIR spectrophotometer and baseline-corrected. Phot

Section S2: Synthesis and characterization

Schemes 1 and 2 provide a comprehensive description of the synthetic procedure.

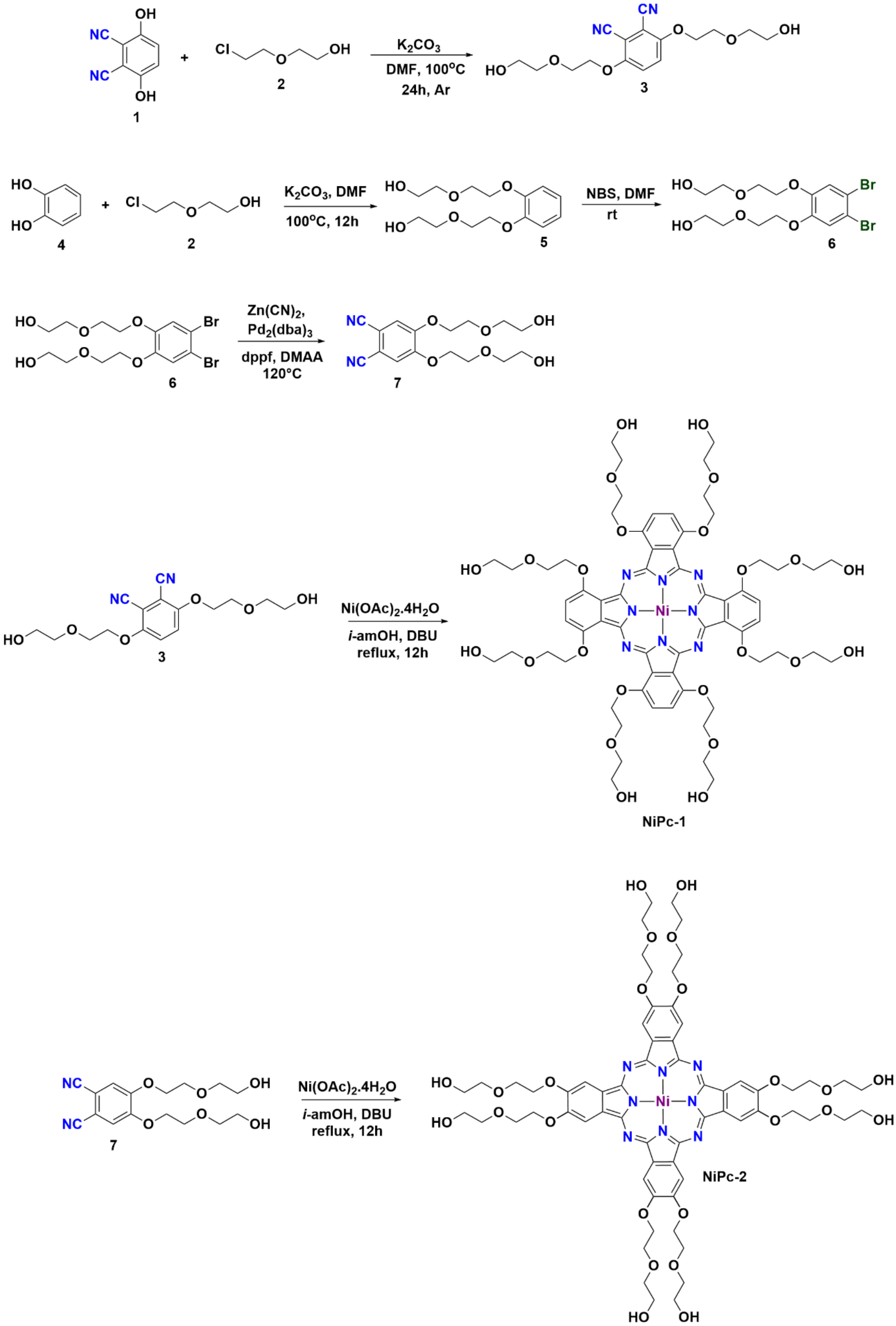

Scheme 1. Synthesis of **NiPc-1**, **NiPc-2**, and of the corresponding phthalonitrile precursors.

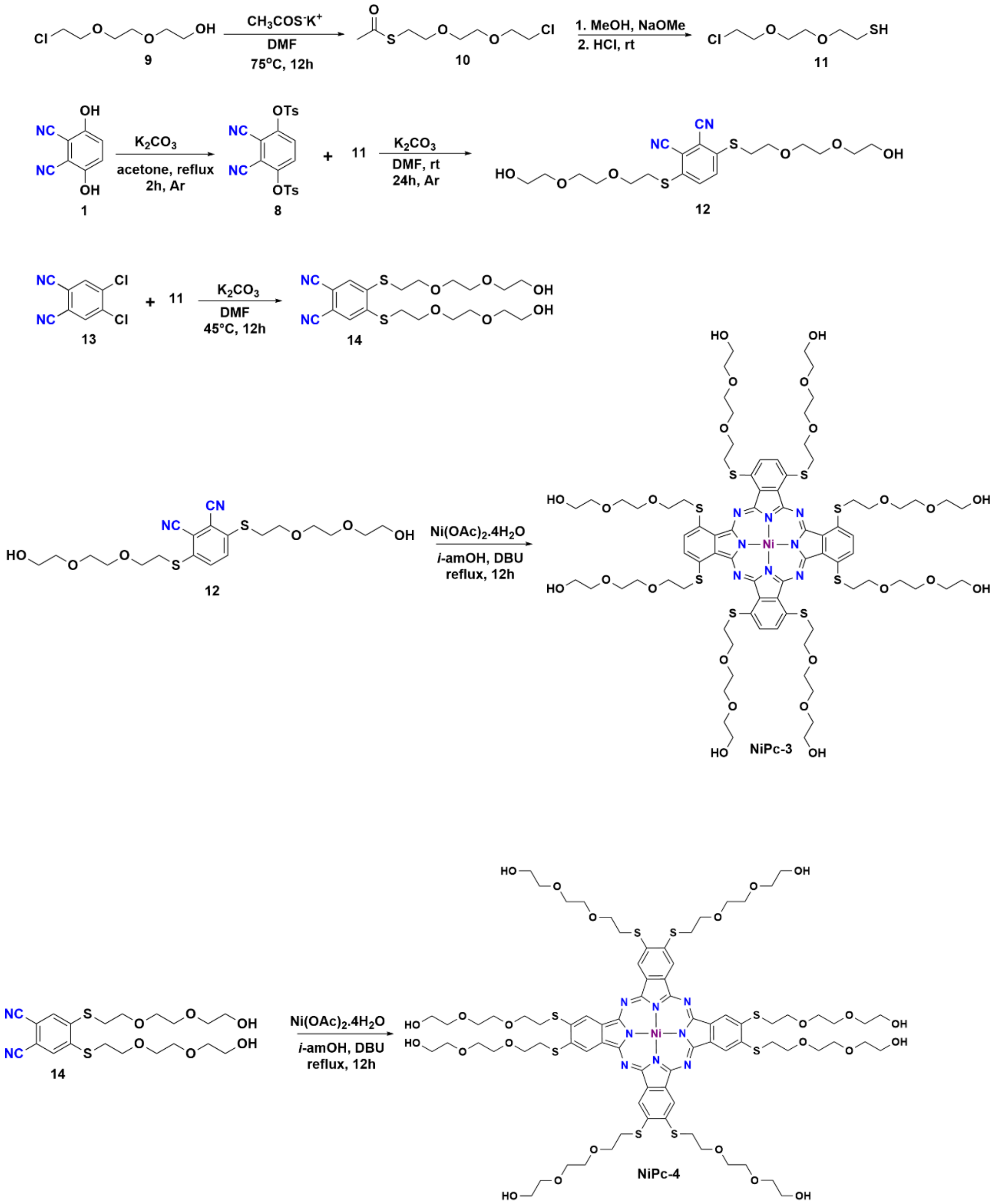

Scheme 2. Synthesis of **NiPc-3**, **NiPc-4**, and of the corresponding phthalonitrile precursors.

S2.1. 3,6-Bis(2-(2-hydroxyethoxy)ethoxy)phthalonitrile (3)

Compound **3** was prepared according to a reported procedure.[1,2] 2,3-dicyanohydroquinone (**1**) (1.00 g, 6.24 mmol), anhydrous K_2CO_3 (4.30 g, 31.20 mmol) and 2-(2-chloroethoxy)ethan-1-ol (**2**) (2.33 g, 18.72 mmol) were stirred in anhydrous *N,N*-dimethylformamide (DMF) (50 mL) at 100°C for 24h under argon atmosphere. The solvent was removed and the reaction mixture was then diluted with dichloromethane (100 mL) and extracted with distilled water (3 x 30 mL). The organic layer was dried over Na_2SO_4, filtered and concentrated. The oily product was purified over a silica gel column using dichloromethane:acetone mixture (9 : 1)

as eluent to obtain **3**. Molecular formula: $C_{16}H_{20}N_2O_6$ (pale yellow solid). Yield: 51% (1.07 g, 3.18 mmol).

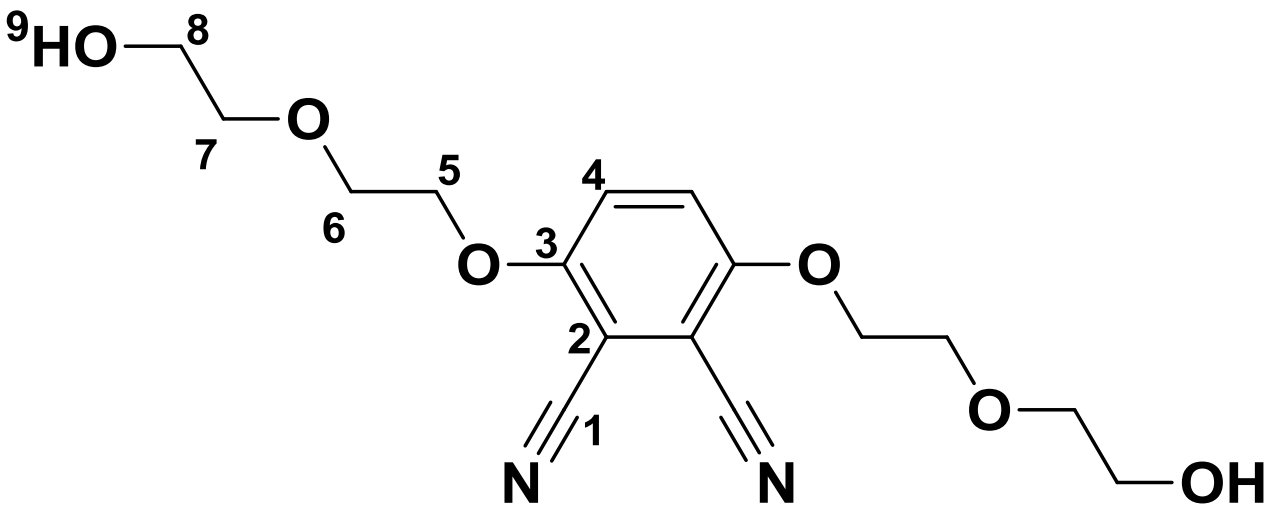

^{1}H-NMR (400 MHz, DMSO-*d*$_6$), δ (ppm) = 7.64 (s, 2H, 4-*H*), 4.60 (s, 2H, 9-*H*), 4.34 – 4.28 (m, 4H, 5-*H*), 3.80 – 3.74 (m, 4H, 6-*H*), 3.50 (pseudo s, 8H, 7,8-*H*).

^{13}C-{^{1}H}-NMR (101 MHz, DMSO-*d*$_6$), δ (ppm) = 154.91 (C-3), 120.76 (C-4), 113.48 (C-1), 102.93 (C-2), 72.46 (C-7), 69.63 (C-5), 68.55 (C-6), 60.12 (C-8).

MS-ESI-EM (CH_2Cl_2, M = $C_{16}H_{20}N_2O_6$): *m/z* calcd. for $[M+Na]^+$ = 359.12136; found for $[M+Na]^+$ = 359.12084.

S2.2. 1,2-bis(2-(2-hydroxyethoxy)ethoxy(benzene (5)

A reported procedure was adapted.[3,4] Catechol (**4**) (1.00 g, 9.10 mmol) was dissolved in DMF (300 mL) and K_2CO_3 (12.57 g, 0.91 mmol) and 2-(2-chloroethoxy)ethanol (**2**) (4.52 g, 36 mmol) were added. The mixture was heated at 80°C for 24h under argon atmosphere. The solvent was removed in vacuum. The residue was dissolved in chloroform (100 mL). The solution was washed with aqueous sodium hydroxide and water. The organic layer was dried over Na_2SO_4 and concentrated. The oily product was purified over a silica gel column using chloroform:acetone mixture (7:3) as eluent to obtain **5**. Molecular formula: $C_{14}H_{22}O_6$ (orange oil). Yield: 97% (2.52 g, 8.80 mmol).

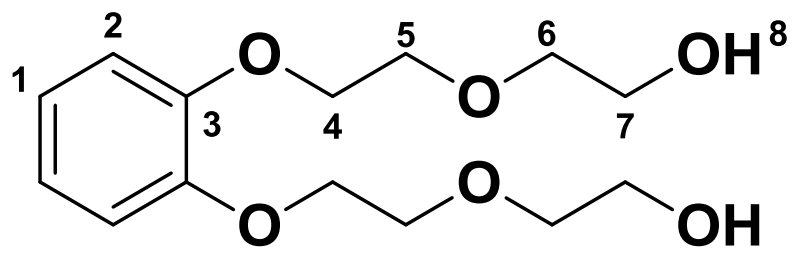

^{1}H-NMR (400 MHz, DCM-*d*$_2$), δ (ppm) = 6.91 (s, 4H, 1, 2-*H*), 4.14 – 4.07 (m, 4H, 4-*H*), 3.85 – 3.80 (m, 4H, 5-*H*), 3.74 (s, 2H, 8-*H*), 3.69 (s, 4H, 7-*H*), 3.64 – 3.59 (m, 4H, 6-*H*).

^{13}C-{^{1}H}-NMR (101 MHz, DCM-*d*$_2$), δ (ppm) = 148.93 (C-3), 121.67 (C-1), 114.32 (C-2), 72.93 (C-6), 69.58 (C-5), 68.86 (C-4), 61.63 (C-7).

MS-ESI-EM (CH_2Cl_2, M = $C_{14}H_{22}O_6$): *m/z* calcd. for $[M+Na]^+$ = 309.13086; found for $[M+Na]^+$ =309.13032.

S2.3. 1,2-dibromo-4,5-bis[2-(2-hydroxyethoxy)ethoxy]benzene (6)

Following a reported procedure,[4] diol **5** (3.66 g, 12.8 mmol) was dissolved in DMF (25 mL), and a solution of NBS (4.98 g, 28.2 mmol) in DMF (25 mL) was added dropwise. After stirring for 2 days, the yellow-orange solution was diluted with water (50 mL), solid Na_2SO_3 was added and the mixture was extracted with $CHCl_3$. The organic extracts were washed with water, dried over Na_2SO_4, and the solvent was

removed under reduced pressure. The product was purified over a silica gel column using acetone:cyclohexane mixture (7:2-2:8 (v/v)), yielding **6** as a viscous yellow oil. Molecular formula: $C_{14}H_{20}Br_2O_6$. Yield: 75% (4.42 g, 9.95 mmol).

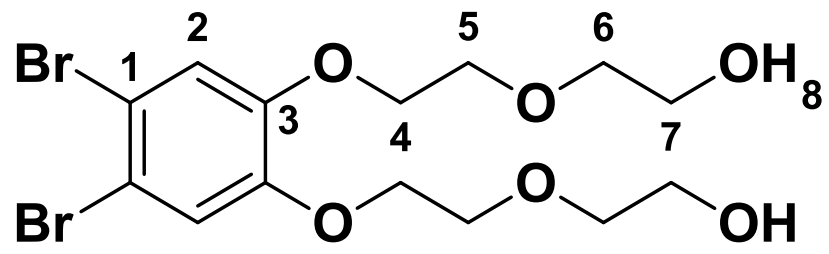

^{1}H-NMR (400 MHz, DCM-d_2), δ (ppm) = 7.12 (s, 2H, 2-*H*), 4.13 – 4.07 (m, 4H, 4-*H*), 3.86 – 3.82 (m, 4H, 5-*H*), 3.68 (m, 4H, 7-*H*), 3.61 (m, 4H, 6-*H*), 3.17 (s, 2H, 8-*H*).

^{13}C-{^{1}H}-NMR (101 MHz, DCM-d_2), δ (ppm) = 148.80 (C-3), 118.22 (C-2), 115.36 (C-1), 73.14 (C-6), 69.31 (C-4), 69.29 (C-5), 61.76 (C-7).

MS-ESI-EM (CH_2Cl_2, M = $C_{14}H_{20}Br_2O_6$): *m/z* calcd. for $[M+H]^+$ = 444.96798; found for $[M+H]^+$ = 444.96678, for $[M+Na]^+$ = 466.94821.

S2.4. 1,2-dicyano-4,5-bis[2-(2-hydroxyethoxy)ethoxy]benzene (7)

As reported in literature,[4] dibromide **6** (1.74 g, 3.92 mmol), $Zn(CN)_2$ (689 mg, 5.88 mmol) and dppf (60.8 mg, 0.11 mmol) were placed in a schlenk tube, 1,3-dimethylamylamine (DMAA) (5 mL) was added, and the mixture was flushed with argon. A degassed suspension of $Pd_2(dba)_3$ (71.8 mg, 78.4 µmol) in DMAA (5 mL) was added and the mixture was heated to 120°C for 2.5 h. After cooling to room temperature, the mixture was diluted with ethylacetate (20 mL) and filtered through a layer of silica. The combined filtrates were concentrated under reduced pressure to give a brown oil, which was further purified over a silica gel column using chloroform:methanol mixture (96:4) as eluent to obtain dinitrile **7** as a white powder. Molecular formula: $C_{16}H_{20}N_2O_6$. Yield: 85% (1.12 g, 3.33 mmol).

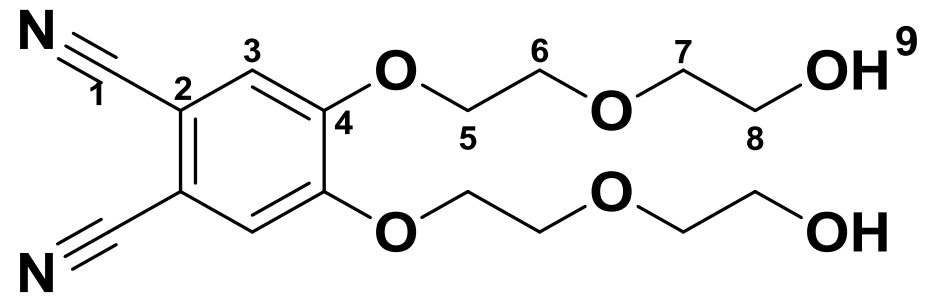

^{1}H-NMR (400 MHz, DMSO-d_6), δ (ppm) = 7.70 (s, 2H, 3-*H*), 4.61 – 4.53 (m, 2H, 9-*H*), 4.29 – 4.22 (m, 4H, 5-*H*), 3.79 – 3.72 (m, 4H, 6-*H*), 3.51 – 3.49 (m, 8H, 7,8-*H*).

^{13}C-{^{1}H}-NMR (101 MHz, DMSO-d_6), δ (ppm) = 151.73 (C-4), 117.18 (C-3), 116.22 (C-1), 107.41 (C-2), 72.49 (C-7), 69.09 (C-5), 68.41 (C-6), 60.18 (C-8).

MS-ESI-EM (CH_2Cl_2, M = $C_{16}H_{20}N_2O_6$): *m/z* calcd. for $[M+H]^+$ = 337.13941; found for $[M+H]^+$ = 337.13899.

S2.5. 3,6-bis(4’-methylphenylsulfonyloxy)phthalonitrile (8)

As previously reported,[5–7] toluenesulfonyl chloride (5.20 g, 27 mmol) was added to a mixture of 2,3-dicyanohydroquinone (**1**) (2 g,13 mmol) and potassium carbonate (6.9 g, 50 mmol) in acetone (15 ml). The mixture was heated to reflux for 2 h. Thin layer chromatography (TLC) was performed to determine the consumption of compound **1**. The mixture was cooled to room temperature, poured into water (40

ml), and stirred for 1 h. The light brown product was filtered and oven dried to obtain **8**. Molecular formula: $C_{22}H_{16}N_2O_6S_2$. Yield: 79% (4.5 g, 9.60 mmol).

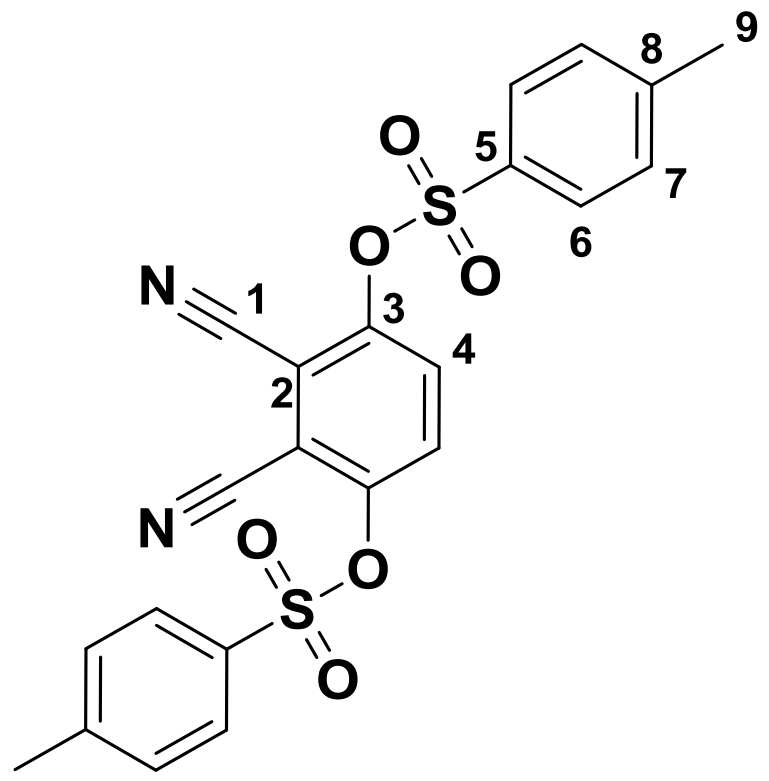

^{1}H-NMR (500 MHz, DCM-d_2), δ (ppm) = 7.80 (d, J = 8.6 Hz, 4H, 6-H), 7.71 (s, 2H, 4-H), 7.43 (d, J = 8.6 Hz, 4H, 7-H), 2.49 (s, 6H, 9-H).

^{13}C-{^{1}H}-NMR (126 MHz, DCM-d_2), δ (ppm) = 149.47 (C-3), 148.07 (C-8), 130.99 (C-7), 129.83 (C-4), 129.15 (C-6), 112.59 (C-2), 111.19 (C-1), 22.04 (C-9).

MS-ESI-EM (CH_2Cl_2, M = $C_{22}H_{16}N_2O_6S_2$): m/z calcd. for $[M+H]^+$ = 469.05225; found for $[M+H]^+$ = 469.05243.

S2.6. S-(2-(2-hydroxyethoxy)ethyl) ethanethioate (10)

Compound **10** was prepared by following a modified literature procedure.[8] 2-(2-(2-chloroethoxy)ethoxy)ethan-1-ol (1.05 g, 6.26 mmol, 1.00 eq.) and potassium thioacetate (1.76 g, 13.7 mmol, 2.20 eq.) are dissolved in dry DMF (20 mL). The resulting mixture was stirred at 75°C overnight. After evaporating the solvent under reduced pressure, the residue was diluted in ethyl acetate (20 mL) and water (20 mL). The combined organic layer was dried over Na_2SO_4, concentrated under reduced pressure and purified on a silica gel column using cyclohexane:ethyl acetate mixture (1:1 → ethyl acetate → ethyl acetate + 10% MeOH) to obtain **10** as a yellow oil. Molecular formula: $C_8H_{16}O_4S$. Yield: 51% (661 mg, 3.17 mmol).

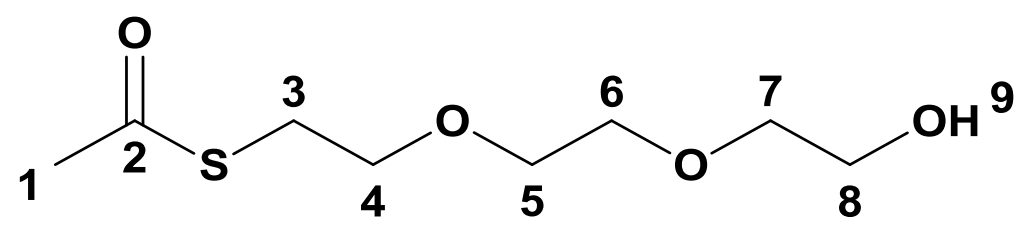

^{1}H-NMR (400 MHz, DCM-d_2), δ (ppm) = 3.67 – 3.62 (m, 2H, 8-H), 3.61 – 3.57 (m, 4H, 5,6-H), 3.55 (t, J = 6.5 Hz, 2H, 4-H), 3.55 – 3.52 (m, 2H, 7-H), 3.05 (t, J = 6.5 Hz, 2H, 3-H), 2.59 (t, J = 6.0 Hz, 1H, 9-H), 2.31 (s, 3H, 1-H).

^{13}C-{^{1}H}-NMR (101 MHz, DCM-d_2), δ (ppm) = 195.64 (C-2), 72.90 (C-7), 70.65 (C-5), 70.62 (C-6), 70.02 (C-4), 61.97 (C-8), 30.73 (C-1), 29.13 (C-3).

MS-ESI-EM (CH_2Cl_2, M = $C_8H_{16}O_4S$): m/z calcd. for $[M+Na]^+$ = 231.06615; found for $[M+Na]^+$ = 231.06607.

S2.7. 2-(2-(2-sulfanylethoxy)ethoxy)ethan-1-ol (11)

Compound **11** was prepared by dissolving **10** (154 mg, 0.67 mmol, 1.00 eq.) in methanol (5 mL) which was bubbled through with argon for 0.5 h prior to use.

Sodium methoxide (109 mg, 2.01 mmol, 3.00 eq.) was added and the resulting mixture was stirred for 30 min at room temperature. After acidifying the mixture with 1 M HCl (5 mL) the mixture was extracted with ethyl acetate (3x). The combined organic layer was dried over Na_2SO_4 and the solvent was evaporated under reduced pressure to obtain **11** as colorless oil. The obtained product was used for next steps without further purifications. Molecular formula: $C_6H_{14}O_3S$; yield: 99% (110 mg, 0.66 mmol).

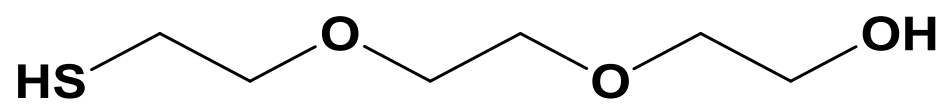

MS-ESI-EM (CH_2Cl_2, M = $C_6H_{14}O_3S$): *m/z* calcd. for $[M+Na]^+$ = 189.05559; found for $[M+Na]^+$ = 189.05568.

S2.8. 3,6-bis((2-(2-(2-hydroxyethoxy)ethoxy)ethyl)thio)phthalonitrile (12)

3,6-Bis(4'-methylphenylsulfonyloxy)phthalonitrile (**8**) (1 g, 2.13 mmol) was dissolved in dry DMF (30 mL) in the presence of dry K_2CO_3 (885 mg, 6.40 mmol) under argon atmosphere at room temperature for 35 min. A solution of compound **11** (1 g, 6.39 mmol) in dry DMF (10mL) was added and stirred at room temperature overnight. The reaction mixture was evaporated under reduced pressure and then mixed with dichloromethane: water mixture. Organic phase was dried over Na_2SO_4 and the solvent was evaporated under reduced pressure. The obtained product was further purified by silica column chromatography using dichloromethane:acetone mixture (9:1) as eluent to obtain **12** as white powder. Molecular formula: $C_{20}H_{28}N_2O_6S_2$. Yield: 40% (390 mg, 0.85 mmol).

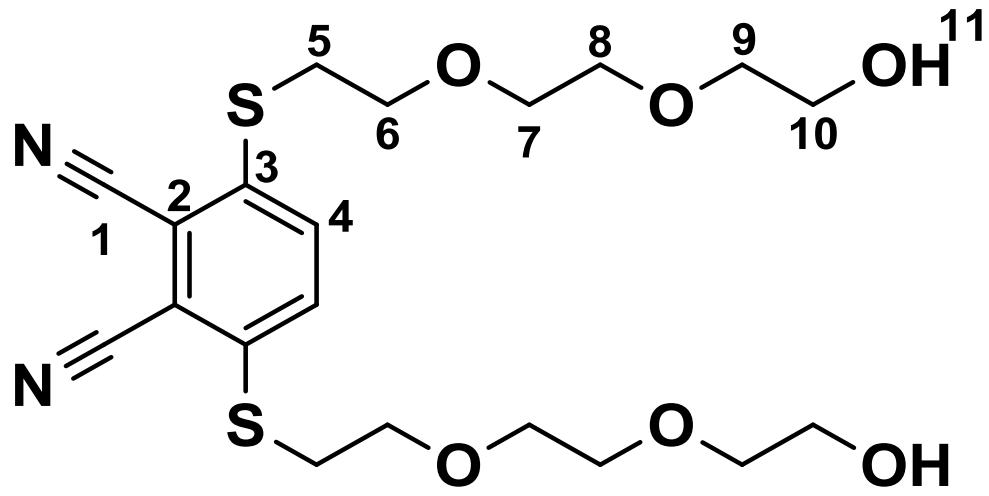

^{1}H-NMR (400 MHz, DMSO-d_6), δ (ppm) = 7.90 (s, 2H, 4-*H*), 4.56 (s, 2H, 11-*H*), 3.66 (t, *J* = 6.1 Hz, 4H, 6-*H*), 3.54 – 3.51 (m, 4H, 7-*H*), 3.49 – 3.48 (m, 4H, 10-*H*), 3.47 – 3.45 (m, 4H, 8-*H*), 3.43 – 3.38 (m, 4H, 9-*H*), 3.41 – 3.34 (m, 4H, 5-*H*).

^{13}C-{^{1}H}-NMR (101 MHz, DMSO-d_6), δ (ppm) = 140.48 (C-3), 132.89 (C-4), 114.91 (C-2), 114.22 (C-1), 72.27 (C-9), 69.65 (C-7), 69.61 (C-8), 68.71 (C-6), 60.11 (C-10), 32.49 (C-5).

MS-ESI-EM (CH_2Cl_2, M = $C_{20}H_{28}N_2O_6S_2$): *m/z* calcd. for $[M+Na]^+$ = 479.12810; found for $[M+Na]^+$ = 479.12651.

S2.8. 4,5-bis((2-(2-(2-hydroxyethoxy)ethoxy)ethyl)thio)phthalonitrile (14)

4,5-Dichlorophthalonitrile (**13**) (0.45 g, 2.3 mmol) and compound **11** (952 mg, 5.04 mmol) were dissolved and stirred with dry DMF (50 mL) containing excess amount of dry K_2CO_3 (1.11 g, 8.05 mmol) under argon atmosphere at 45°C for overnight. The reaction process was checked by TLC [silica gel (acetone:hexane)(60:40)]. After cooling to room temperature, the reaction mixture was filtered off and

evaporated to dryness under reduced pressure. Purification of the crude product was carried out by column chromatography on silica gel using acetone:hexane mixture (60:40) as eluent to give compound **14** as a colourless solid. Molecular formula: $C_{20}H_{28}N_2O_6S_2$. Yield: 62% (646 mg, 1.41 mmol).

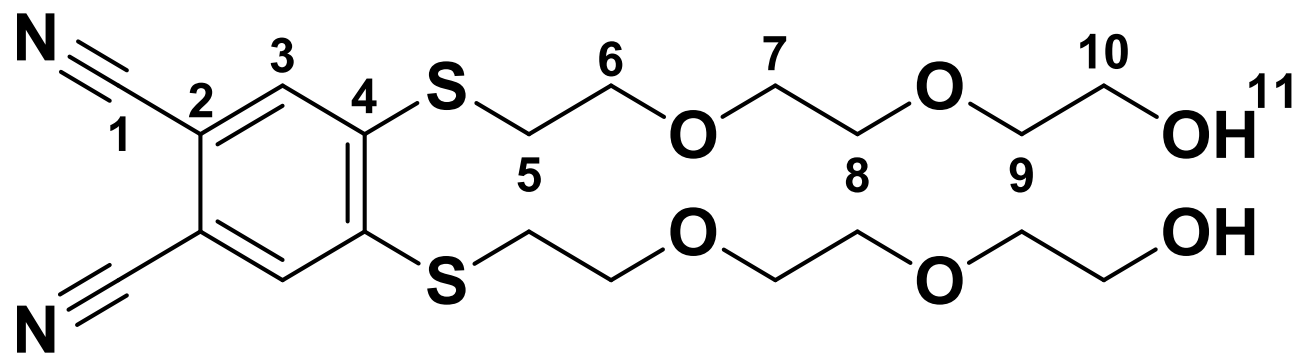

^{1}H-NMR (400 MHz, $CDCl_3$), δ (ppm) = 7.62 (s, 2H, 3-*H*), 3.77 (t, J = 6.1 Hz, 4H, 6-*H*), 3.71 – 3.68 (m, 4H, 10-*H*), 3.63 (s, 8H, 7,8-*H*), 3.58 – 3.55 (m, 4H, 9-*H*), 3.22 (t, J = 6.1 Hz, 4H, 5-*H*), 2.58 (s, 2H, 11-*H*).

^{13}C-{^{1}H}-NMR (101 MHz, $CDCl_3$), δ (ppm) = 143.91 (C-4), 129.52 (C-3), 115.47 (C-1), 111.37 (C-2), 72.45 (C-9), 70.58 (C-7), 70.20 (C-8), 69.17 (C-6), 61.58 (C-10), 32.67 (C-5).

MS-ESI-EM (CH_2Cl_2, M = $C_{20}H_{28}N_2O_6S_2$): *m/z* calcd. for $[M+Na]^+$ = 479.12810; found for $[M+Na]^+$ = 479.12719.

S2.9. NiPc-1

3,6-Disubstituted phthalonitrile (**3**) (0.20 g, 0.59 mmol), nickel acetate tetrahydrate (0.074 g, 0.29 mmol) and catalytic amount of DBU was added in 10 mL *i*-amOH (isoamyl alcohol) and were stirred at reflux in a SCHLENK apparatus under argon atmosphere for 12 h. After cooling to room temperature, the reaction mixture was evaporated to dryness under vacuum and dissolved in 10mL of methanol. The product was purified by employing a size exclusion chromatography using a resin (Sephadex-LH20) in methanol. The collected fractions were concentrated to obtain the **NiPc-1**. Molecular formula: $C_{64}H_{80}N_8NiO_{24}$ (green solid). Yield: 14% (116 mg, 0.08 mmol).

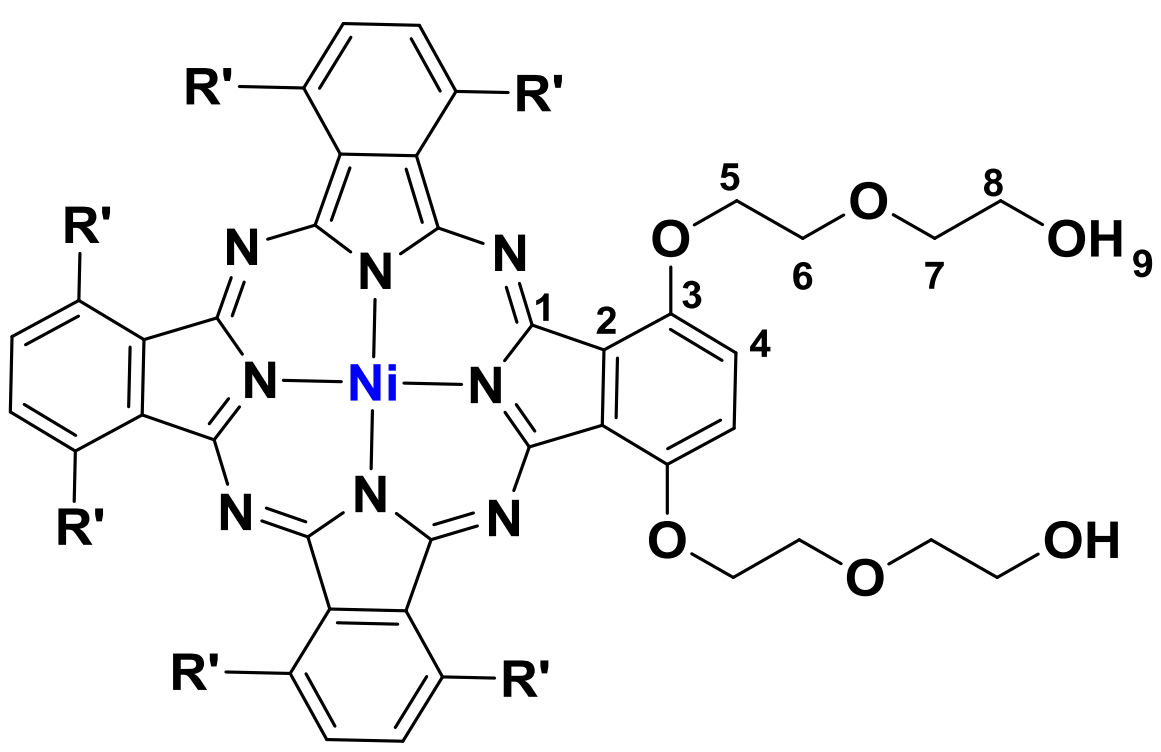

^{1}H-NMR (400 MHz, DMF-d_7), δ (ppm) = 7.79 (s, 8H, 4-*H*), 5.03 (t, *J* = 5.0 Hz, 16H, 5-*H*), 4.64 – 4.59 (m, 8H, 9-*H*), 4.18 (t, *J* = 5.0 Hz, 16H, 6-*H*), 3.72 – 3.70 (m, 16H, 7-*H*), 3.68 – 3.64 (m, 16H, 8-*H*).

^{13}C-{^{1}H}-NMR (101 MHz, DMF-d_7), δ (ppm) = 151.36 (C-3), 145.77 (C-1), 127.63 (C-2), 120.08 (C-4), 73.67 (C-7), 72.12 (C-5), 70.54 (C-6), 61.69 (C-8).

MALDI-TOF-MS: (CH_2Cl_2, M = $C_{64}H_{80}N_8NiO_{24}$): *m/z* calcd. for $[M]^+$ = 1402.46; found for $[M]^+$= 1402.24.

S2.10. NiPc-2

4,5-Disubstituted phthalonitrile (**7**) (0.30 g, 0.89 mmol), nickel acetate tetrahydrate (0.110 g, 0.44 mmol) and catalytic amount of DBU were added in 10 mL *i*-amOH (isoamyl alcohol) and stirred at reflux in a SCHLENK apparatus under argon atmosphere for 12 h. After cooling to room temperature, the reaction mixture was evaporated to dryness under vacuum and dissolved in 10 mL of methanol. The product was purified by employing a size exclusion chromatography using a resin (Sephadex-LH20) in methanol. The collected fractions were concentrated to obtain the **NiPc-2**. Molecular formula: $C_{64}H_{80}N_8NiO_{24}$ (green solid). Yield: 18% (225 mg, 0.16 mmol).

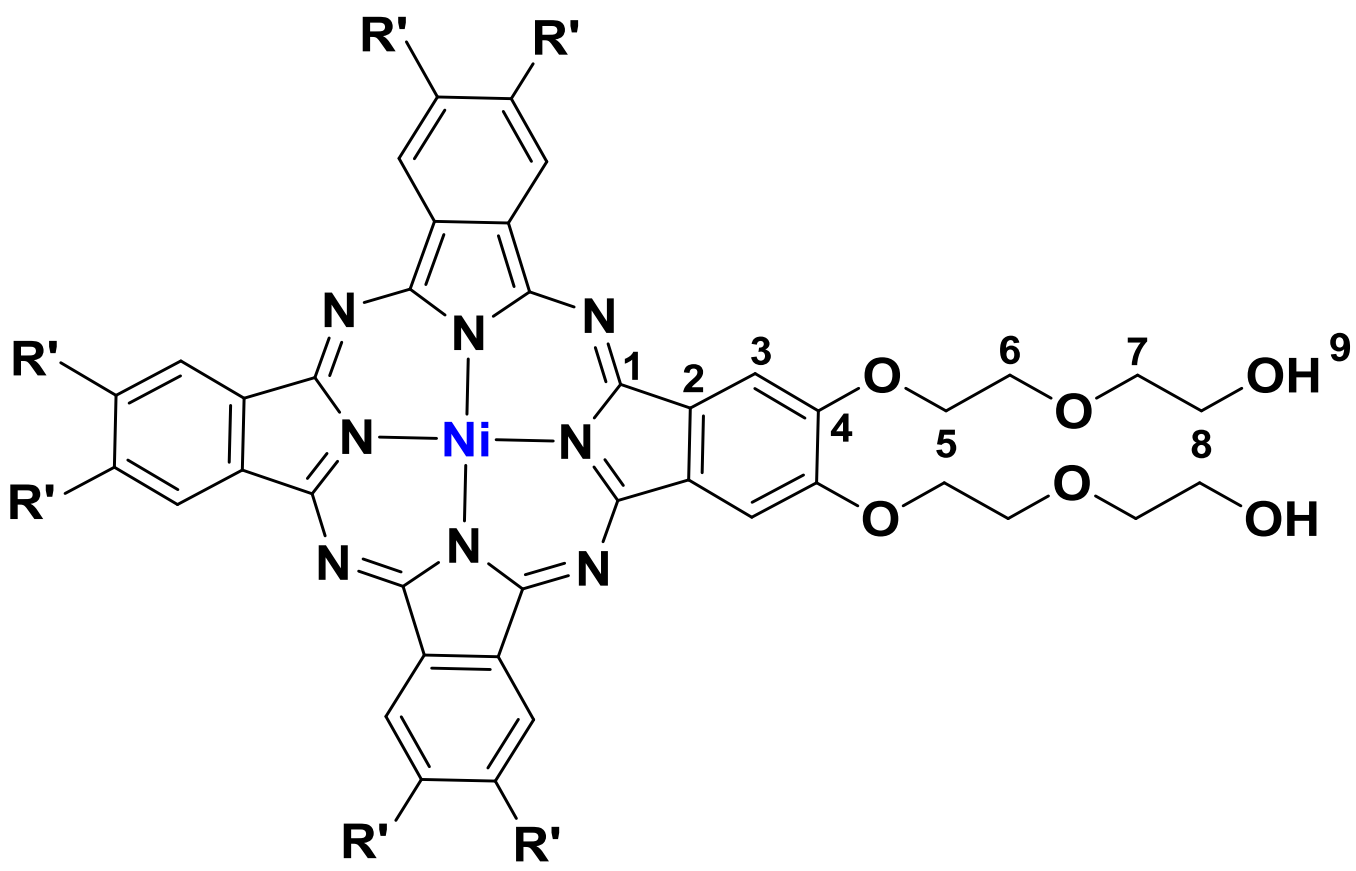

^{1}H-NMR (500 MHz, DMSO-d_6, 350 K), δ (ppm) = 7.87 (br, s, 8H, 3-*H*), 4.58 (br, s, 16H, 5-*H*), 4.48 (br, s, 8H, 9-*H*), 4.21 (br, s, 16H, 6-*H*), 3.90 – 3.77 (m, 32H, 7,8-*H*).

^{13}C-{^{1}H}-NMR (126 MHz, DMSO-d$_6$, 350K), δ (ppm) = 150.26 (C-4), 142.20 (C-1), 129.03 (C-2), 103.74 (C-3), 72.45 (C-7), 68.97 (C-5), 68.47 (C-6), 60.26 (C-8).

MALDI-TOF-MS: (CH_2Cl_2, M = $C_{64}H_{80}N_8NiO_{24}$): *m/z* calcd. for $[M]^+$ = 1402.46; found for $[M+H]^+$ = 1403.62.

S2.11. NiPc-3

3,6-Disubstituted phthalonitrile (**12**) (0.20 g, 0.44 mmol), nickel acetate tetrahydrate (0.055 g, 0.22 mmol) and catalytic amount of DBU were added in 10 mL *i*-amOH (isoamyl alcohol) and stirred at reflux in a SCHLENK apparatus under argon atmosphere for 12 h. After cooling to room temperature, the reaction mixture was evaporated to dryness under vacuum and dissolved in 10 mL of methanol. The product was purified by employing a size exclusion chromatography using a resin (Sephadex-LH20) in methanol. The collected fractions were concentrated to obtain the **NiPc-3**. Molecular formula: $C_{80}H_{112}N_8NiO_{24}S_8$ (brown oil). Yield: 10% (82 mg, 0.04 mmol).

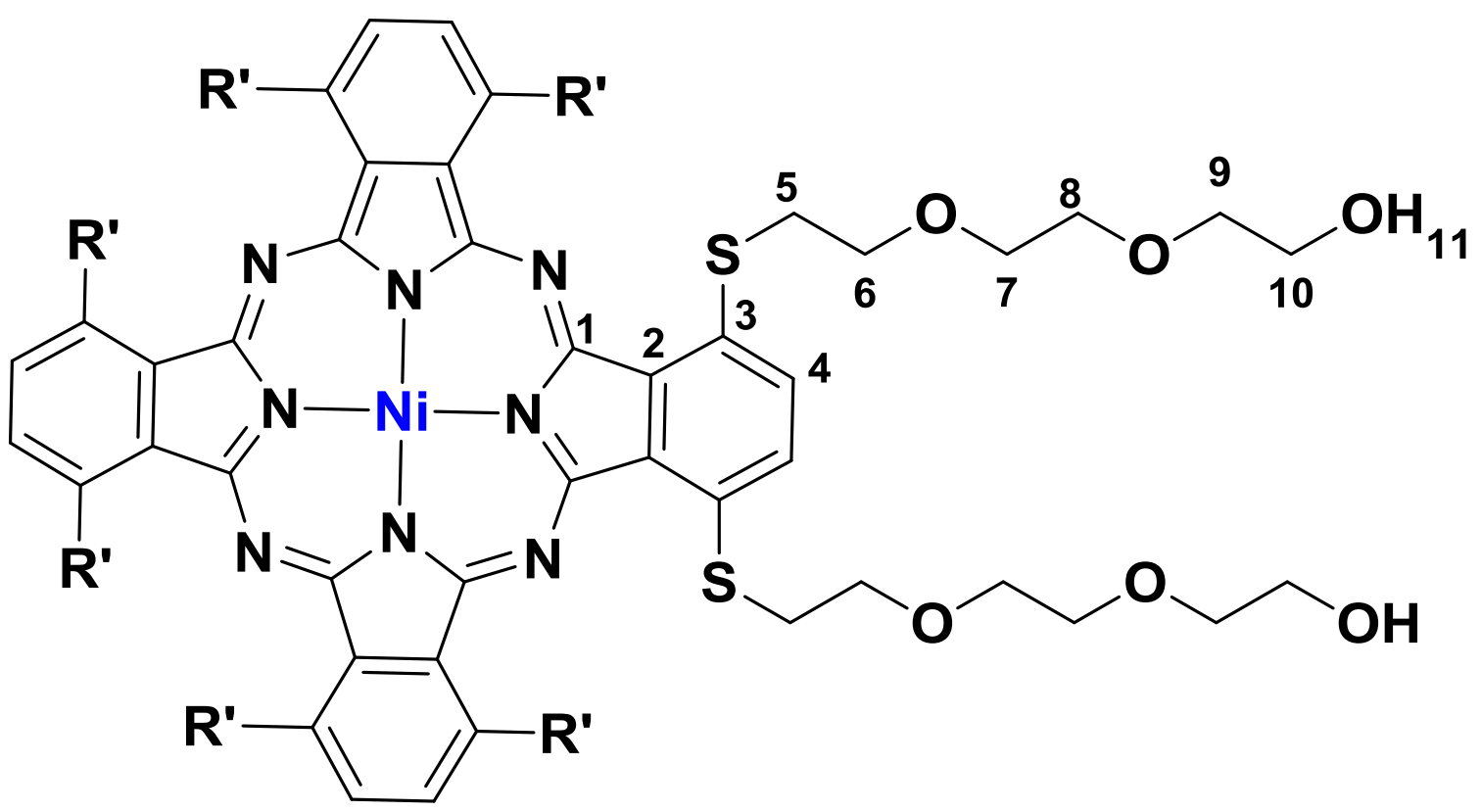

^{1}H-NMR (400 MHz, DMF-d_7), δ (ppm) = 7.58 (s, 8H, 4-*H*), 4.62 (s, 8H, 11-*H*), 3.88 (t, *J* = 7.0 Hz, 16H, 6-*H*), 3.76 – 3.74 (m, 16H, 7-*H*), 3.71 – 3.69 (m, 16H, 8-*H*), 3.66 (br, m, 16H, 10-*H*), 3.60 – 3.57 (m, 16H, 9-*H*), 3.39 (br, s, 16H, 5-*H*).

^{13}C-{^{1}H}-NMR (101 MHz, DMF-d_7), δ (ppm) = 145.73 (C-1), 132.92 (C-2), 131.71 (C-3), 124.99 (C-4), 73.56 (C-9), 71.00 (C-8), 70.85 (C-7), 70.02 (C-6), 61.68 (C-10), 31.58 (C-5).

MALDI-TOF-MS: (CH_2Cl_2, M = $C_{80}H_{112}N_8NiO_{24}S_8$): *m/z* calcd. for $[M]^+$ = 1884.98; found for $[M+Na]^+$ =1907.74, for $[M]^+$ = 1884.31, for $[M-(OCH_2CH_2)]^+$ = 1840.19, for $[M-(OCH_2CH_2)_2]^+$ = 1796.84.

S2.12. NiPc[β-S($CH_2CH_2O)_3H]_8$ (NiPc-4)

4,5-Disubstituted phthalonitrile (**14**) (0.30 g, 0.65 mmol), nickel acetate tetrahydrate (0.082 g, 0.33 mmol) and catalytic amount of DBU were added in 10 mL *i*-amOH (isoamyl alcohol) and stirred at reflux in a SCHLENK apparatus under argon atmosphere for 12 h. After cooling to room temperature, the reaction mixture was evaporated to dryness under vacuum and dissolved in 10mL of methanol. The product was purified by employing a size exclusion chromatography using a resin (Sephadex-LH20) in methanol. The collected fractions were concentrated to obtain the **NiPc-4**. Molecular formula: $C_{80}H_{112}N_8NiO_{24}S_8$ (green solid). Yield: 19% (235 mg, 0.12 mmol).

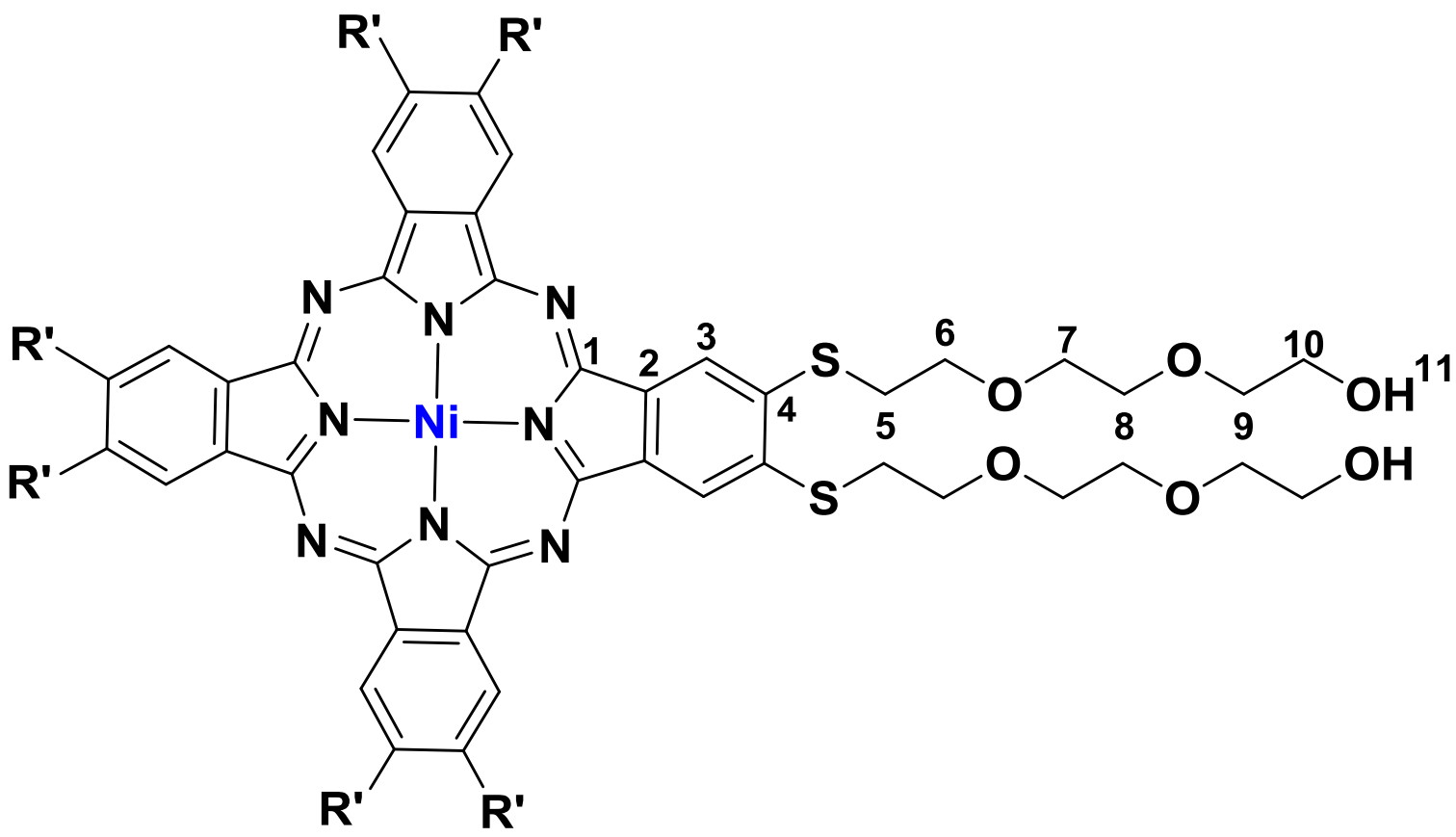

^{1}H-NMR (500 MHz, DMSO-d_6, 360 K), δ (ppm) = 8.20 (br, s, 8H, 3-*H*), 4.16 (br, s, 8H, 11-*H*), 4.07 (br, s, 16H, 6-*H*), 3.81 (br, s, 16H, 7-*H*), 3.72 (br, s, 16H, 8-*H*), 3.54 (br, m, 48H, 5,9,10-*H*).

^{13}C-{^{1}H}-NMR (126 MHz, DMSO-d_6, 360 K), δ (ppm) = 142.71 (C-1), 138.68 (C-4), 132.31 (C-2), 119.16 (C-3), 71.97 (C-9), 69.58 (C-7), 69.44 (C-8), 68.67 (C-6), 59.93 (C-10), 33.09. (C-5).

MALDI-TOF-MS: (CH_2Cl_2, M = $C_{80}H_{112}N_8NiO_{24}S_8$): *m/z* calcd. for $[M]^+$ = 1884.98; found for $[M+Na]^+$ = 1907.84, $[M]^+$ = 1884.96, $[M-(OCH_2CH_2)]^+$ = 1840.82.

Section S3: NMR spectra

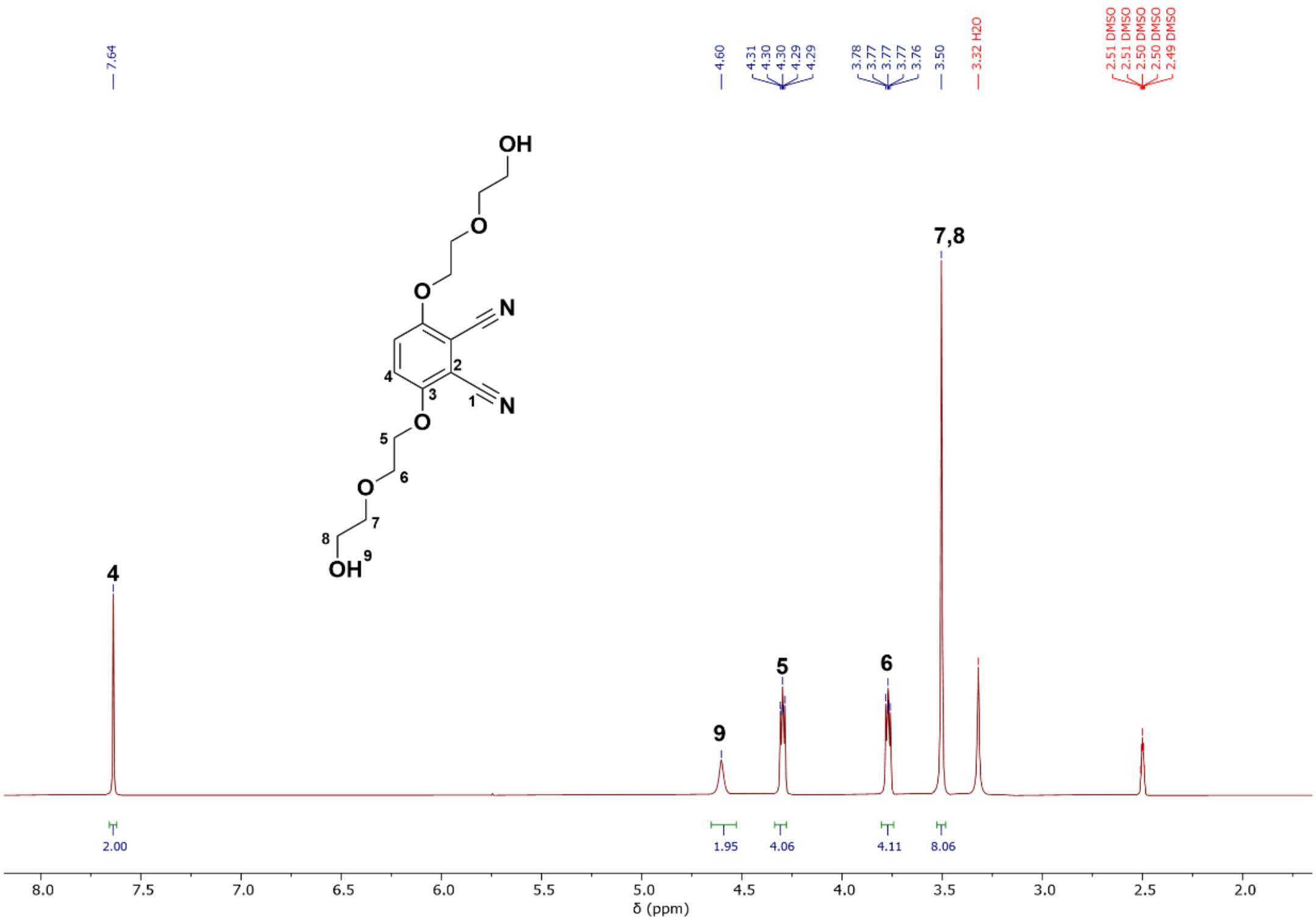

Figure S1: ^{1}H-NMR spectrum (400 MHz, DMSO-d_6) of **3**.

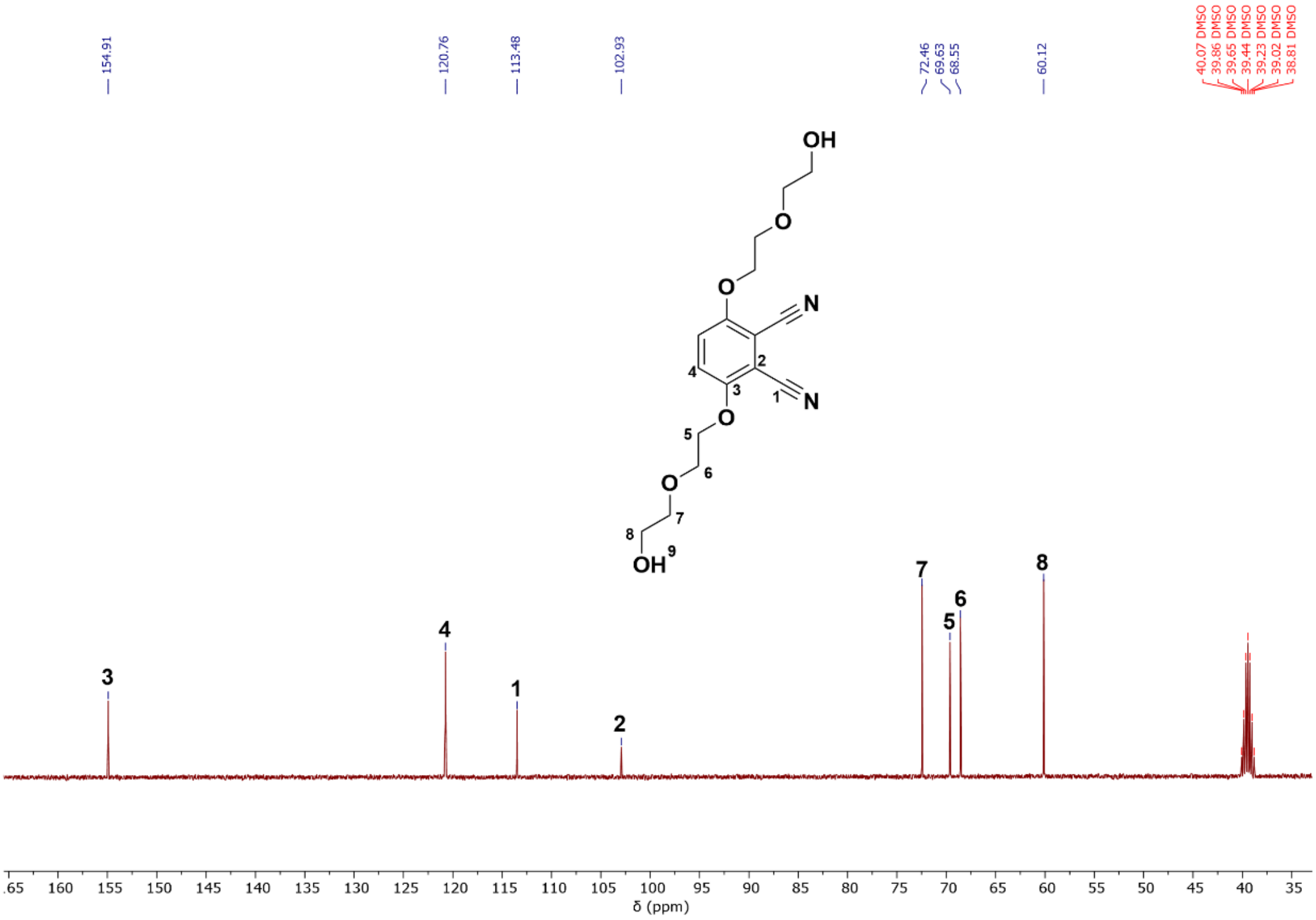

Figure S2: ^{13}C-{^{1}H}-NMR spectrum (101 MHz, DMSO-d_6) of **3**.

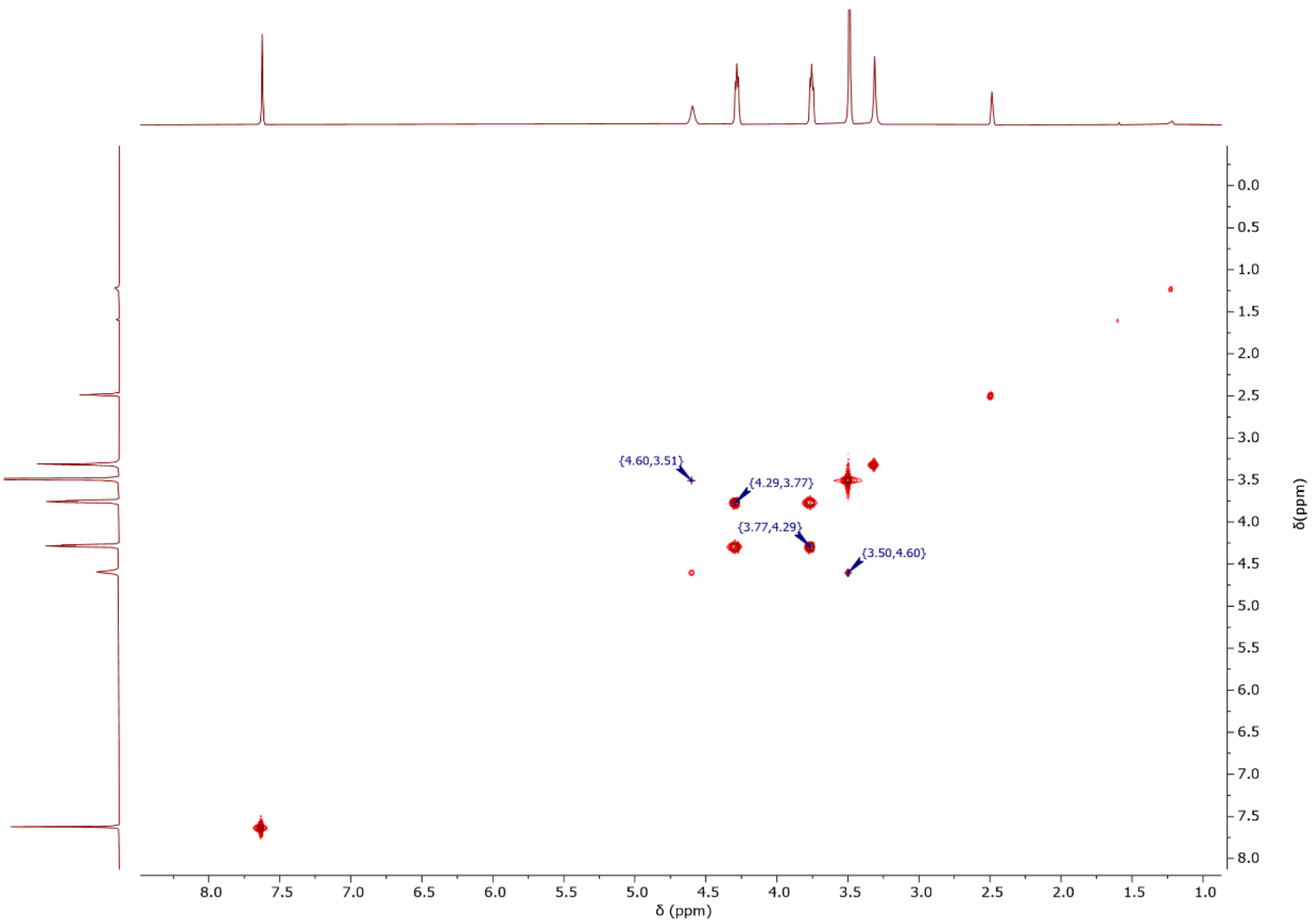

Figure S3: ^{1}H-^{1}H-COSY-NMR spectrum (400 MHz, DMSO-d_6) of **3**.

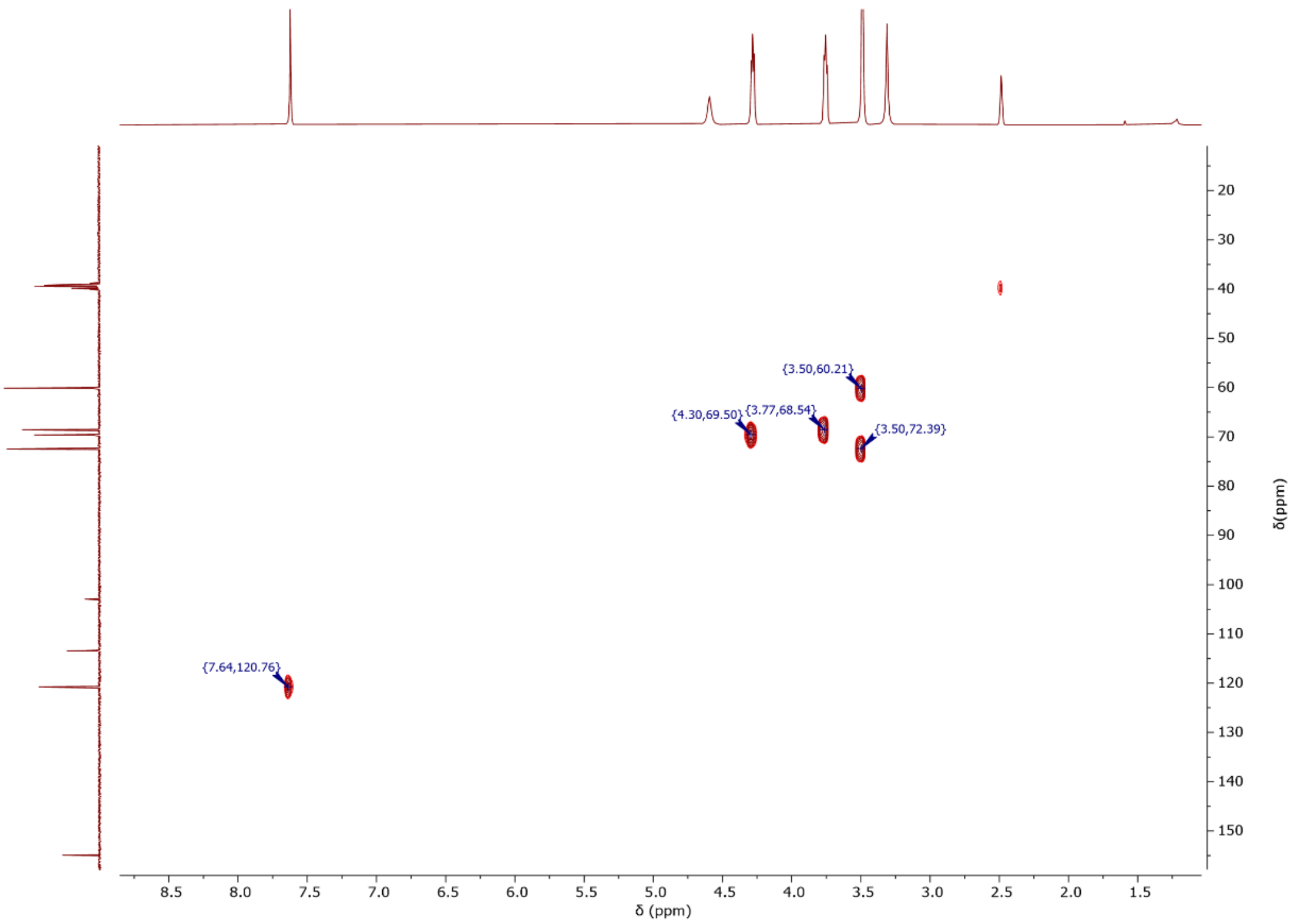

Figure S4: $^{1}H/^{13}C$-gHSQC-NMR spectrum (400 MHz/101 MHz, DMSO-d_6) of **3**.

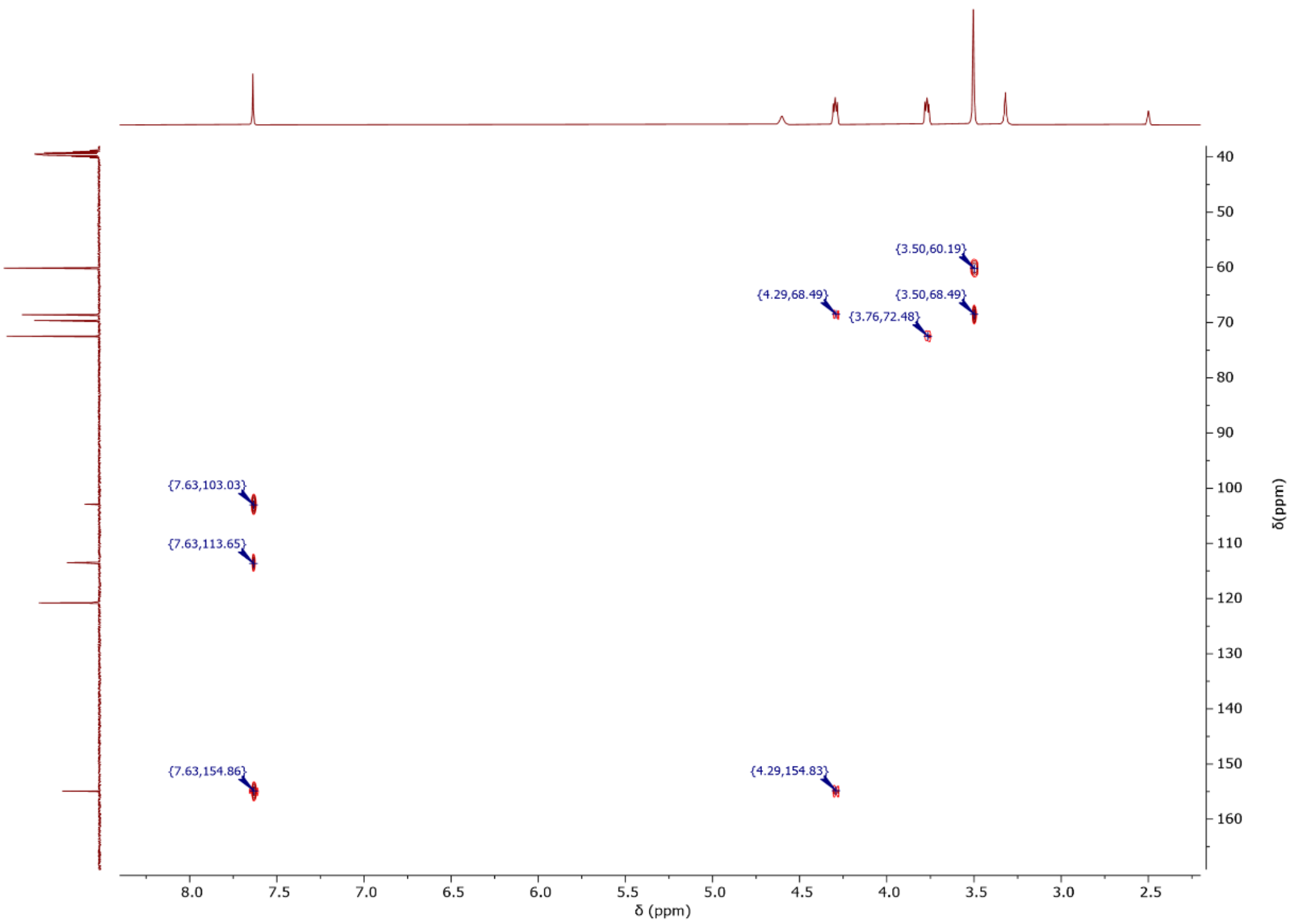

Figure S5: $^{1}H/^{13}C$-gHMBC-NMR spectrum (400 MHz/101 MHz, DMSO-d_6) of **3**.

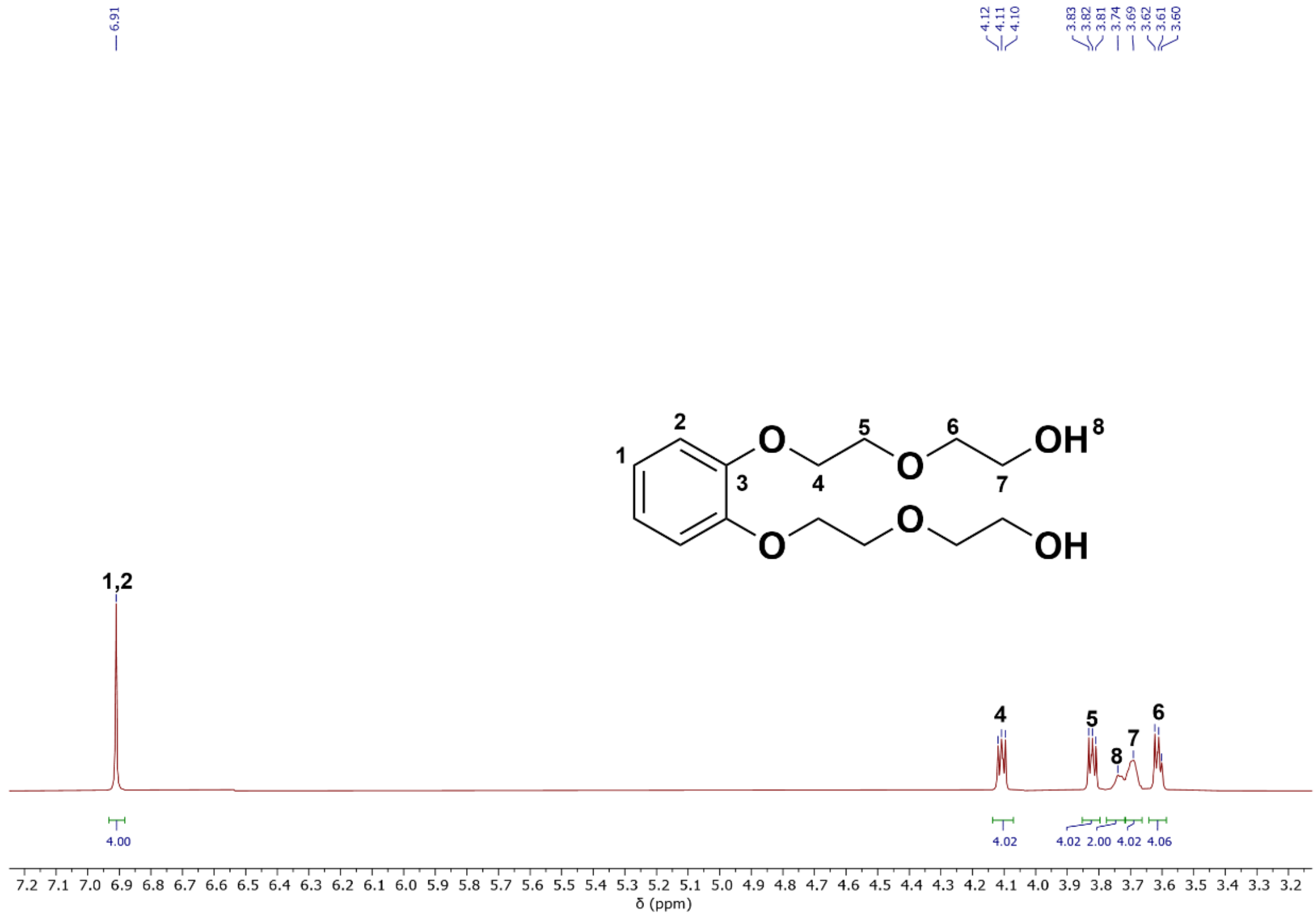

Figure S6: ^{1}H-NMR spectrum (400 MHz, DCM-d_2) of **5**.

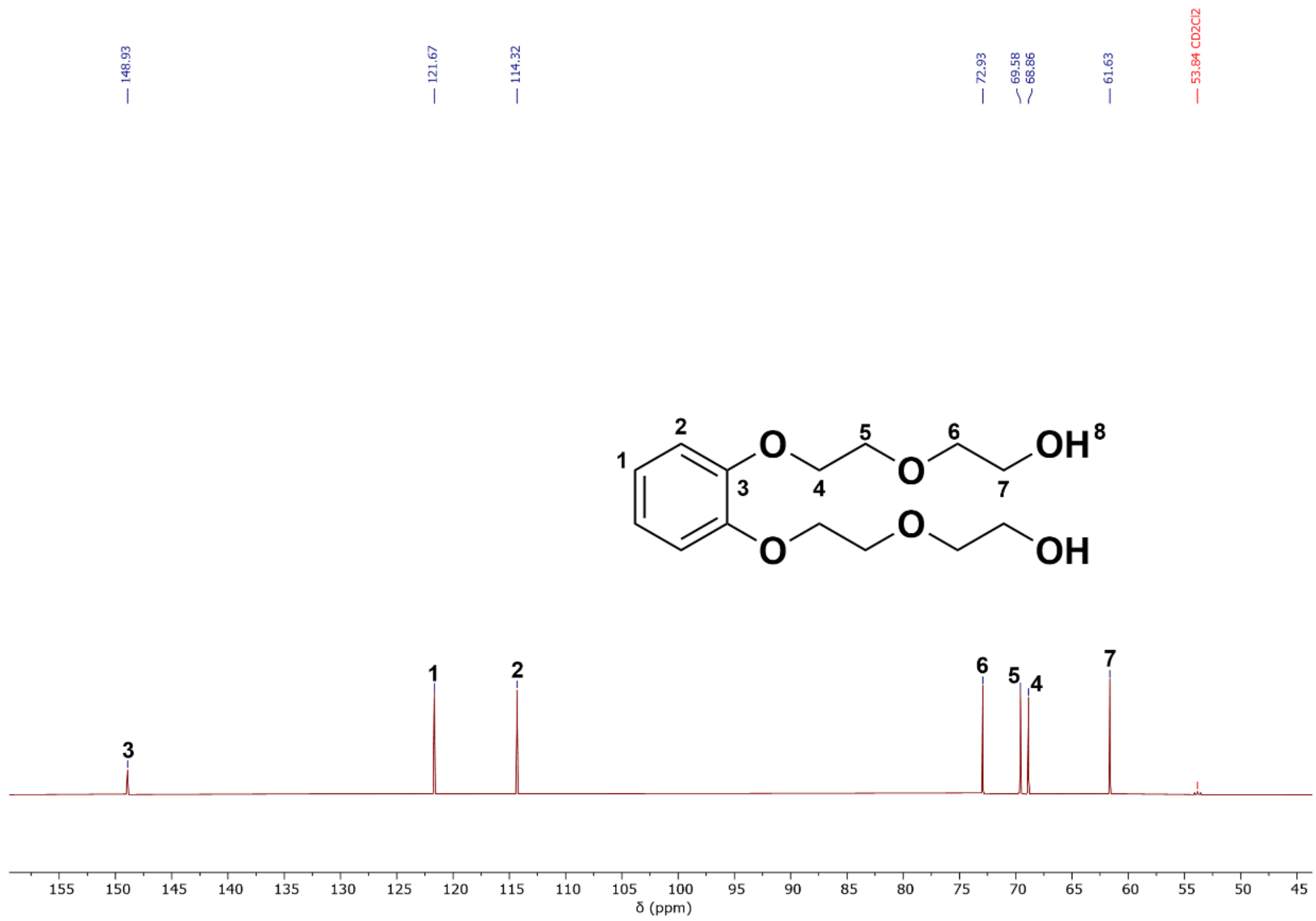

Figure S7: ^{13}C-{^{1}H}-NMR spectrum (101 MHz, DCM-d_2) of **5**.

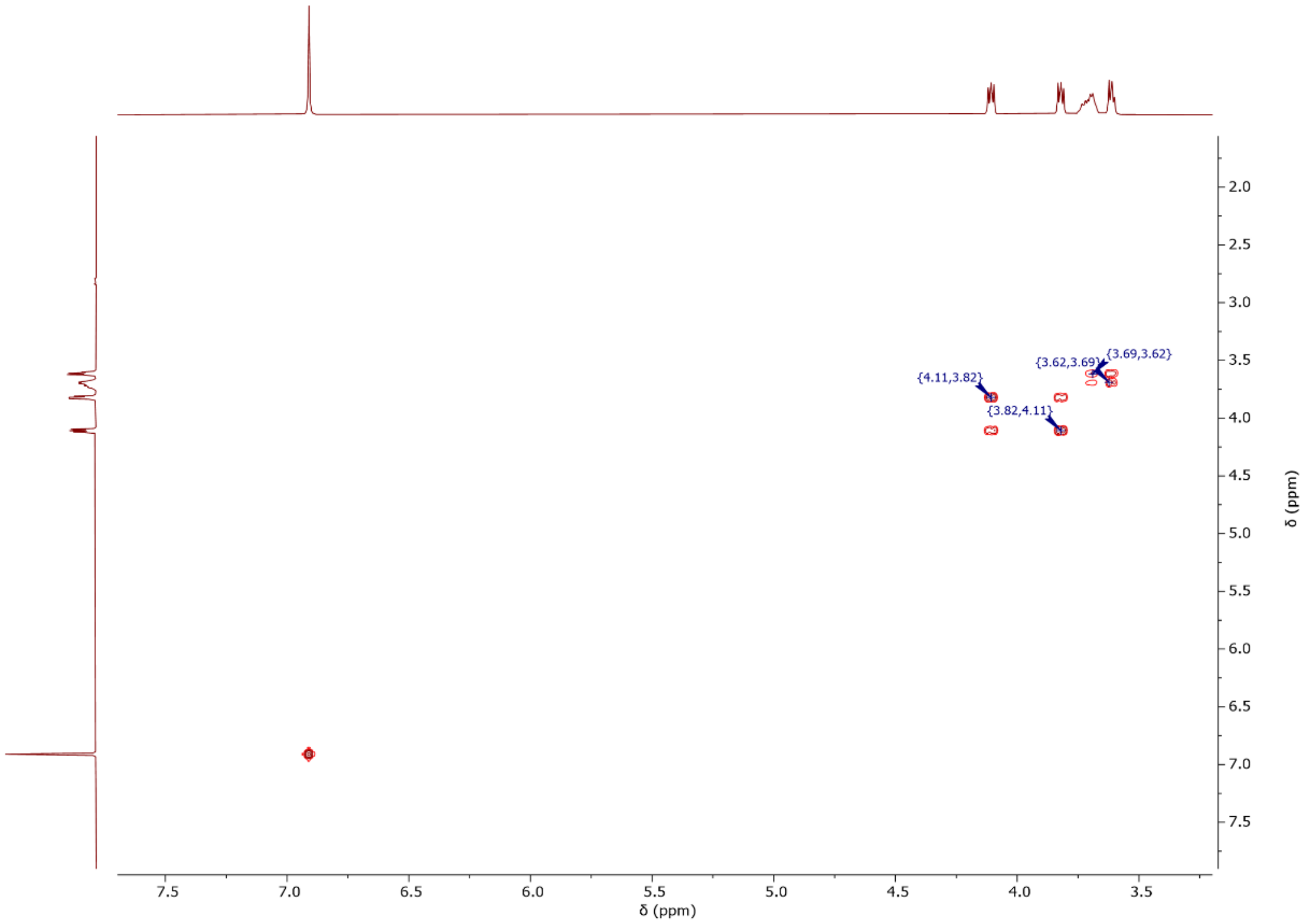

Figure S8: ^{1}H-^{1}H-COSY-NMR spectrum (400 MHz, DCM-d_2) of **5**.

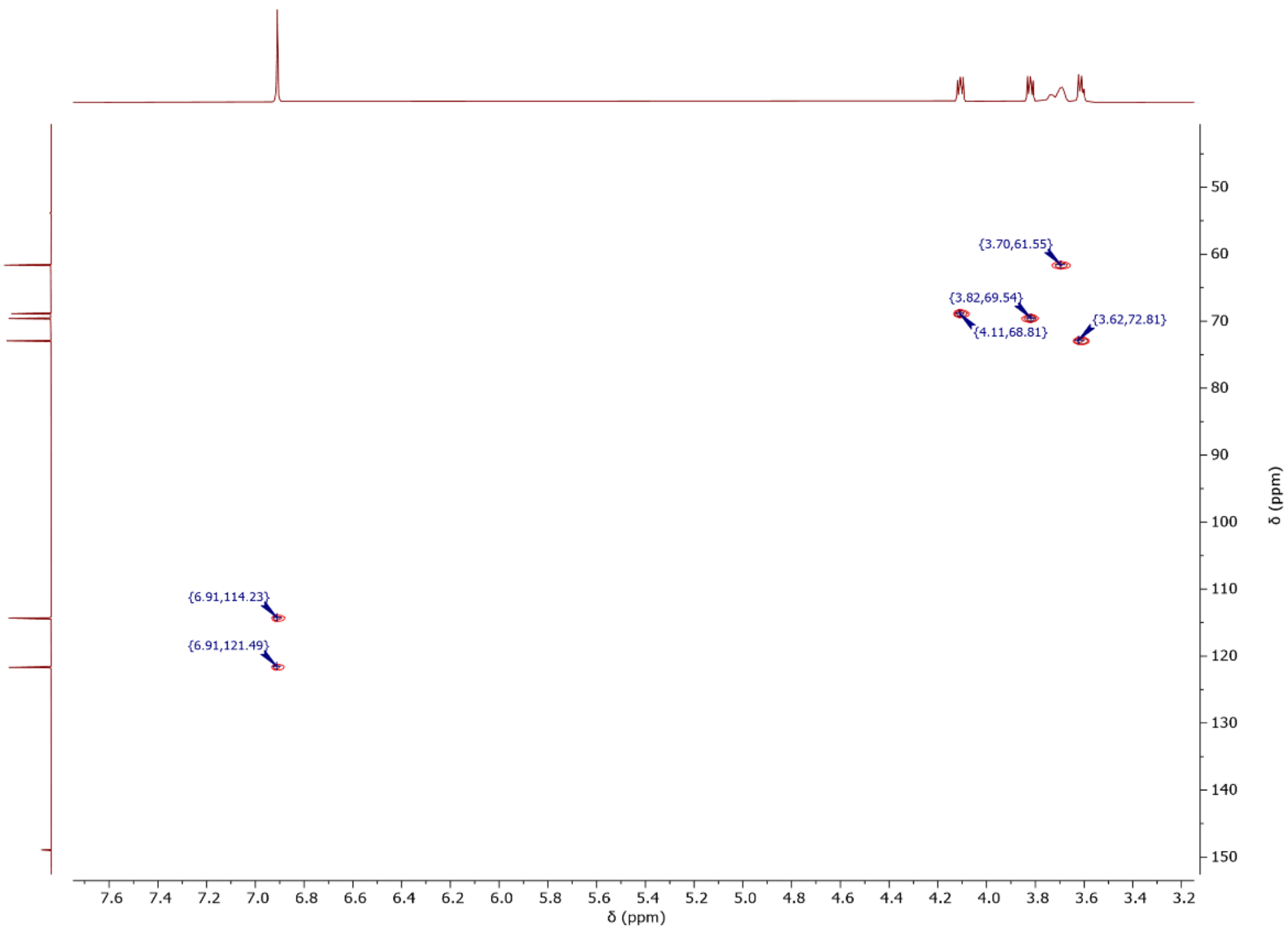

Figure S9: ^{1}H/^{13}C-gHSQC-NMR spectrum (400 MHz/101 MHz, DCM-d_2) of **5**.

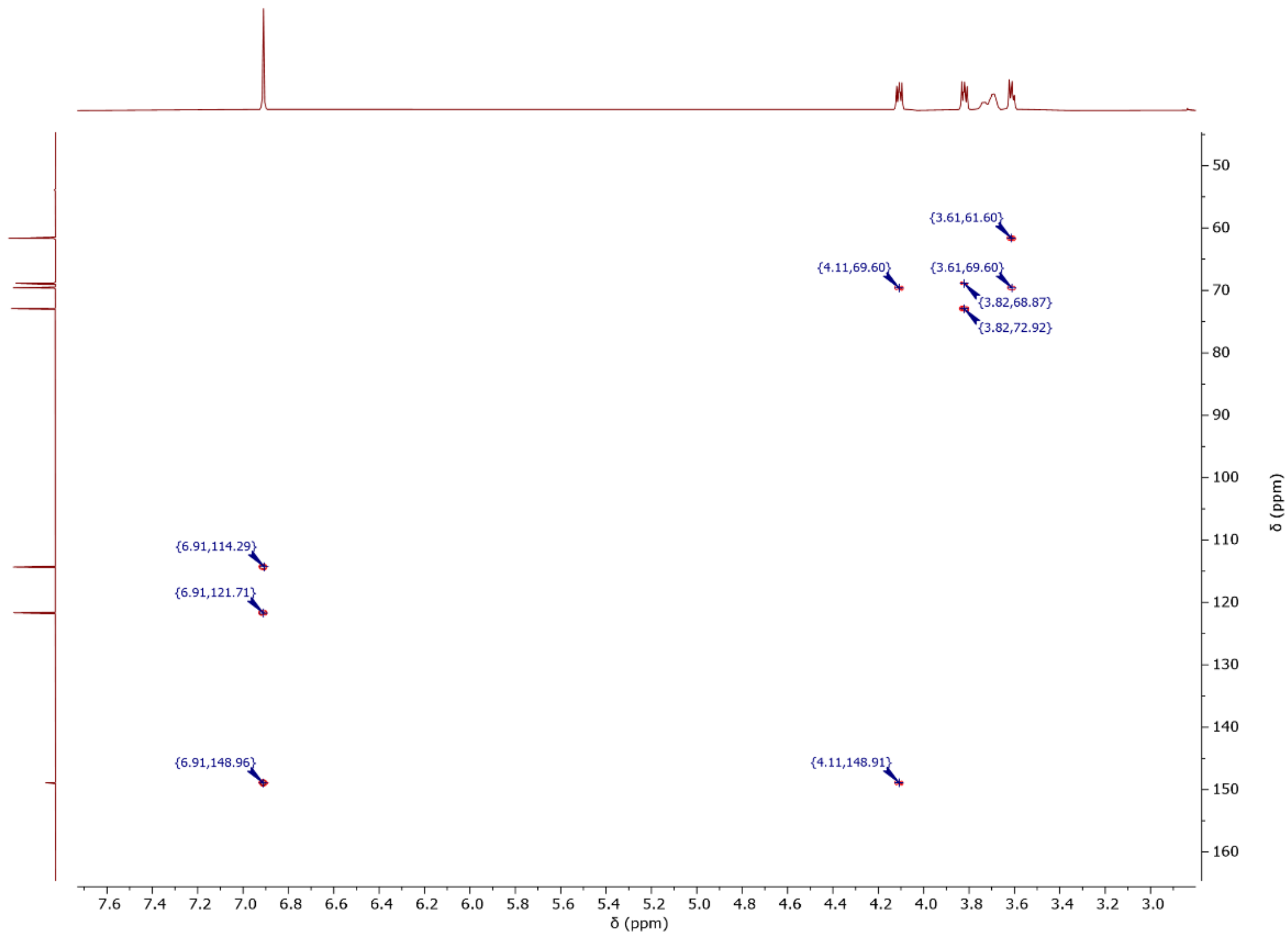

Figure S10: $^{1}H/^{13}C$-gHMBC-NMR spectrum (400 MHz/101 MHz, DCM-d_2) of 1**5**.

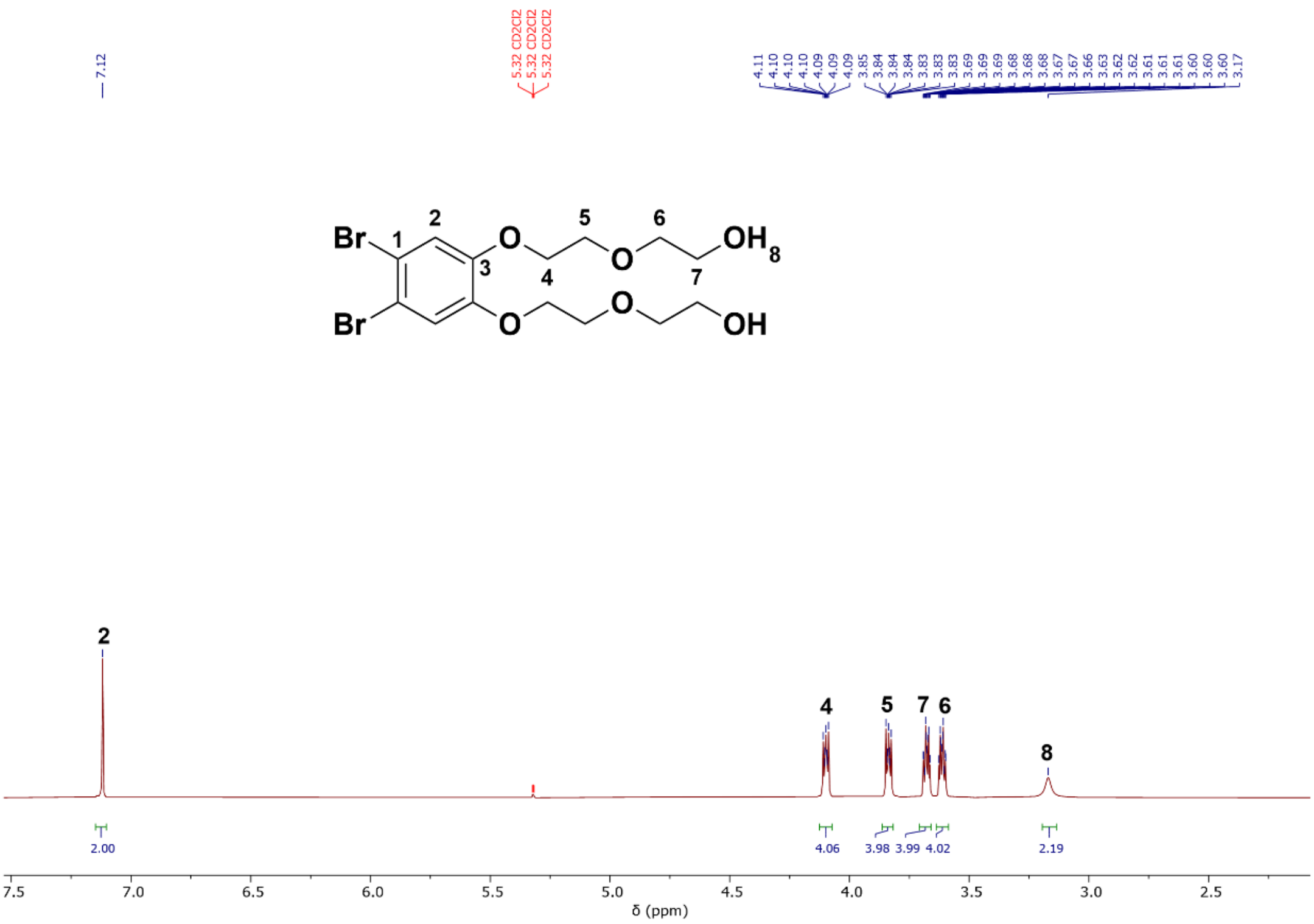

Figure S11: ^{1}H-NMR spectrum (400 MHz, DCM-d_2) of **6**.

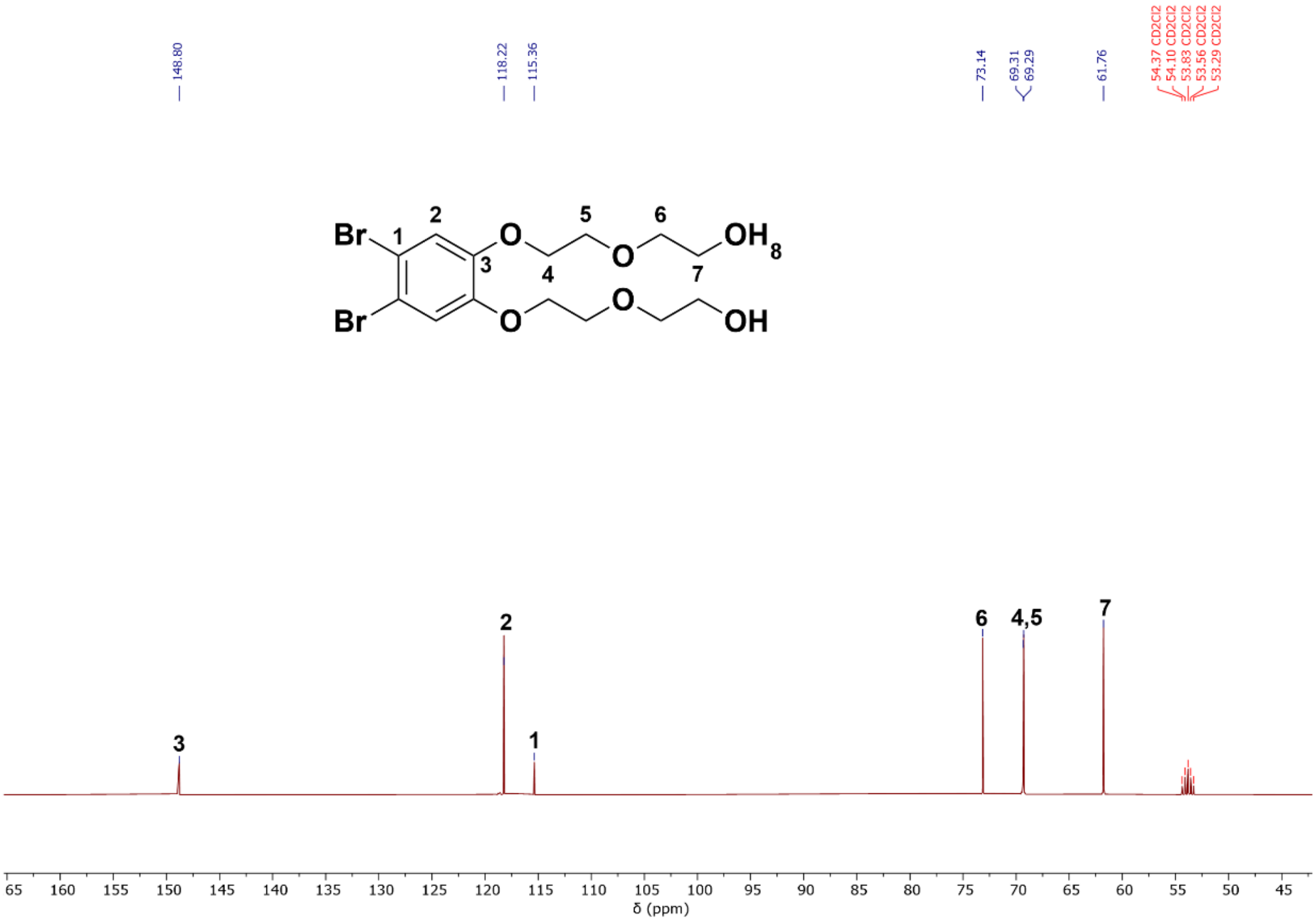

Figure S12: ^{13}C-{^{1}H}-NMR spectrum (101 MHz, DCM-d_2) of **6**.

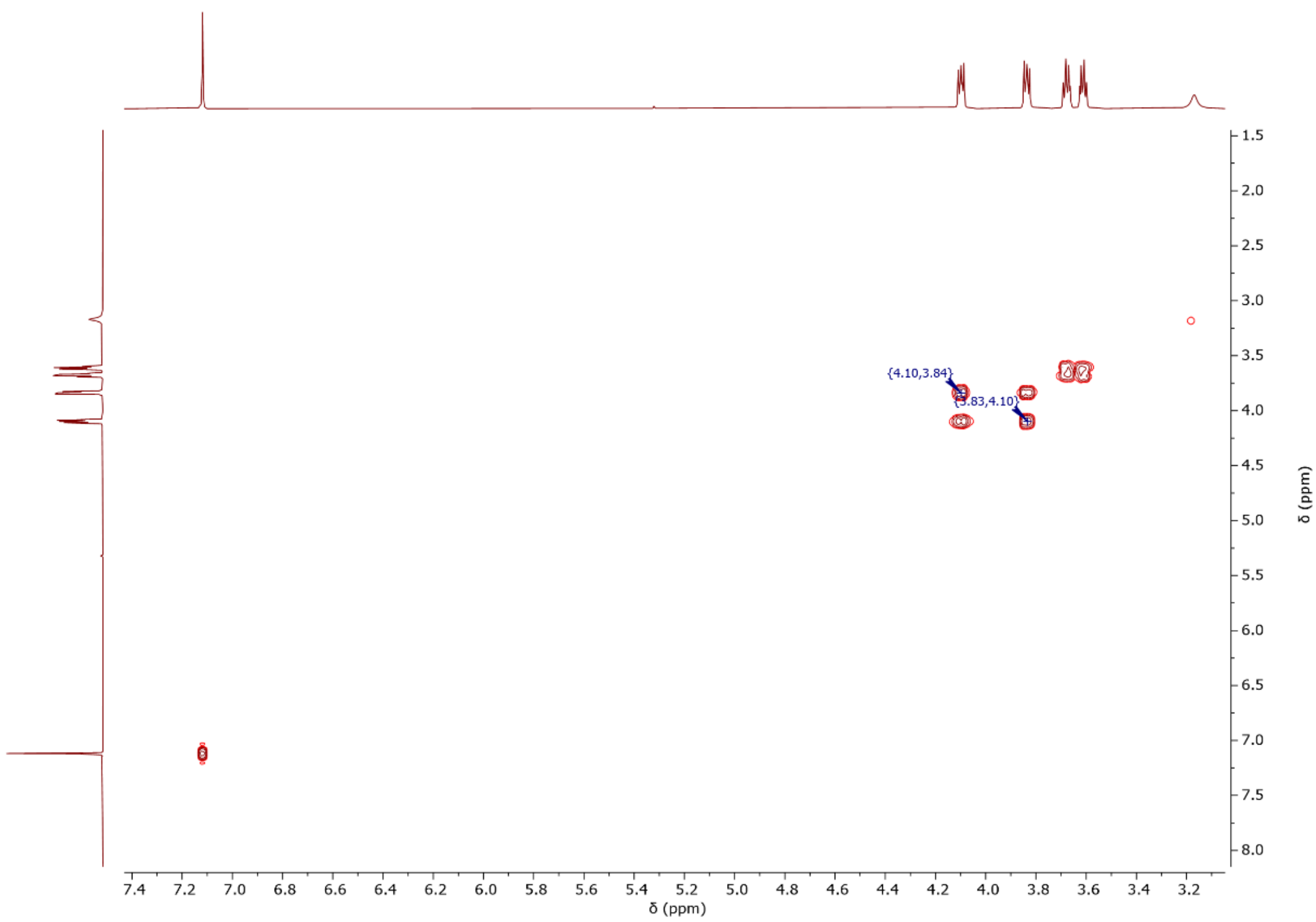

Figure S13: ^{1}H-^{1}H-COSY-NMR spectrum (400 MHz, DCM-d_2) of **6**.

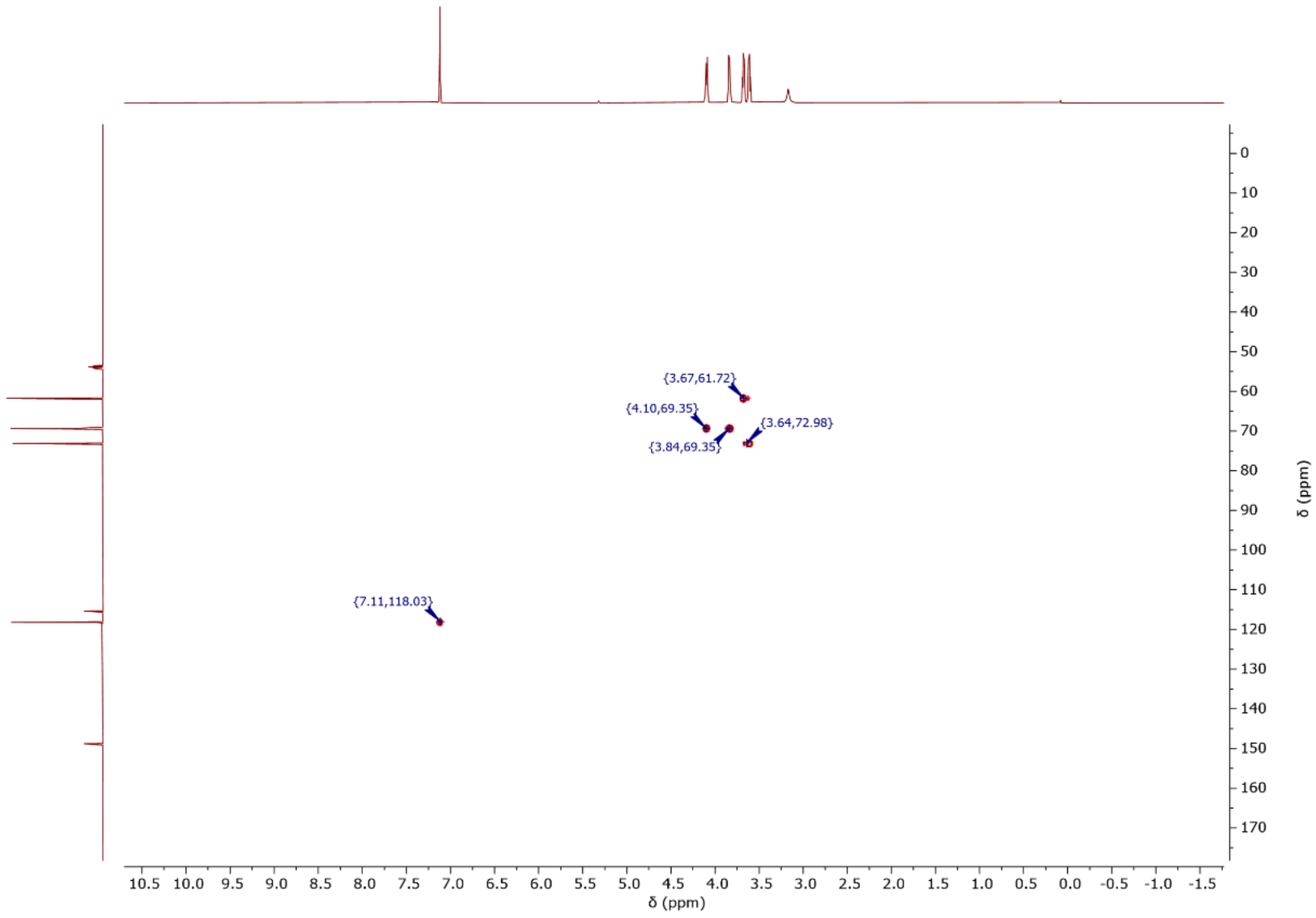

Figure S14: $^1H/^{13}C$-gHSQC-NMR spectrum (400 MHz/101 MHz, DCM-d_2) of **6**.

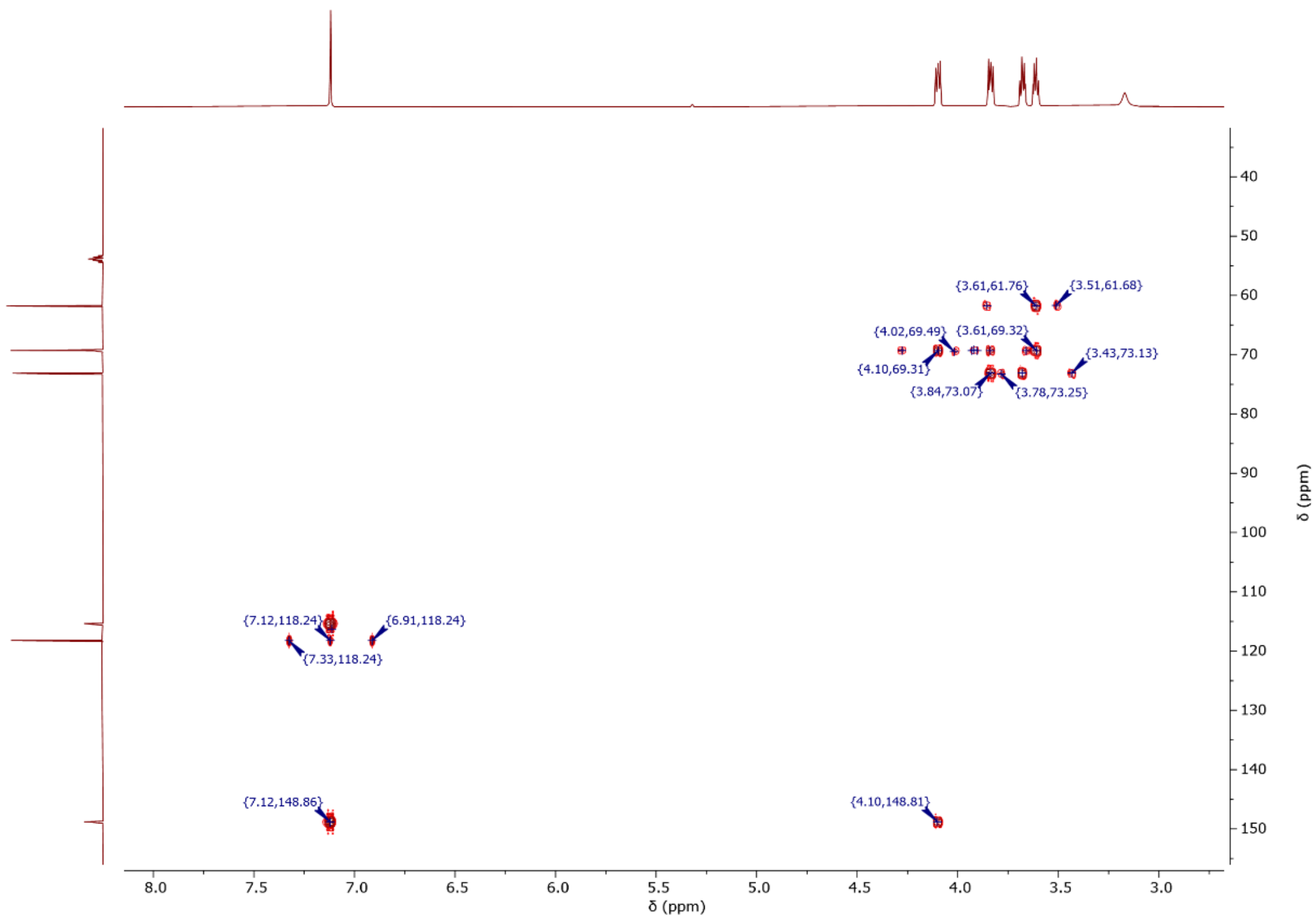

Figure S15: $^1H/^{13}C$-gHMBC-NMR spectrum (400 MHz/101 MHz, DCM-d_2) of **6**.

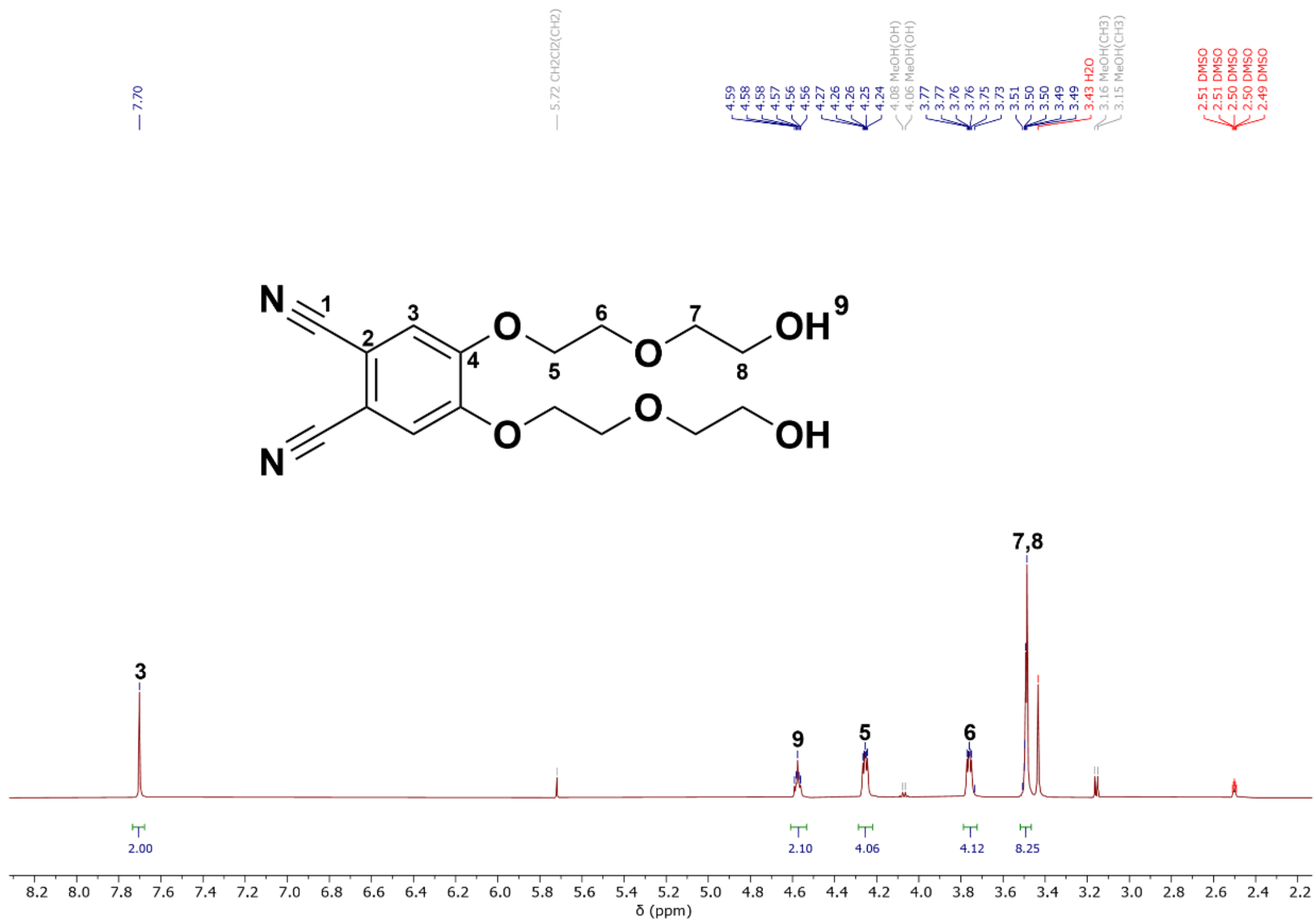

Figure S16: ^{1}H-NMR spectrum (400 MHz, DMSO-d_6) of **7**.

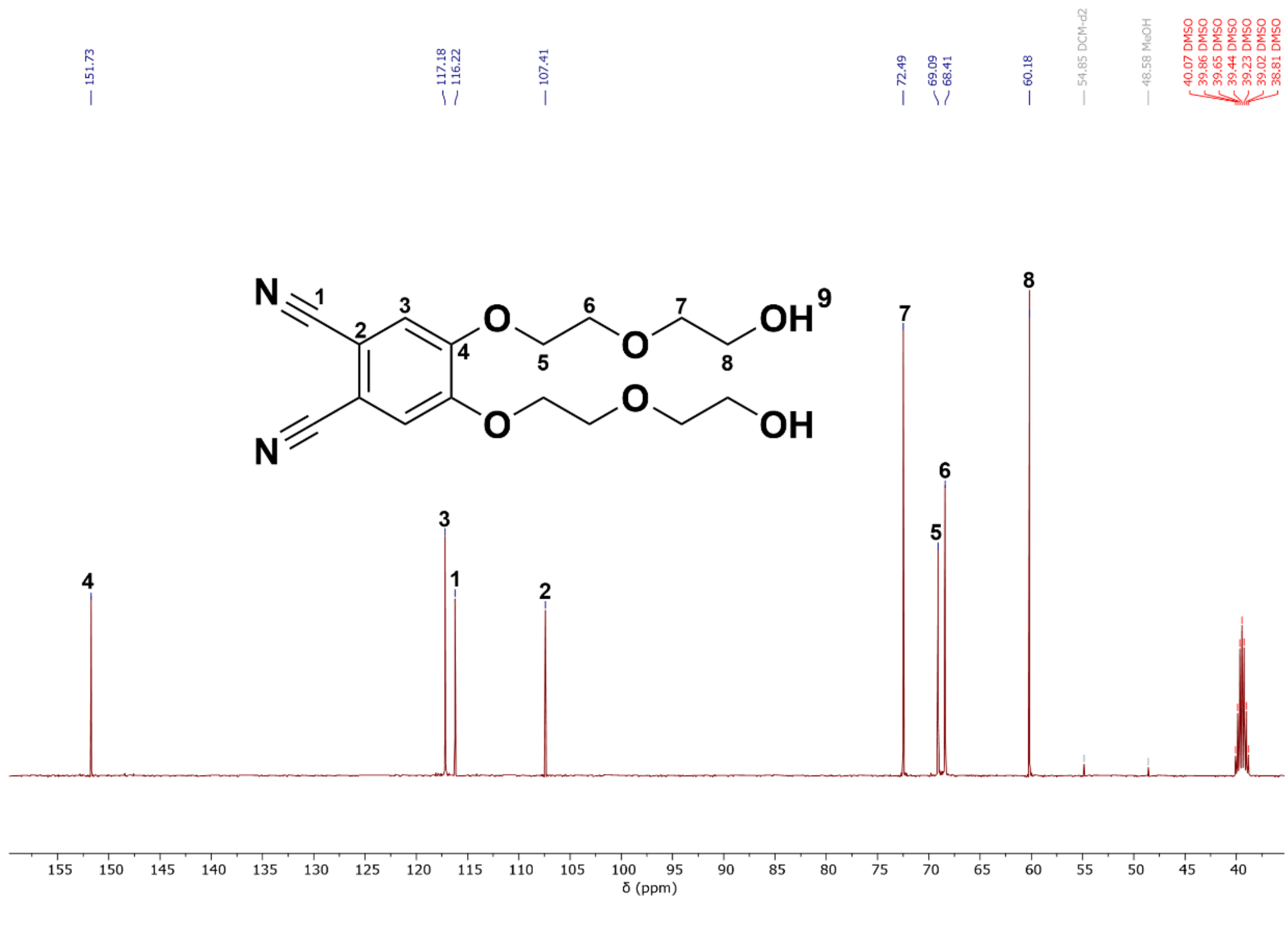

Figure S17: ^{13}C-{^{1}H}-NMR spectrum (101 MHz, DMSO-d_6) of **7**.

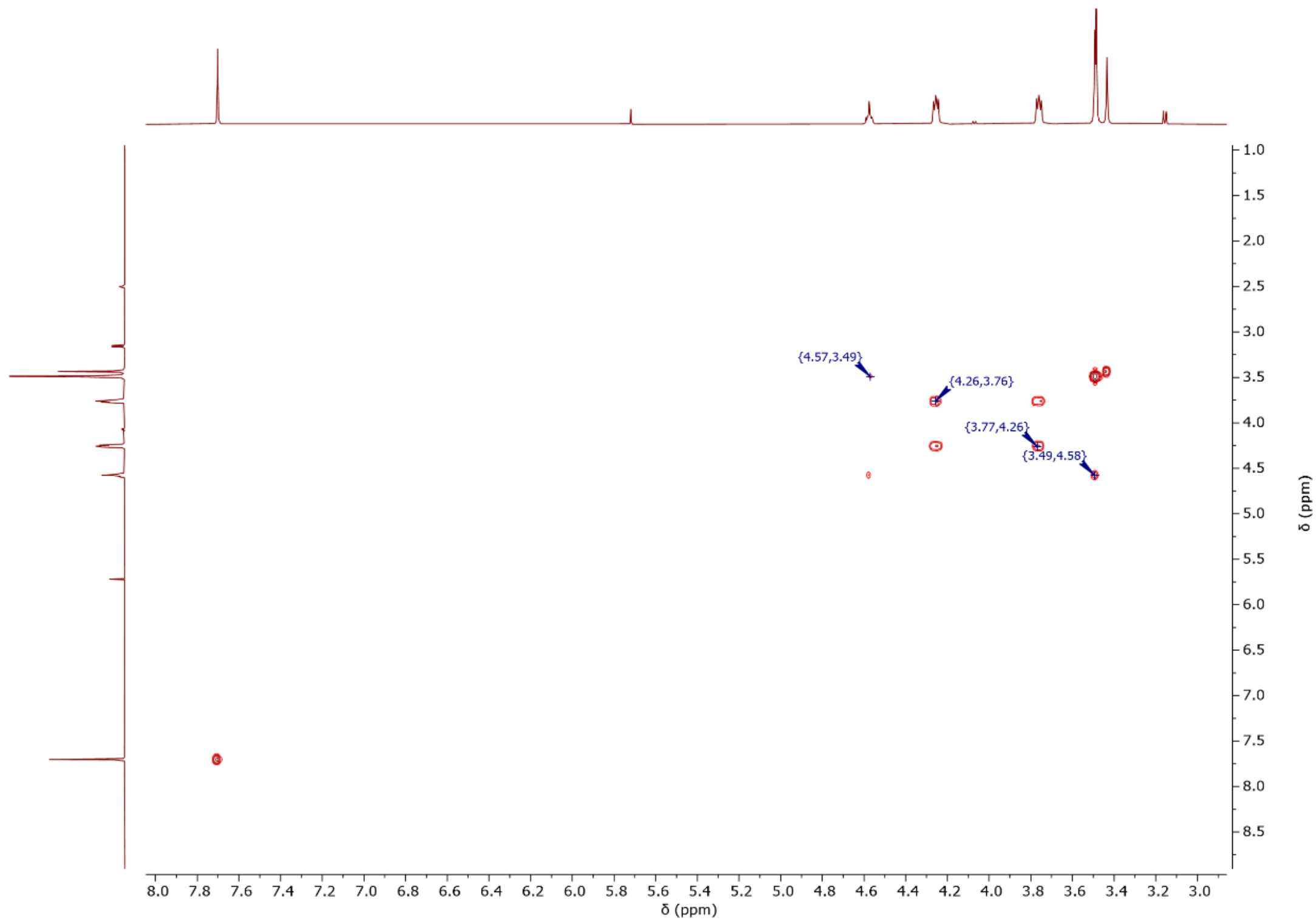

Figure S18: ^{1}H-^{1}H-COSY-NMR spectrum (400 MHz, DMSO-d_6) of **7**.

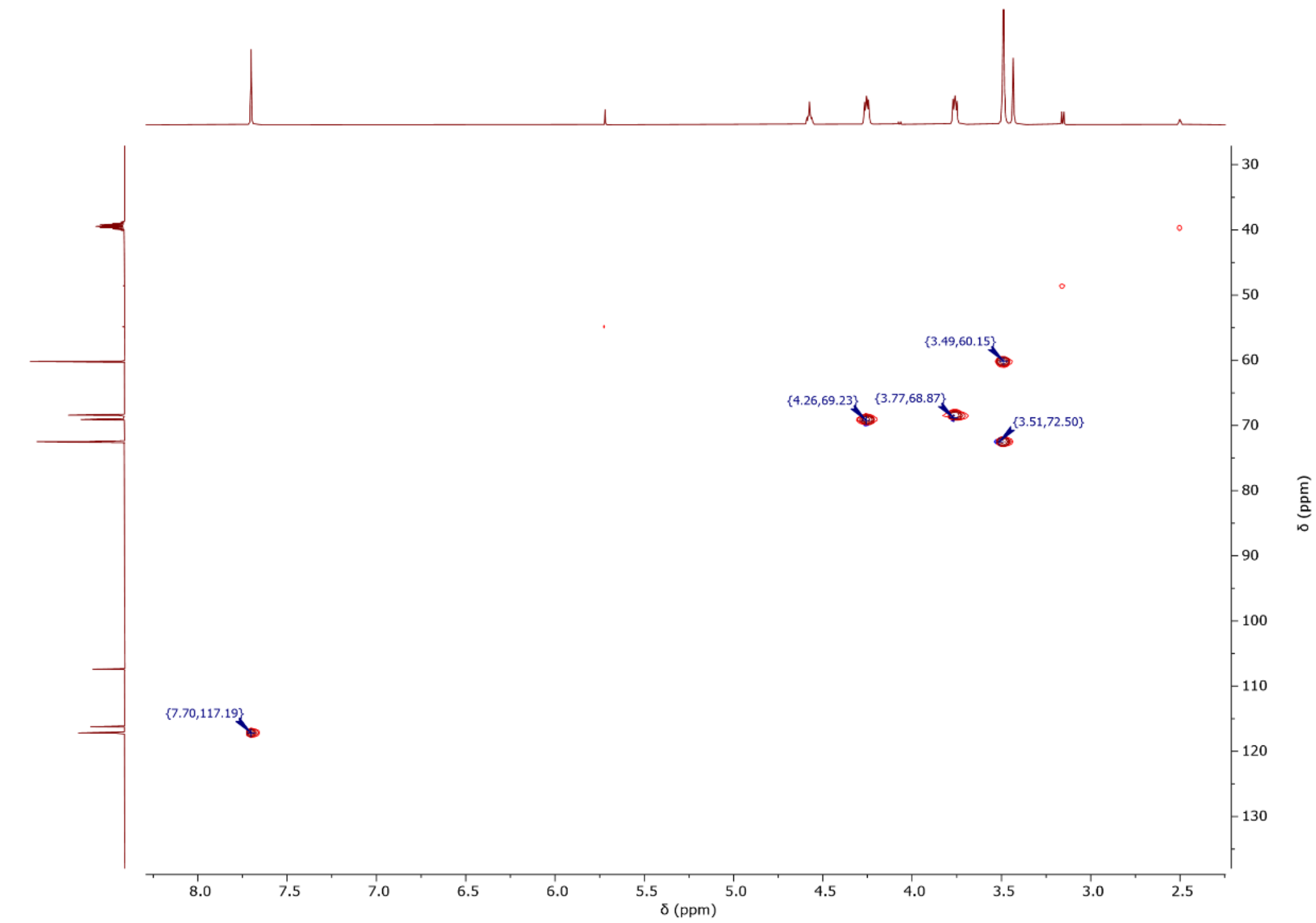

Figure S19: ^{1}H/^{13}C-gHSQC-NMR spectrum (400 MHz/101 MHz, DMSO-d_6) of **7**.

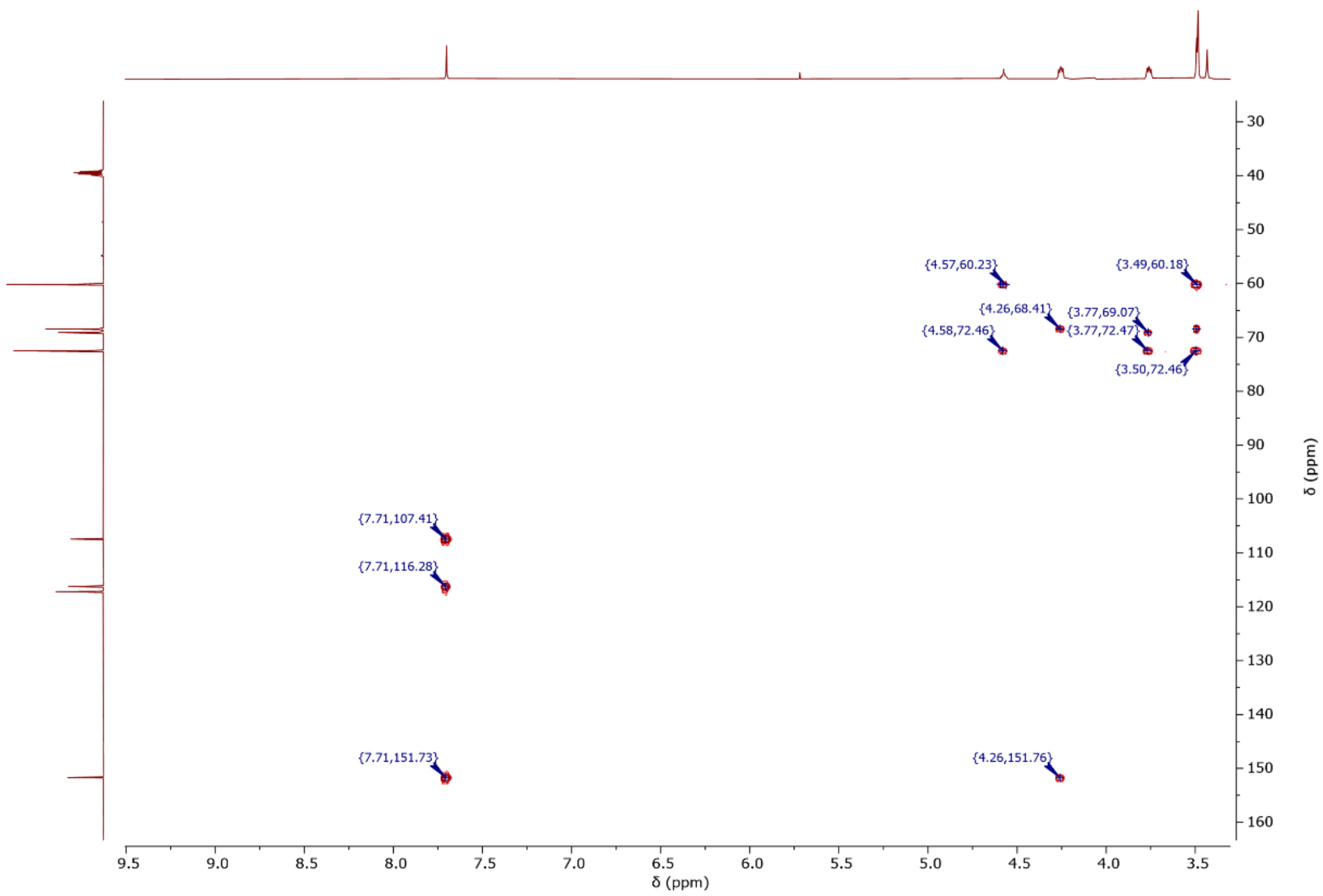

Figure S20: $^{1}H/^{13}C$-gHMBC-NMR spectrum (400 MHz/101 MHz, DMSO-d_6) of **7**.

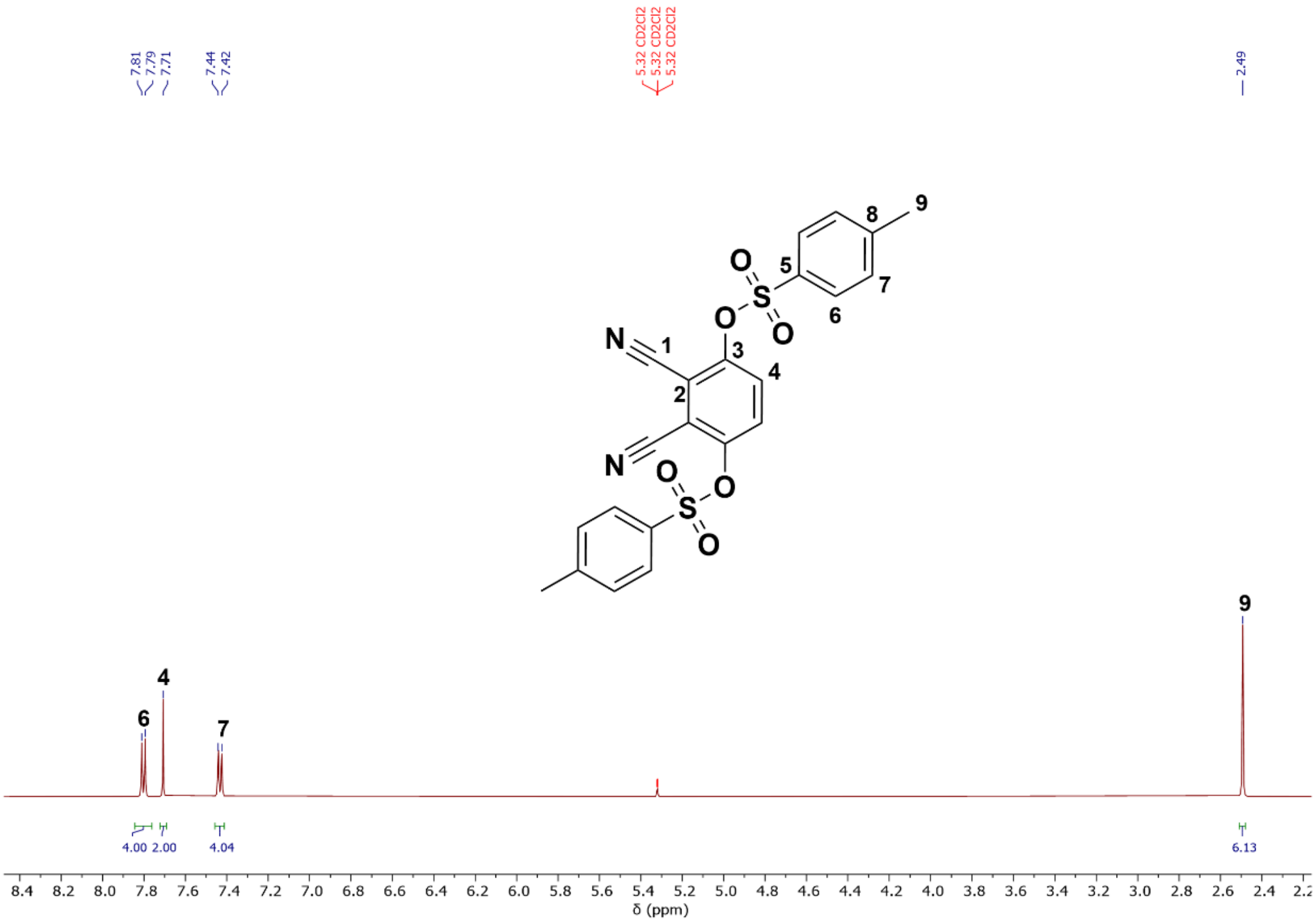

Figure S21: ^{1}H-NMR spectrum (500 MHz, DCM-d_2) of **8**.

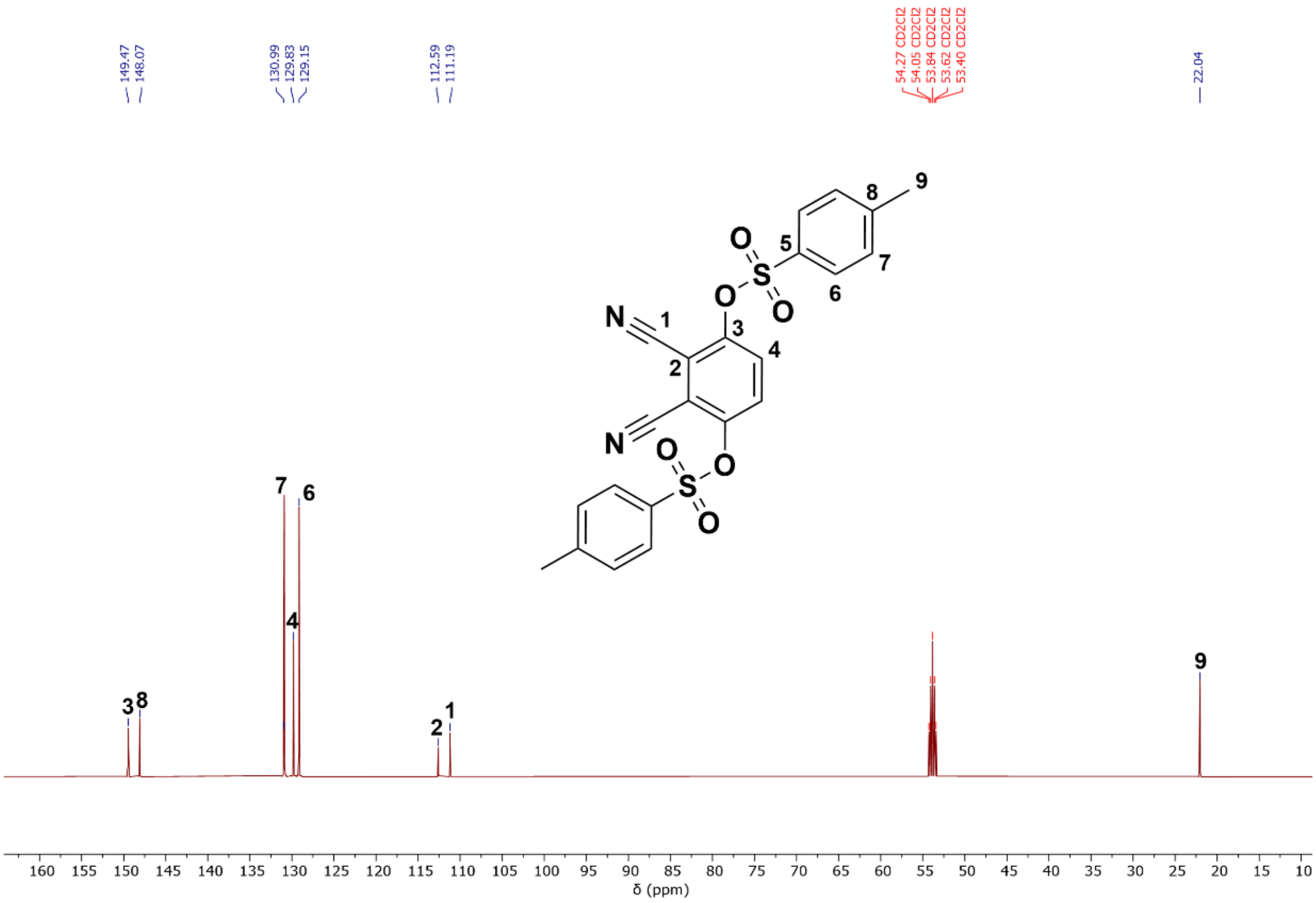

Figure S22: $^{13}C-\{^{1}H\}$-NMR spectrum (126 MHz, DCM-d_2) of **8**.

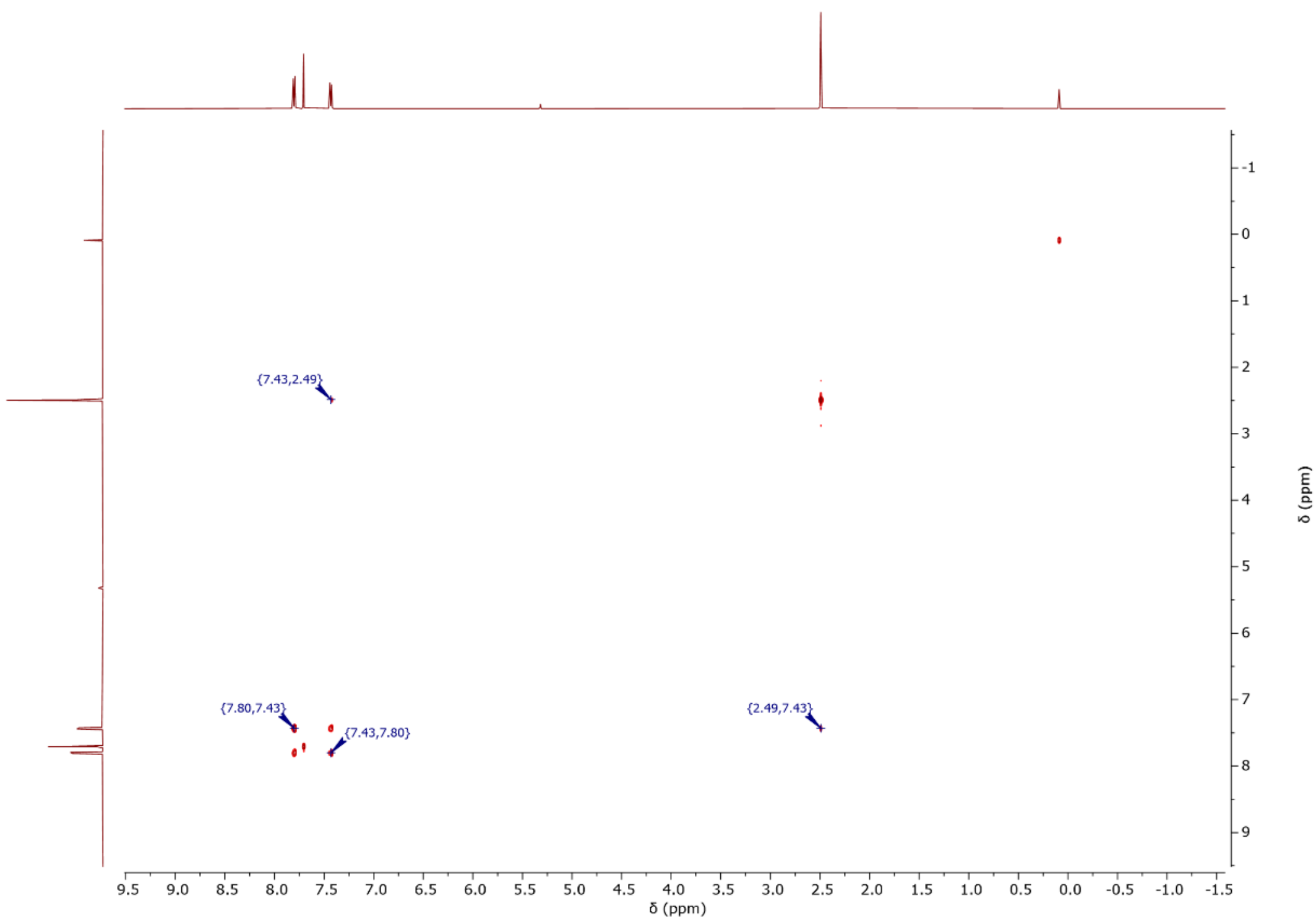

Figure S23: $^{1}H-^{1}H$-COSY-NMR spectrum (500 MHz, DCM-d_2) of **8**.

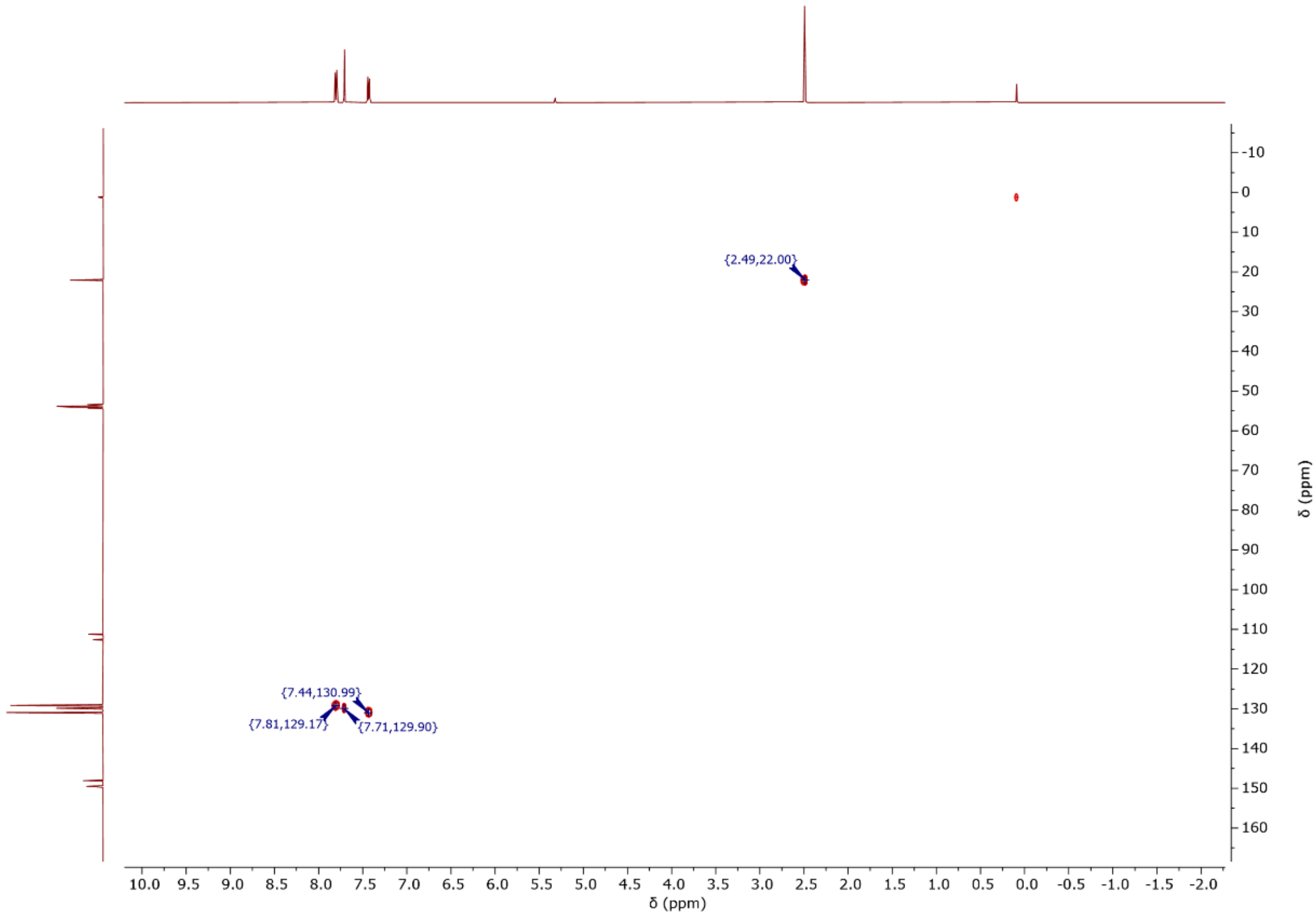

Figure S24: ^{1}H/^{13}C-gHSQC-NMR spectrum (500 MHz/126 MHz, DCM-d_2) of **8**.

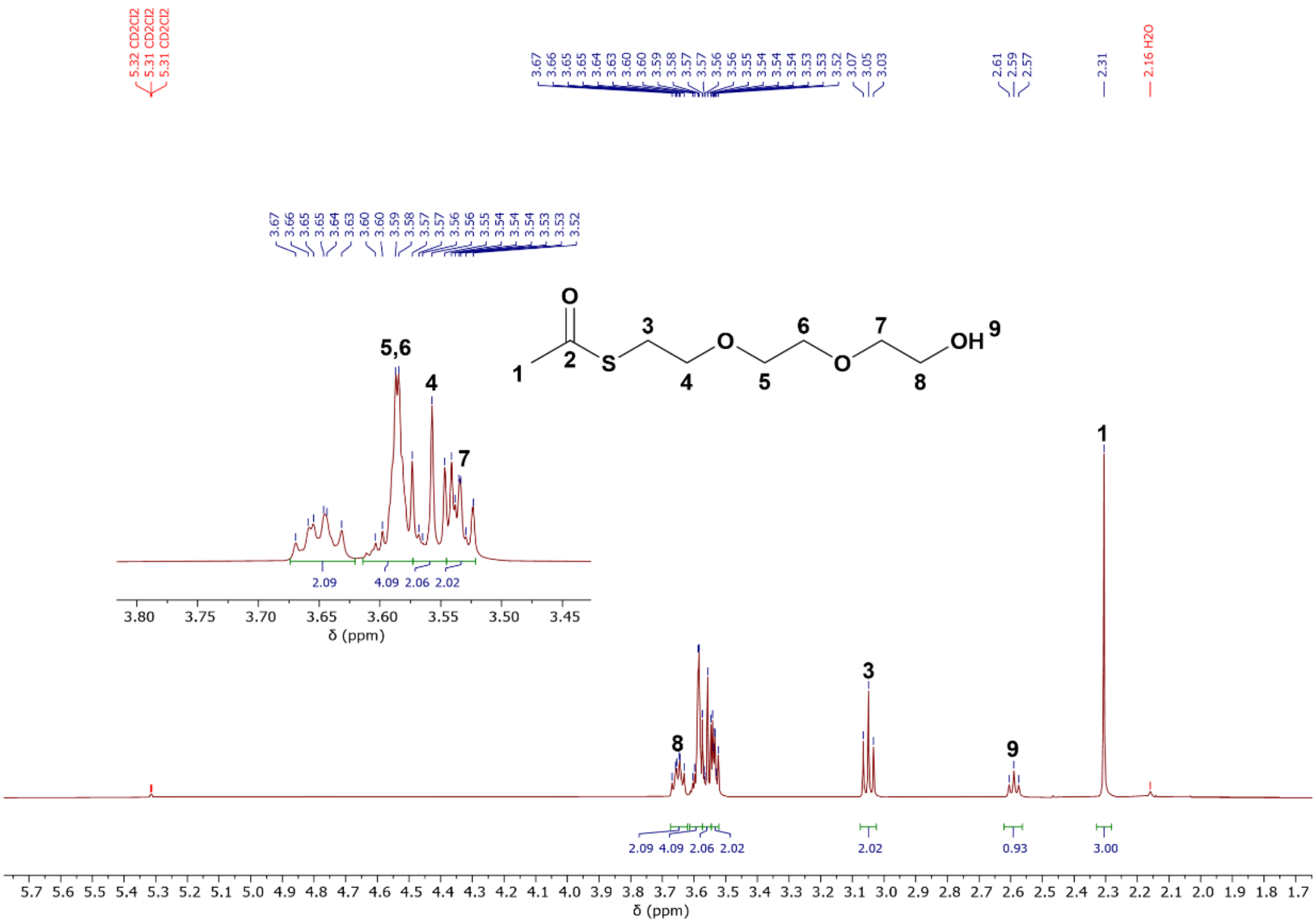

Figure S25: ^{1}H-NMR spectrum (400 MHz, DCM-d_2) of **10**.

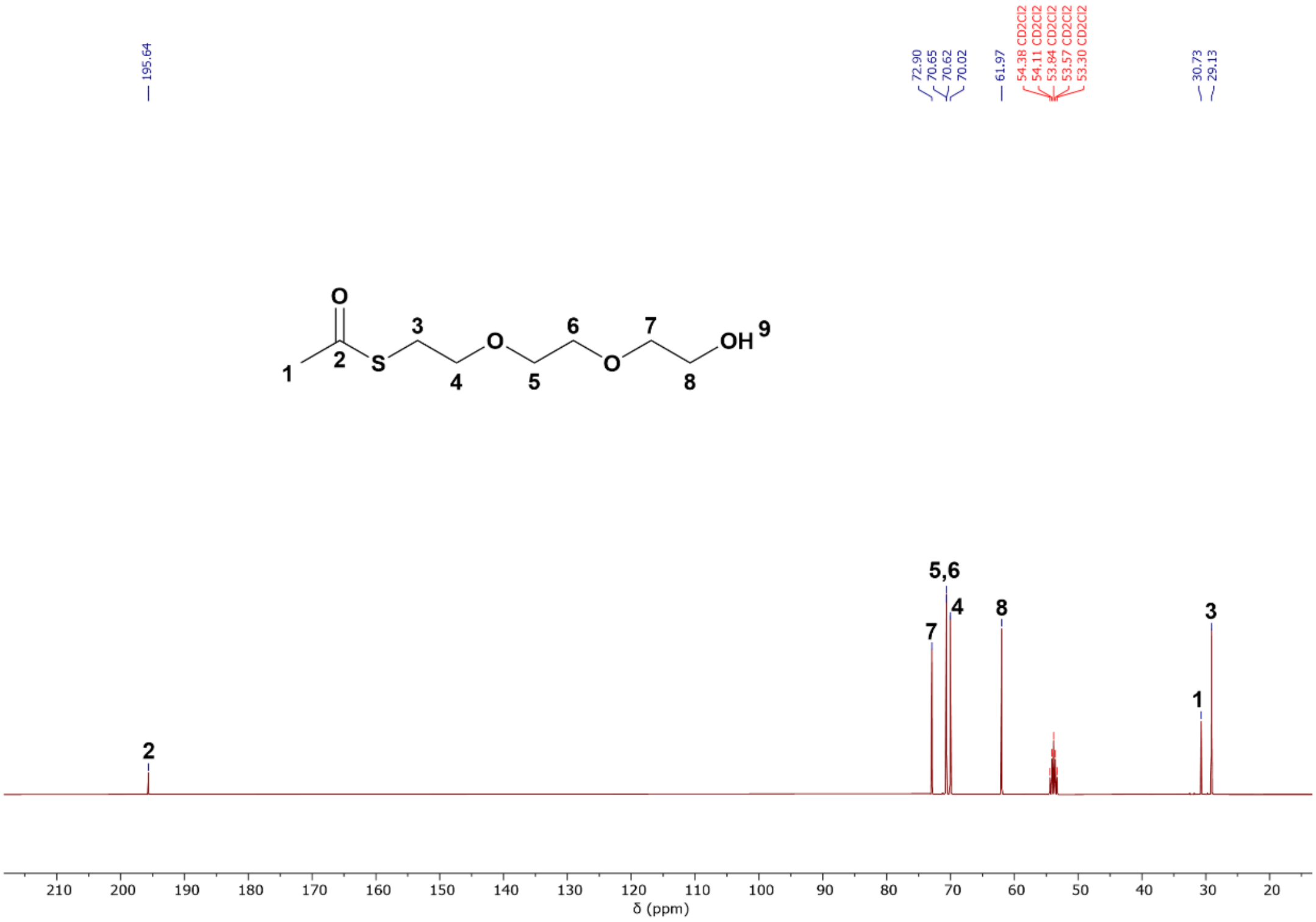

Figure S26: ^{13}C-{^{1}H}-NMR spectrum (101 MHz, DCM-d_2) of **10**.

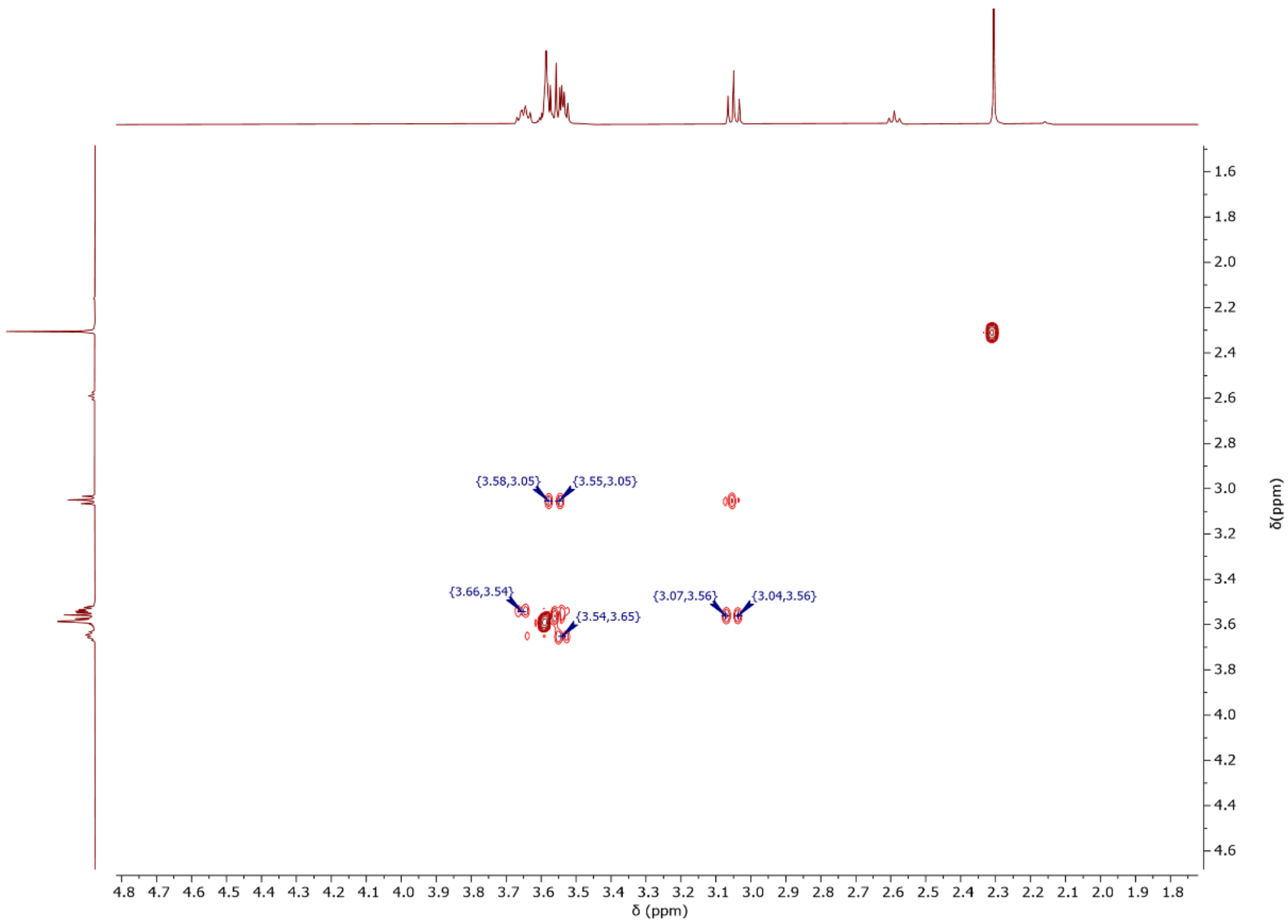

Figure S27: ^{1}H-^{1}H-COSY-NMR spectrum (400 MHz, DCM-d_2) of **10**.

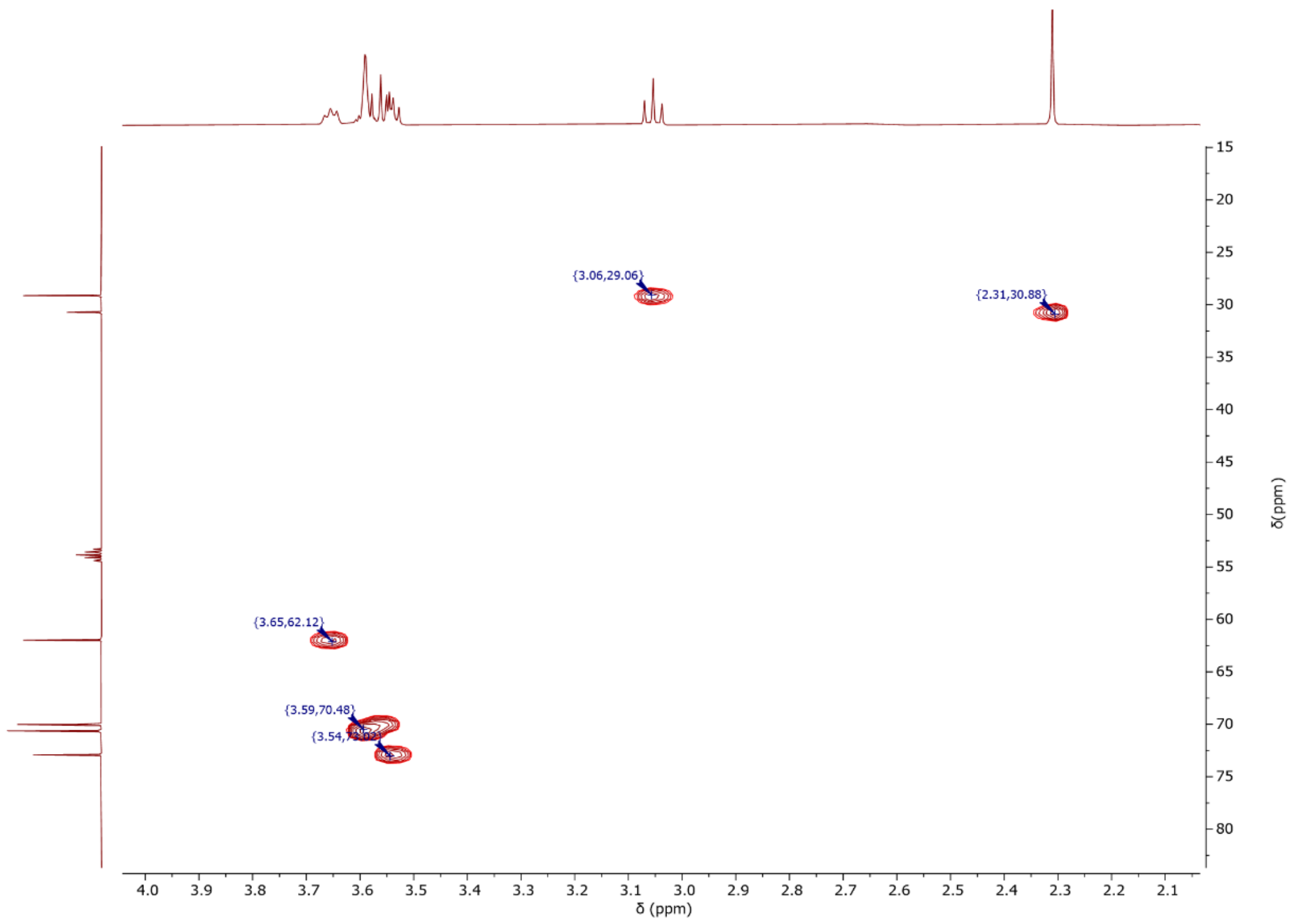

Figure S28: $^{1}H/^{13}C$-gHSQC-NMR spectrum (400 MHz/101 MHz, DCM-d_2) of **10**.

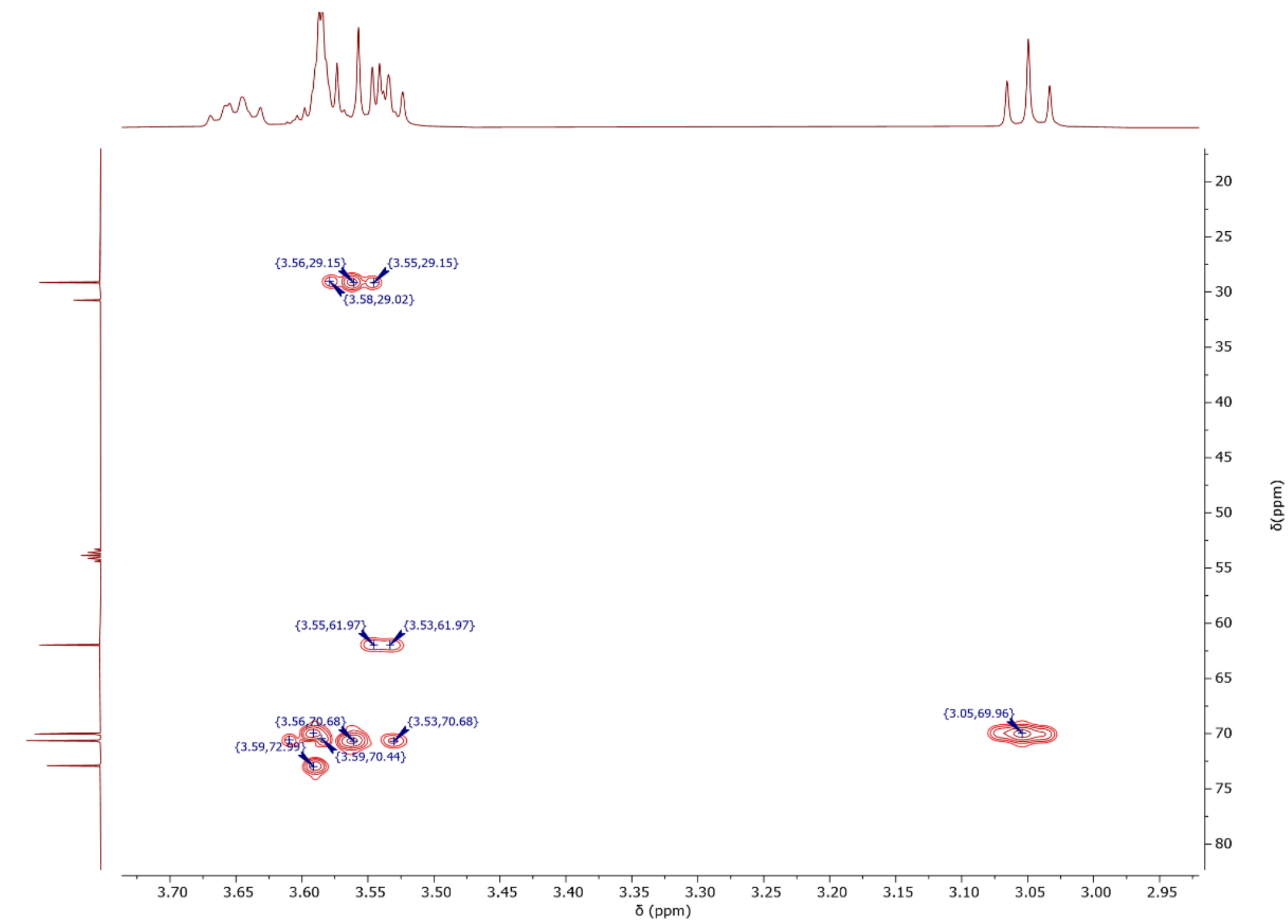

Figure S29: $^{1}H/^{13}C$-gHMBC-NMR spectrum (400 MHz/101 MHz, DCM-d_2) of **10**.

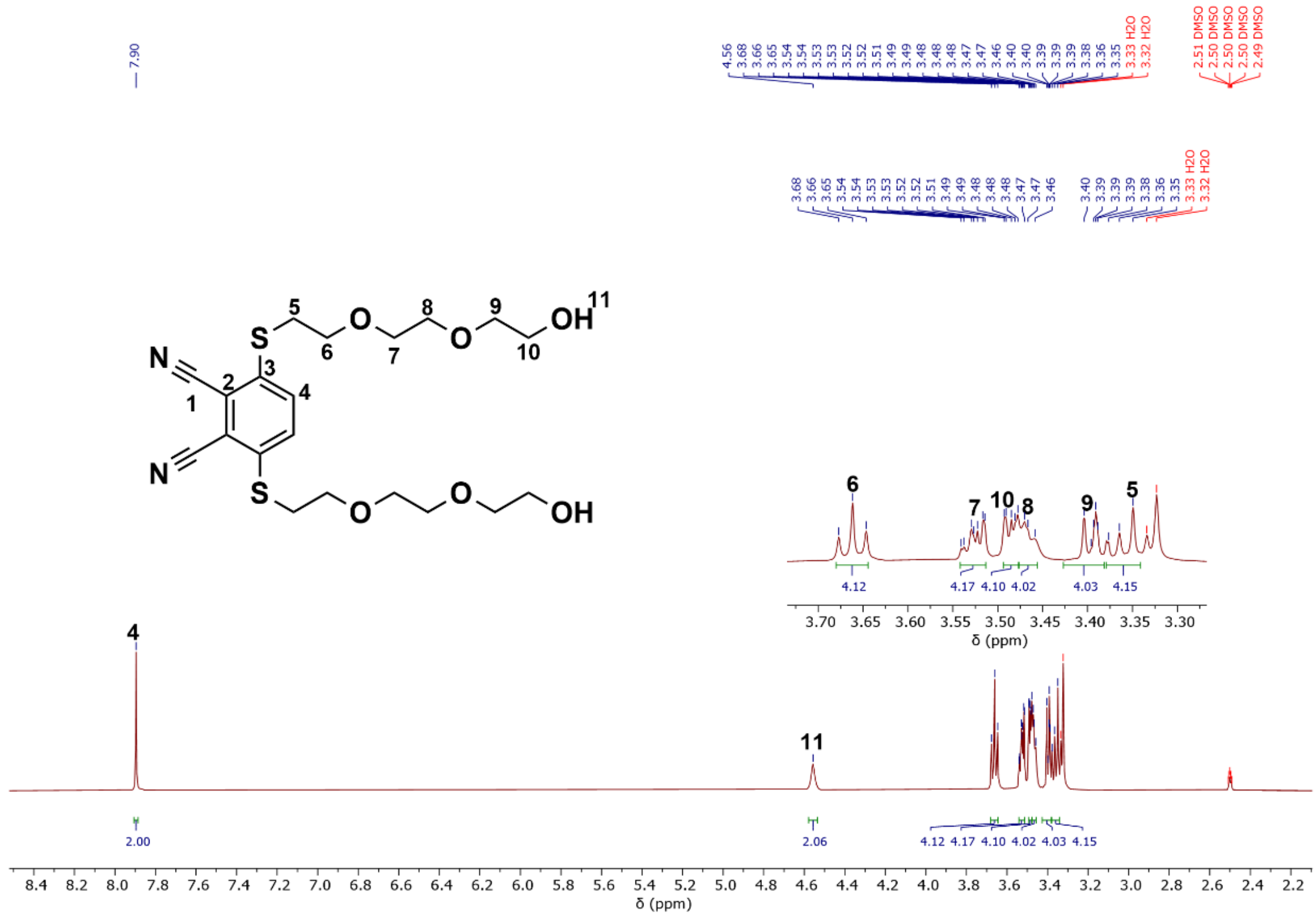

Figure S30: ^{1}H-NMR spectrum (400 MHz, DMSO-d_6) of **12**.

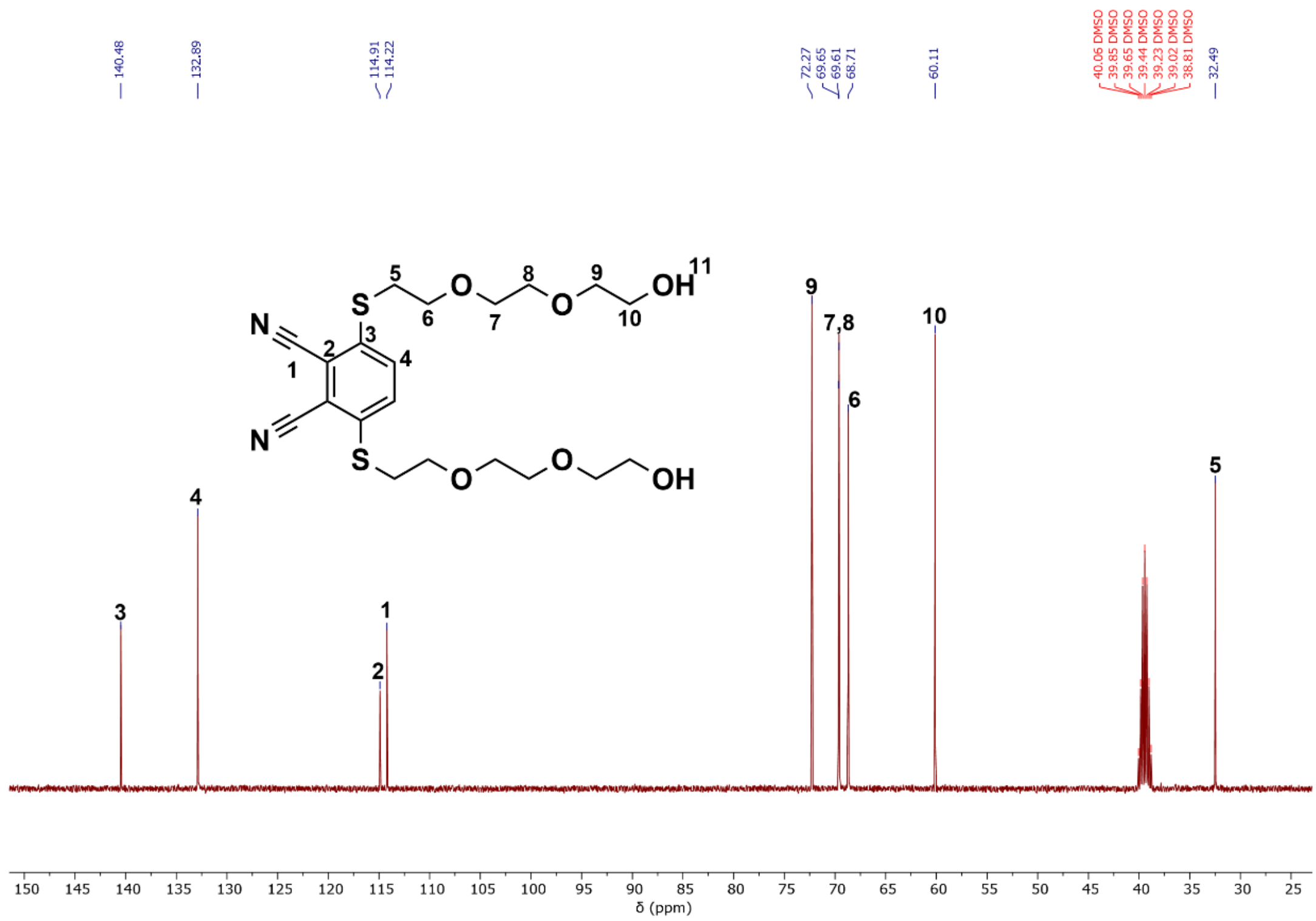

Figure S31: ^{13}C-{^{1}H}-NMR spectrum (101 MHz, DMSO-d_6) of **12**.

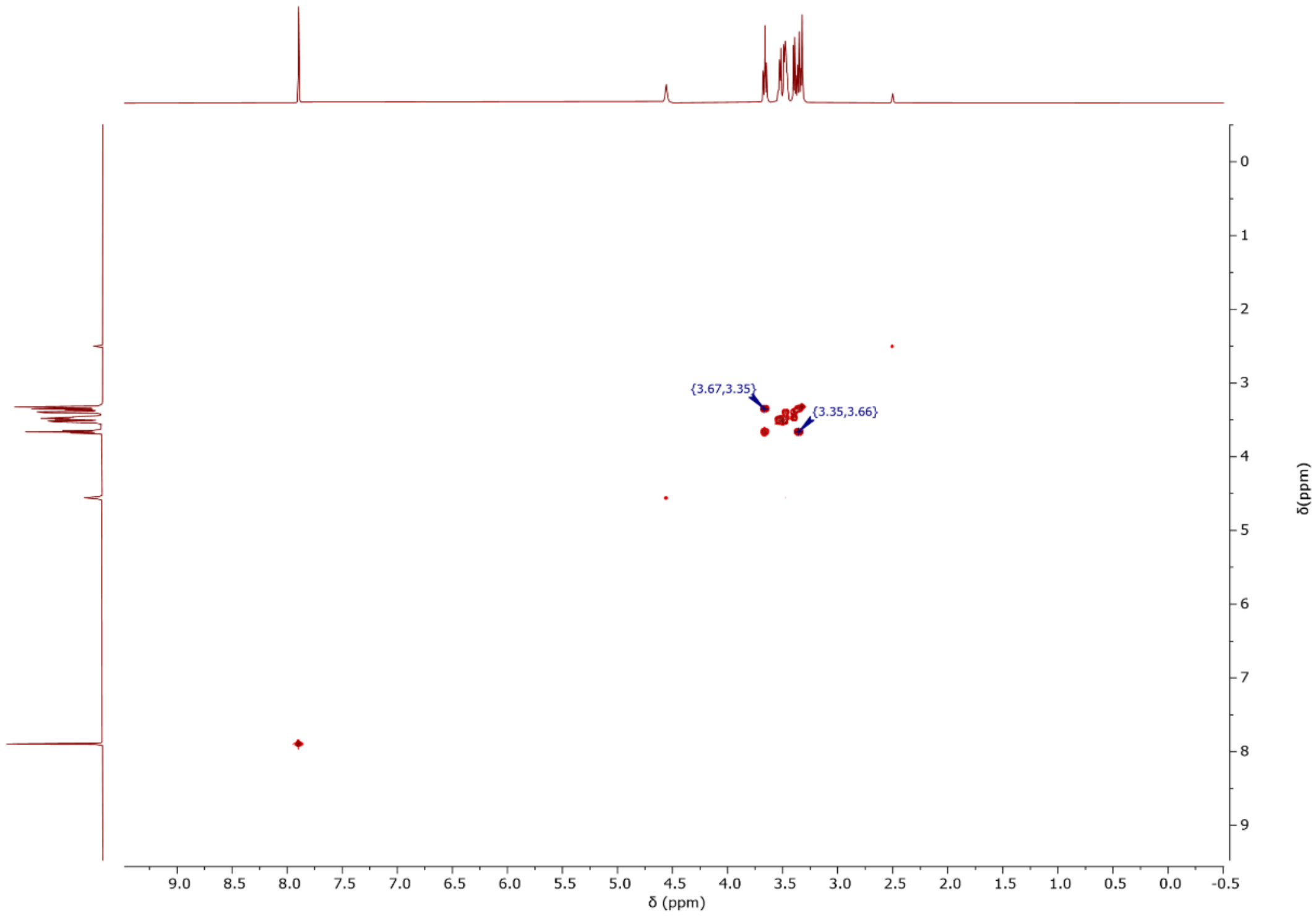

Figure S32: ^{1}H-^{1}H-COSY-NMR spectrum (400 MHz, DMSO-d_6) of **12**.

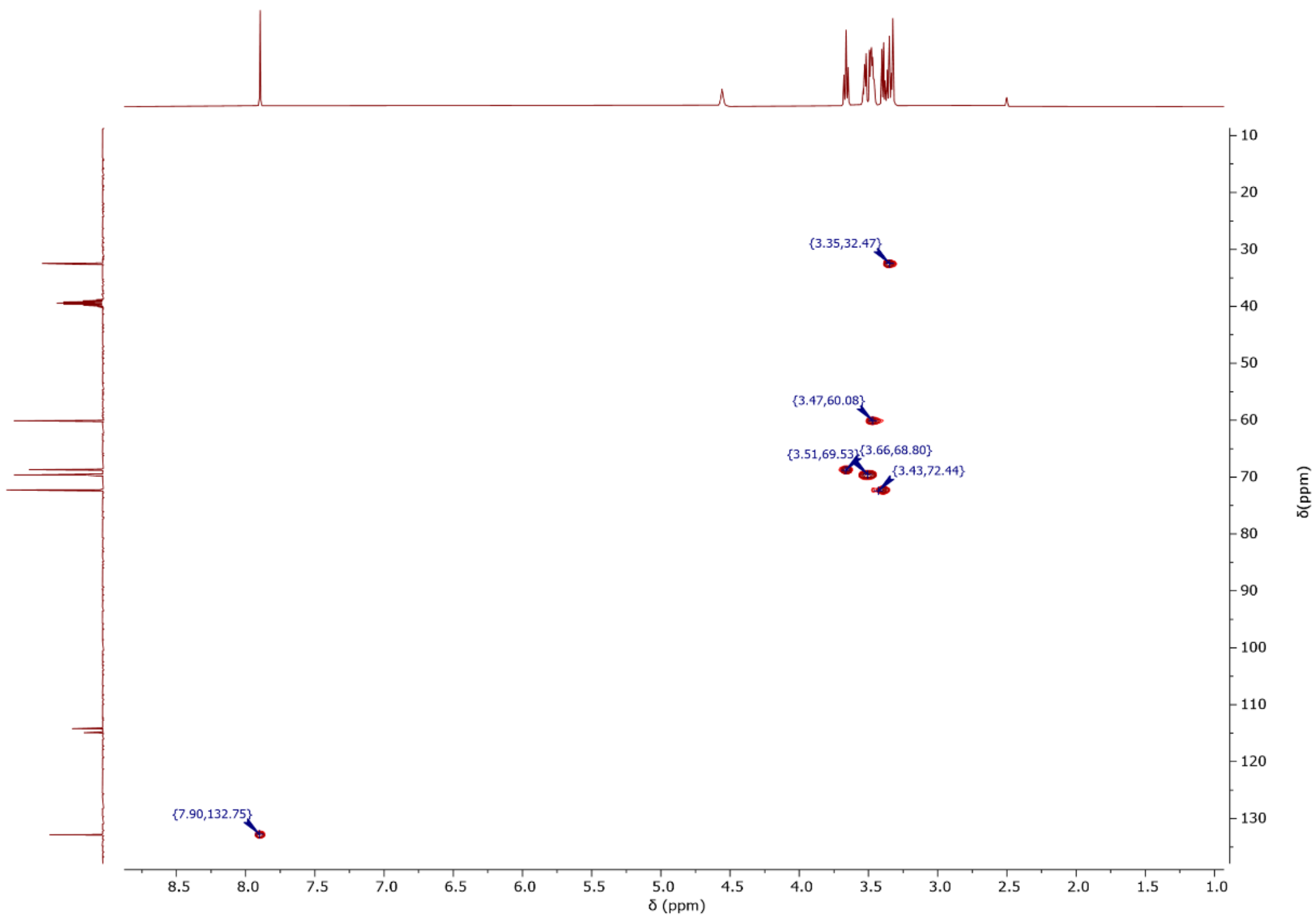

Figure S33: $^{1}H/^{13}C$-gHSQC-NMR spectrum (400 MHz/101 MHz, DMSO-d_6) of **12**.

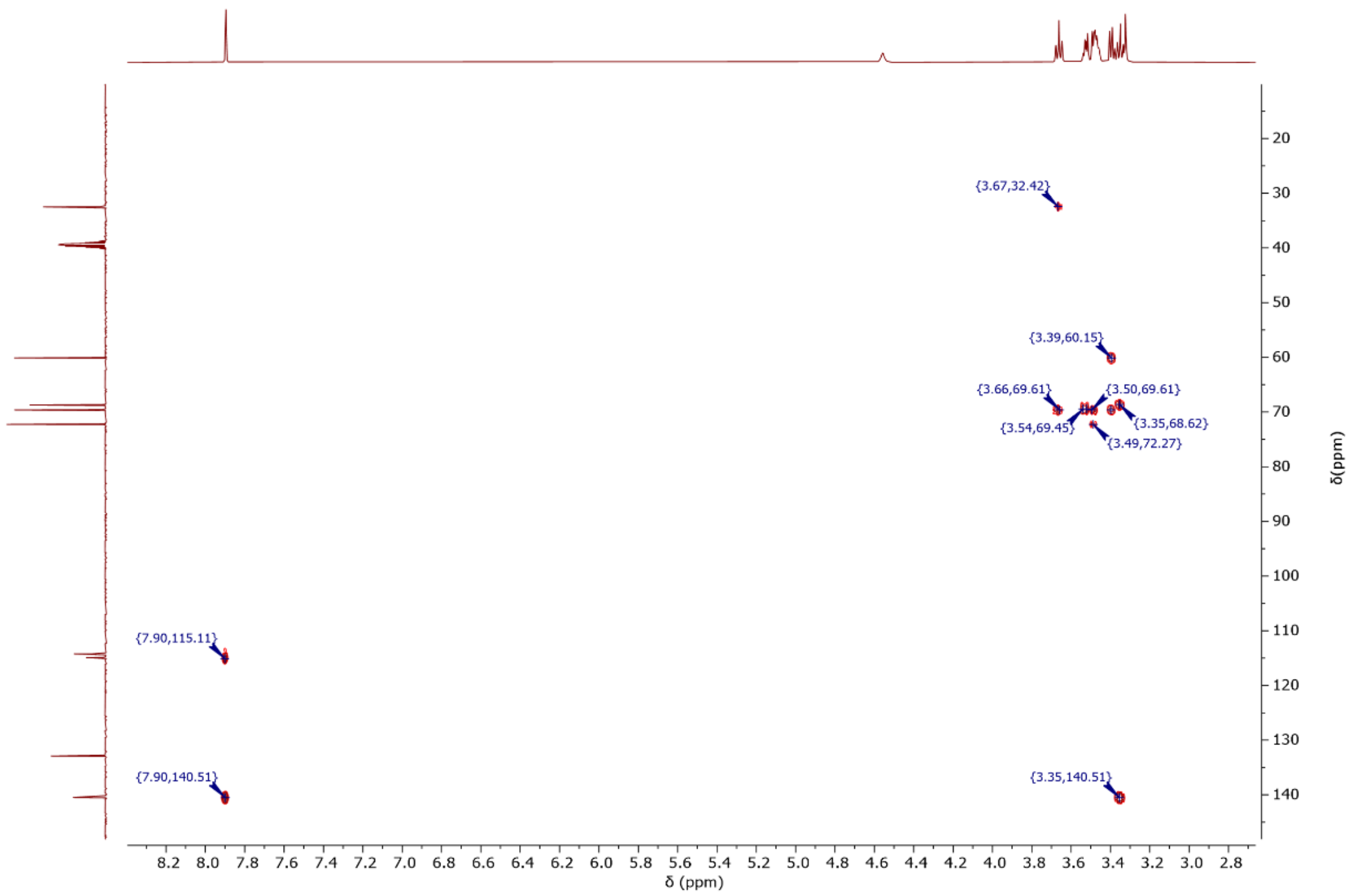

Figure S34: $^{1}H/^{13}C$-gHMBC-NMR spectrum (400 MHz/101 MHz, DMSO-d_6) of **12**.

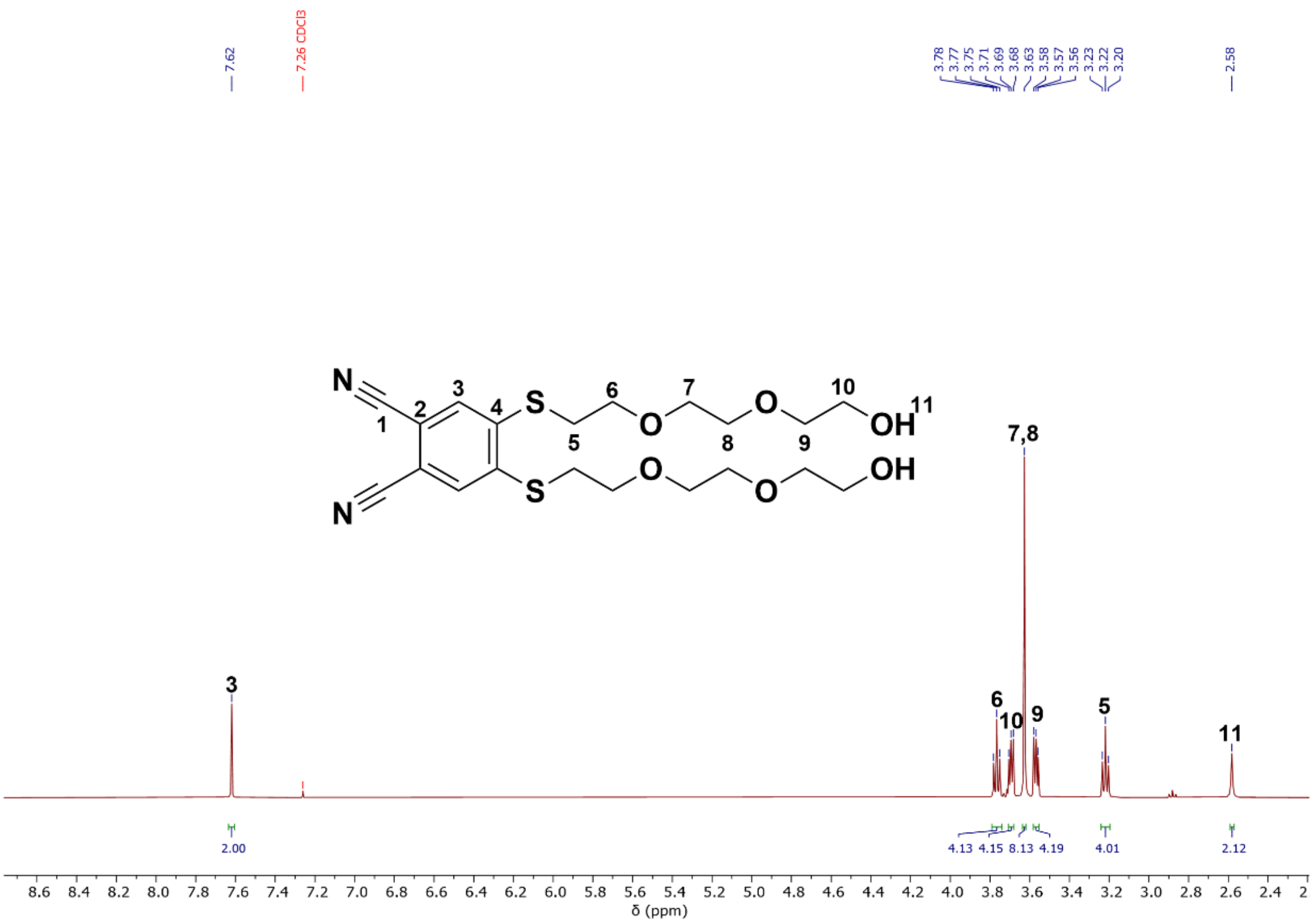

Figure S35: ^{1}H-NMR spectrum (400 MHz, $CDCl_3$) of **14**.

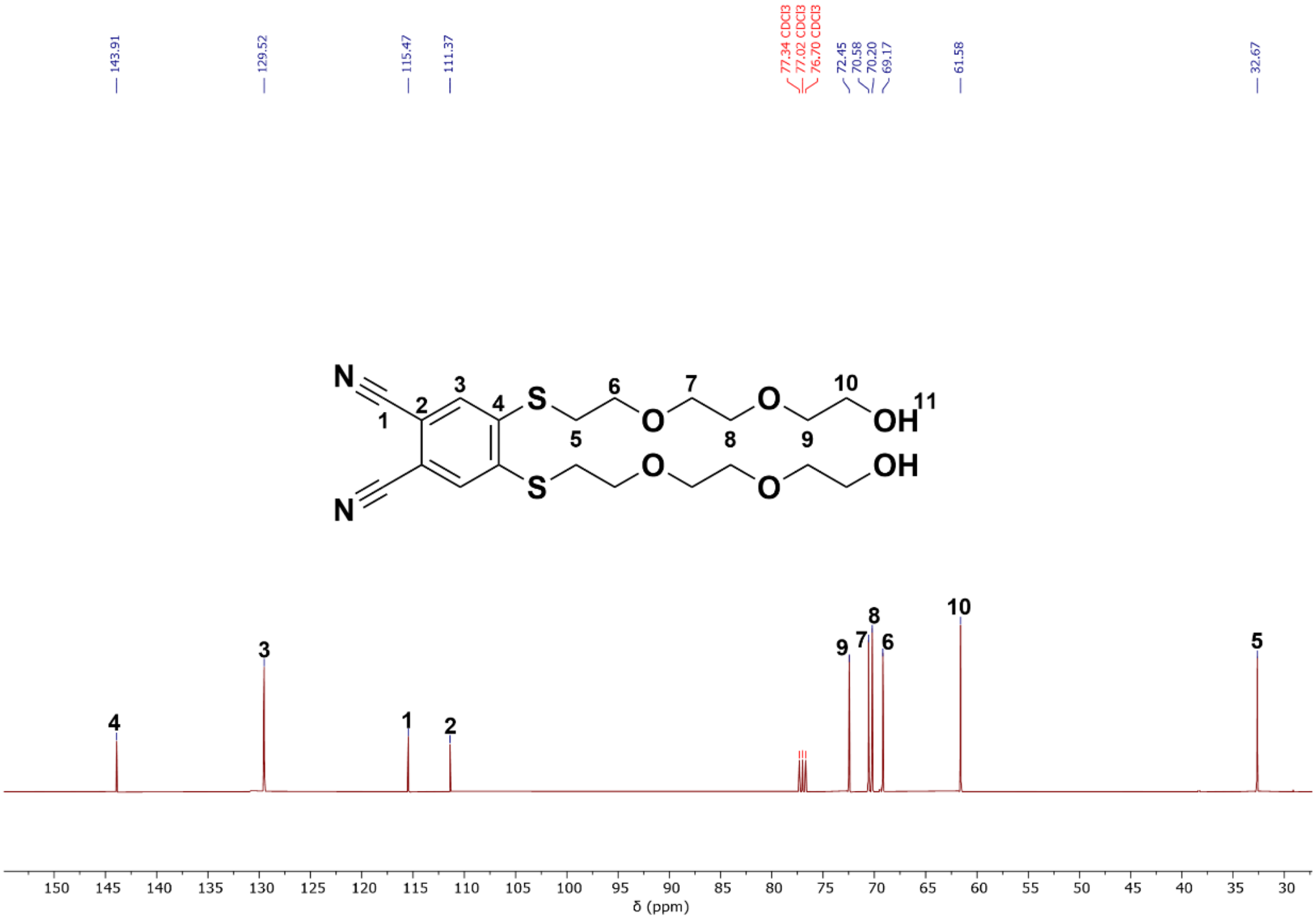

Figure S36: ^{13}C-{^{1}H}-NMR spectrum (101 MHz, $CDCl_3$) of **14**.

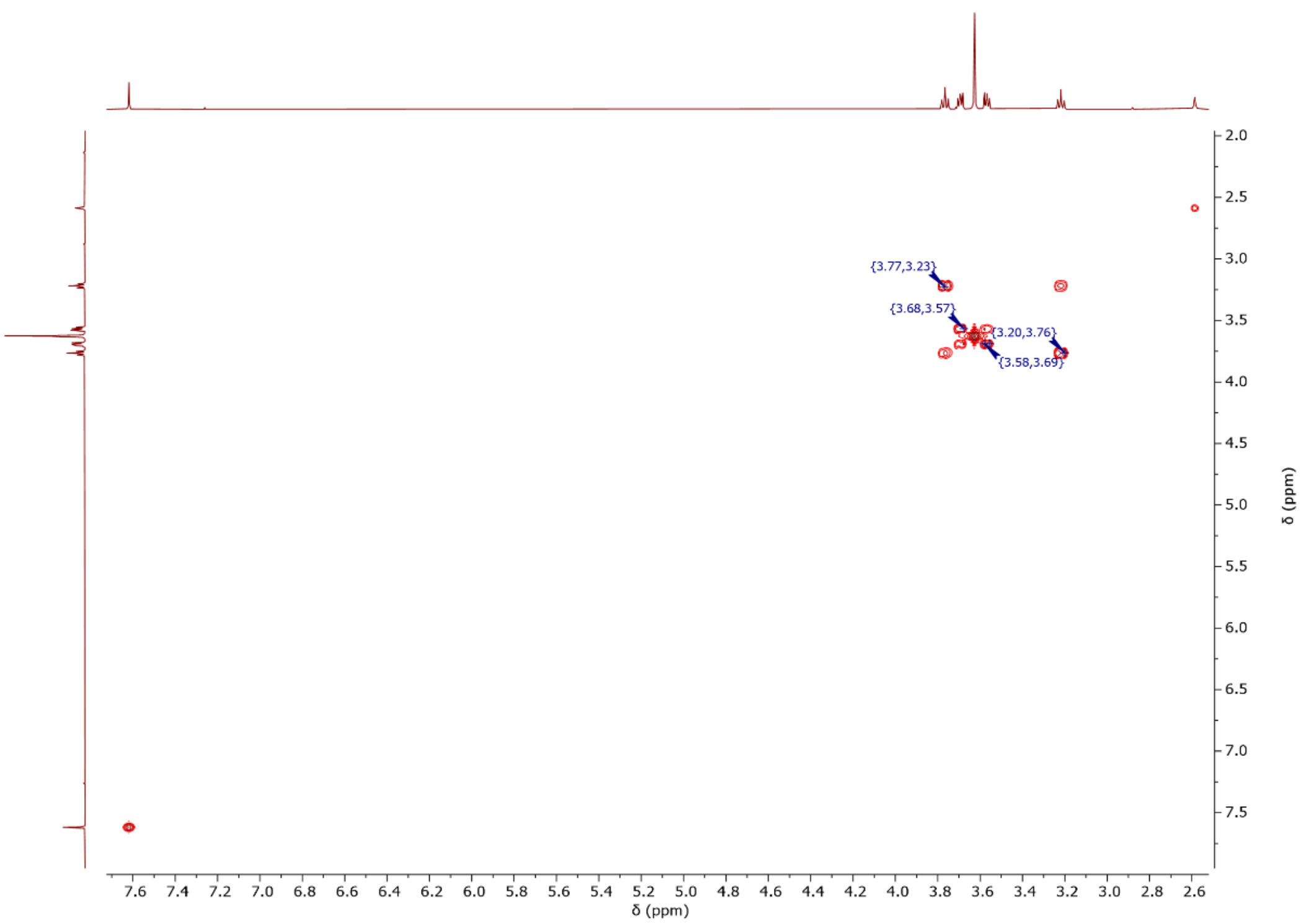

Figure S37: ^{1}H-^{1}H-COSY-NMR spectrum (400 MHz, $CDCl_3$) of **14**.

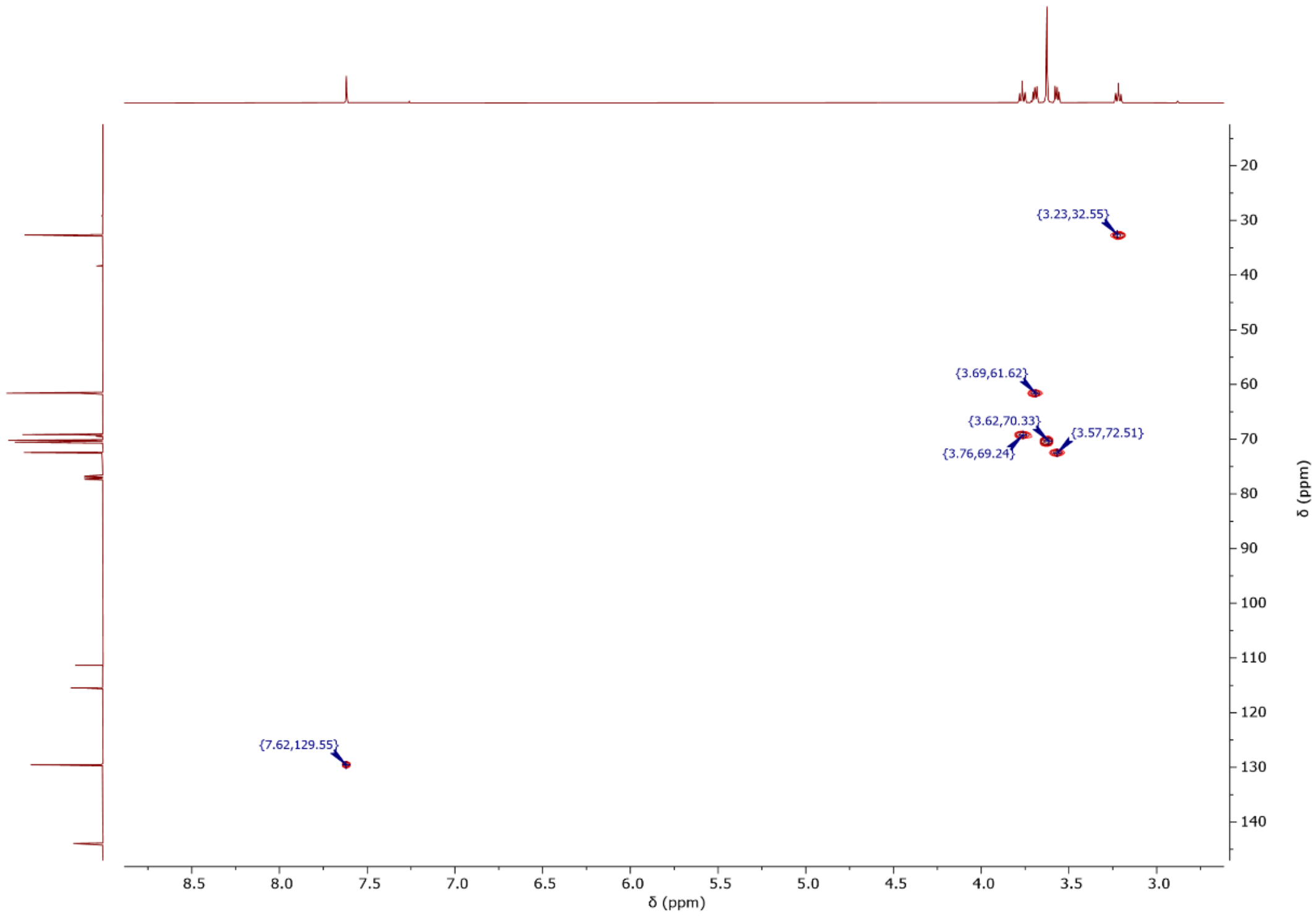

Figure S38: $^{1}H/^{13}C$-gHSQC-NMR spectrum (400 MHz/101 MHz, $CDCl_3$) of **14**.

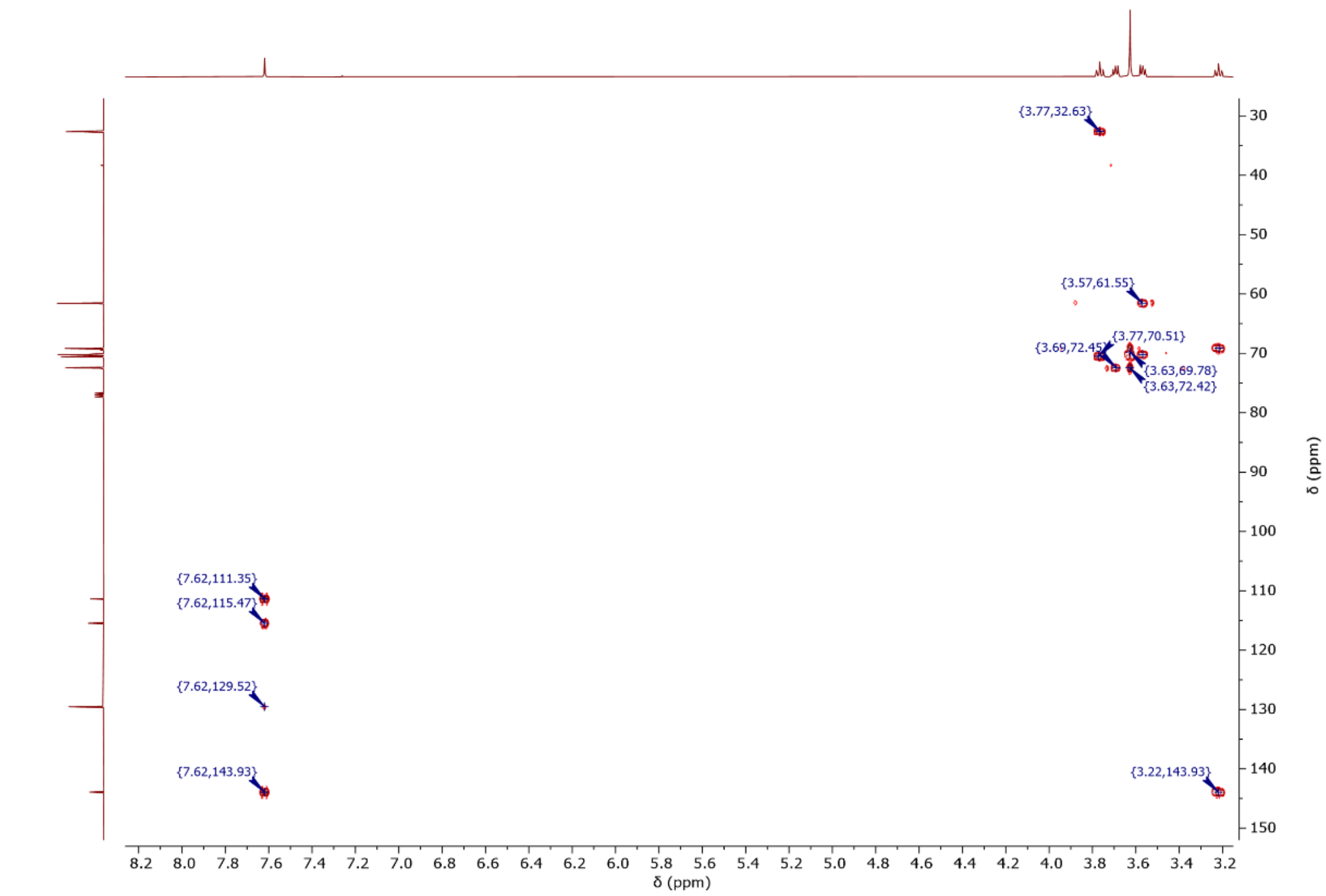

Figure S39: $^{1}H/^{13}C$-gHMBC-NMR spectrum (400 MHz/101 MHz, $CDCl_3$) of **14**.

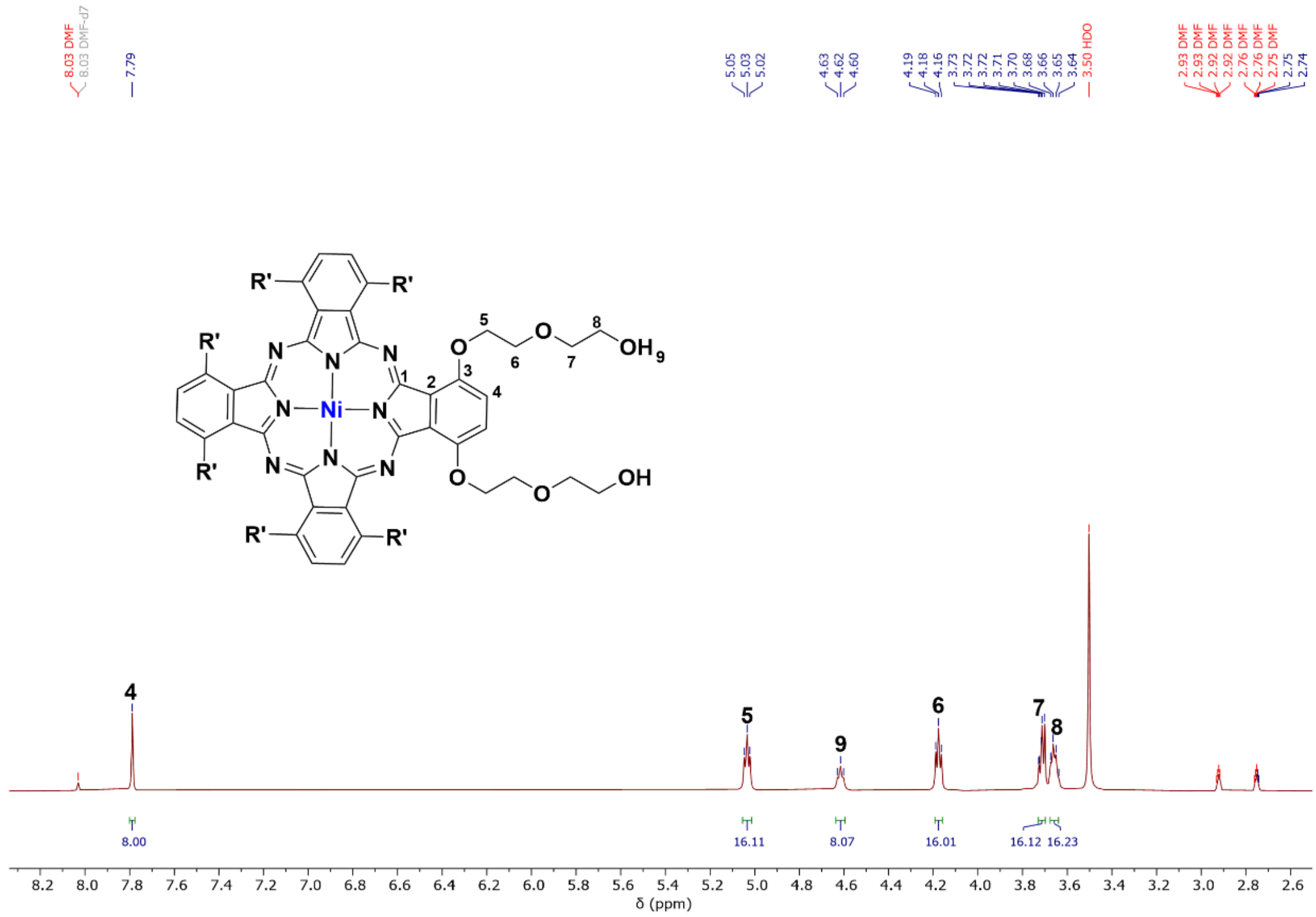

Figure S40: ^{1}H-NMR spectrum (400 MHz, DMF-d_7) of **NiPc-1**.

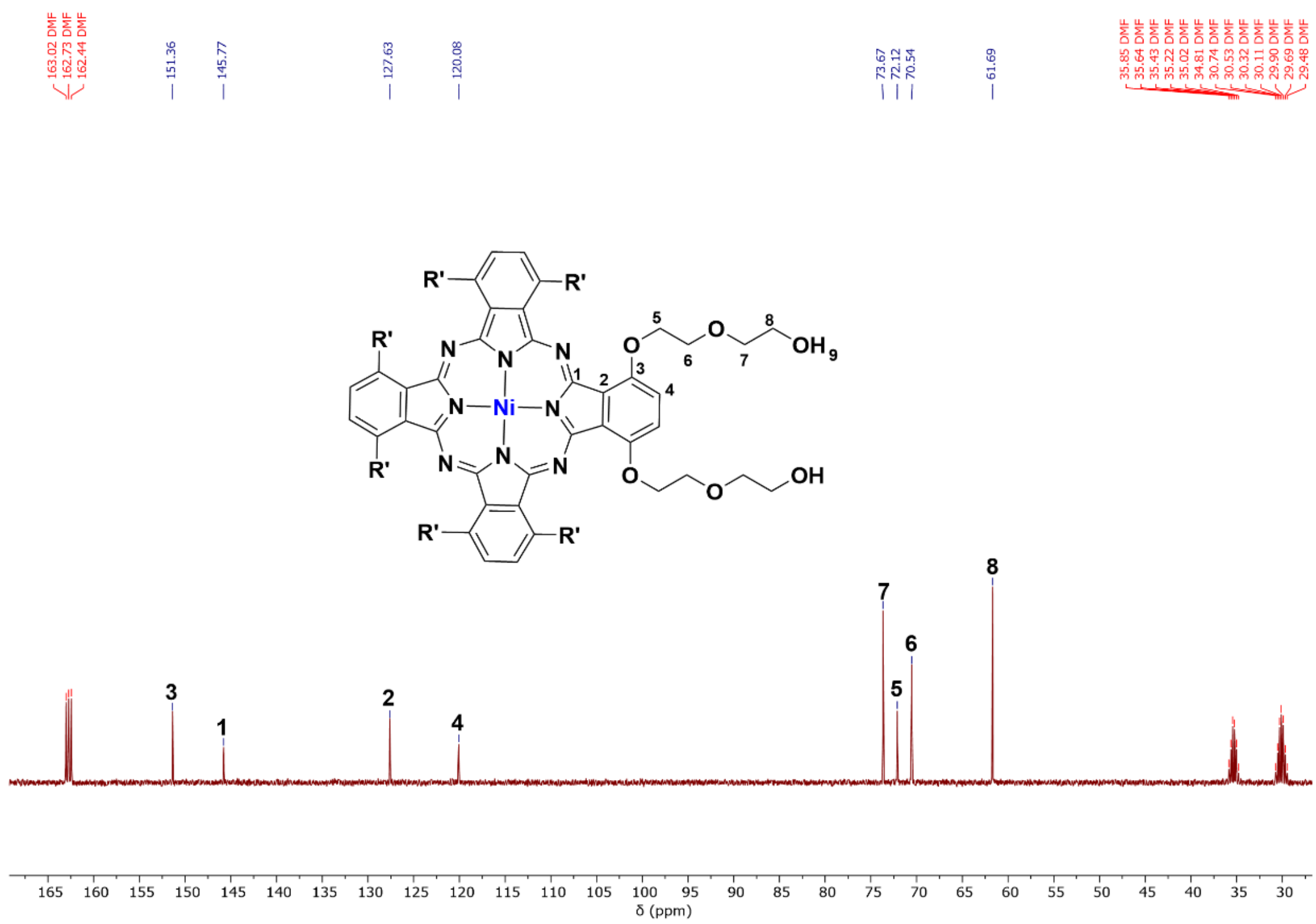

Figure S41: ^{13}C-{^{1}H}-NMR spectrum (101 MHz, DMF-d_7) of **NiPc-1**.

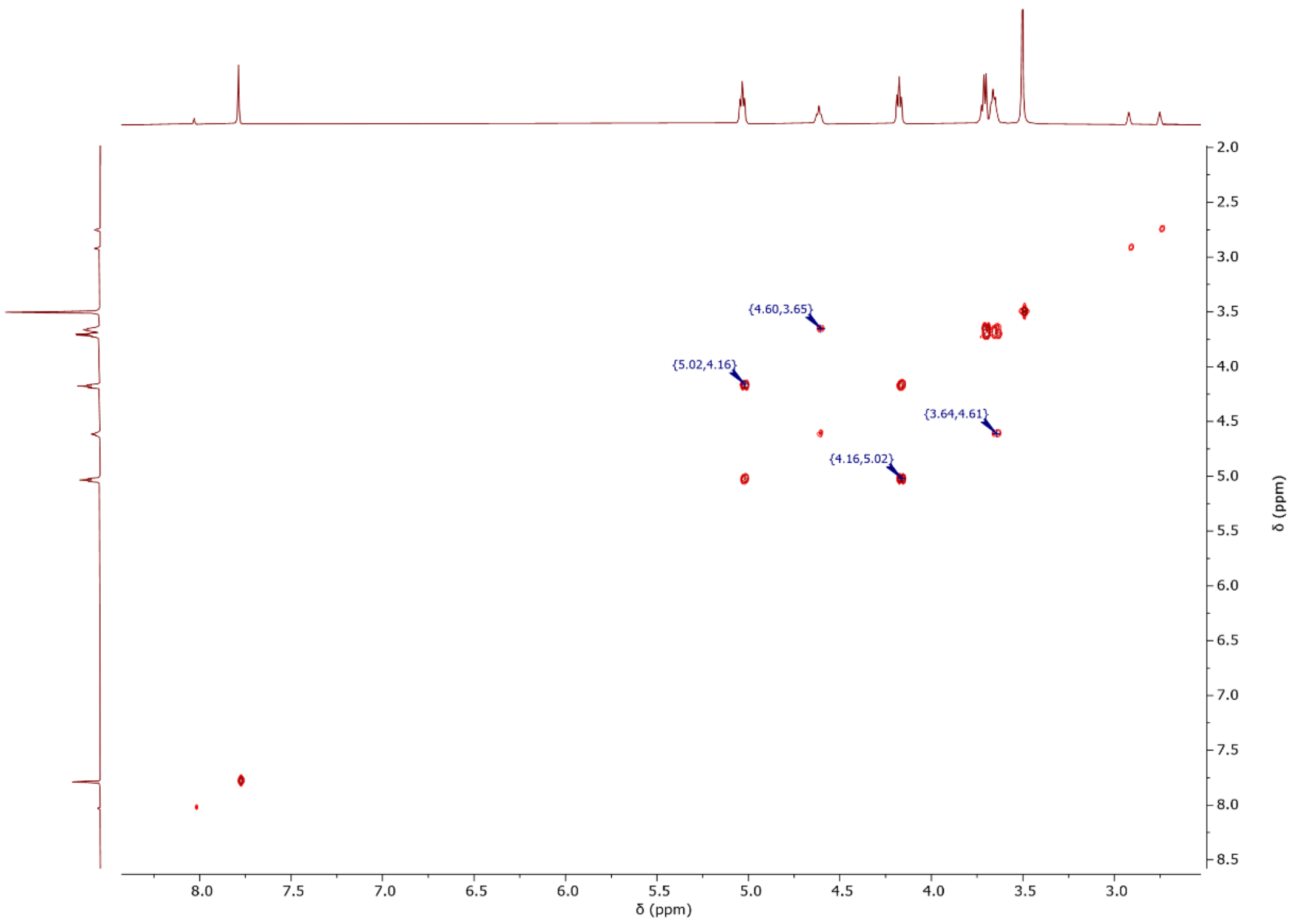

Figure S42: ^{1}H-^{1}H-COSY-NMR spectrum (400 MHz, DMF-d_7) of **NiPc-1**.

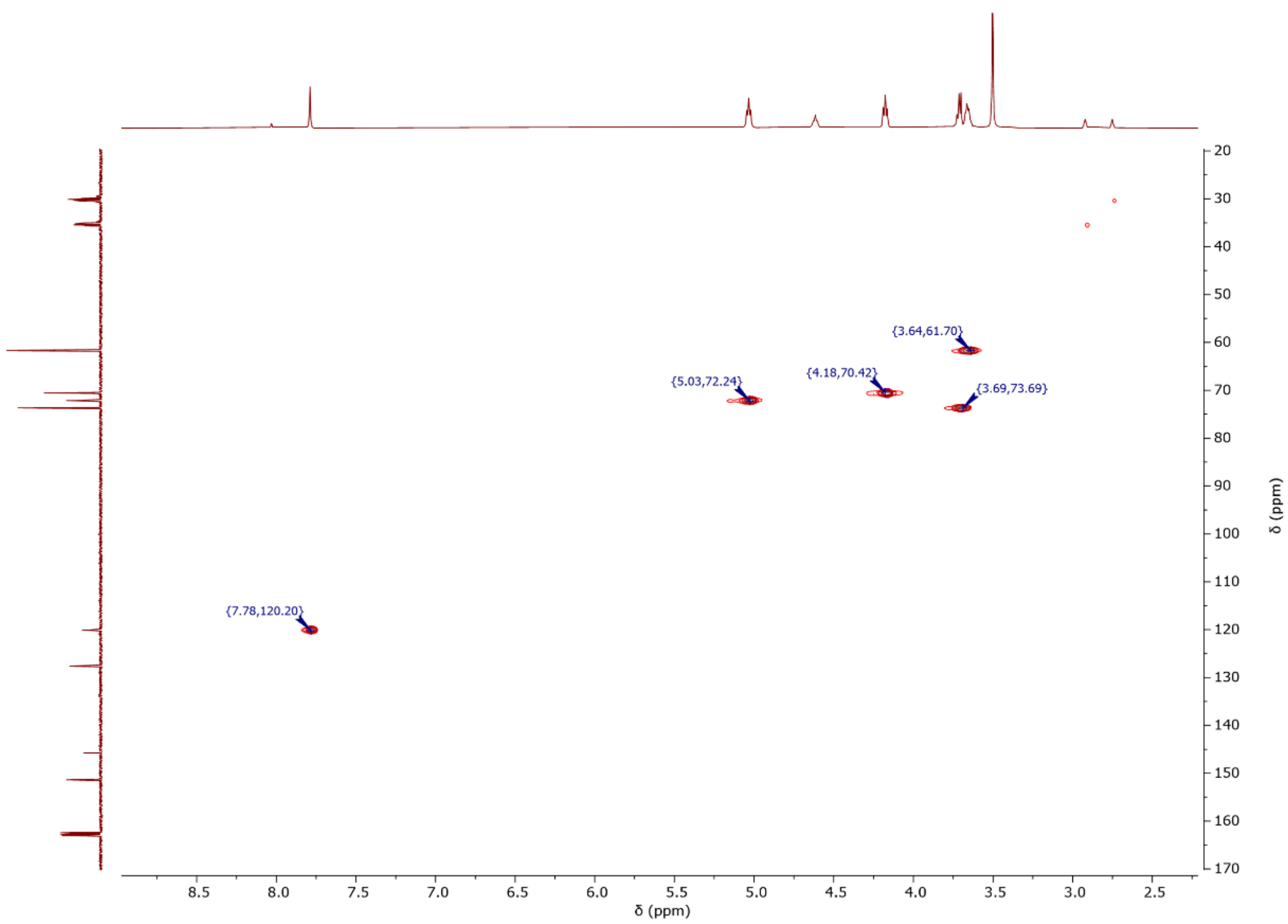

Figure S43: ^{1}H/^{13}C-gHSQC-NMR spectrum (400 MHz/101 MHz, DMF-d_7) of **NiPc-1**.

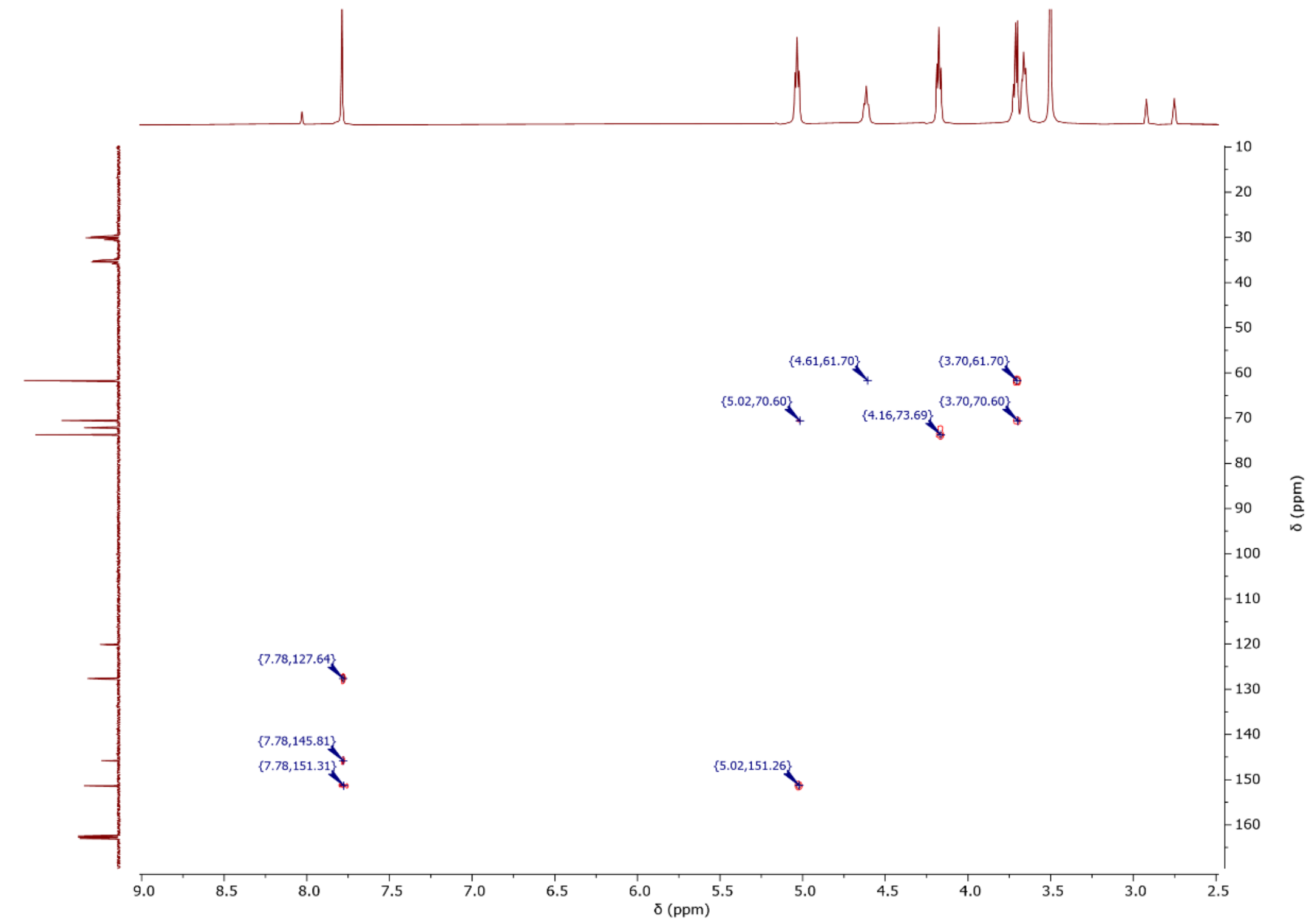

Figure S44: $^{1}H/^{13}C$-gHMBC-NMR spectrum (400 MHz/101 MHz, DMF-d_7) of **NiPc-1**.

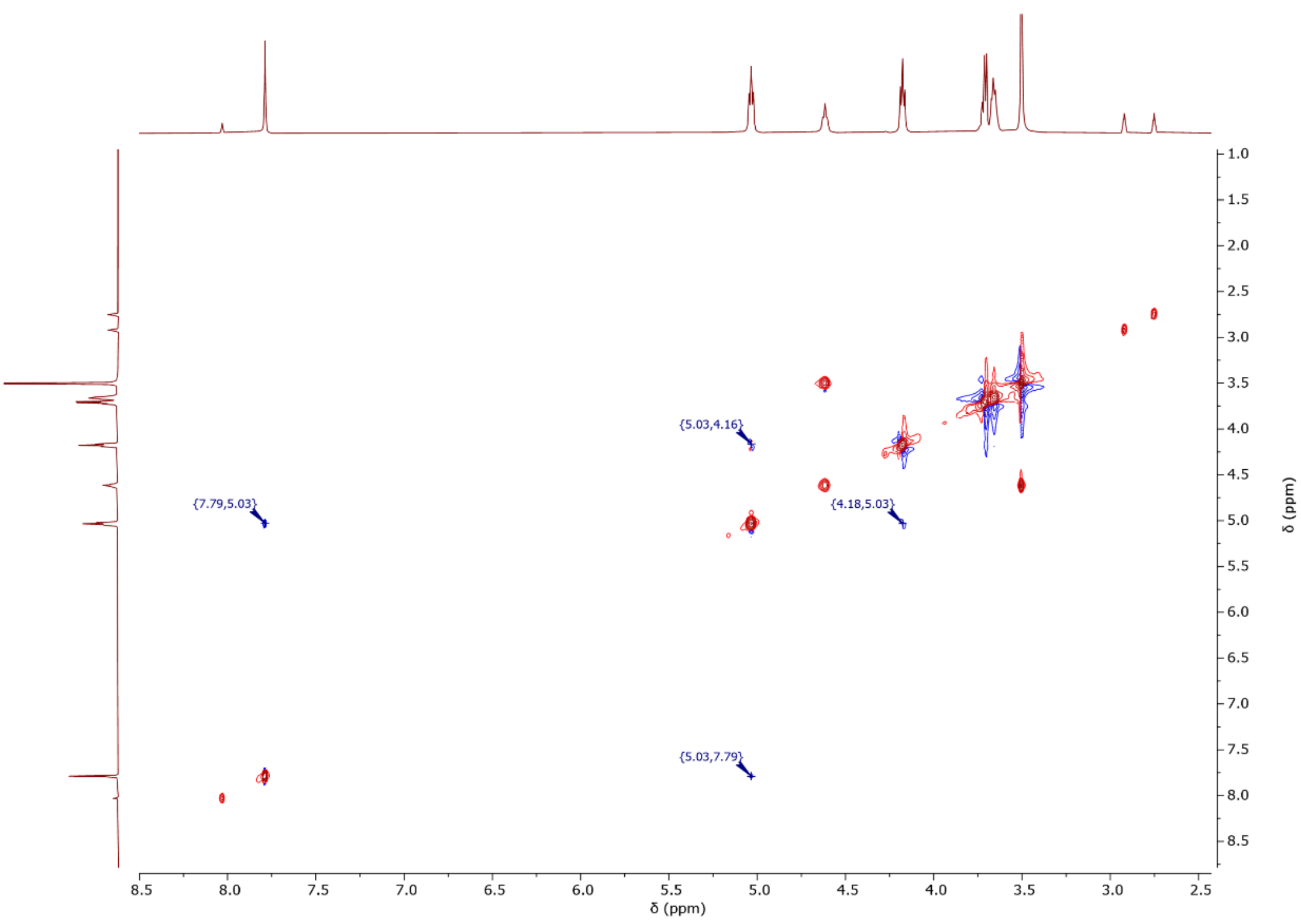

Figure S45: ^{1}H-^{1}H-ROESY-NMRspectrum (400 MHz, DMF-d_7) of **NiPc-1**.

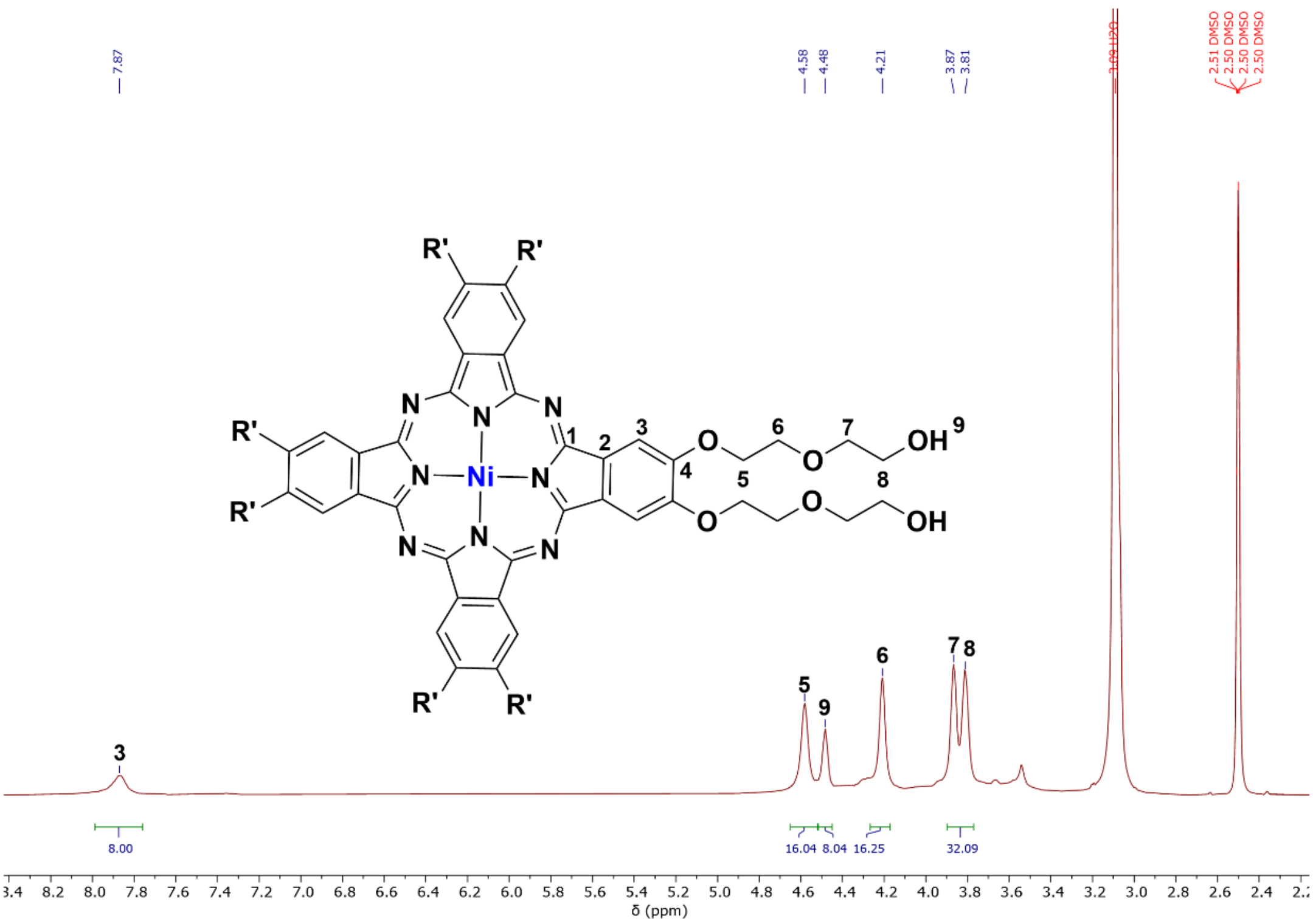

Figure S46: ^{1}H-NMR spectrum (500 MHz, DMSO-d_6, 350 K) of **NiPc-2**.

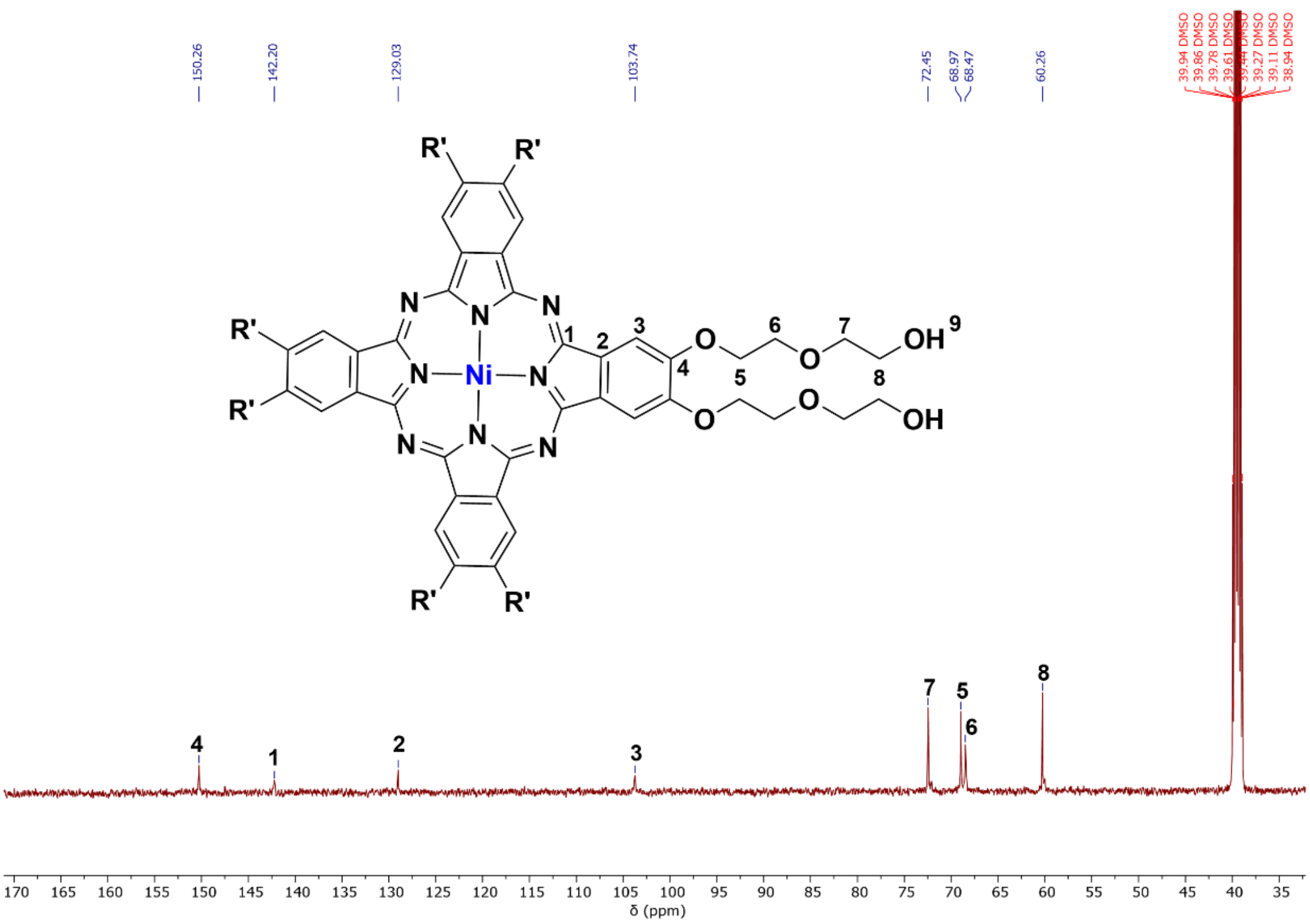

Figure S47: ^{13}C-{^{1}H}-NMR spectrum (126 MHz, DMSO-d_6, 350 K) of **NiPc-2**.

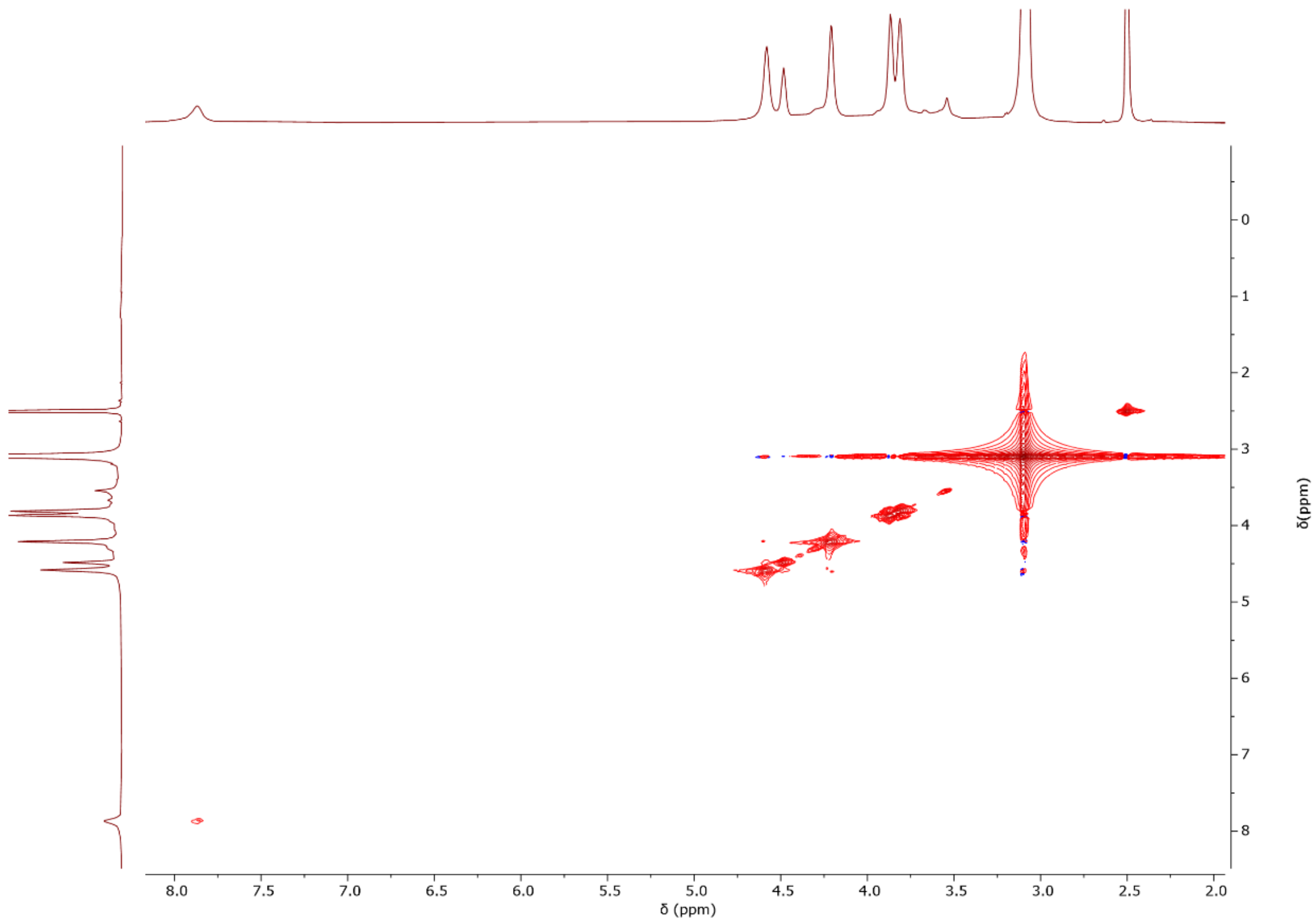

Figure S48: ^{1}H-^{1}H-COSY-NMR spectrum (500 MHz, DMSO-d_6, 350 K) **NiPc-2**.

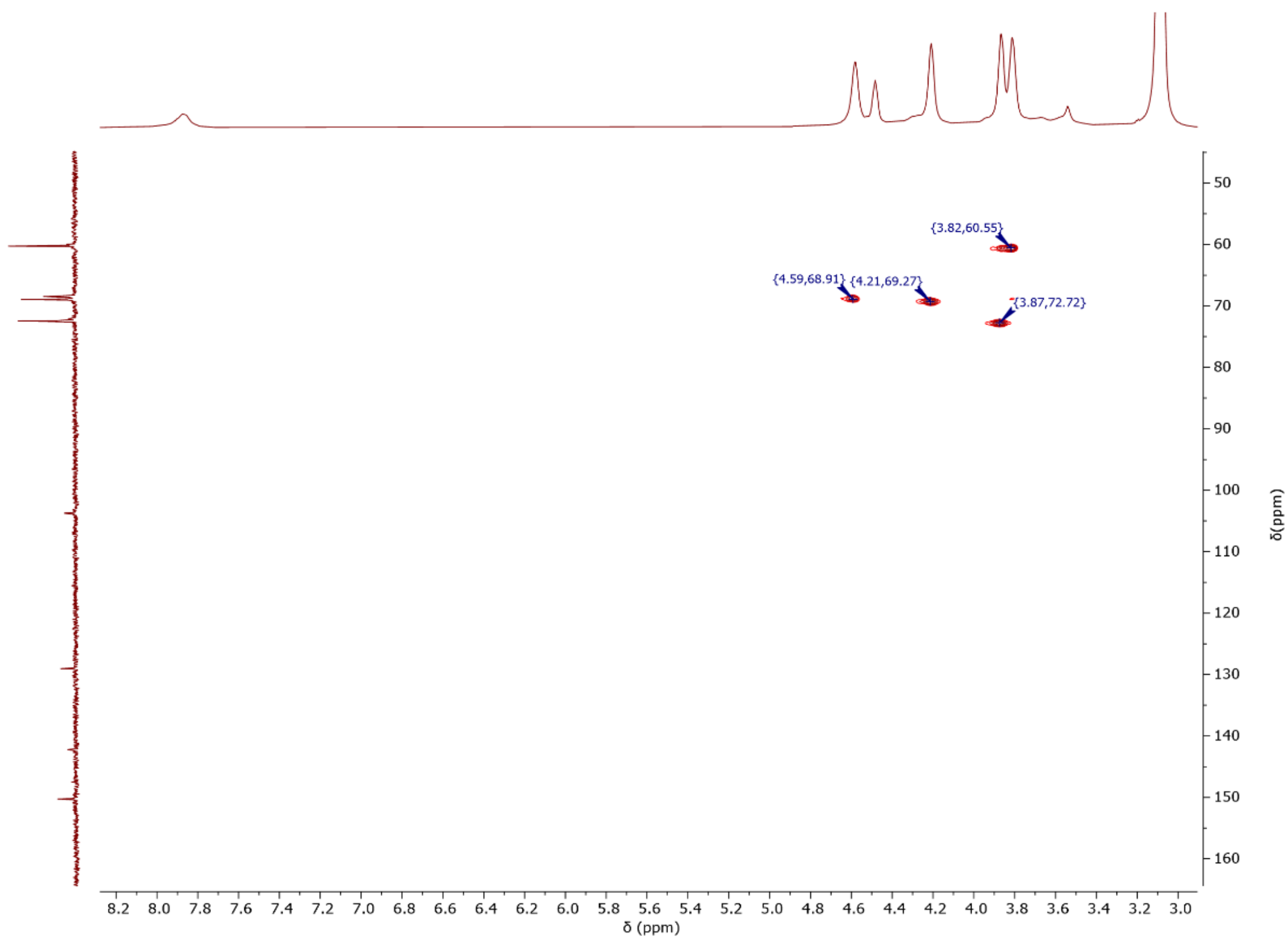

Figure S49: ^{1}H/^{13}C-gHSQC-NMR spectrum (500 MHz/126 MHz, DMSO-d_6, 350 K) of **NiPc-2**.

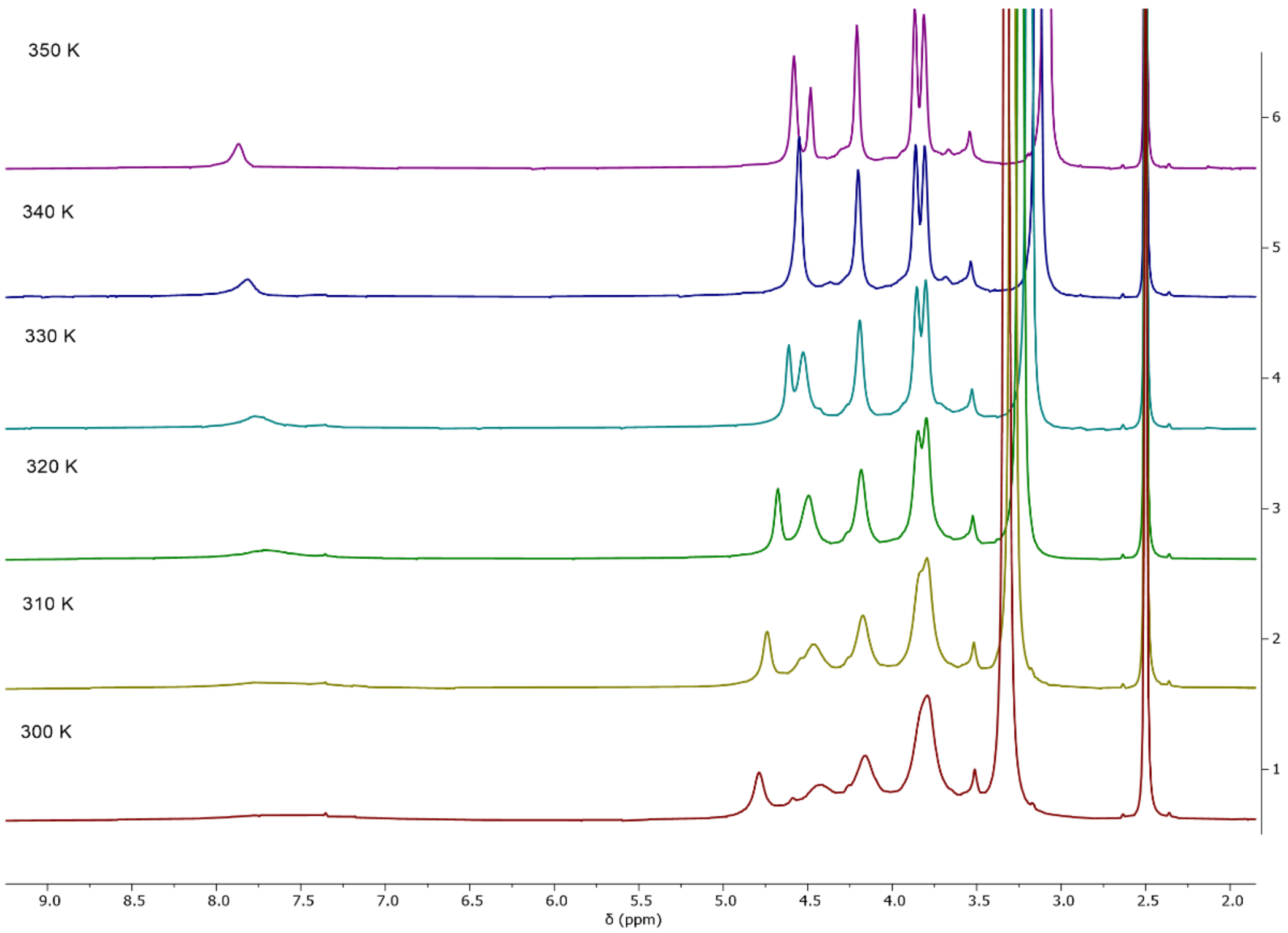

Figure S50: Variable-temperature ^{1}H-NMR spectra (500 MHz DMSO-d_6, 300 – 350 K) of **NiPc-2**.

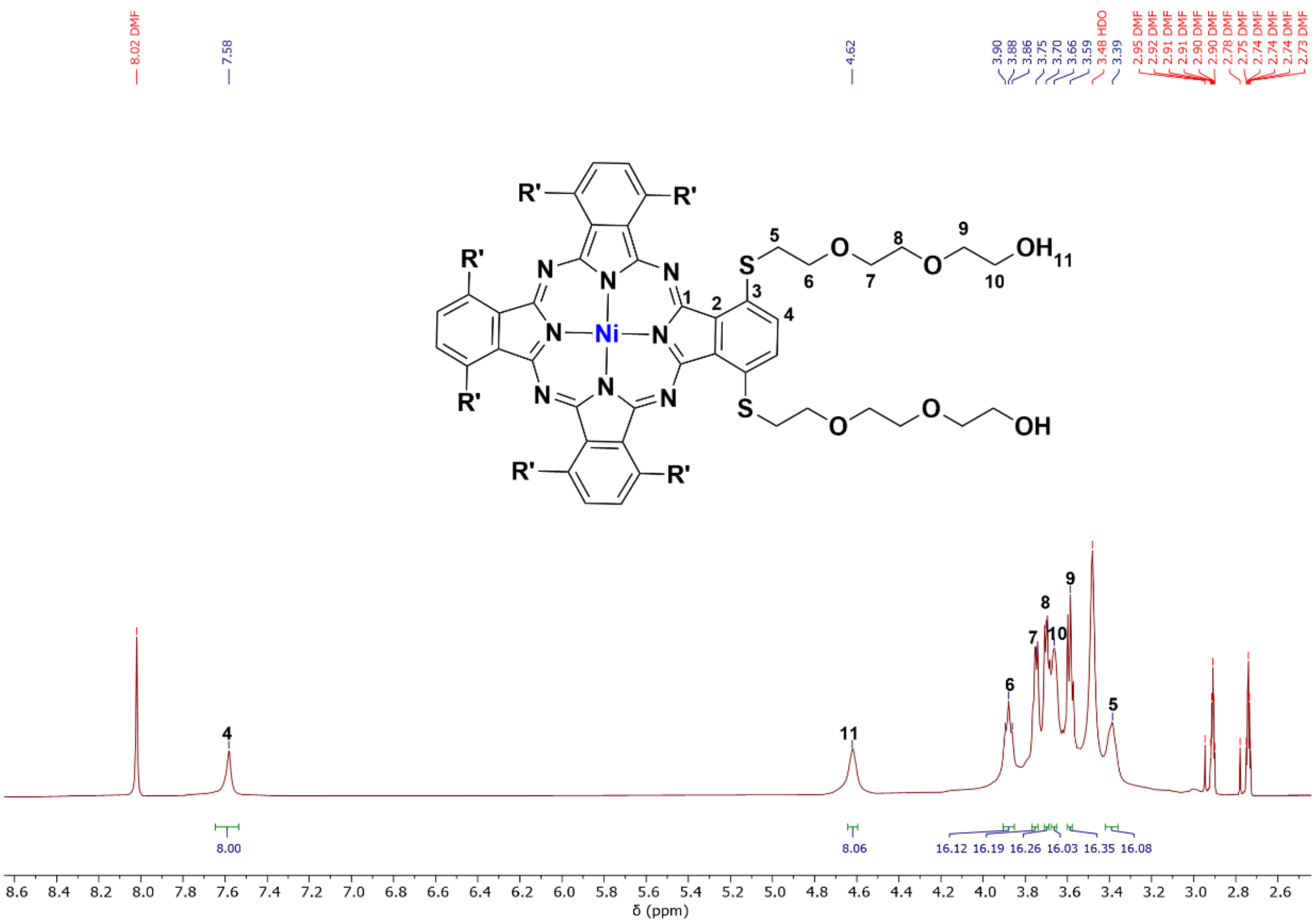

Figure S51: ^{1}H-NMR spectrum (400 MHz, DMF-d_7) of **NiPc-3**.

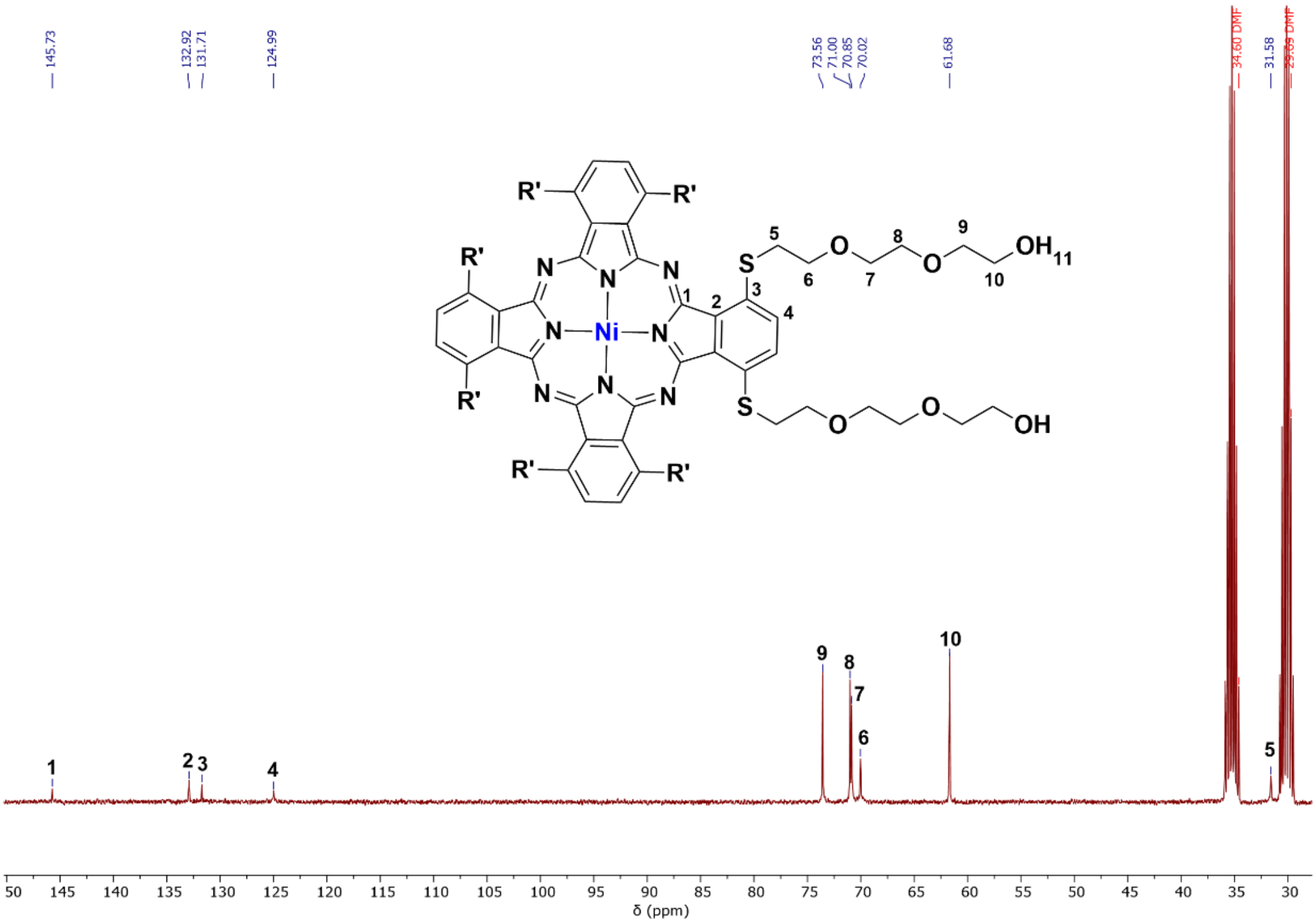

Figure S52: ^{13}C-{^{1}H}-NMR spectrum (101 MHz, DMF-d_7) of **NiPc-3**.

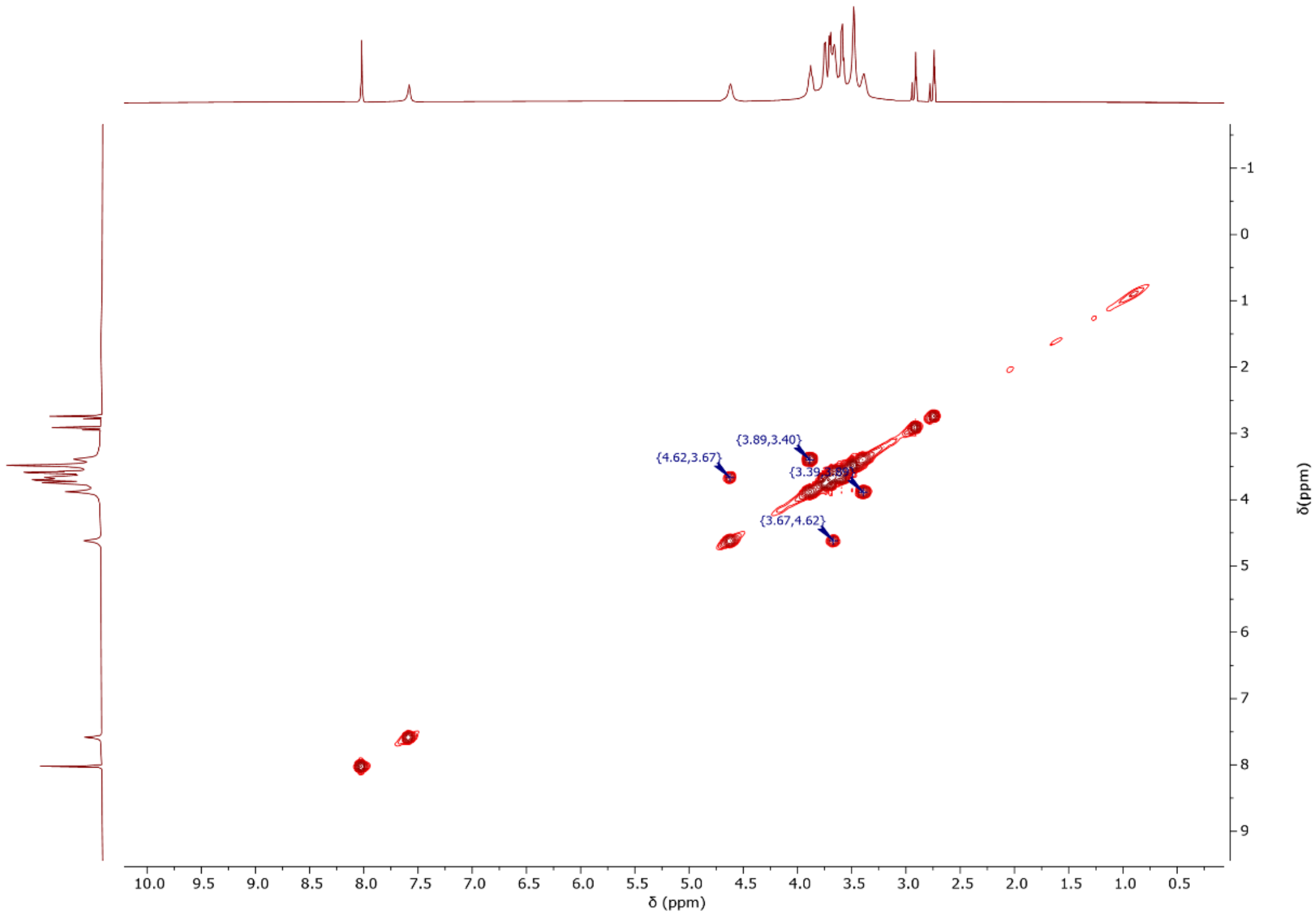

Figure S53: ^{1}H-^{1}H-COSY-NMR spectrum (400 MHz, DMF-d_7) of **NiPc-3**.

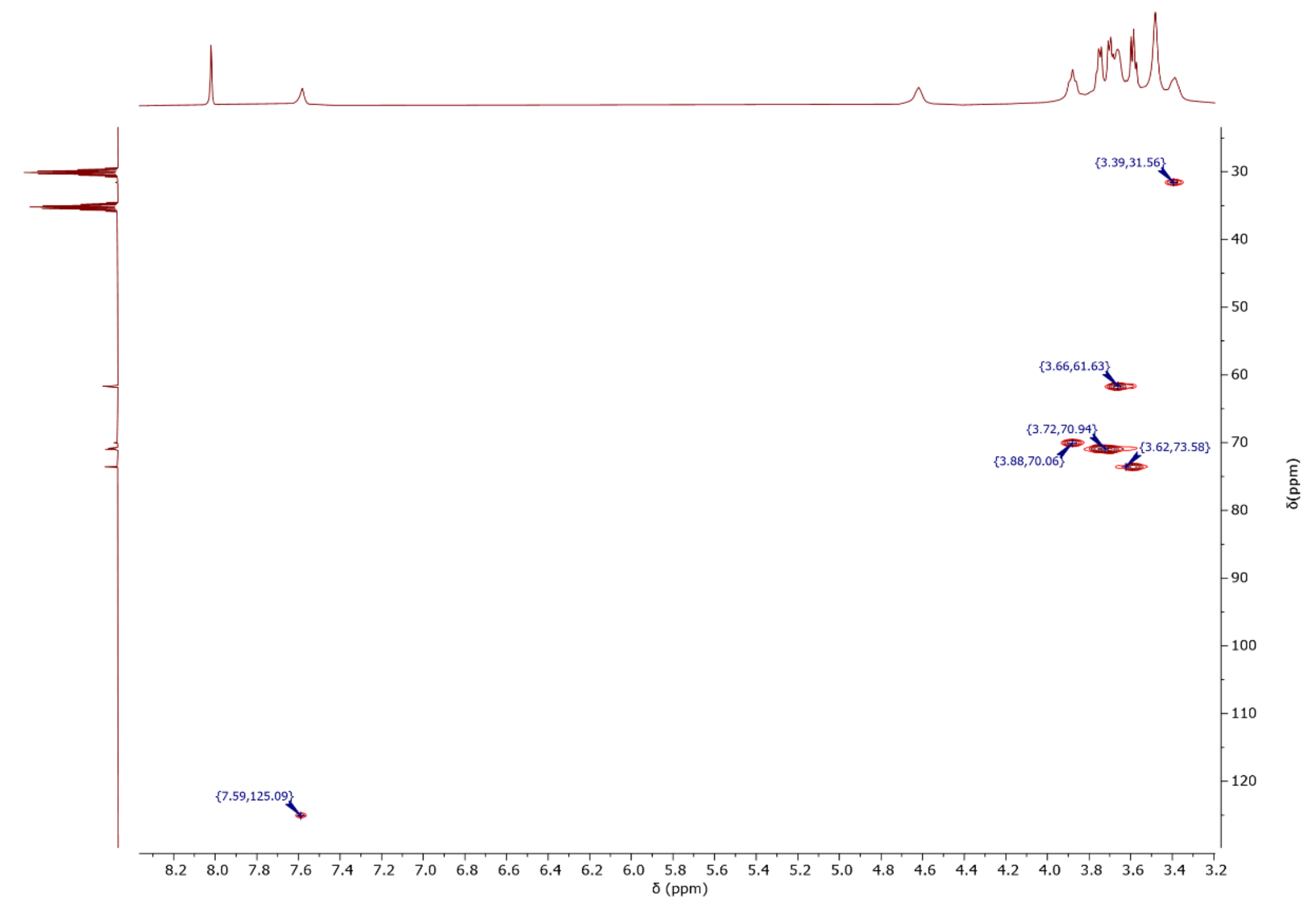

Figure S54: $^{1}H/^{13}C$-gHSQC-NMR spectrum (400 MHz/101 MHz, DMF-d_7) of **NiPc-3**.

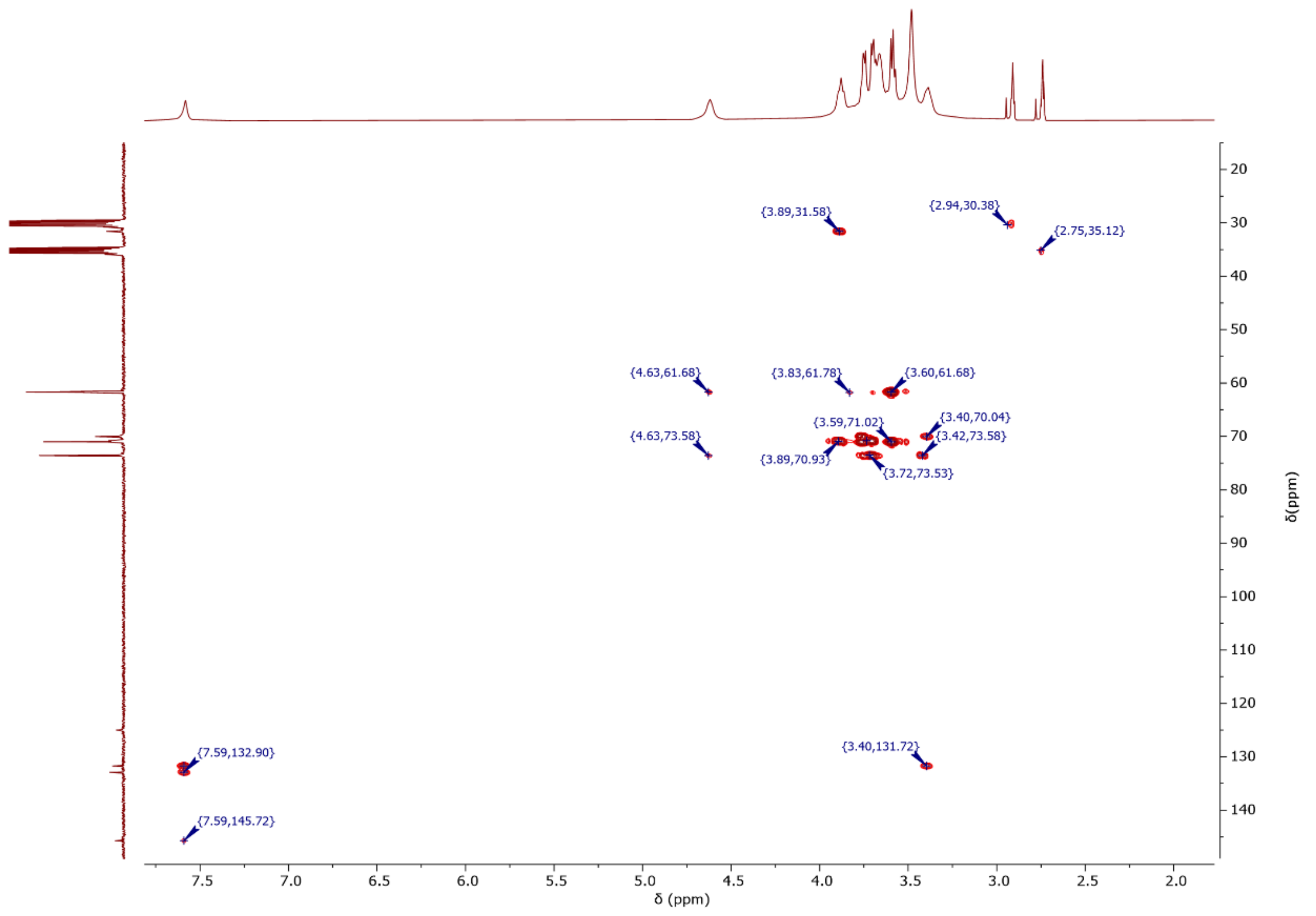

Figure S55: $^{1}H/^{13}C$-gHMBC-NMR spectrum (400 MHz/101 MHz, DMF-d_7) of **NiPc-3**.

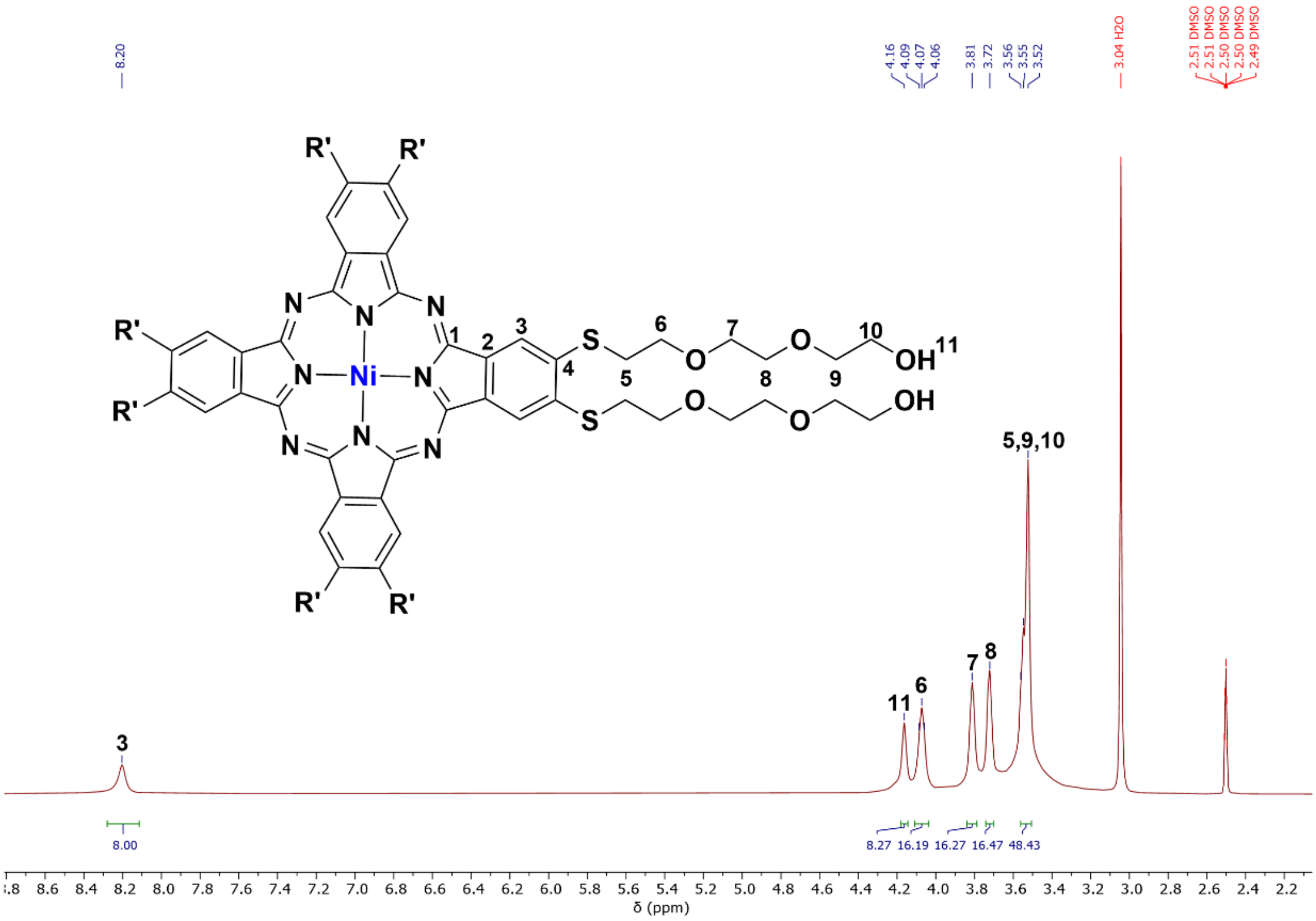

Figure S56: ^{1}H-NMR spectrum (500 MHz, DMSO-d_6, 360 K) of **NiPc-4**.

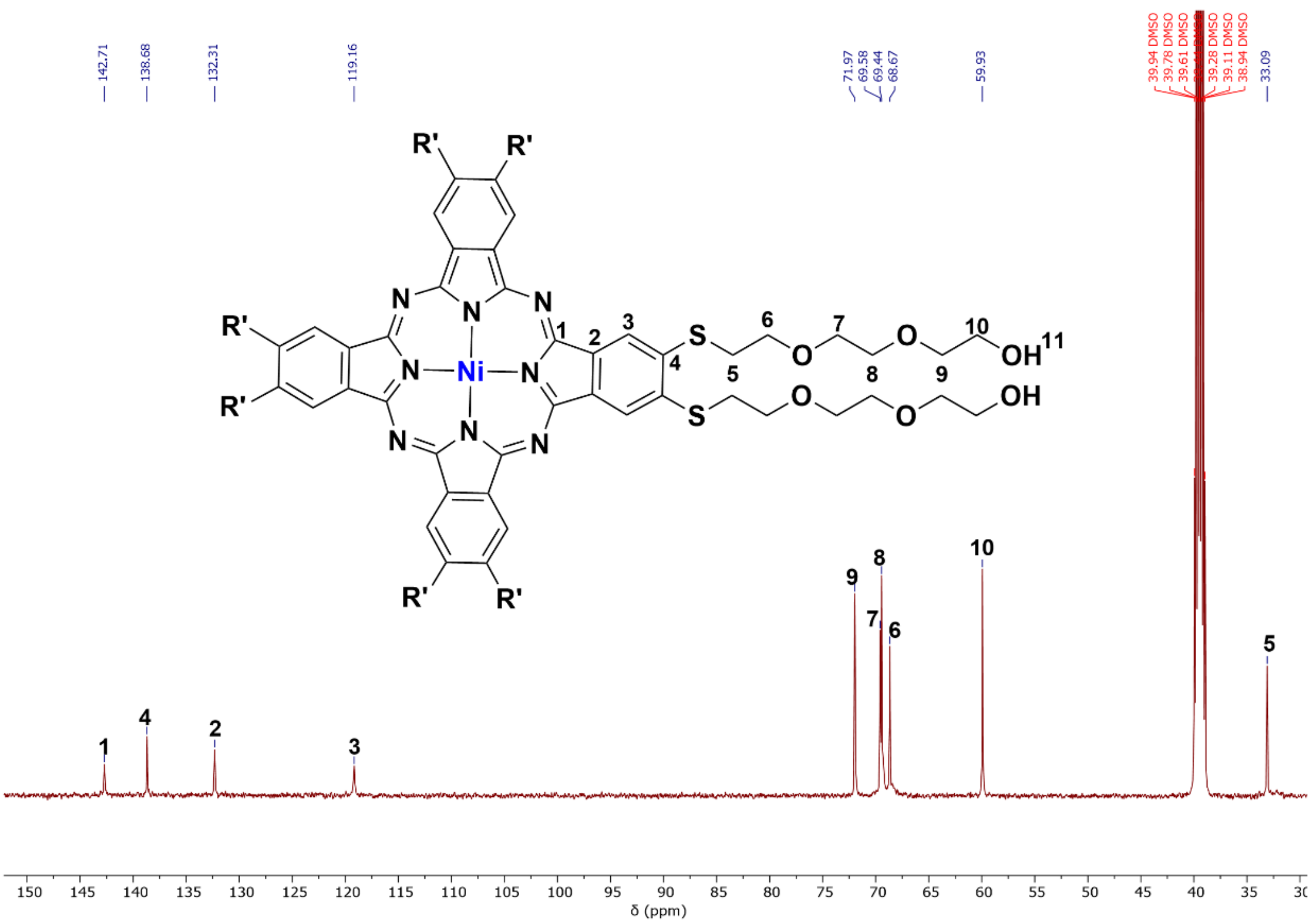

Figure S57: ^{13}C-{^{1}H}-NMR spectrum (126 MHz, DMSO-d_6, 360 K) of **NiPc-4**.

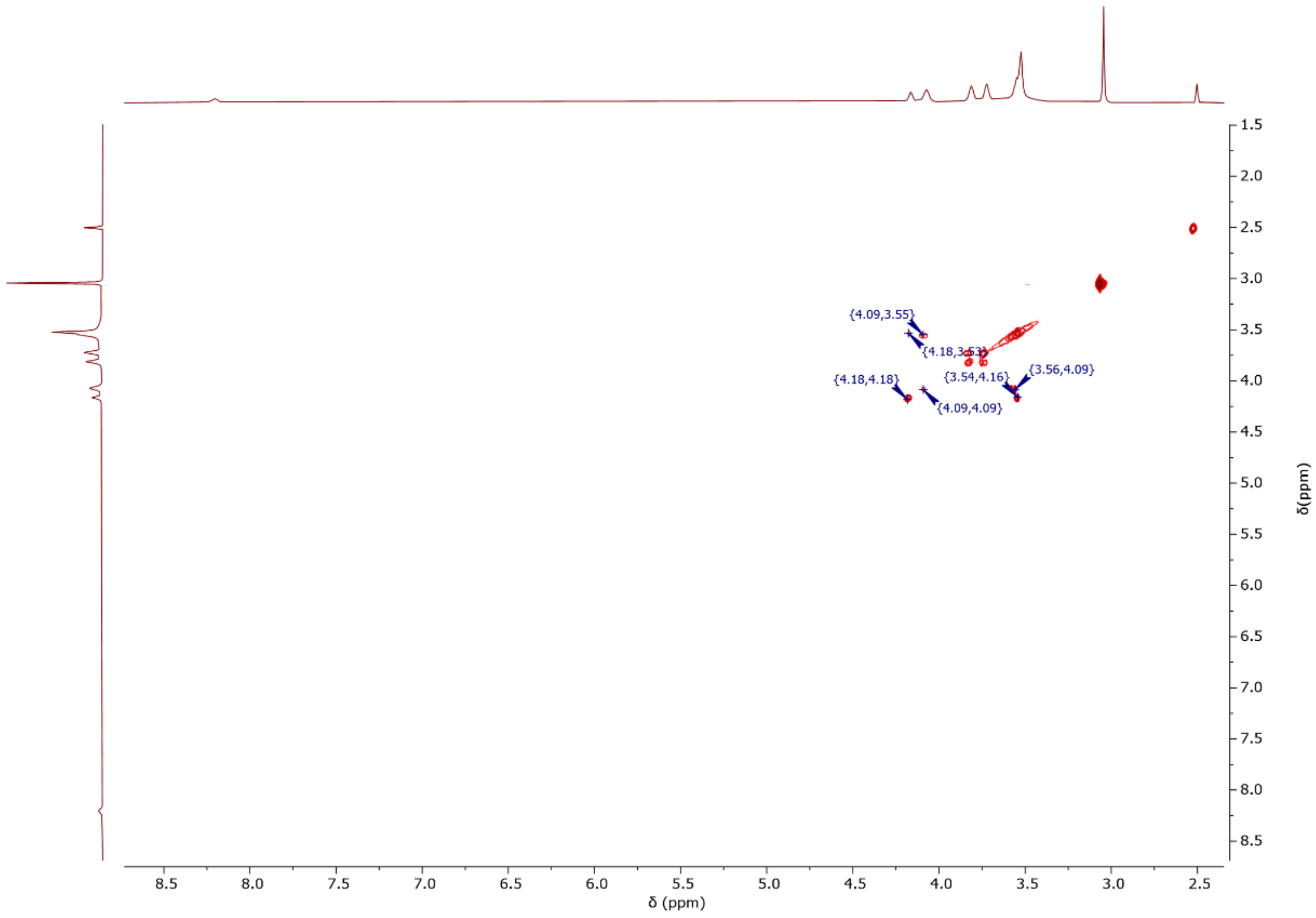

Figure S58: ^{1}H-^{1}H-COSY-NMR spectrum (500 MHz, DMSO-d_6, 360 K) of **NiPc-4**.

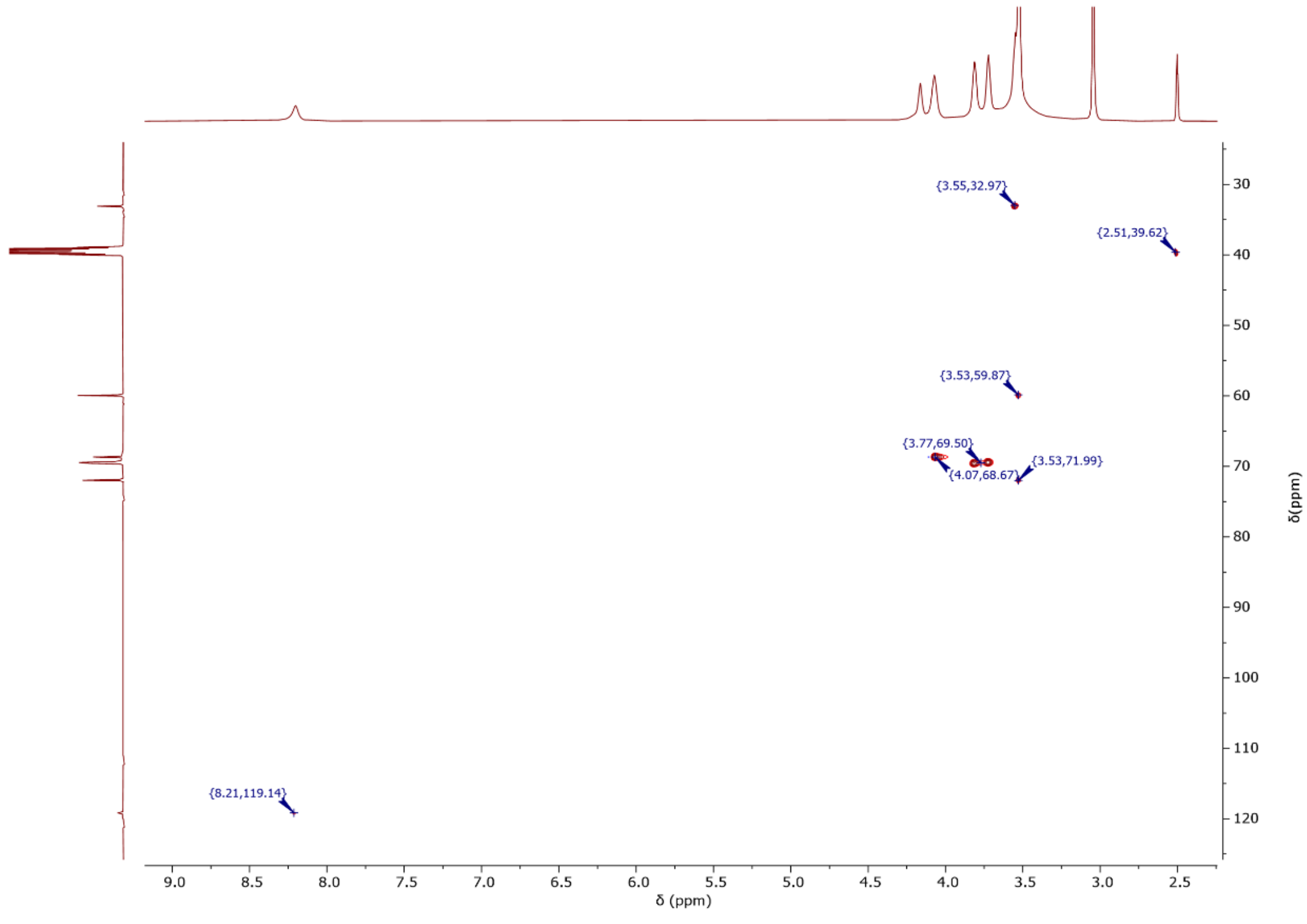

Figure S59: ^{1}H/^{13}C-gHSQC-NMR spectrum (500 MHz/126 MHz, DMSO-d_6, 360 K) **NiPc-4**.

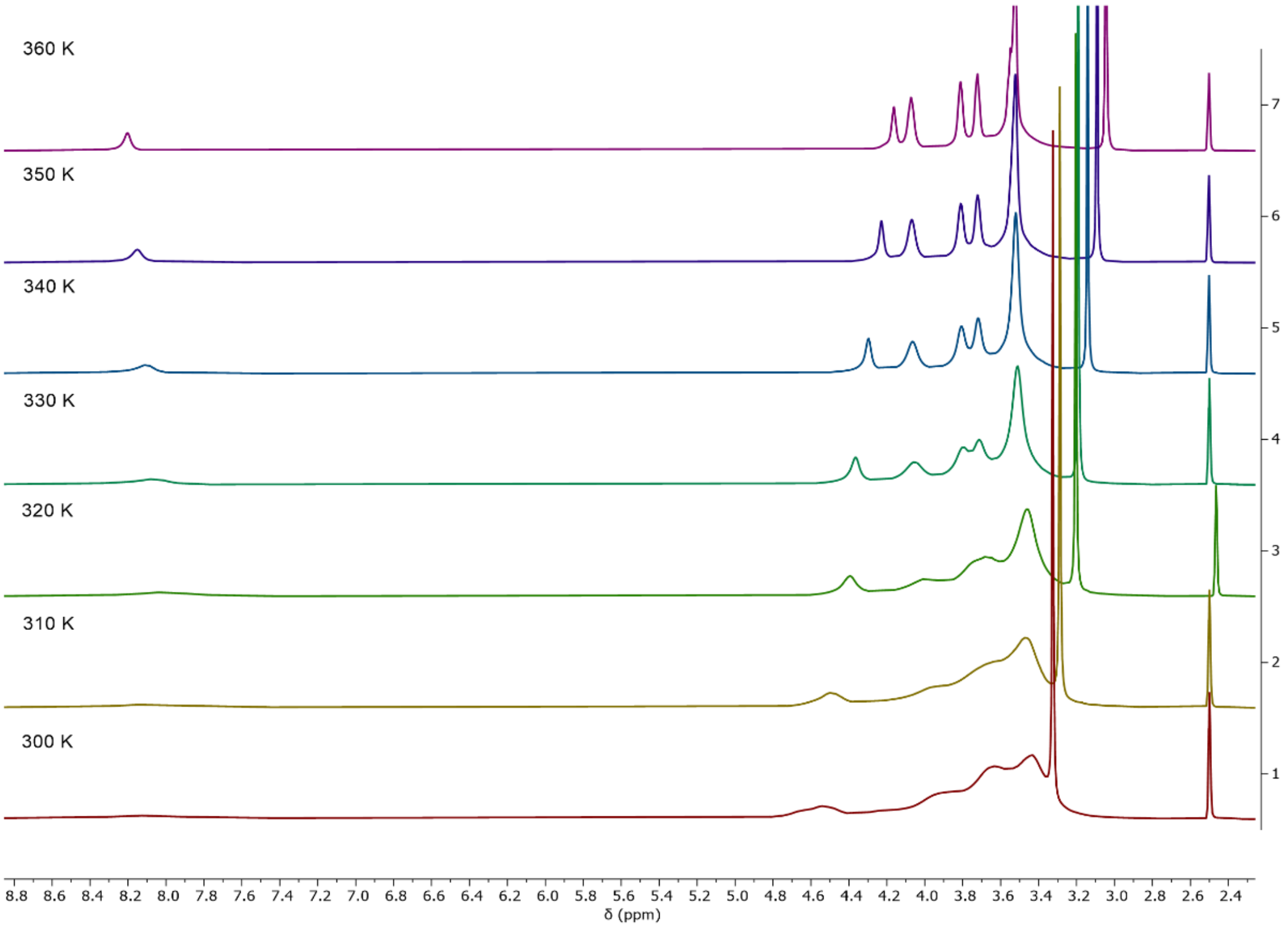

Figure S60: Variable-temperature ^{1}H-NMR spectra (500 MHz DMSO-d_6, 300 – 360 K) **NiPc-4**.

Section S4: Mass spectra

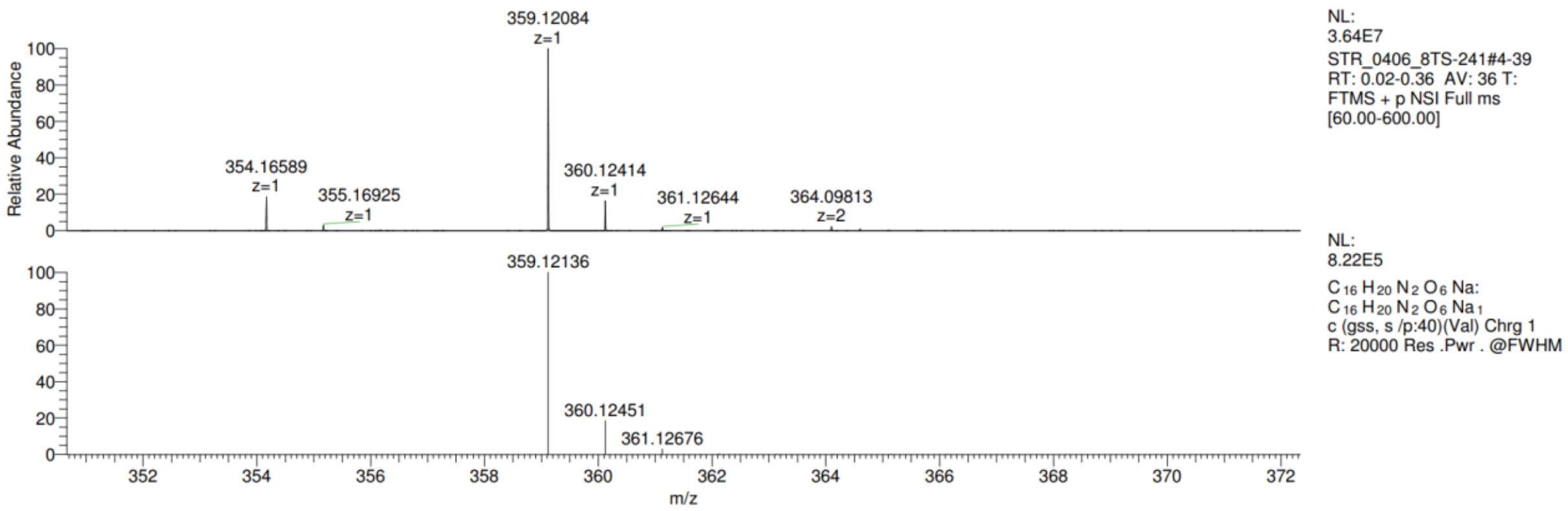

Figure S61: EM-MS-ESI spectrum of **3**.

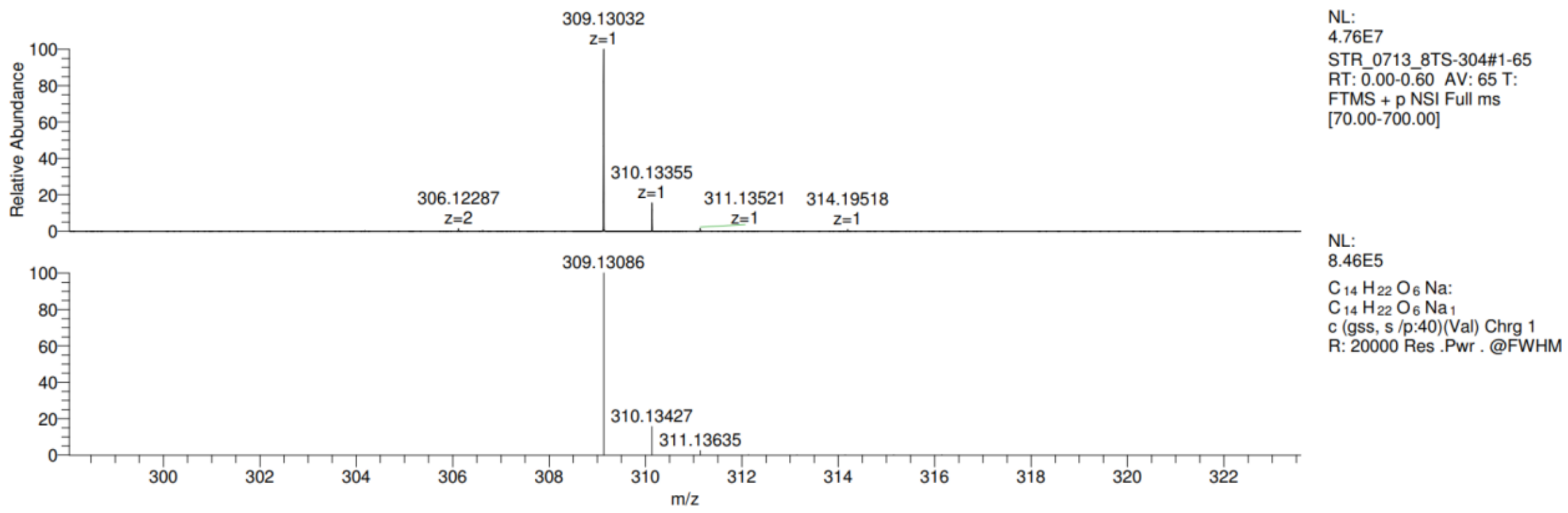

Figure S62: EM-MS-ESI spectrum of **5**.

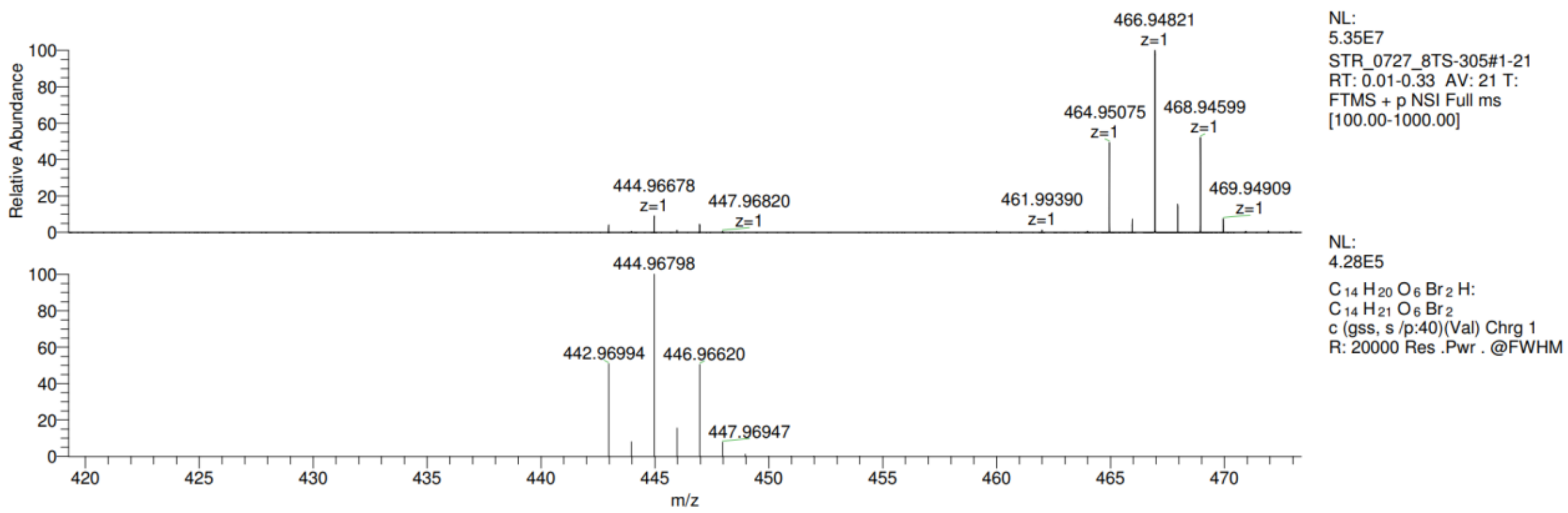

Figure S63: EM-MS-ESI spectrum of **6**.

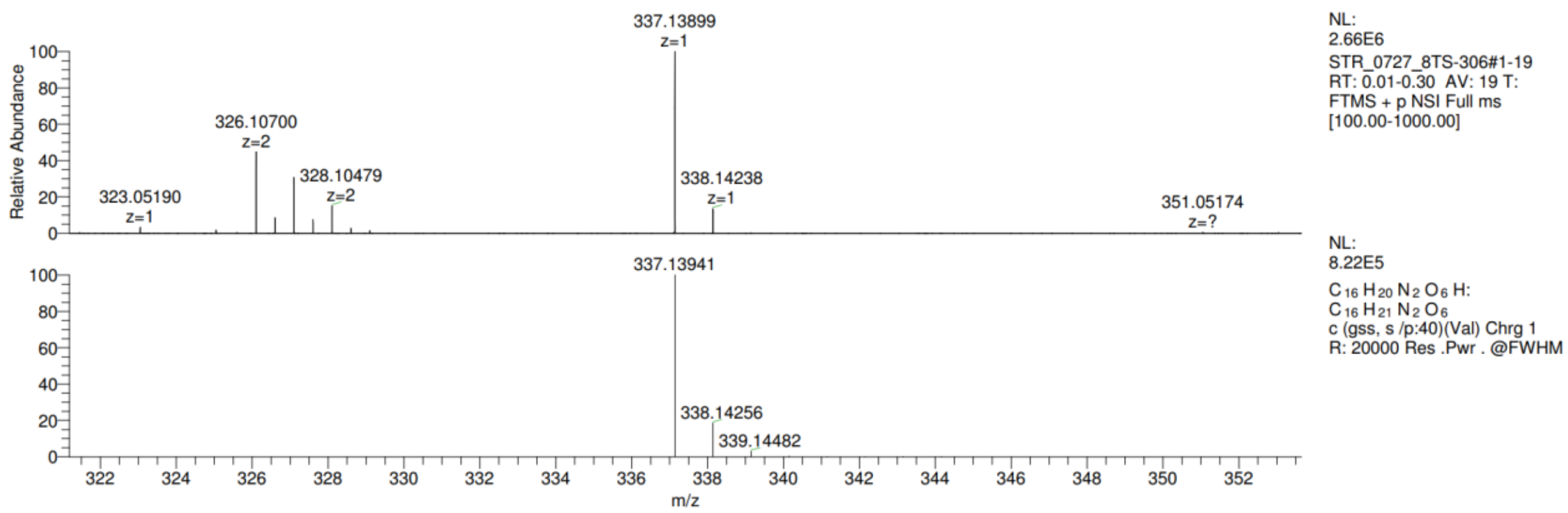

Figure S64: EM-MS-ESI spectrum of **7**.

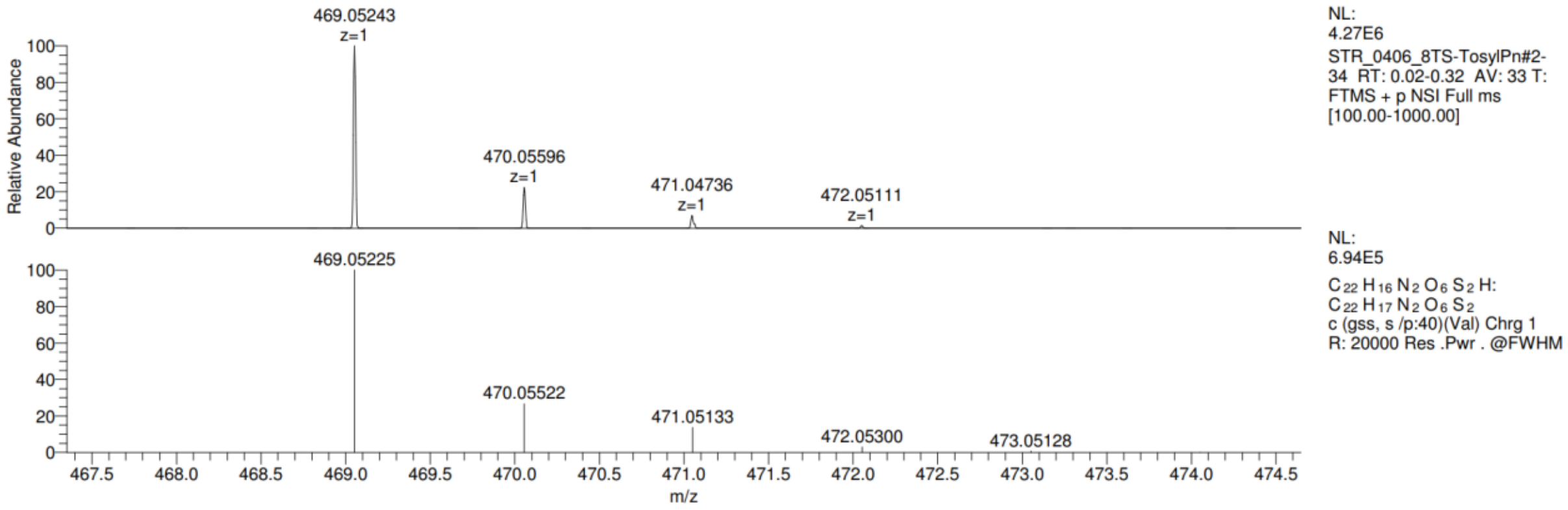

Figure S65: EM-MS-ESI spectrum of **8**.

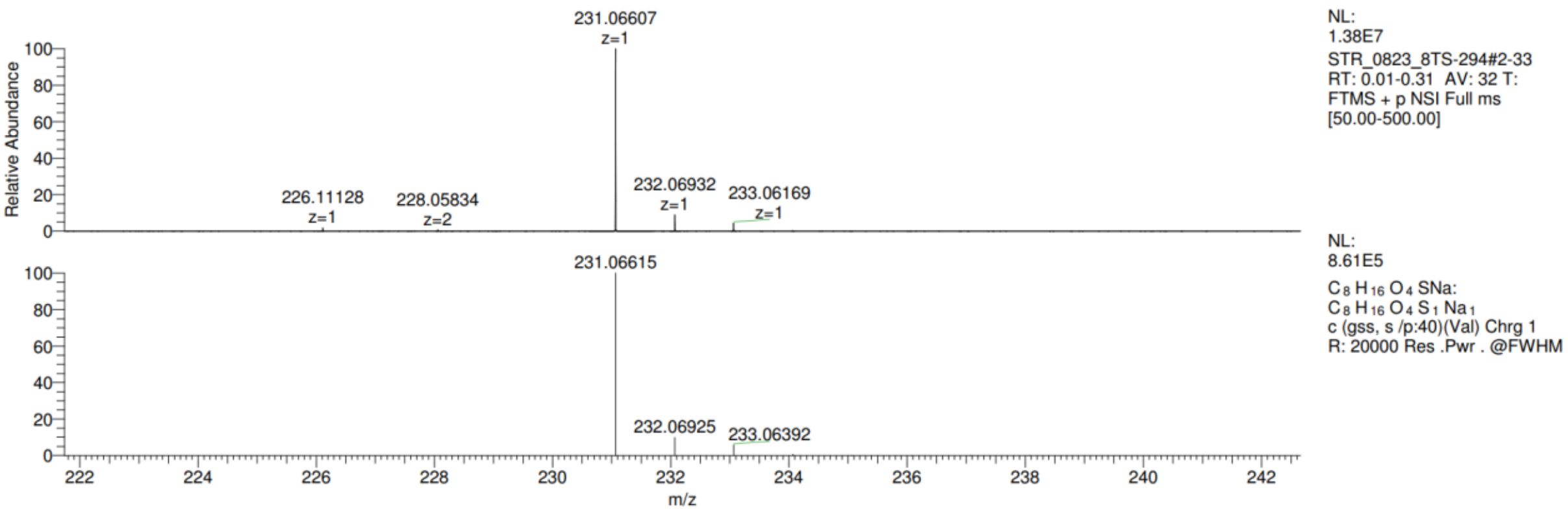

Figure S66: EM-MS-ESI spectrum of **10**.

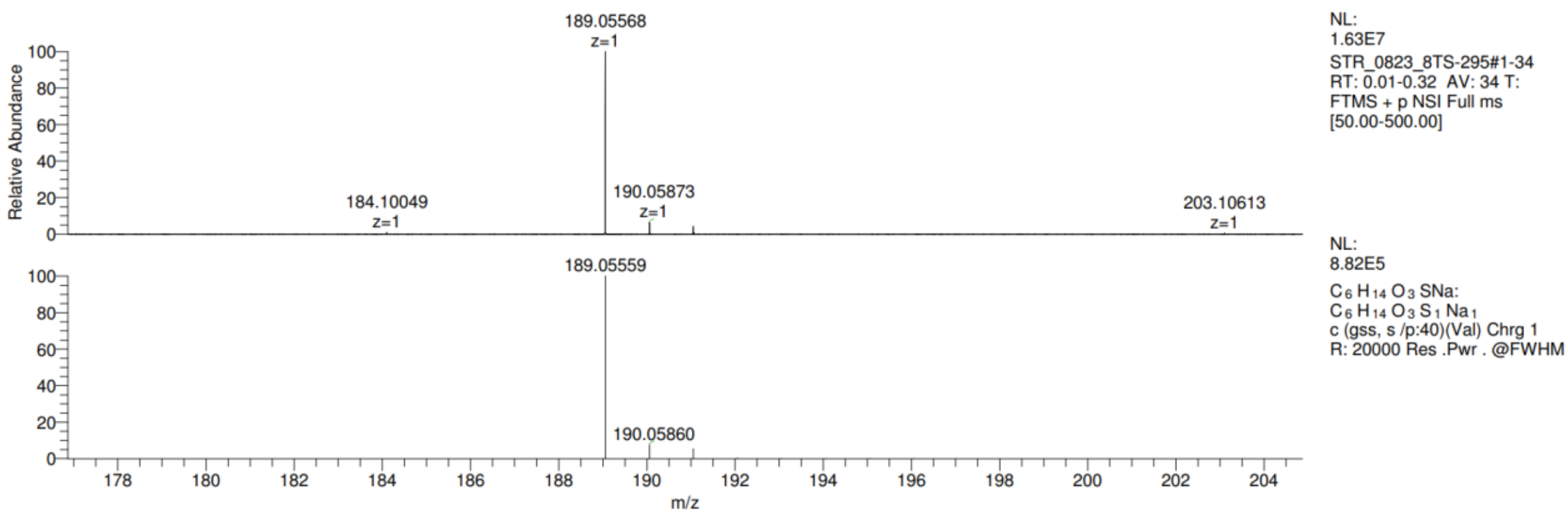

Figure S67: EM-MS-ESI spectrum of **11**.

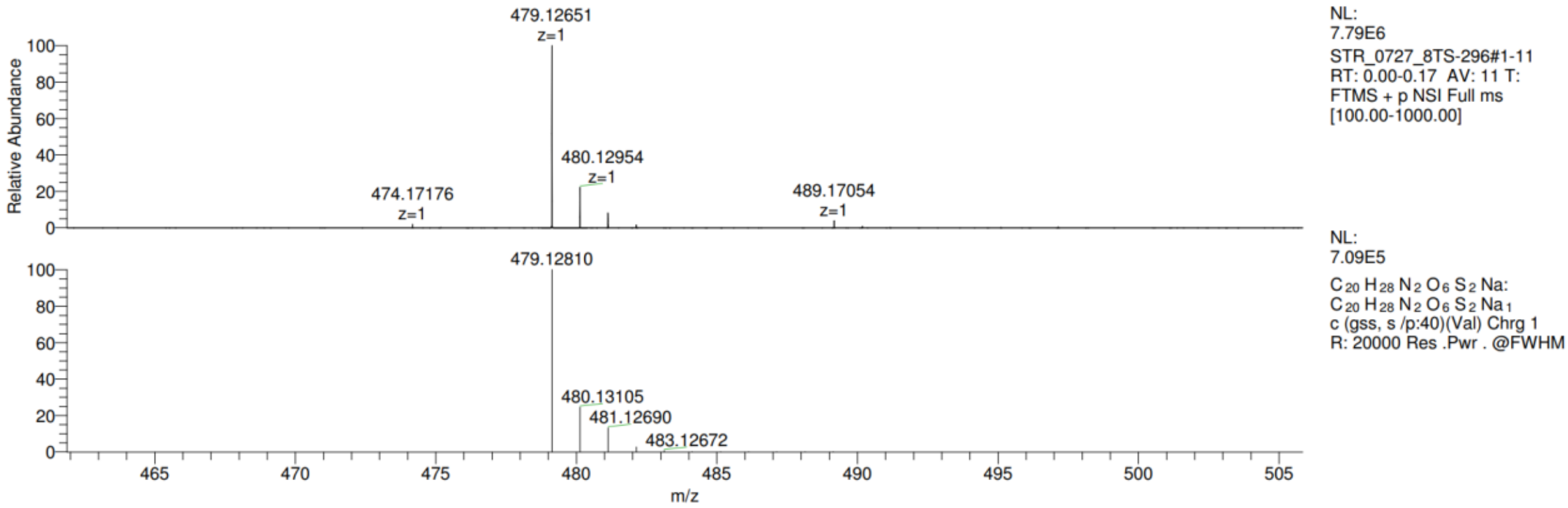

Figure S68: EM-MS-ESI spectrum of **12**.

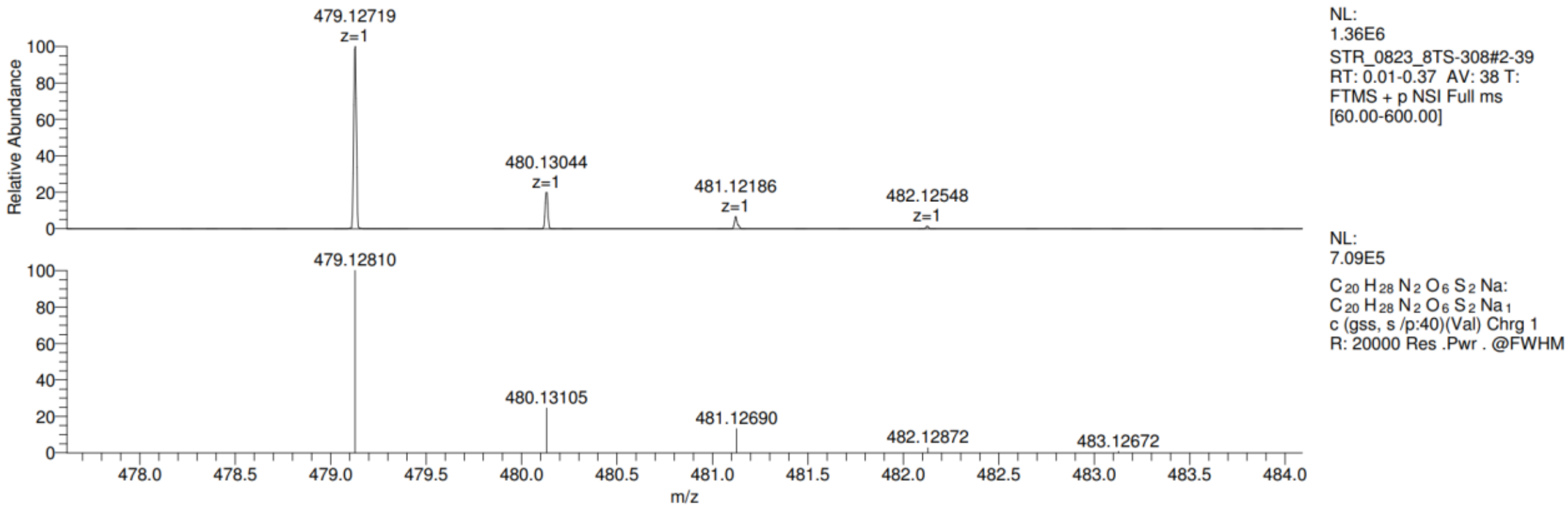

Figure S69: EM-MS-ESI spectrum of **14**.

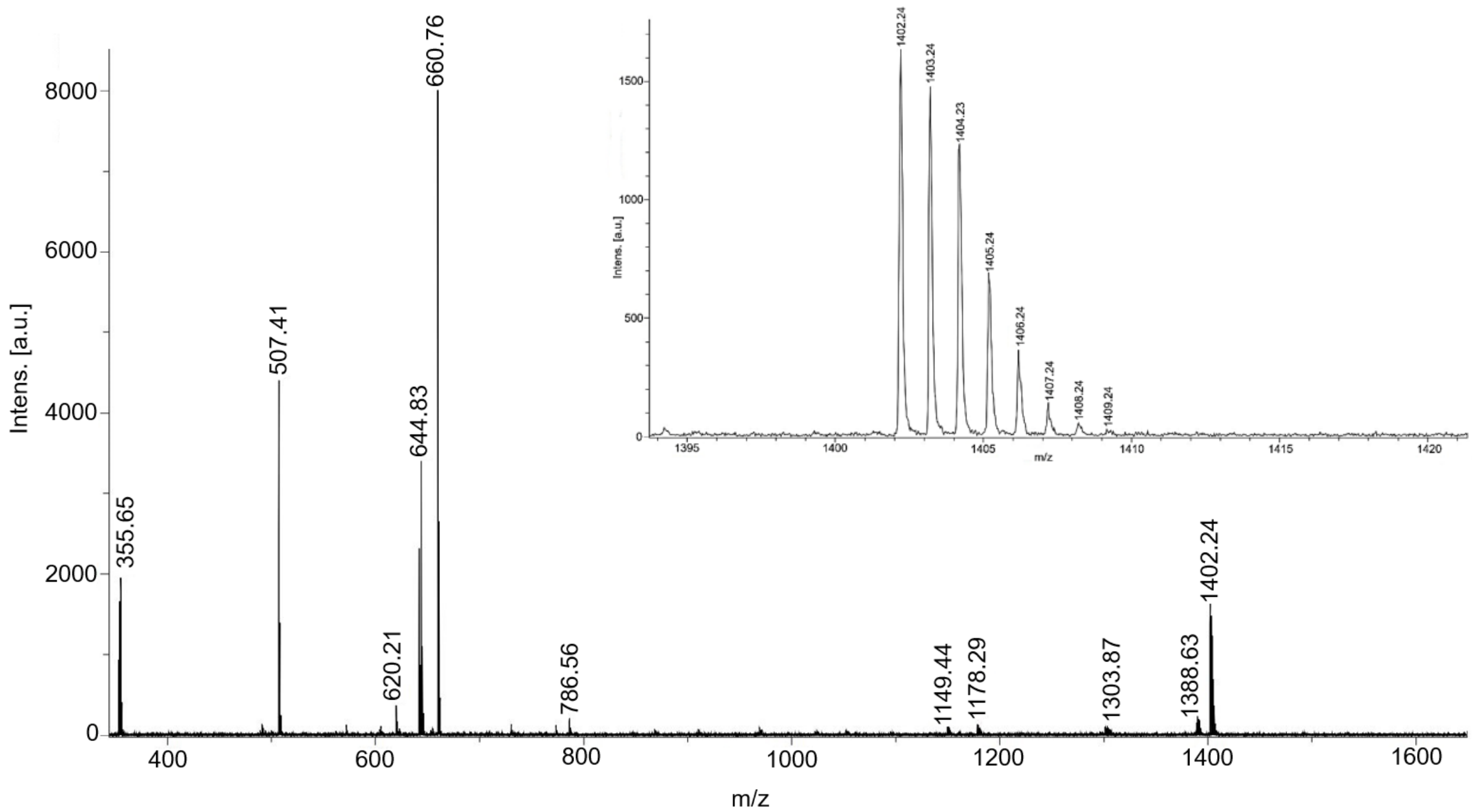

Figure S70: MALDI-TOF mass spectrum of **NiPc-1**, isotopic patterns for the molecular ion (inset).

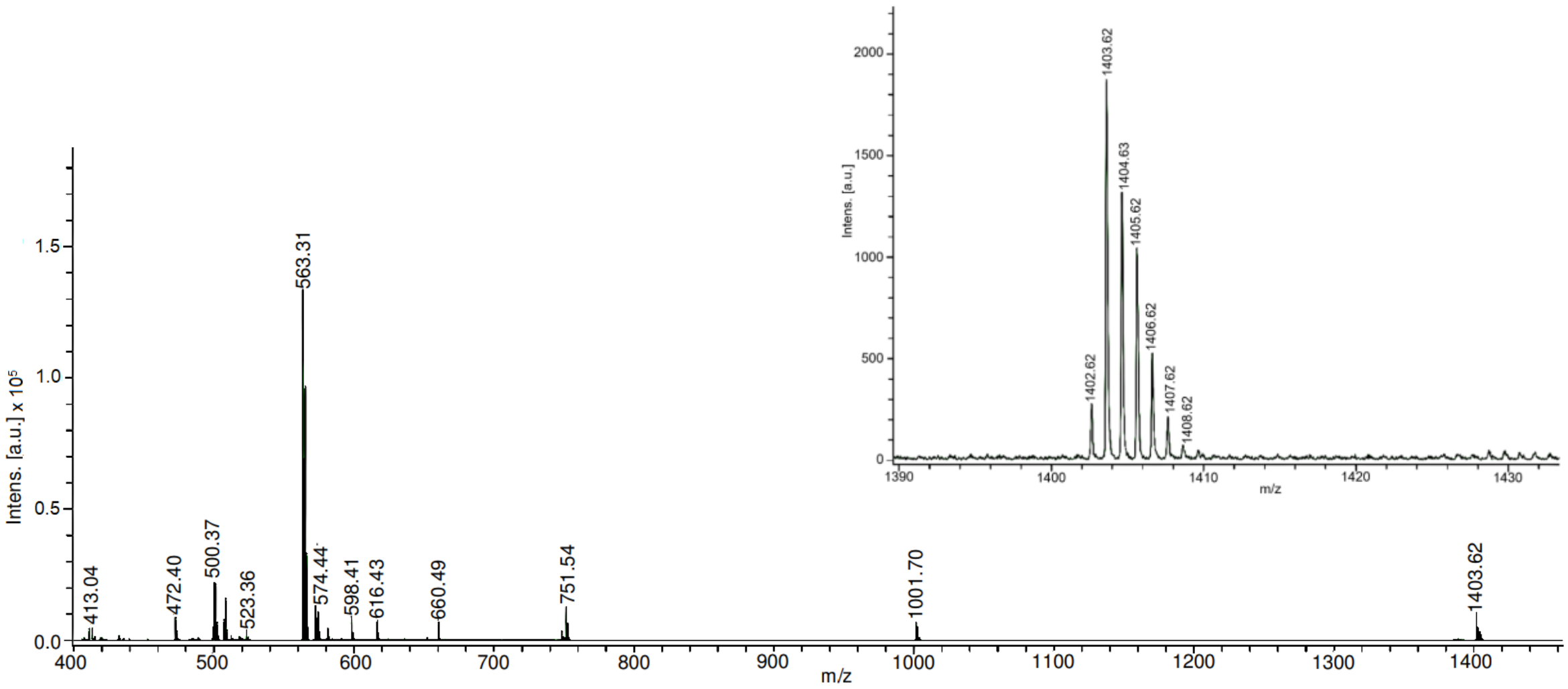

Figure S71: MALDI-TOF mass spectrum of **NiPc-2**, isotopic patterns for the molecular ion (inset).

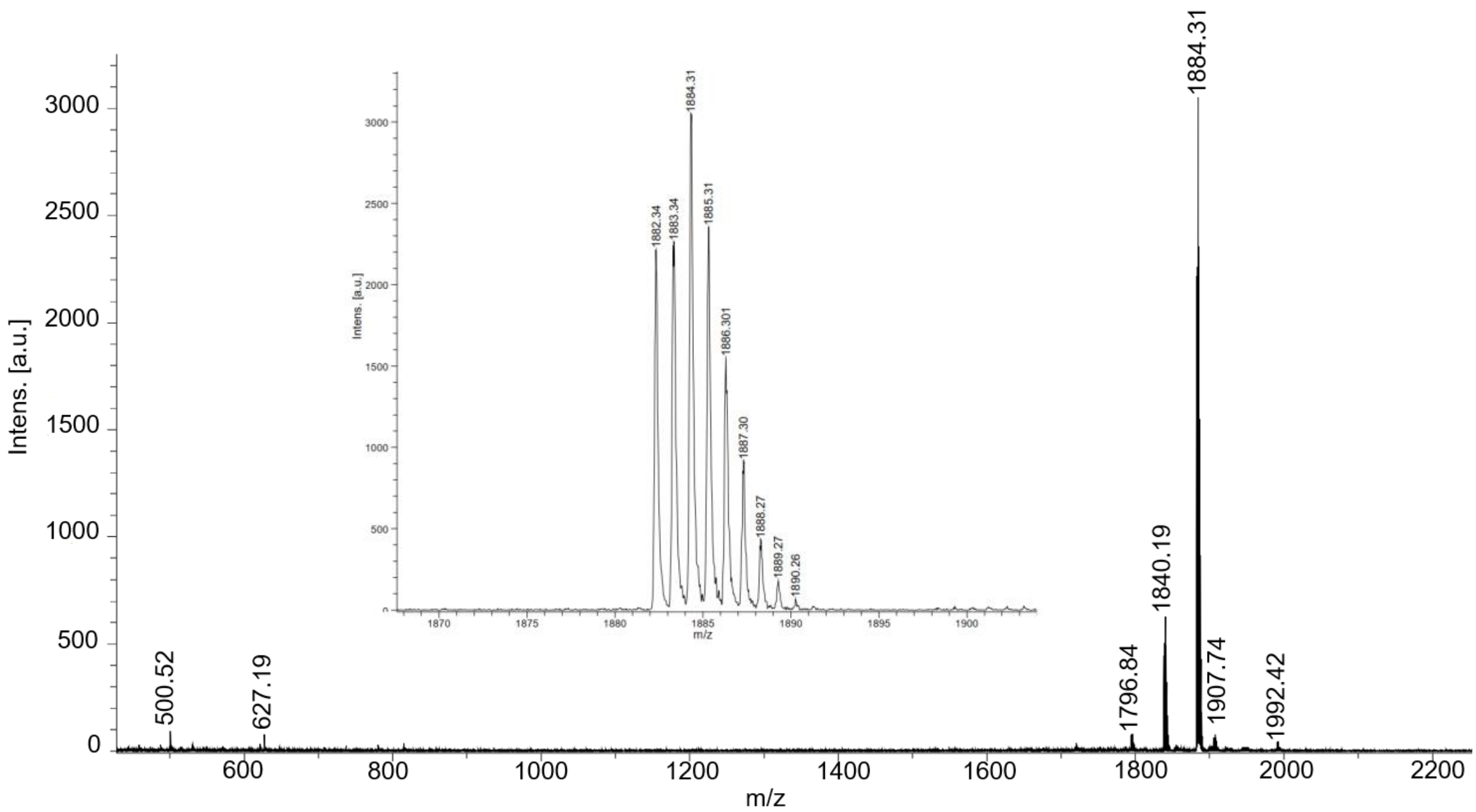

Figure S72: MALDI-TOF mass spectrum of **NiPc-3**, isotopic patterns for the molecular ion (inset).

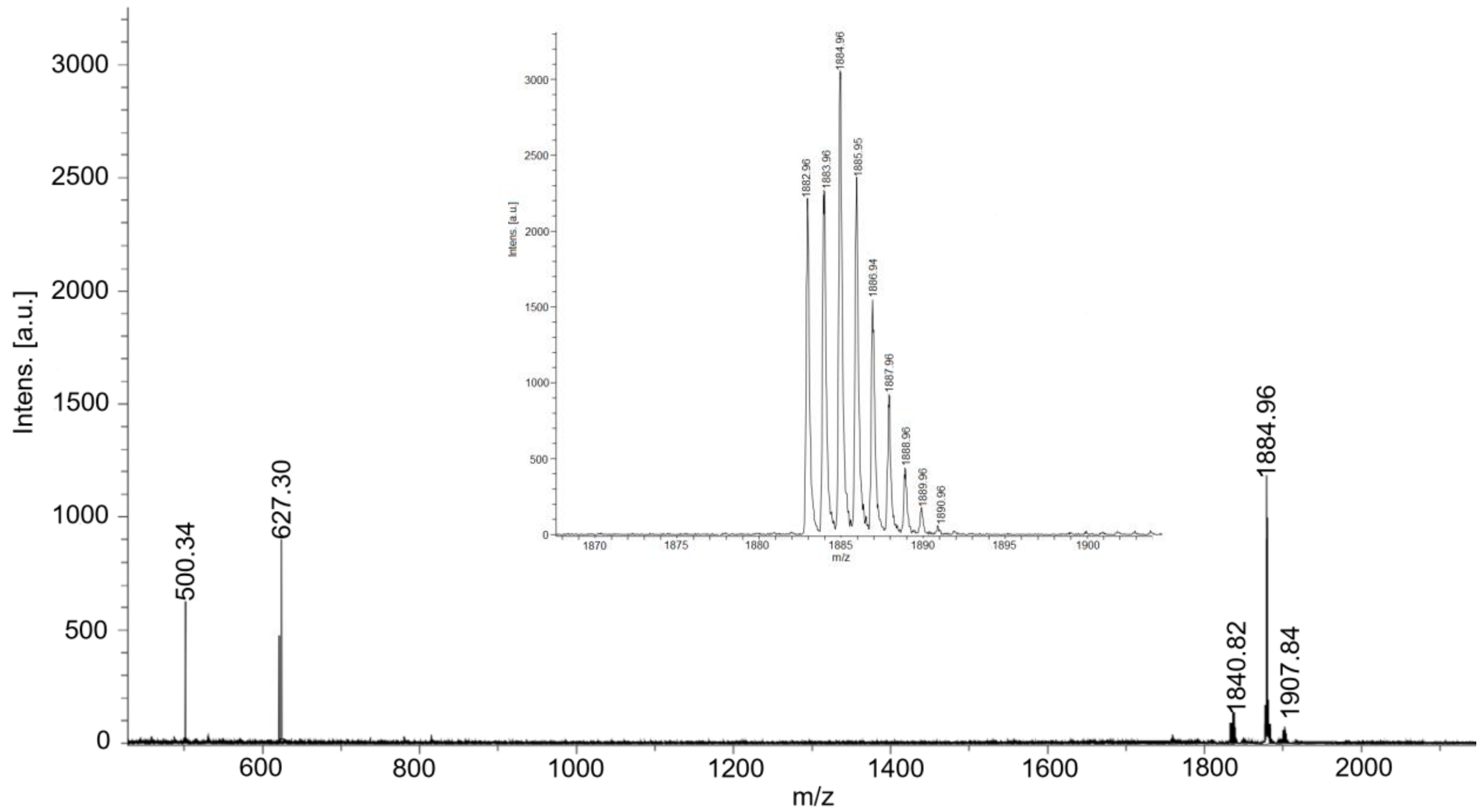

Figure S73: MALDI-TOF mass spectrum of **NiPc-4**, isotopic patterns for the molecular ion (inset).

Section S5: UV-vis absorption studies of NiPcs

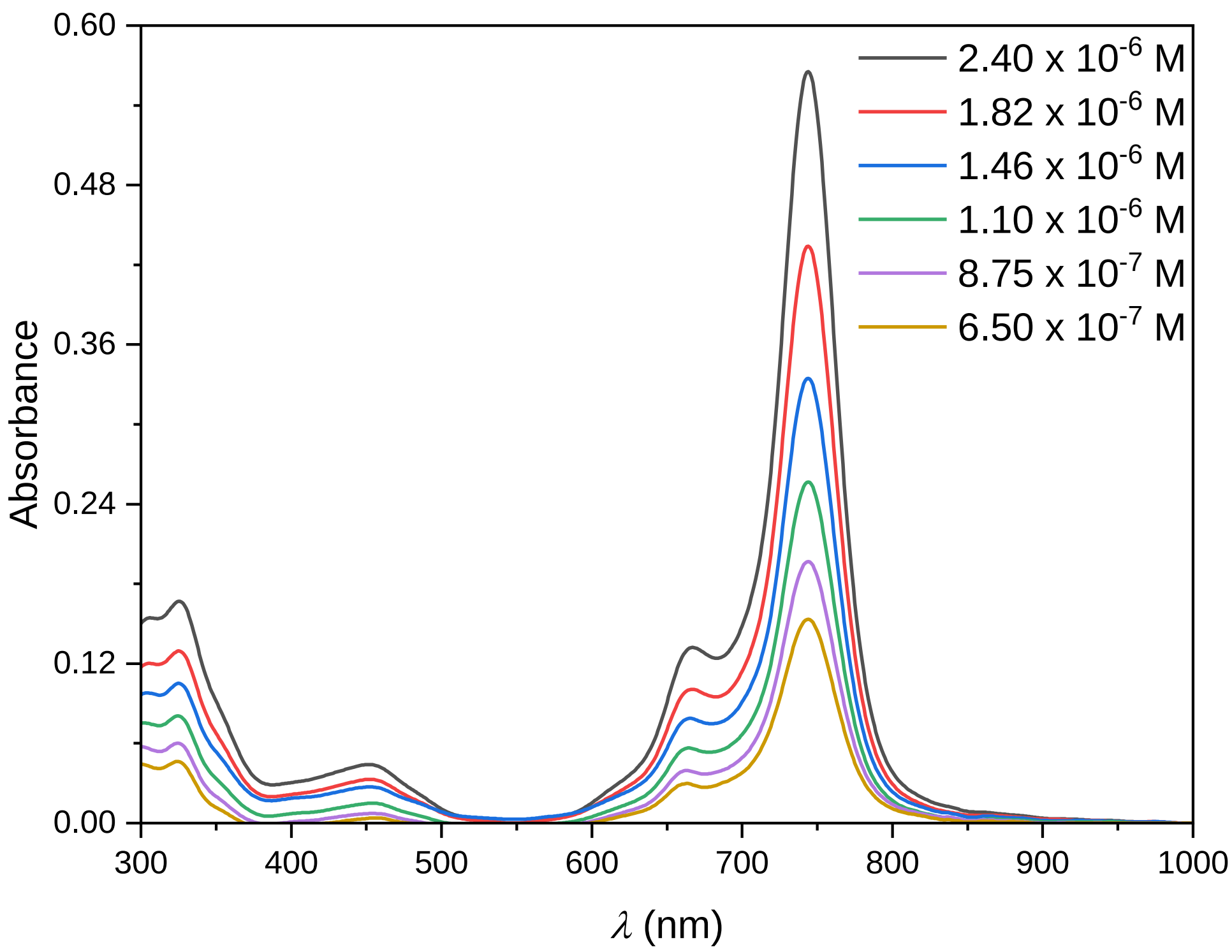

Figure S74: UV-vis absorption spectra of **NiPc-1** in DMSO at different molar concentrations.

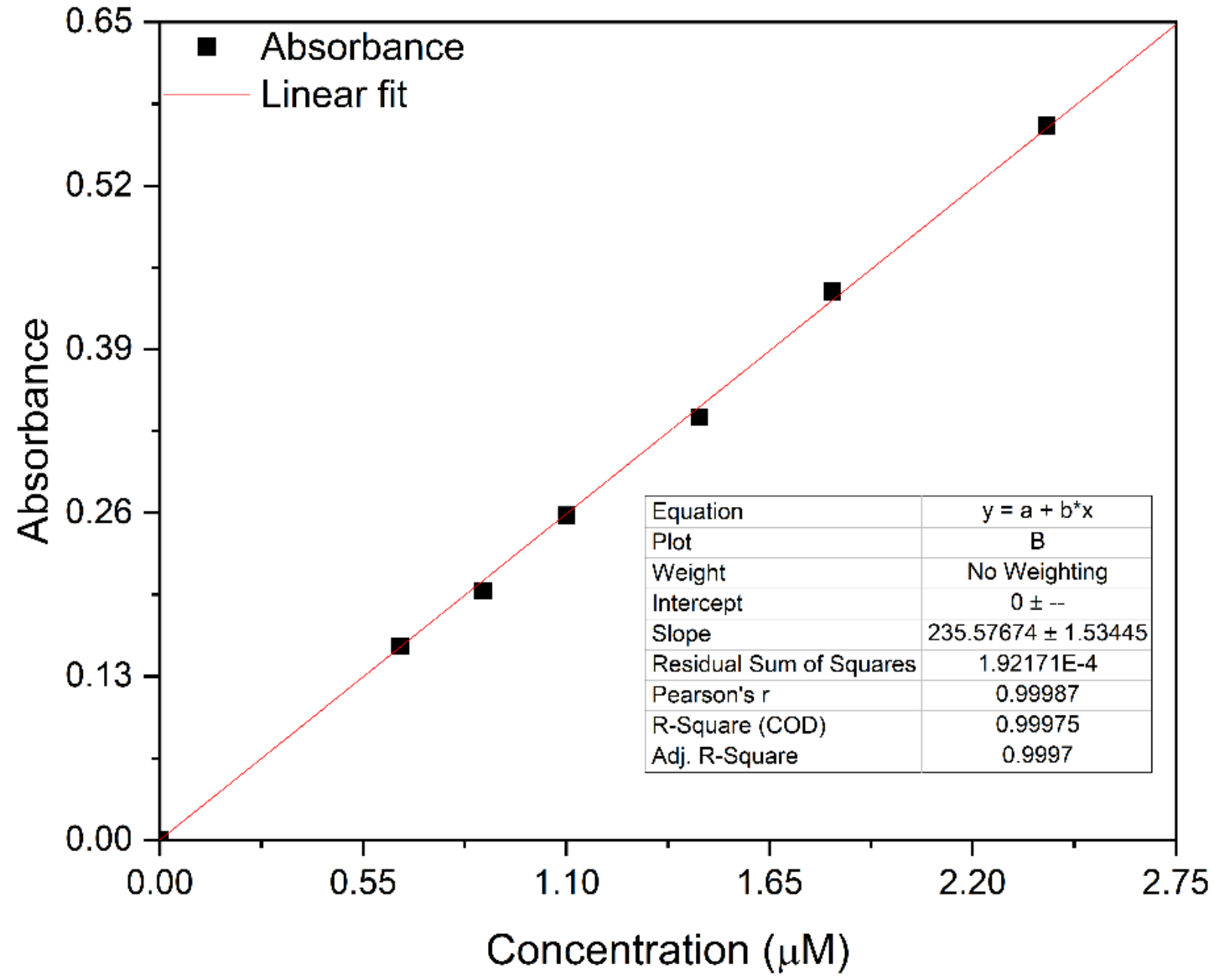

Figure S75: Lambert-Beer law verified for **NiPc-1** in DMSO at 298 K (λ_{max} = 746 nm).

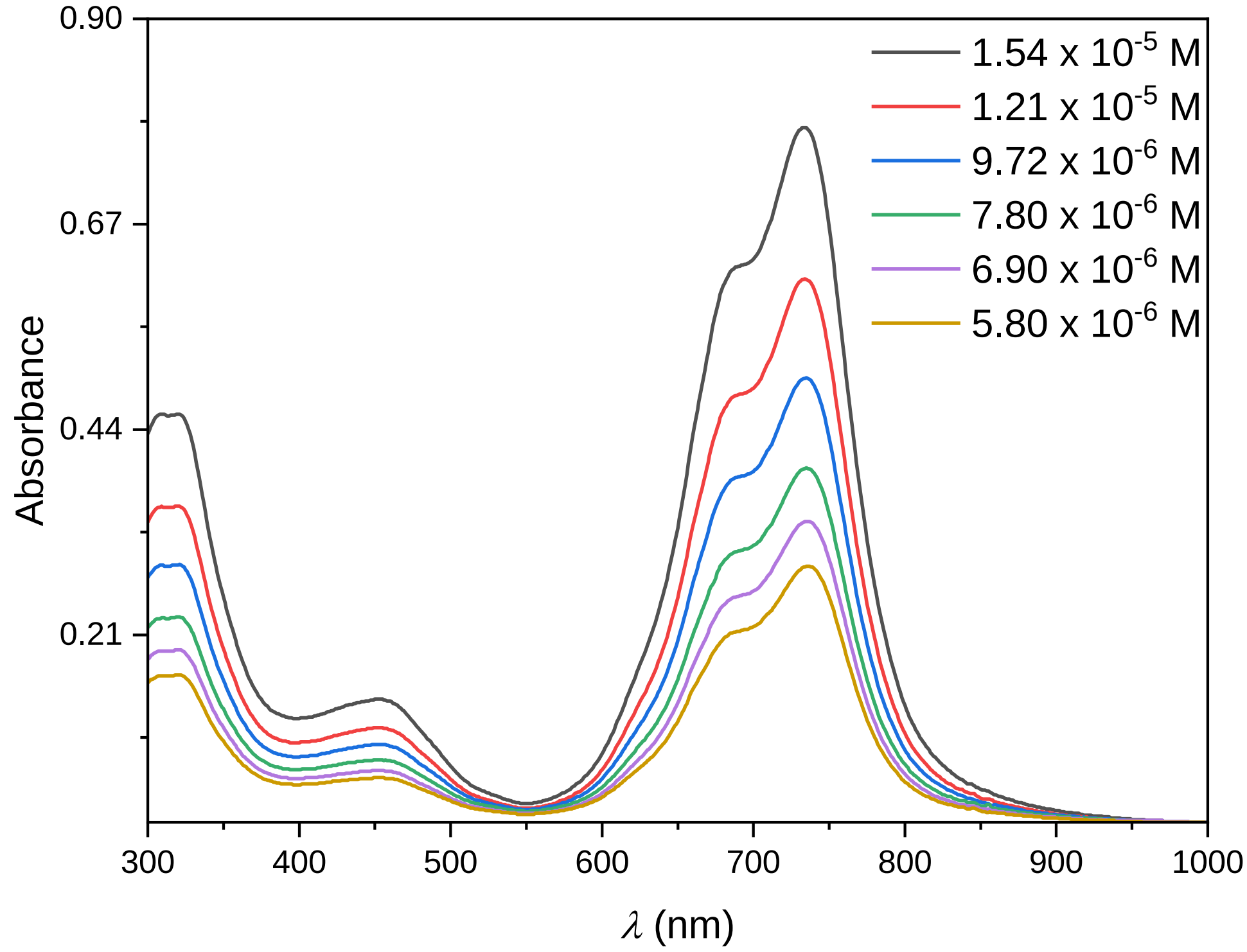

Figure S76: UV-vis absorption spectra of **NiPc-1** in H_2O at different molar concentrations.

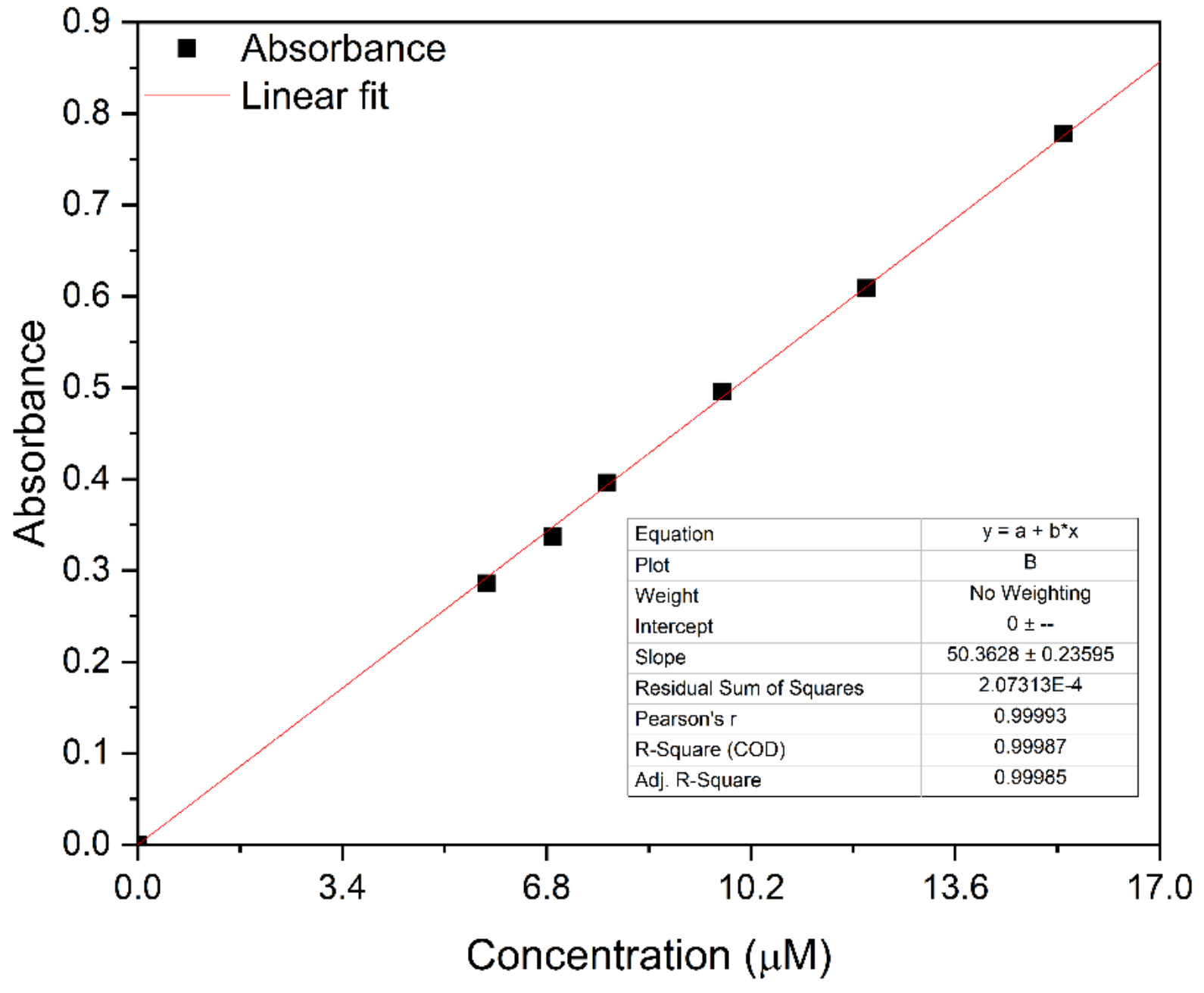

Figure S77: Lambert-Beer law verified for **NiPc-1** in H_2O at 298 K (λ_{max} = 734 nm).

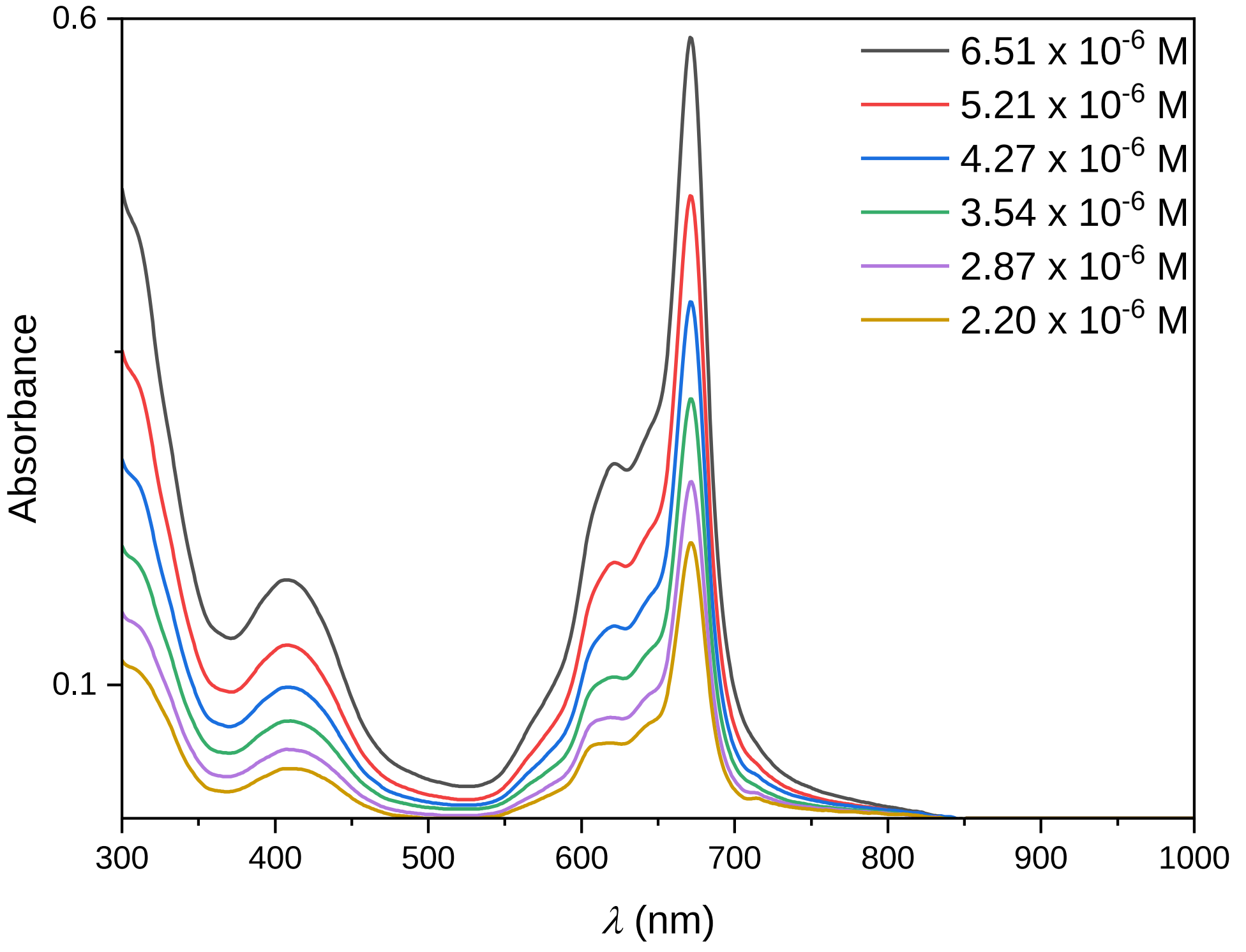

Figure S78: UV-vis absorption spectra of **NiPc-2** in DMSO at different molar concentrations.

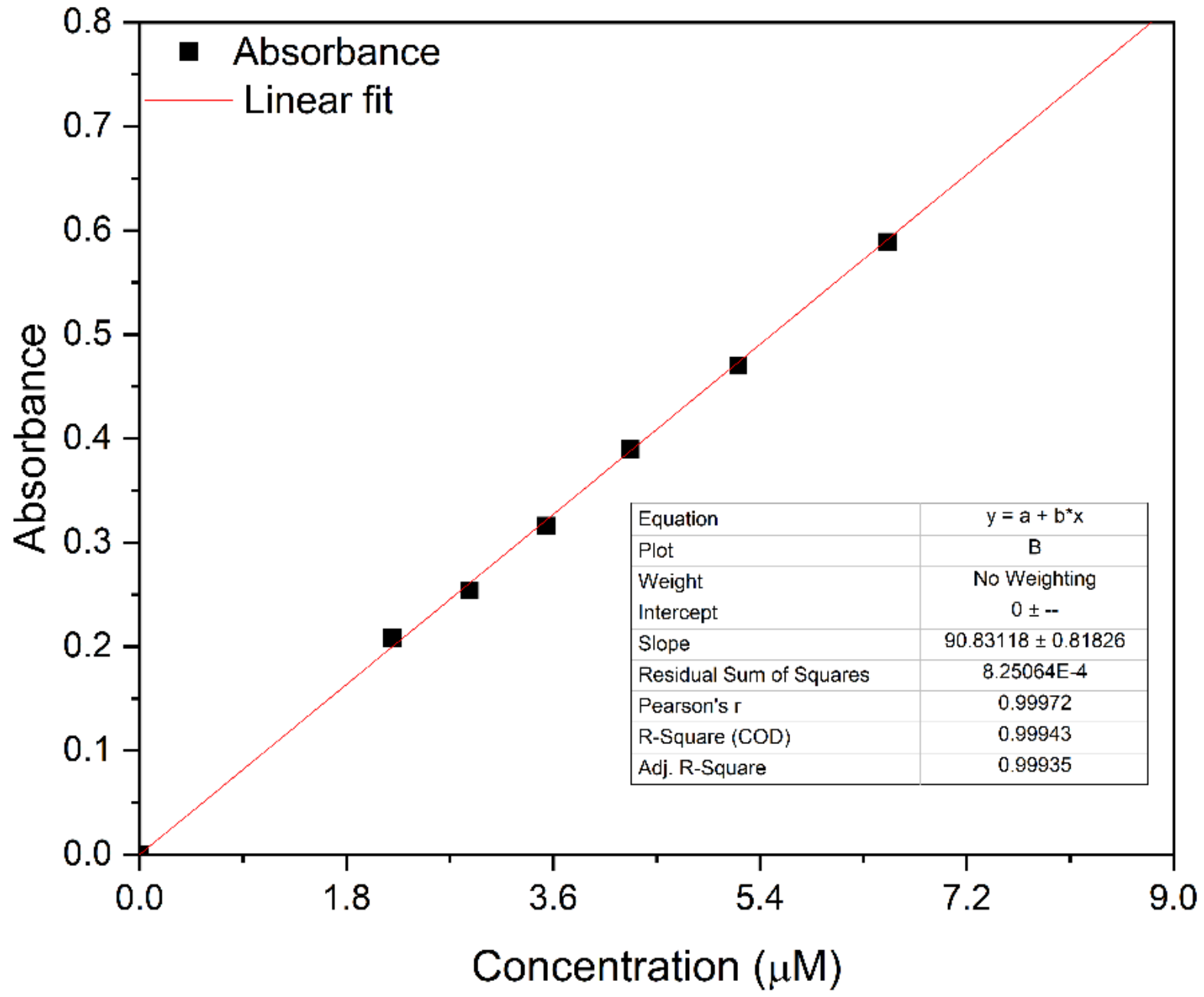

Figure S79: Lambert-Beer law verified for **NiPc-2** in DMSO at 298 K (λ_{max} = 670 nm).

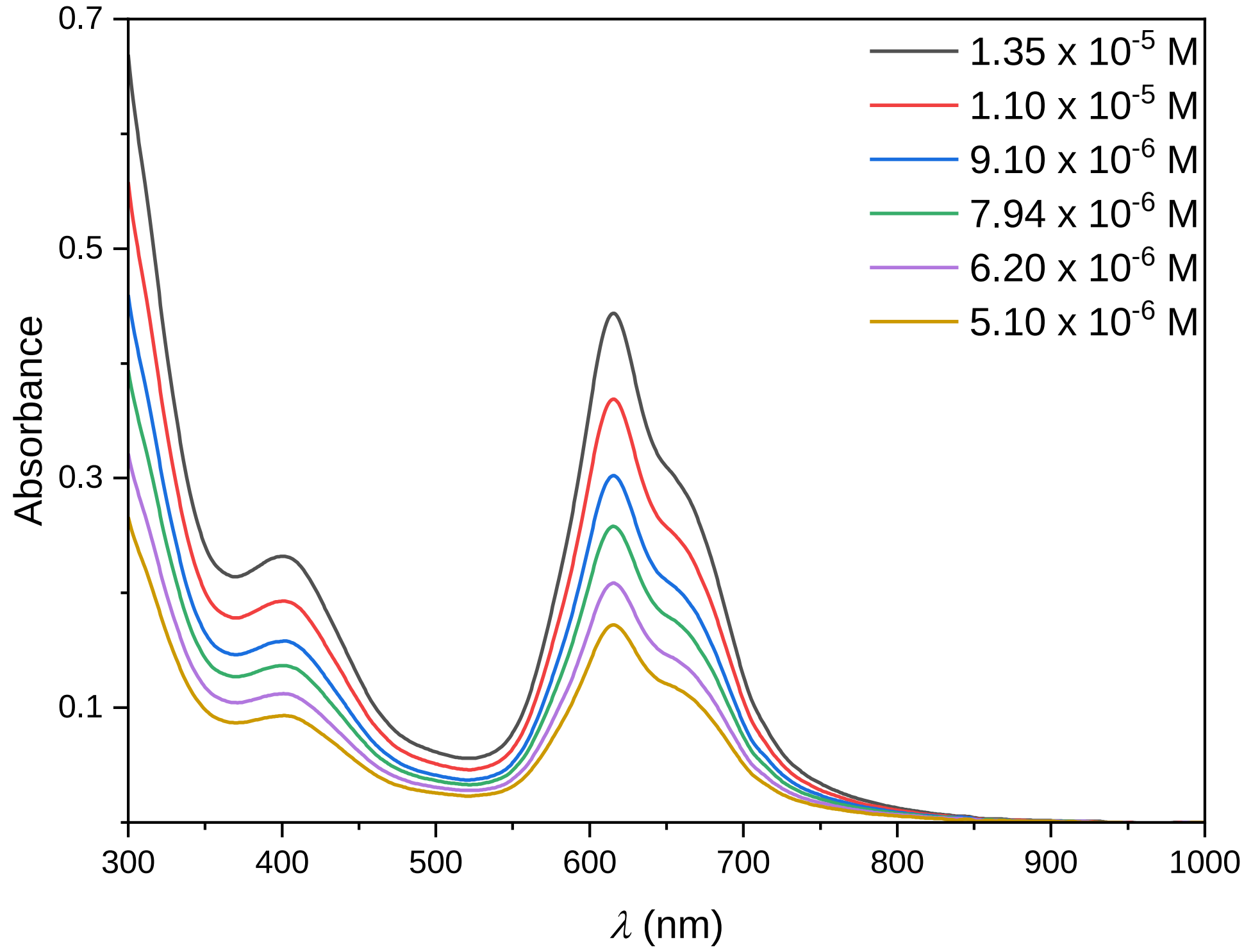

Figure S80: UV-vis absorption spectra of **NiPc-2** in H_2O at different molar concentrations.

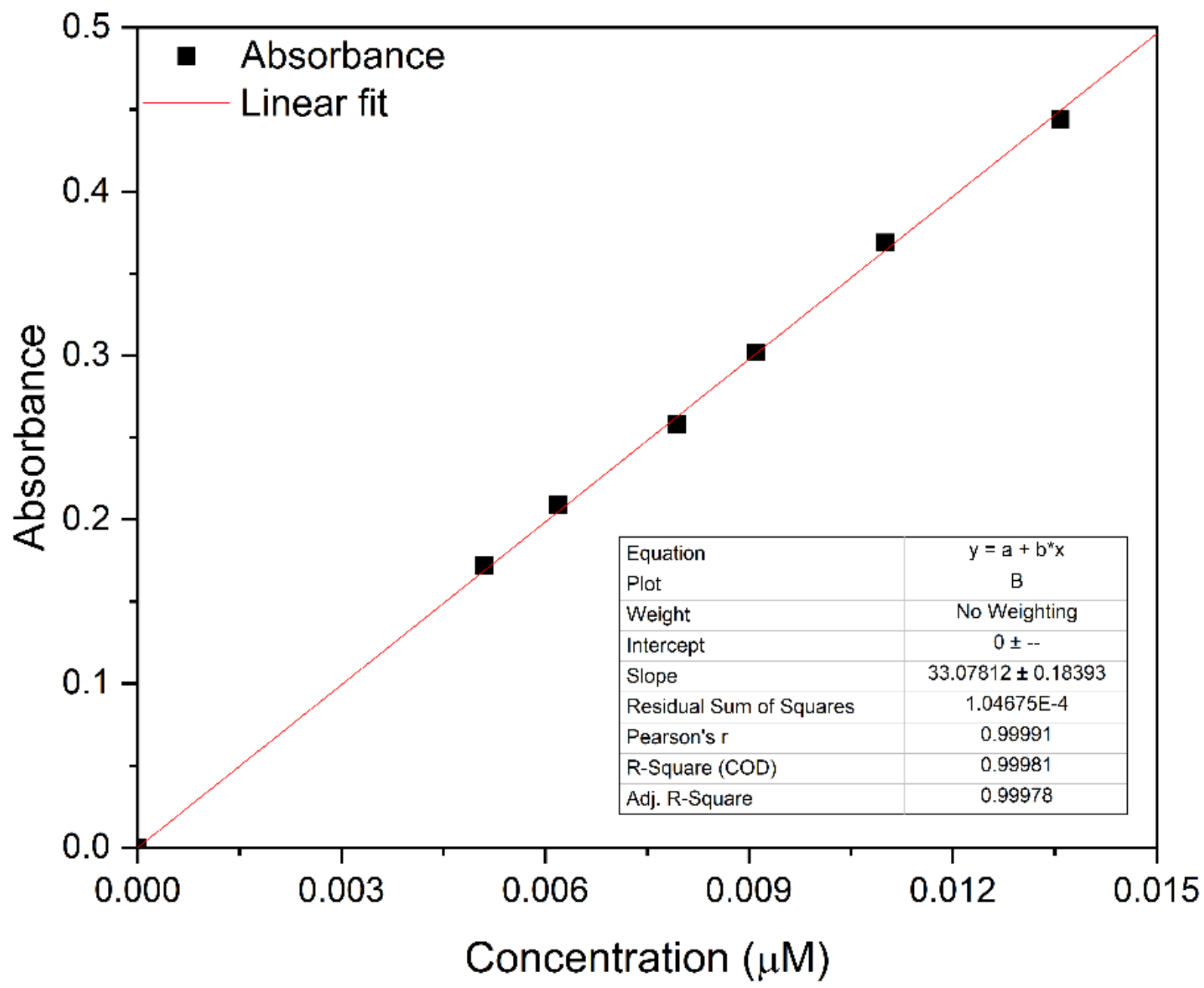

Figure S81: Lambert-Beer law verified for **NiPc-2** in H_2O at 298 K (λ_{max} = 616 nm).

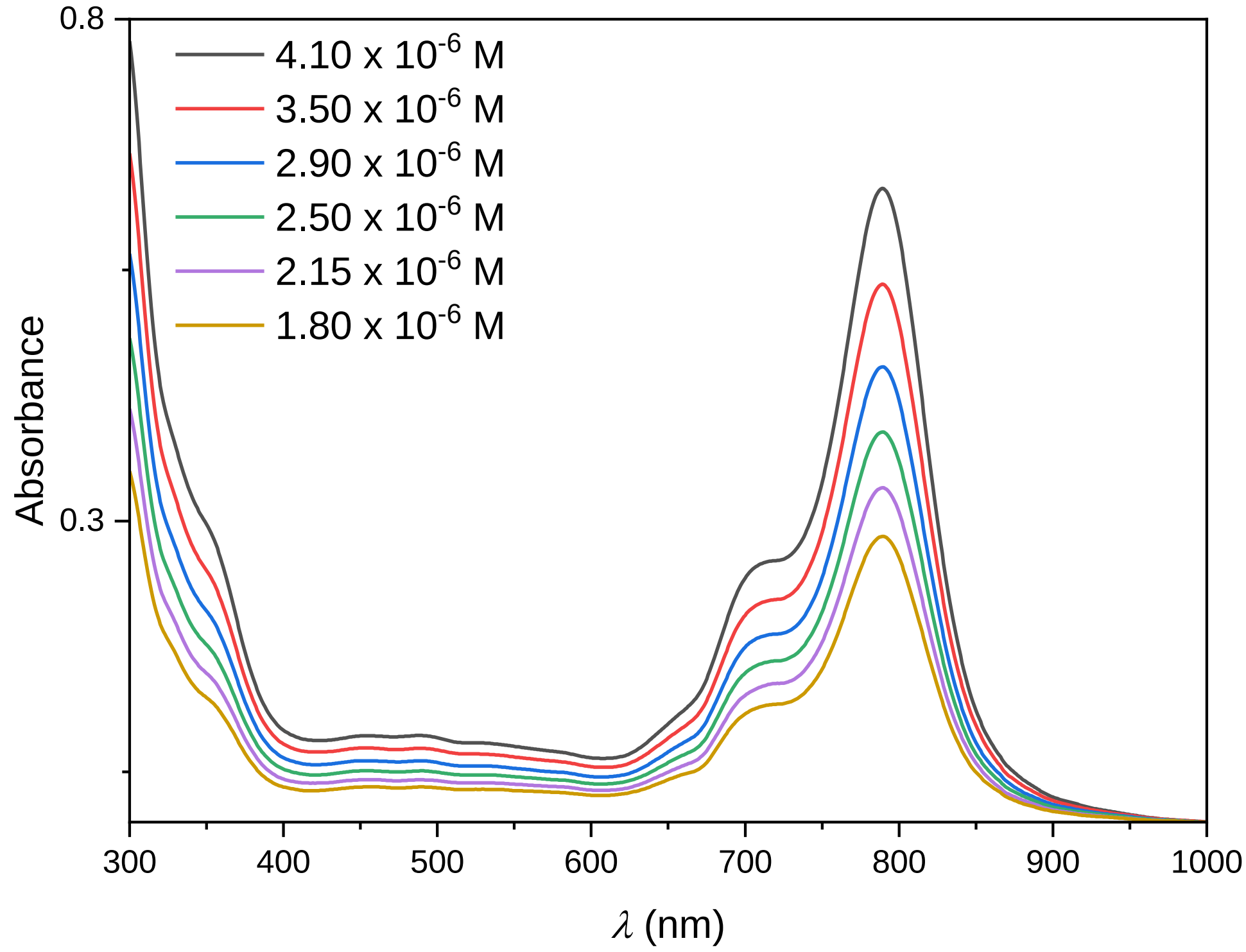

Figure S82: UV-vis absorption spectra of **NiPc-3** in DMSO at different molar concentrations.

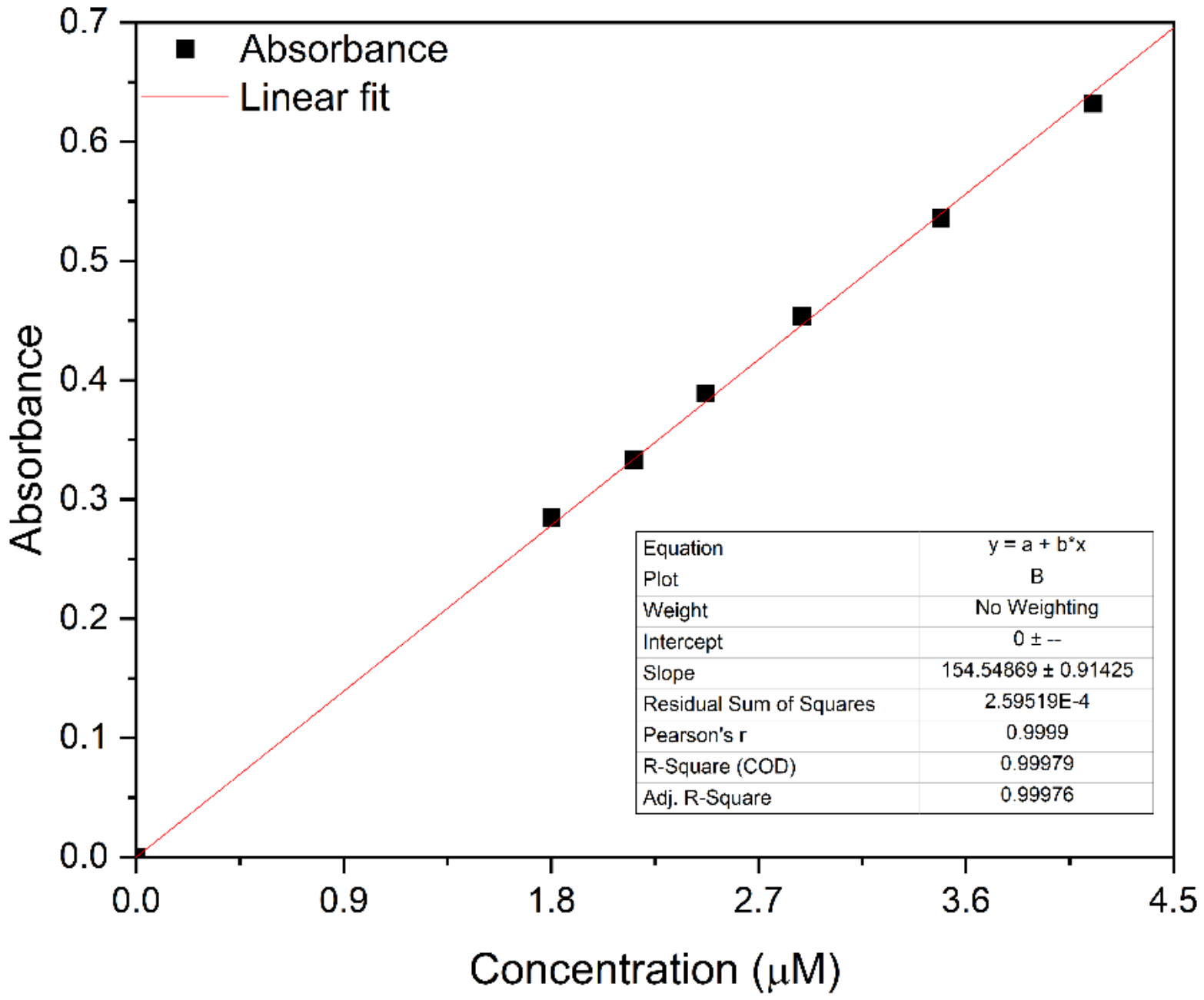

Figure S83: Lambert-Beer law verified for **NiPc-3** in DMSO at 298 K (λ_{max} = 790 nm).

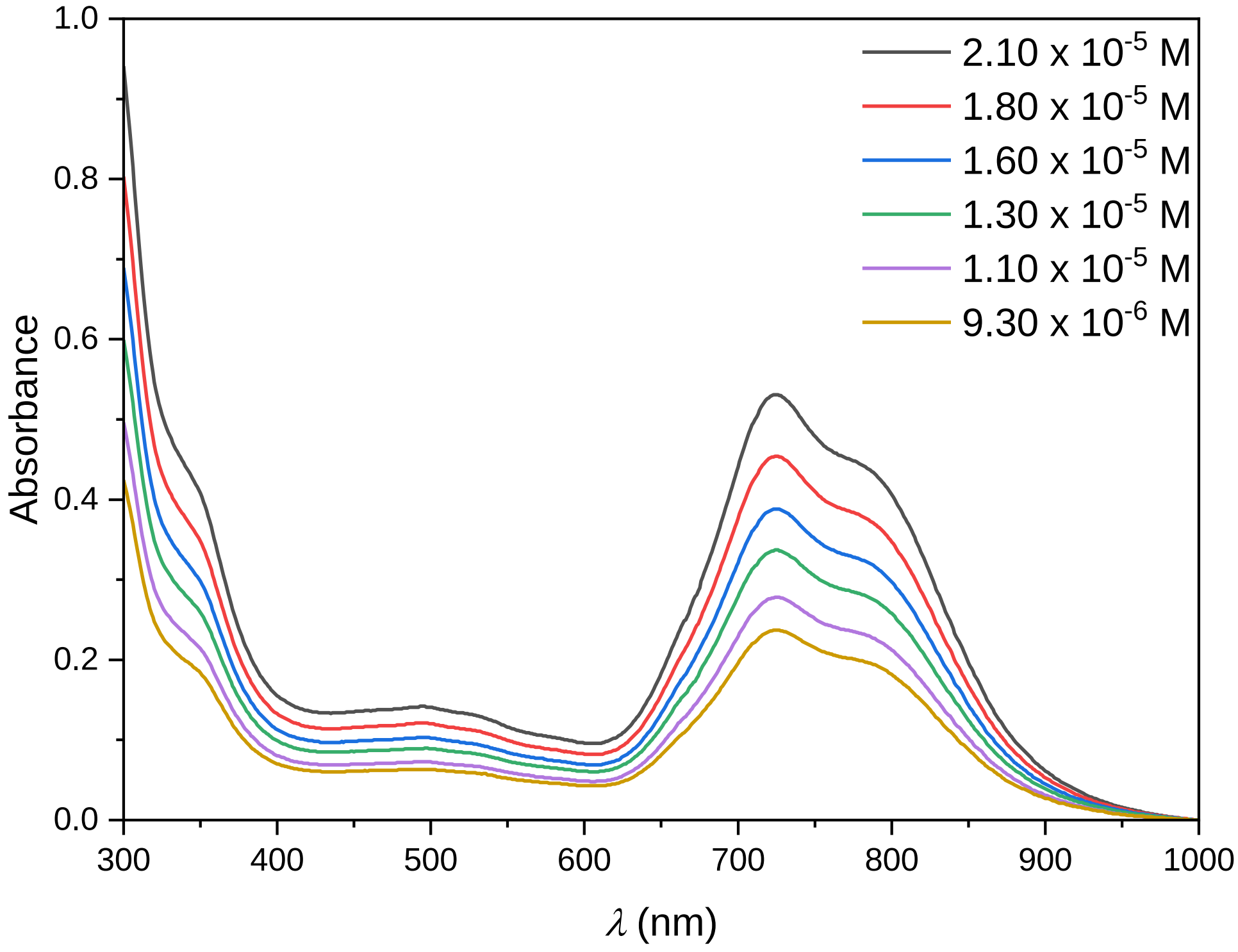

Figure S84: UV-vis absorption spectra of **NiPc-3** in H_2O at different molar concentrations.

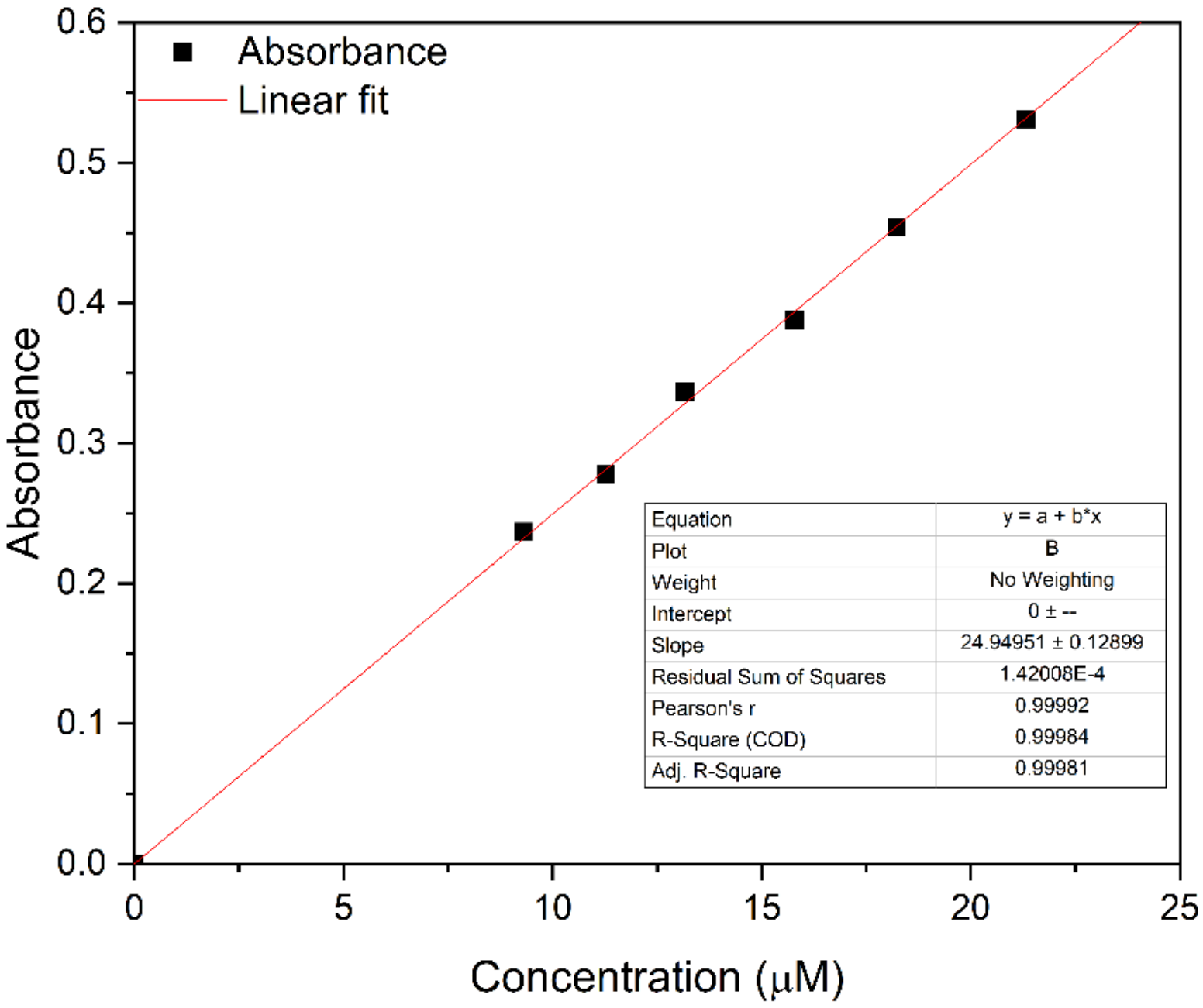

Figure S85: Lambert-Beer law verified for **NiPc-3** in H_2O at 298 K (λ_{max} = 726 nm).

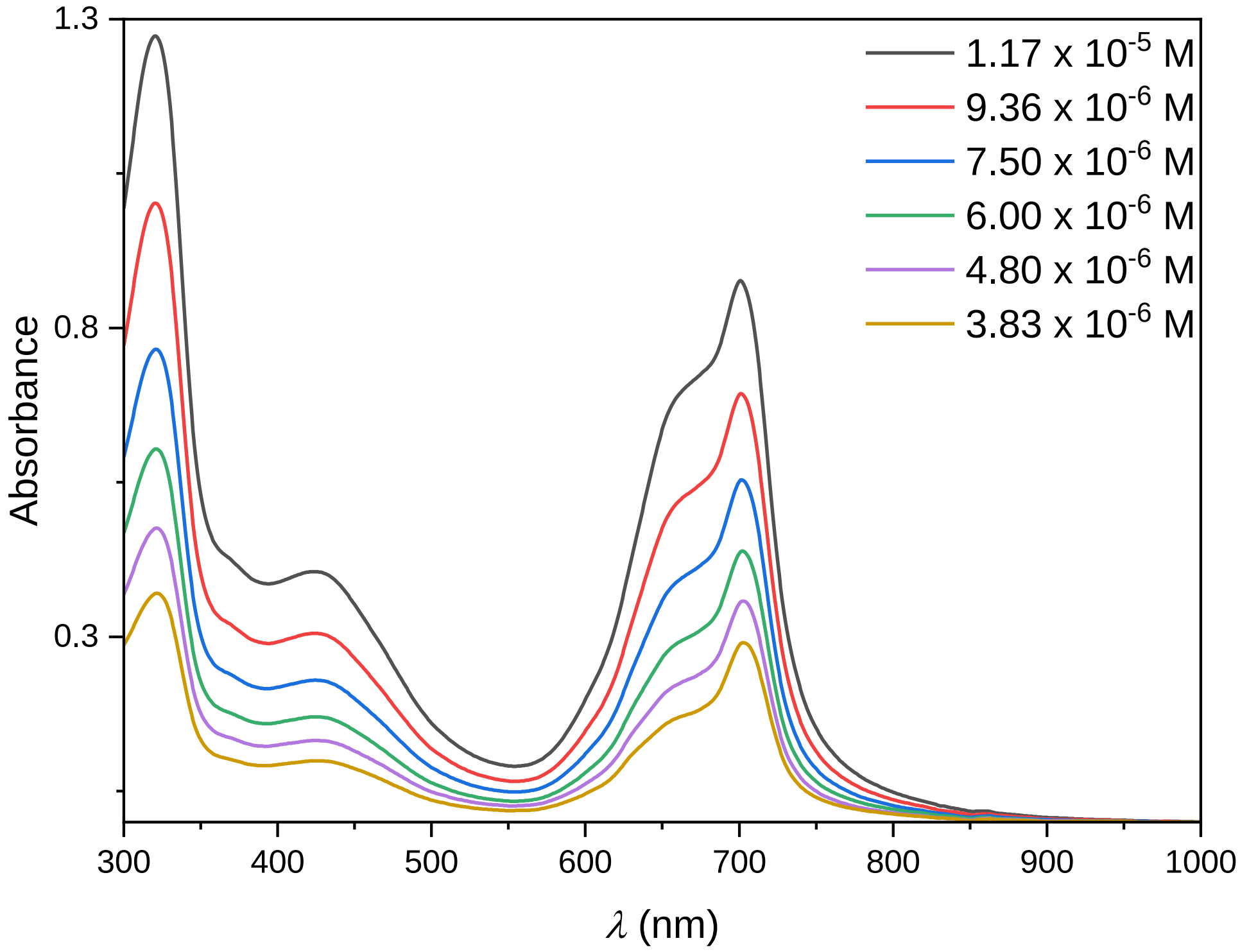

Figure S86: UV-vis absorption spectra of **NiPc-4** in DMSO at different molar concentrations.

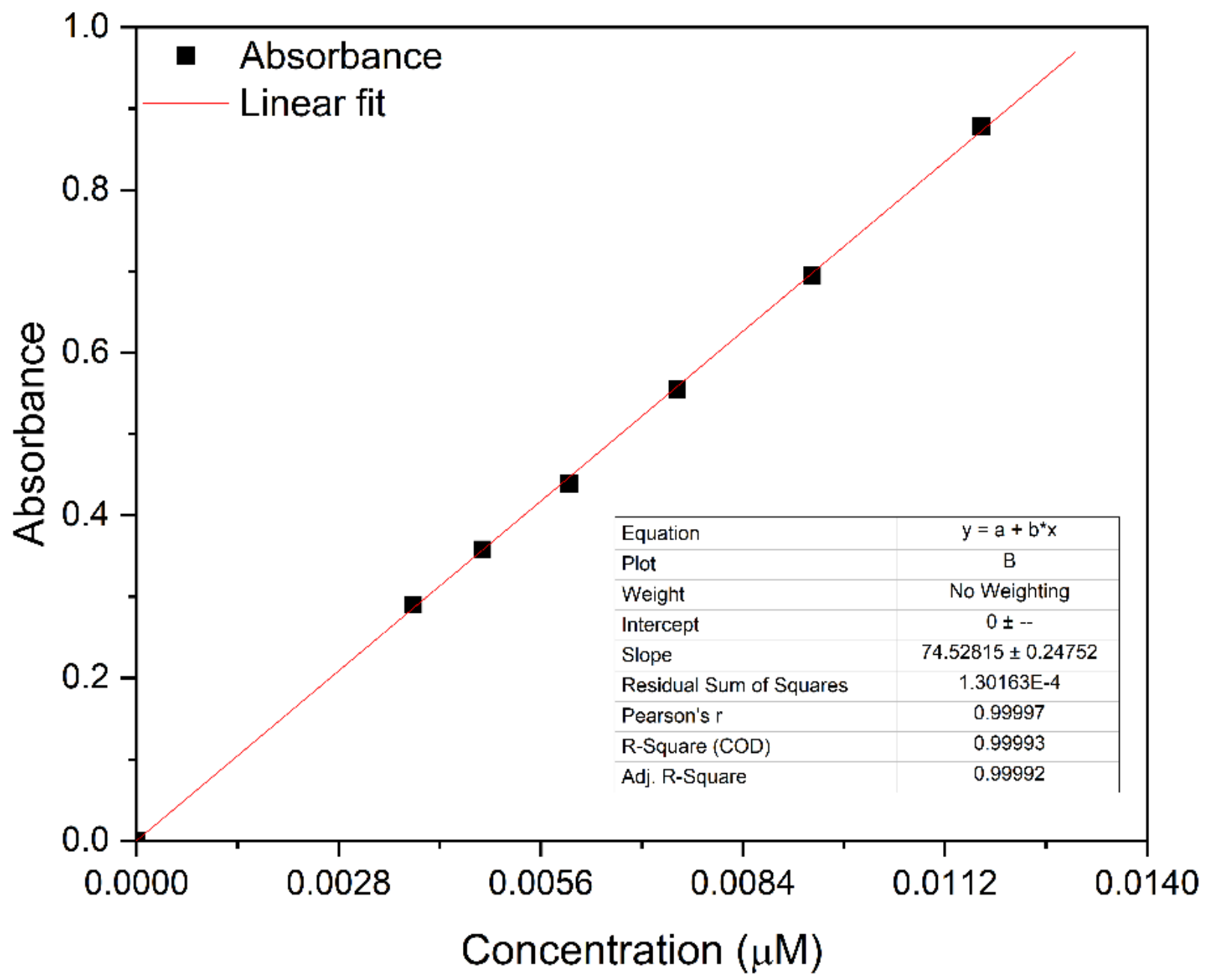

Figure S87: Lambert-Beer law verified for **NiPc-4** in DMSO at 298 K (λ_{max} = 703 nm).

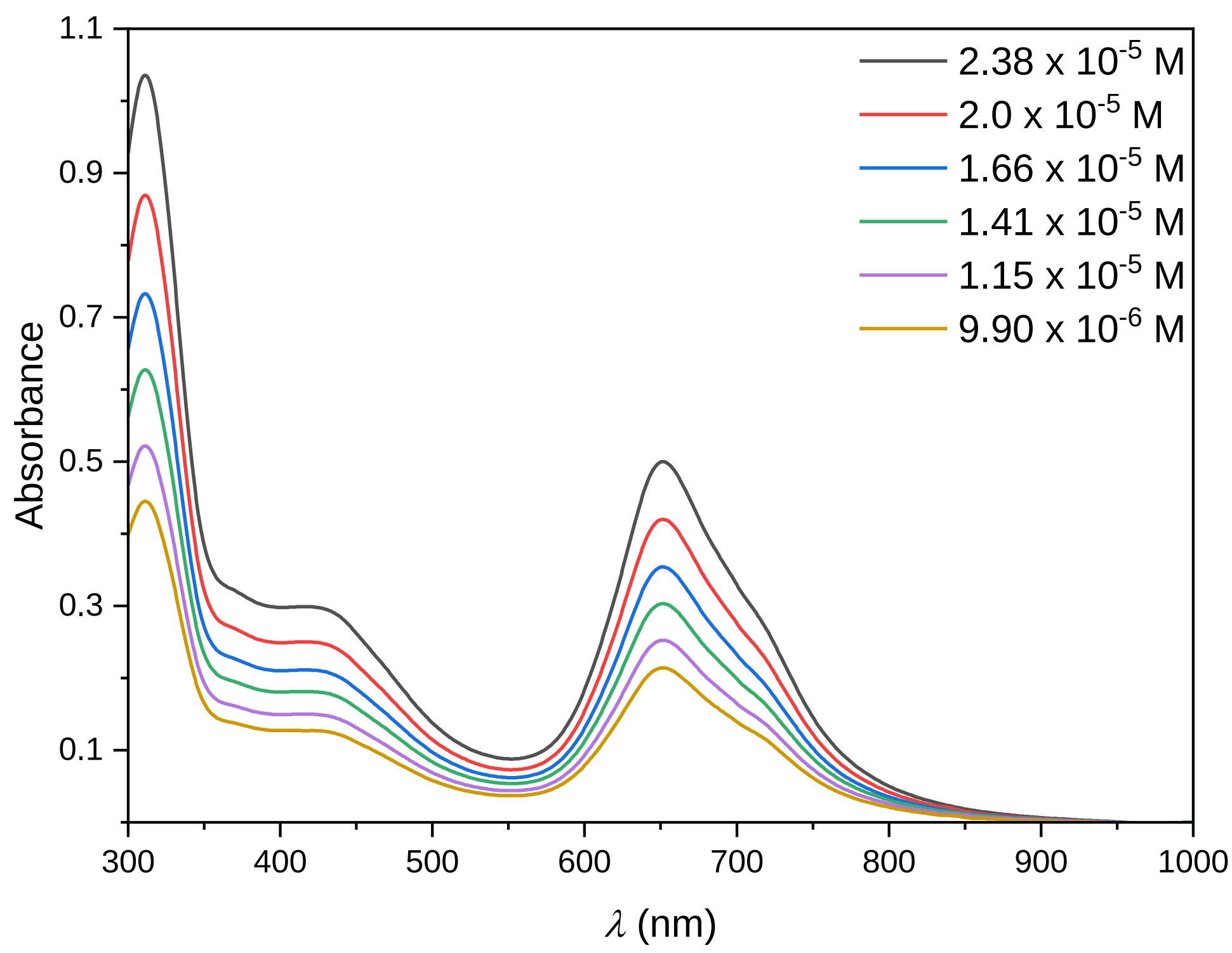

Figure S88: UV-vis absorption spectra of **NiPc-4** in H_2O at different molar concentrations:

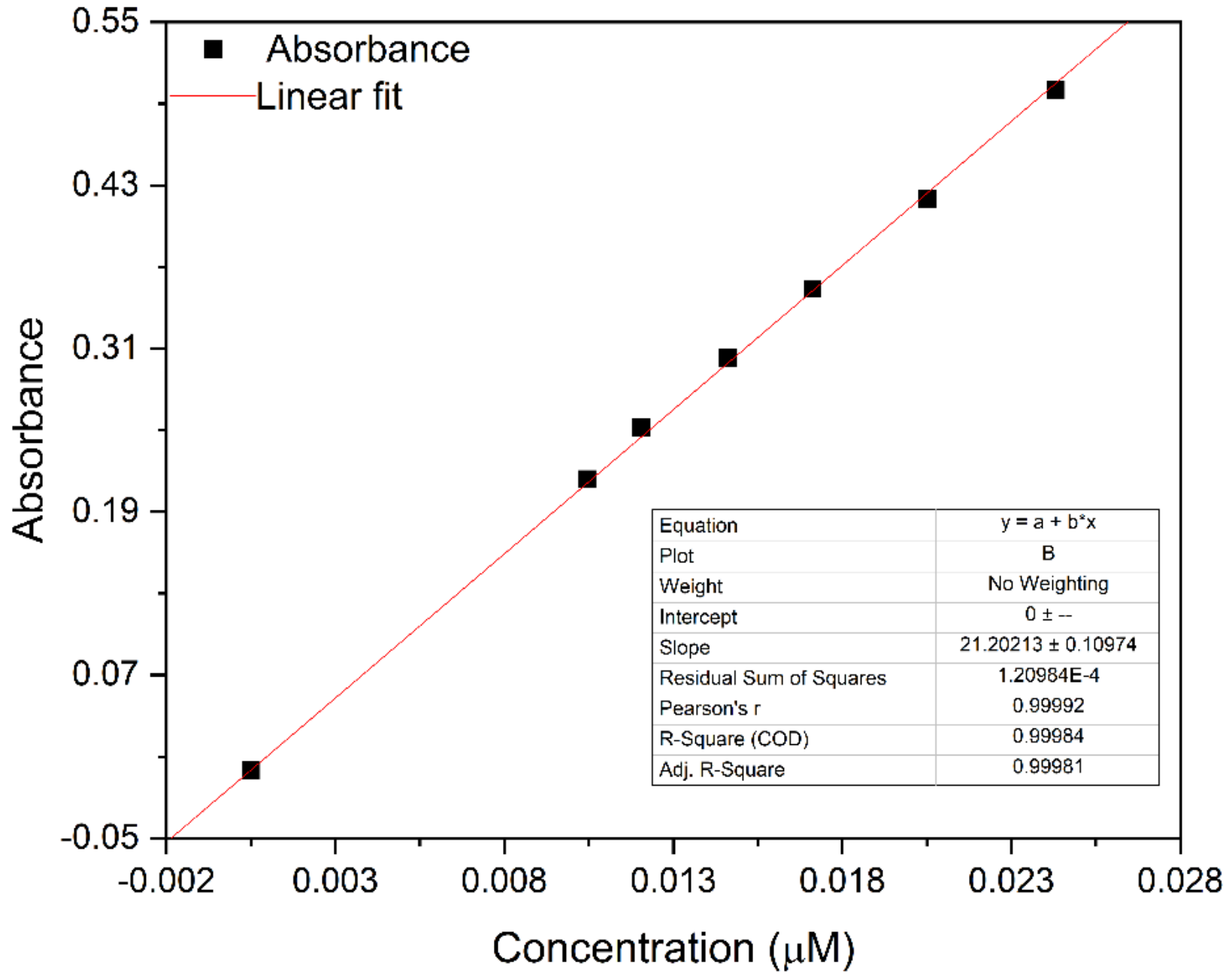

Figure S89: Lambert-Beer law verified for **NiPc-4** in H_2O at 298 K (λ_{max} = 650 nm).

Table S1 Q-bands of the compounds observed in the absorption spectra. The molar absorption coefficient (ε) for each band is also listed.

Compound	Q-band in DMSO		Q-band in H_2O	
	λ_{max} / nm	ε /10^4 M^{-1} cm^{-1}	λ_{max} / nm	ε /10^4 M^{-1} cm^{-1}
**NiPc-1**	746	23	734	5.0
**NiPc-2**	670	9.0	616	3.3
**NiPc-3**	790	15	726	2.5
**NiPc-4**	703	7.4	650	2.1

Section S6: Experimental setup of TAS and PAS

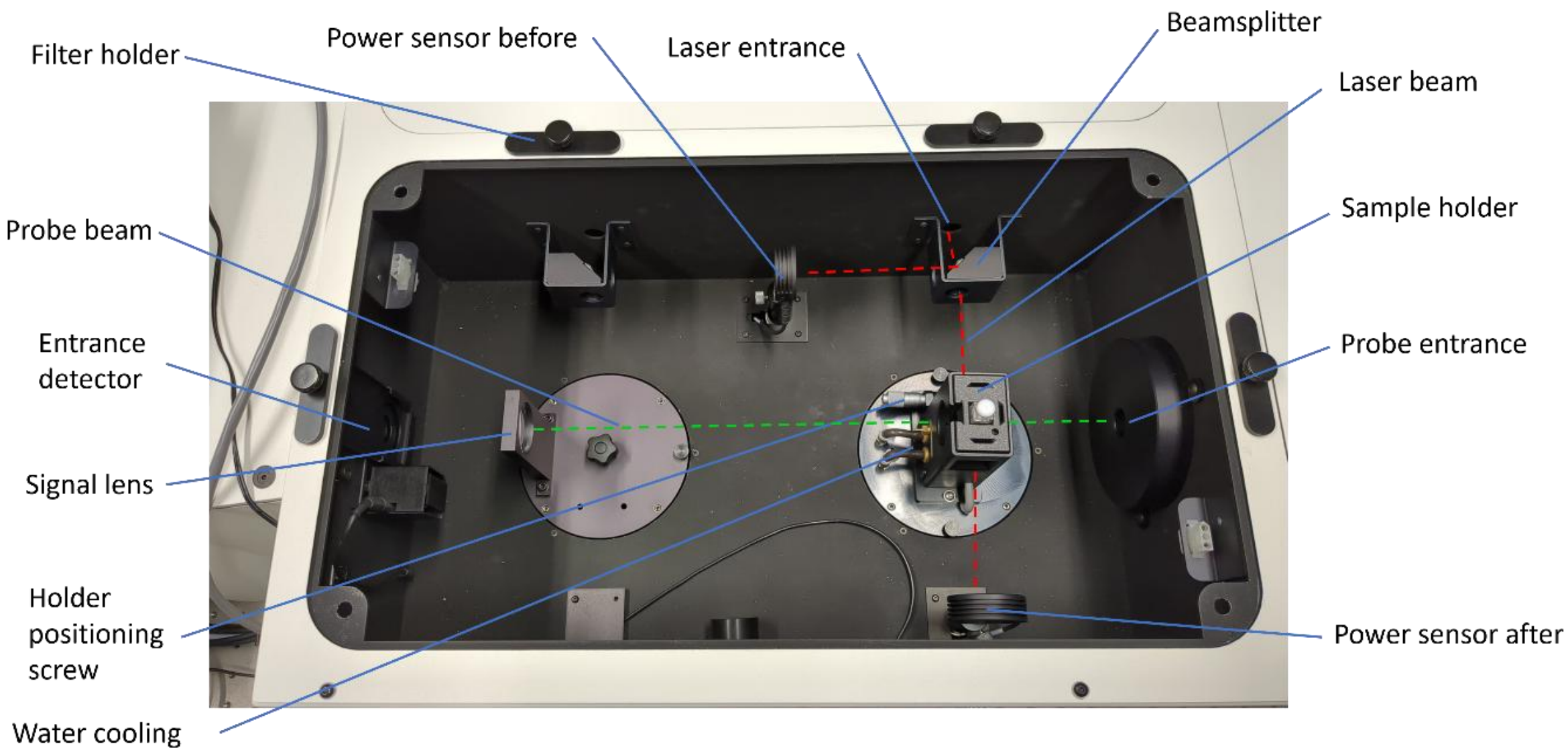

Figure S90: Sample chamber of the LP980 after the implementation of combined TAS and PAS measurements.

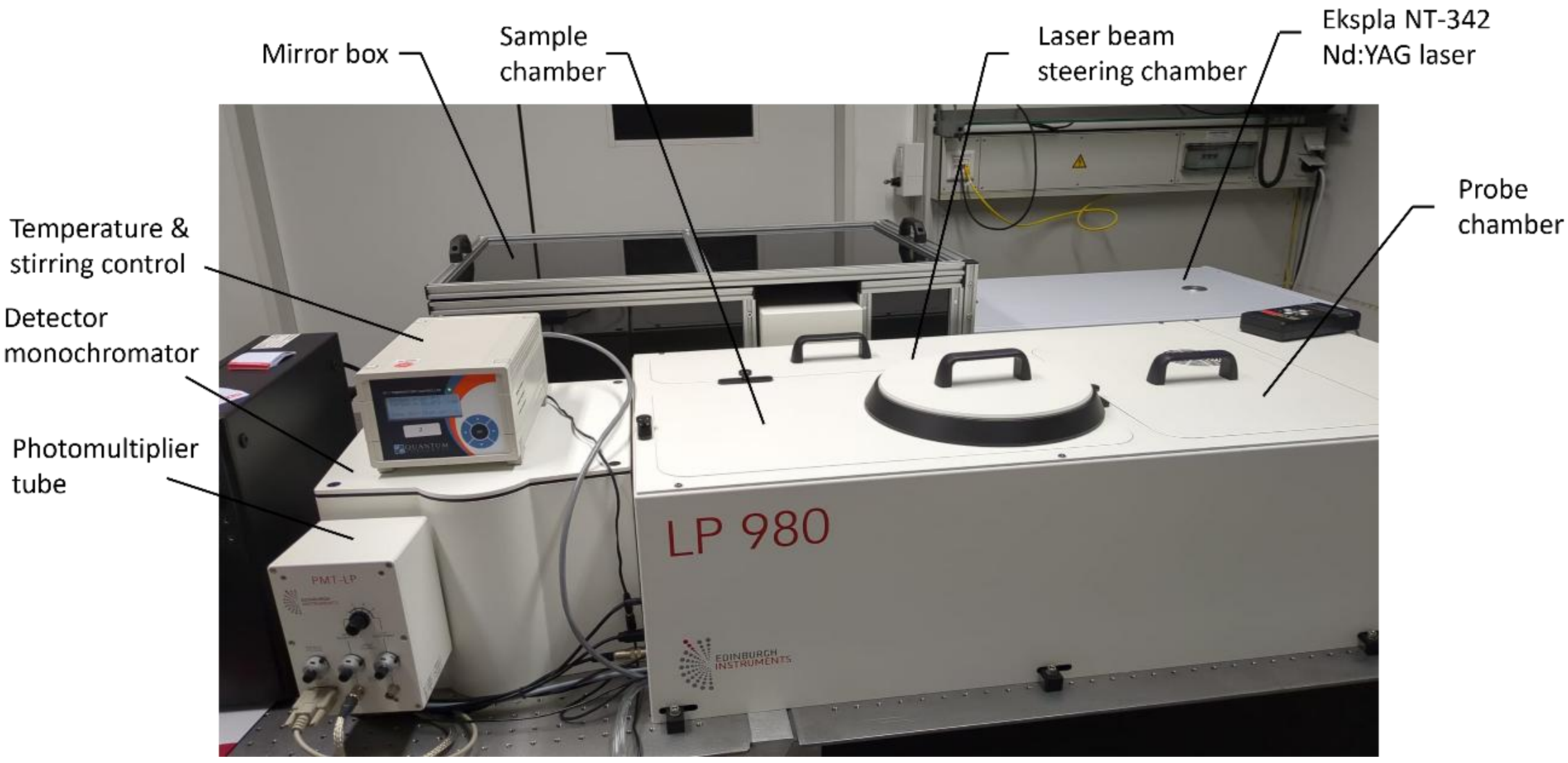

Figure S91: Outside of the LP980 with the key parts highlighted.

Section S7: Time-resolved photoacoustic signal and linear fits

All PAS measurements were performed at 293 K under continuous stirring. The laser pulse energy values measured with the power meter before the sample and corrected by the corresponding calibration factor are indicated in each figure.

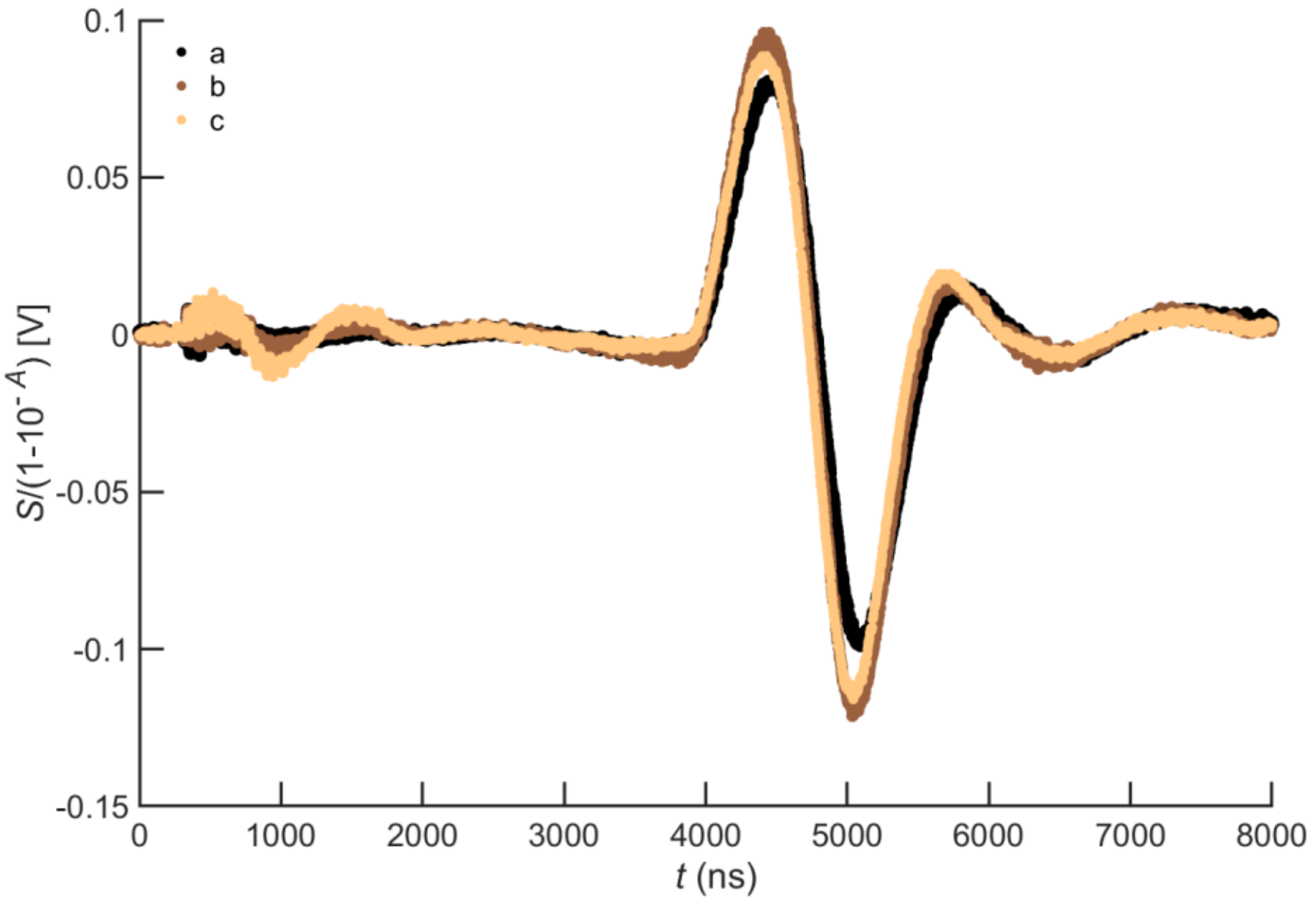

Figure S92: Normalized instrumentally averaged photoacoustic signal of (a) NC before sample measurement; (b) **NiPc-1** and (c) NC after sample measurement, in H_2O with a ND filter of OD 0.1 before the beam splitter. Laser pulse energy of the incident beam measured with the power sensor placed before the sample: (a) 3.0 mJ, (b) 3.3 mJ, and (c) 3.3 mJ. Laser pulse energy of the transmitted beam measured with the power sensor placed after the sample: below the experimental uncertainty (*ca.* 0.5 mJ).

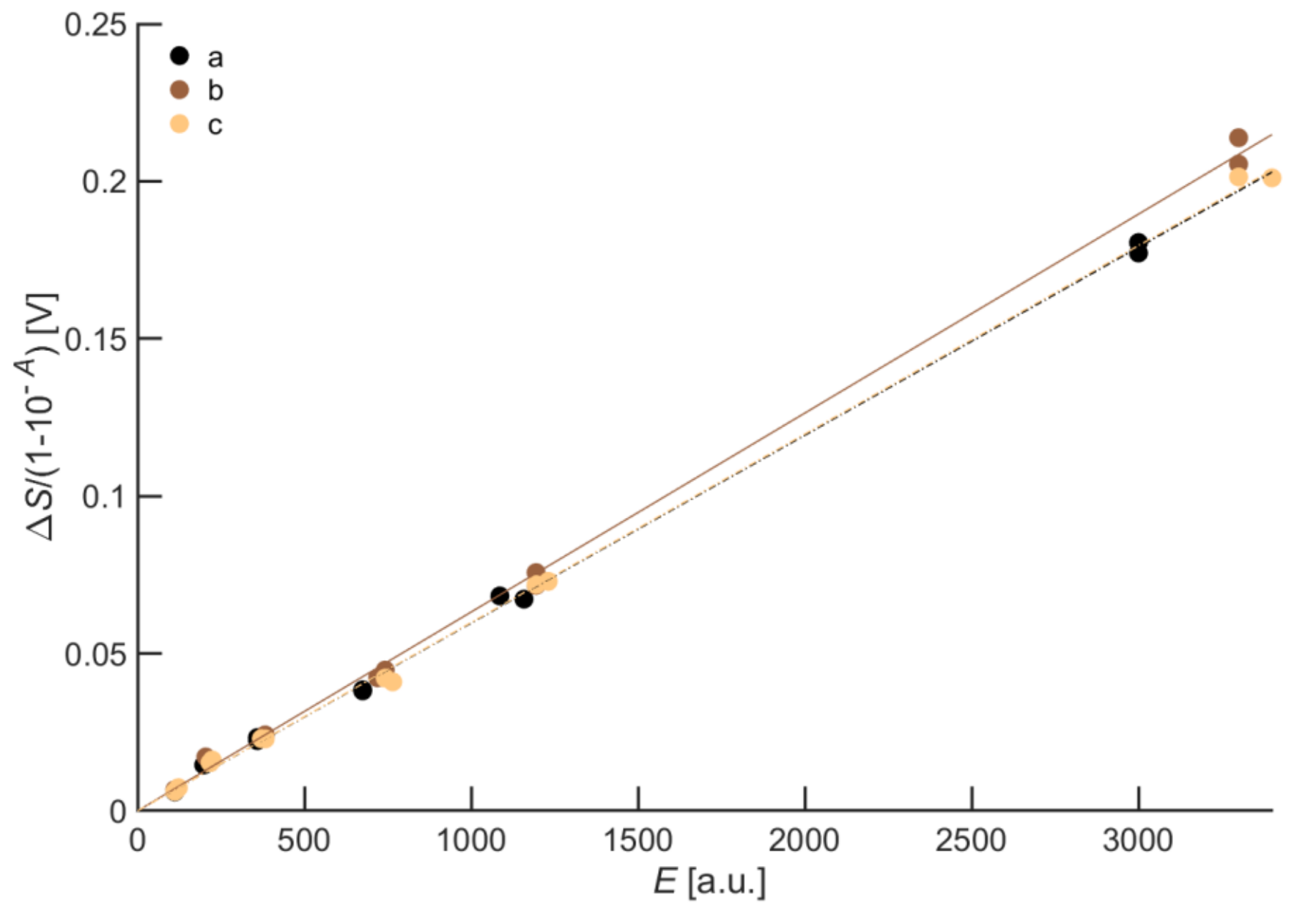

Figure S93: Normalized Δ*S* measured as a function of absorbed relative energy of NC before (a); **NiPc-1** (b) and NC after (c) in H_2O with a ND filter of OD 0.1 before the beam splitter. The solid and dashed lines are the least-squares linear fits through the data points of (a), (b) and (c). The inset is the calculated α_s by dividing the slopes of the linear fits of (a) and (b).

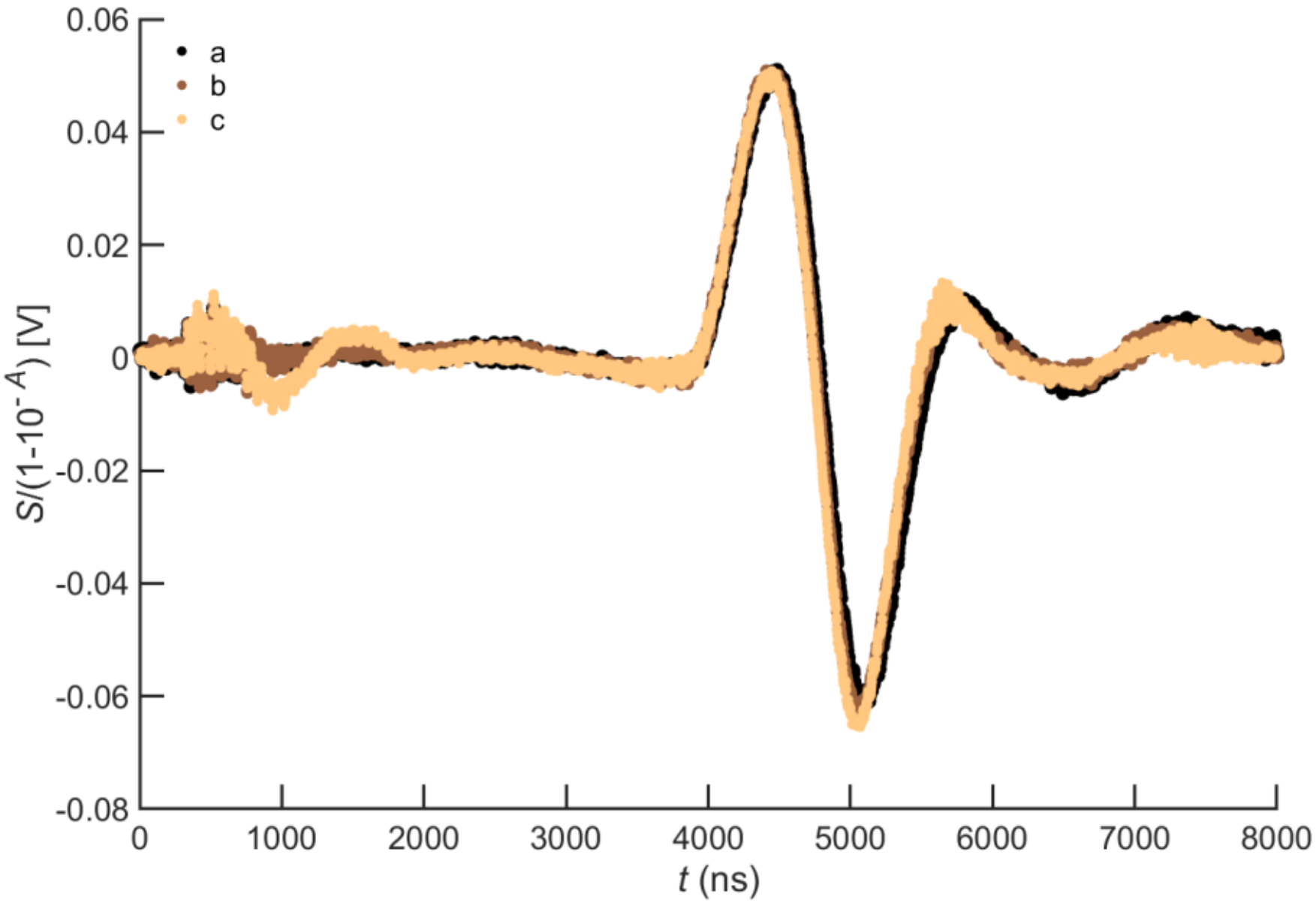

Figure S94: Normalized instrumentally averaged photoacoustic signal of (a) NC before sample measurement; (b) **NiPc-1** and (c) NC after sample measurement, in H_2O with a ND filter of OD 0.2 before the beam splitter. Laser pulse energy of the incident beam measured with the power sensor placed before the sample: (a) 1.9 mJ, (b) 1.9 mJ, and (c) 2.0 mJ. Laser pulse energy of the transmitted beam measured with the power sensor placed after the sample: below the experimental uncertainty (*ca.* 0.5 mJ).

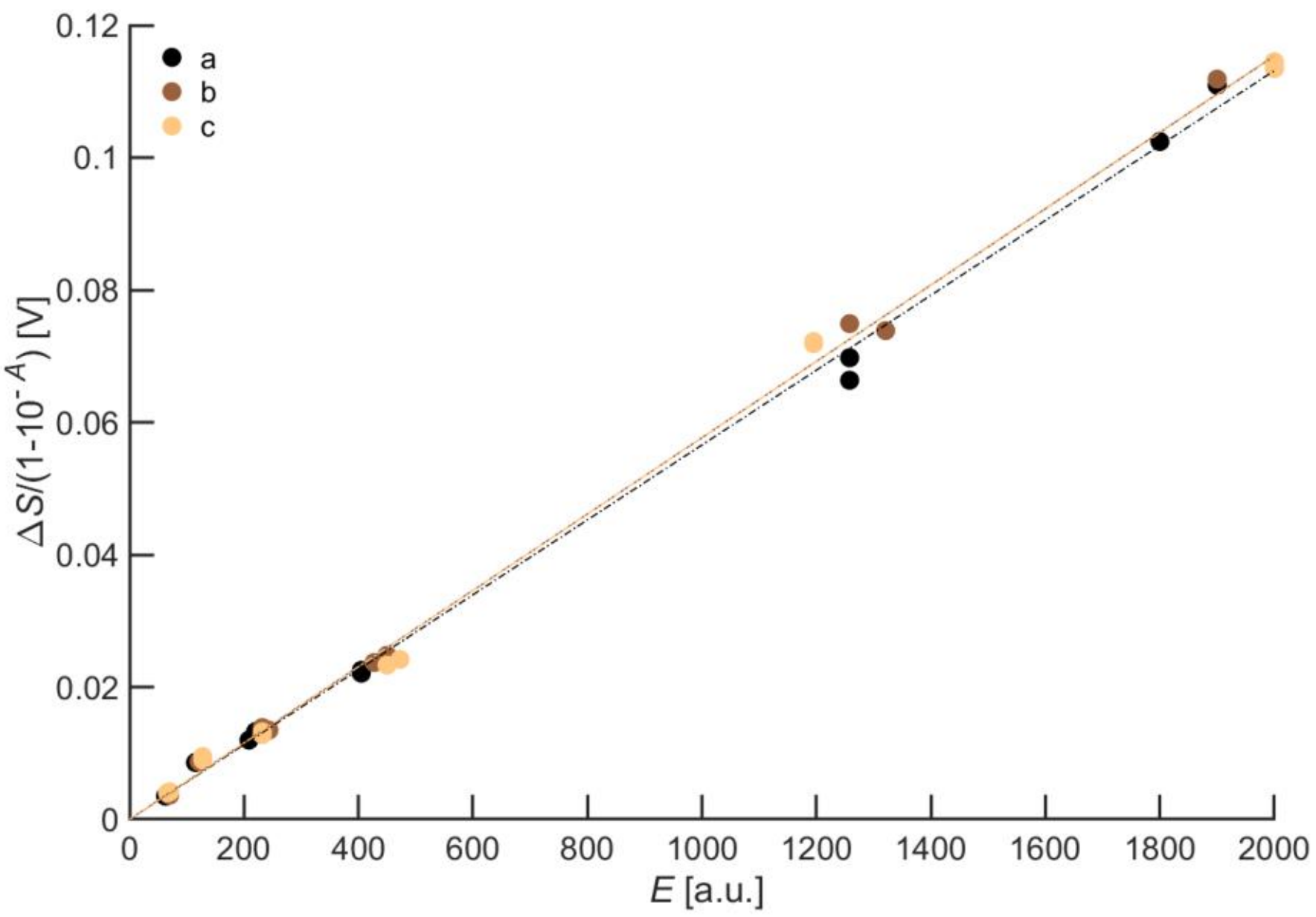

Figure S95: Normalized Δ*S* measured as a function of absorbed relative energy of NC before (a); **NiPc-1** (b) and NC after (c) in H_2O with a ND filter of OD 0.2 before the beam splitter. The solid and dashed lines are the least-squares linear fits

through the data points of (a), (b) and (c). The inset is the calculated α_s by dividing the slopes of the linear fits of (a) and (b).

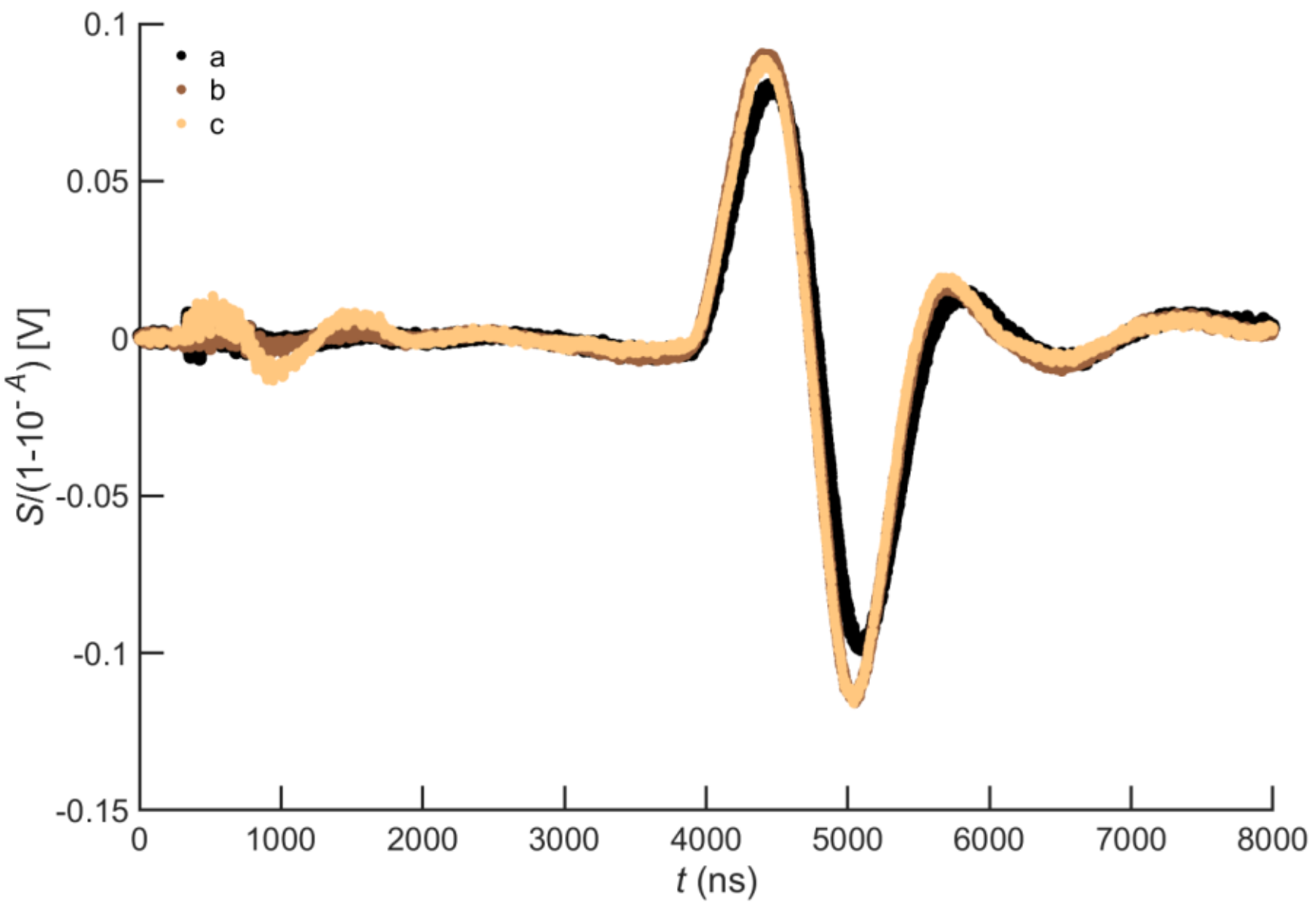

Figure S96: Normalized instrumentally averaged photoacoustic signal of (a) NC before sample measurement; (b) **NiPc-2** and (c) NC after sample measurement, in H_2O with a ND filter of OD 0.1 before the beam splitter. Laser pulse energy of the incident beam measured with the power sensor placed before the sample: (a) 3.0 mJ, (b) 3.4 mJ, and (c) 3.3 mJ. Laser pulse energy of the transmitted beam measured with the power sensor placed after the sample: below the experimental uncertainty (*ca.* 0.5 mJ).

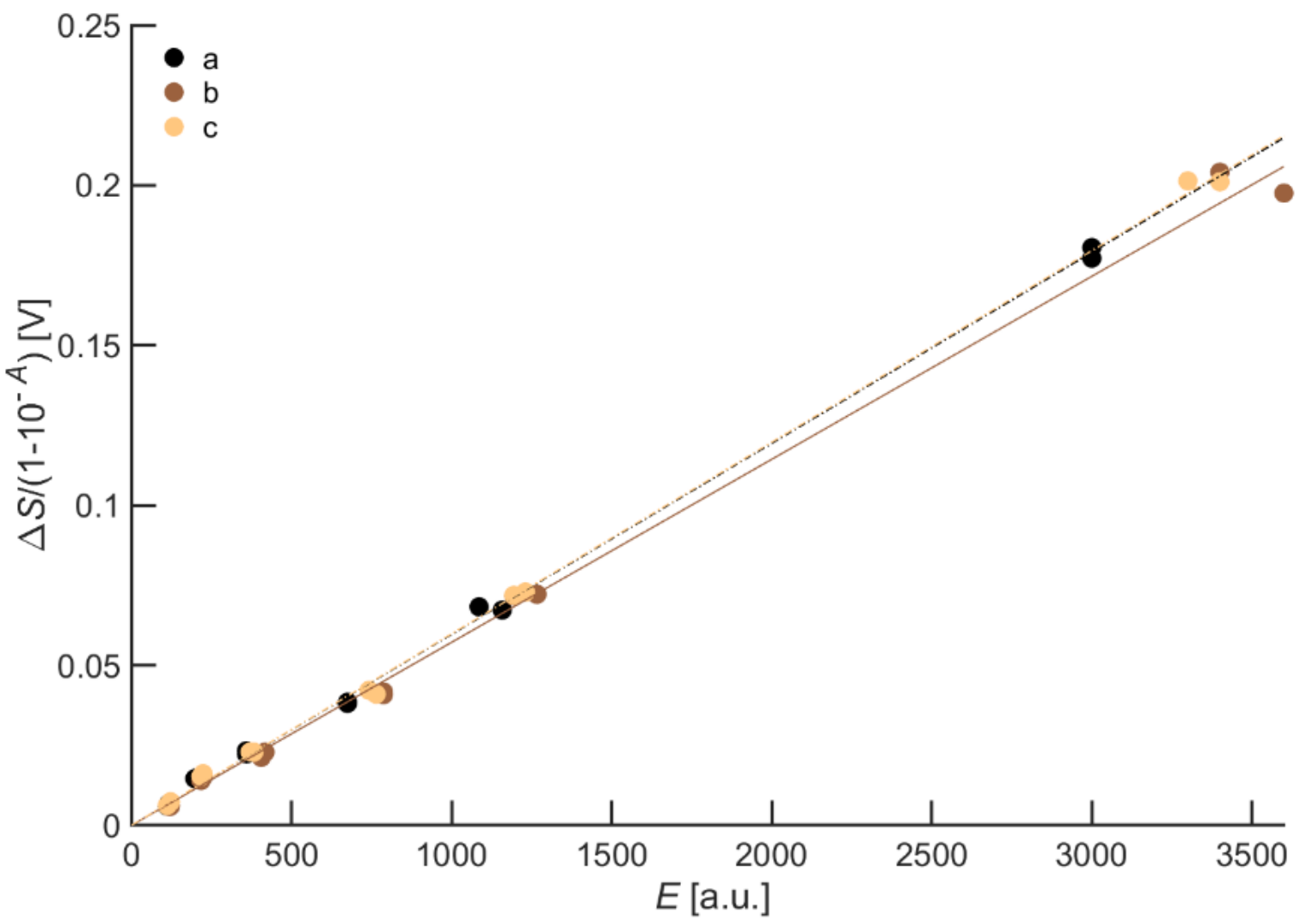

Figure S97: Normalized Δ*S* measured as a function of absorbed relative energy of NC before (a); **NiPc-2** (b) and NC after (c) in H_2O with a ND filter of OD 0.1. The solid and dashed lines are the least-squares linear fits through the data points of (a), (b) and (c). The inset is the calculated α_s by dividing the slopes of the linear fits of (a) and (b).

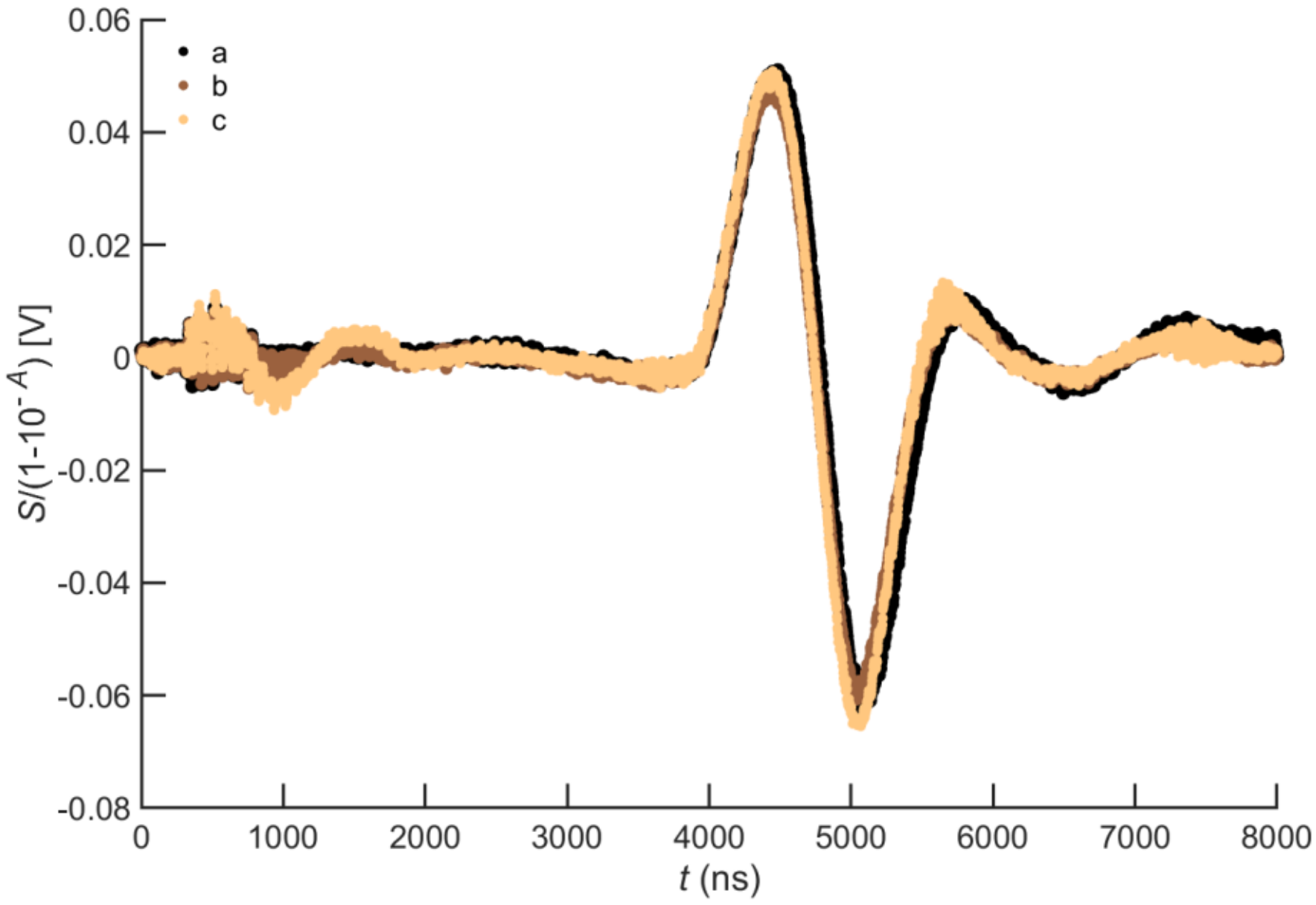

Figure S98: Normalized instrumentally averaged photoacoustic signal of (a) NC before sample measurement; (b) **NiPc-2** and (c) NC after sample measurement, in H_2O with a ND filter of OD 0.2 before the beam splitter. Laser pulse energy of the incident beam measured with the power sensor placed before the sample: (a) 1.9 mJ, (b) 1.9 mJ, and (c) 2.0 mJ. Laser pulse energy of the transmitted beam measured with the power sensor placed after the sample: below the experimental uncertainty (*ca.* 0.5 mJ).

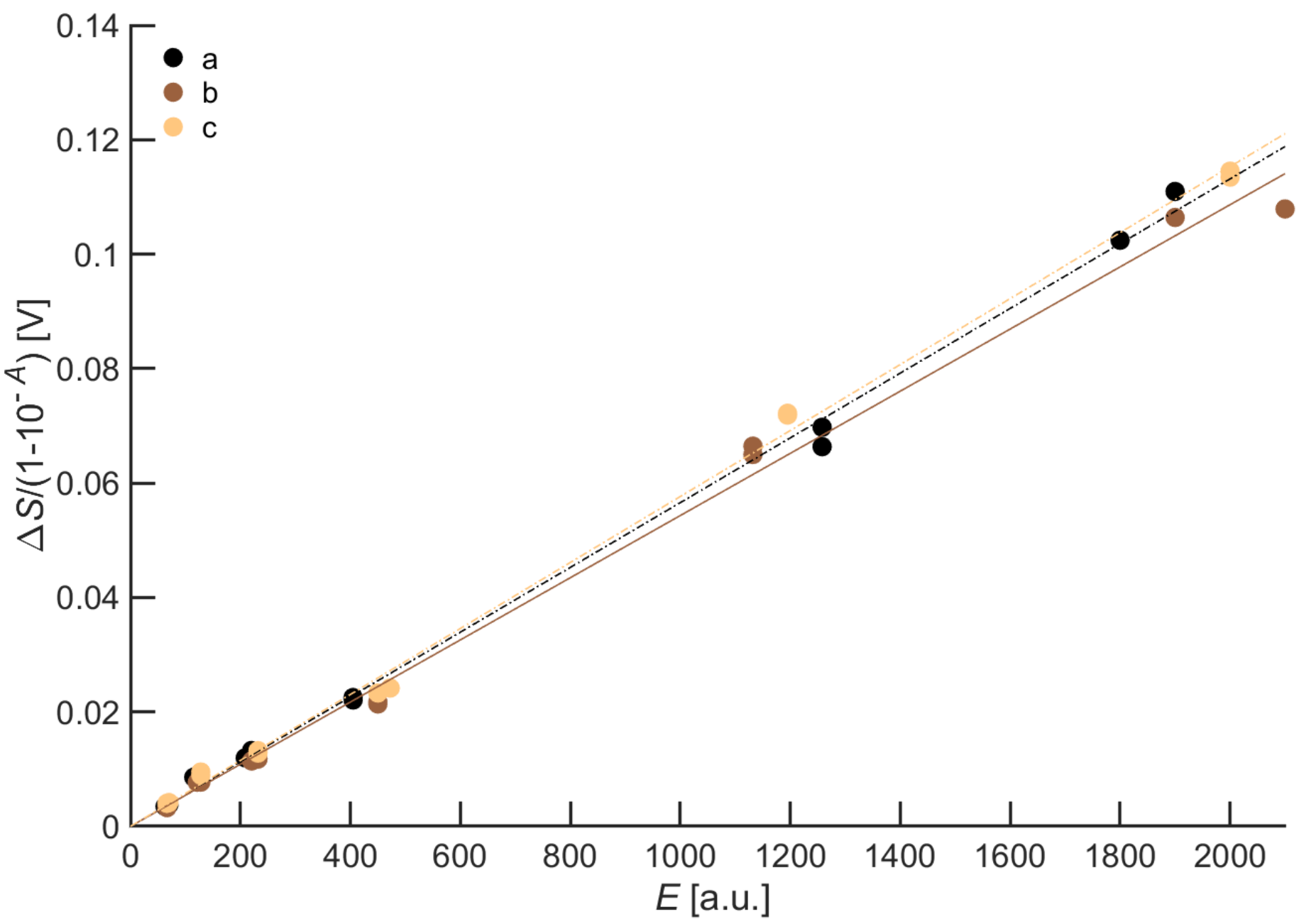

Figure S99: Normalized ΔS measured as a function of absorbed relative energy of NC before (a); **NiPc-2** (b) and NC after (c) in H_2O with a ND filter of OD 0.2. The solid and dashed lines are the least-squares linear fits through the data points of (a), (b) and (c). The inset is the calculated α_s by dividing the slopes of the linear fits of (a) and (b).

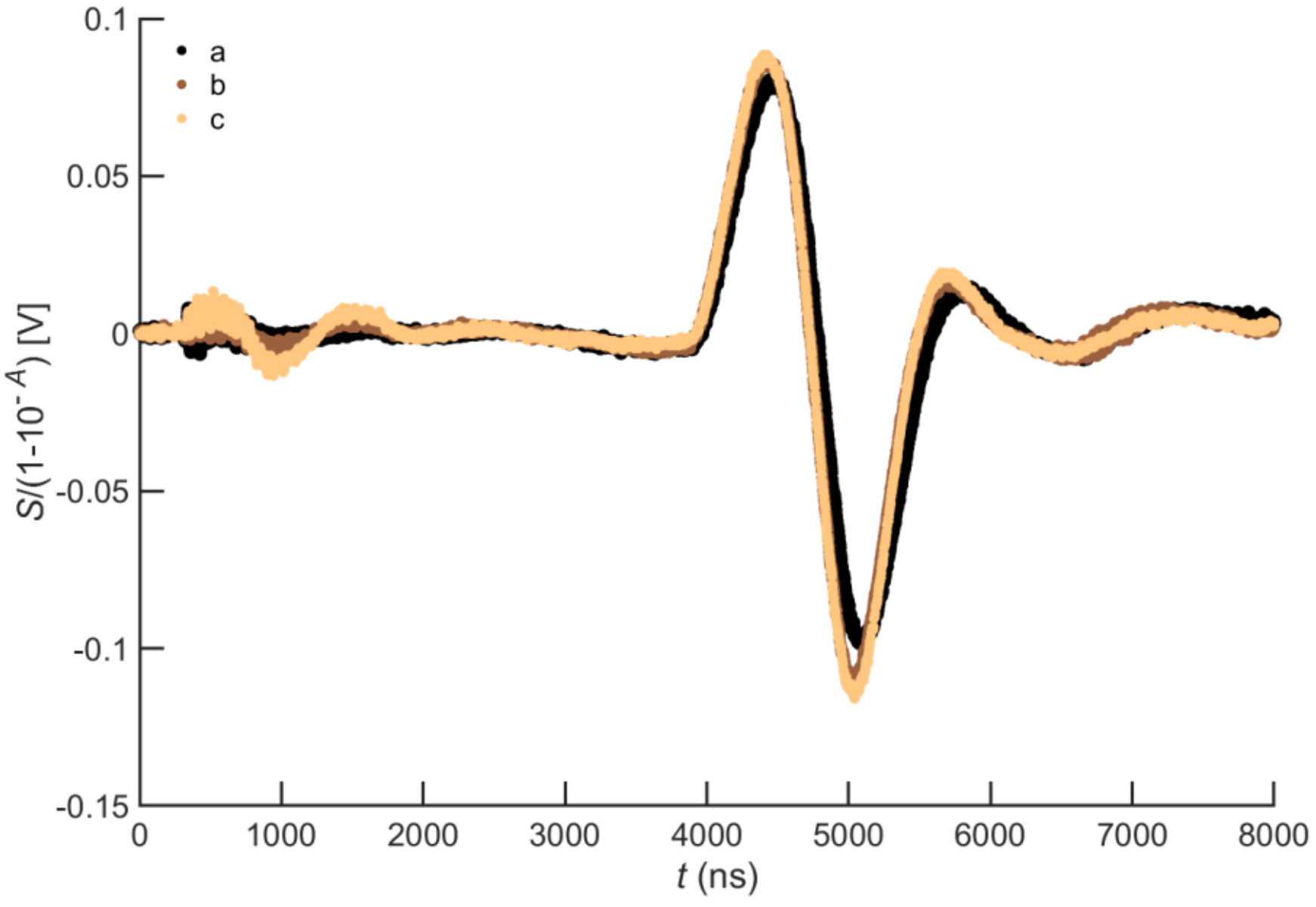

Figure S100: Normalized instrumentally averaged photoacoustic signal of (a) NC before sample measurement; (b) **NiPc-3** and (c) NC after sample measurement, in H_2O with a ND filter of OD 0.1 before the beam splitter. Laser pulse energy of the incident beam measured with the power sensor placed before the sample: (a) 3.0 mJ, (b) 3.4 mJ, and (c) 3.3 mJ. Laser pulse energy of the transmitted beam measured with the power sensor placed after the sample: below the experimental uncertainty (*ca.* 0.5 mJ).

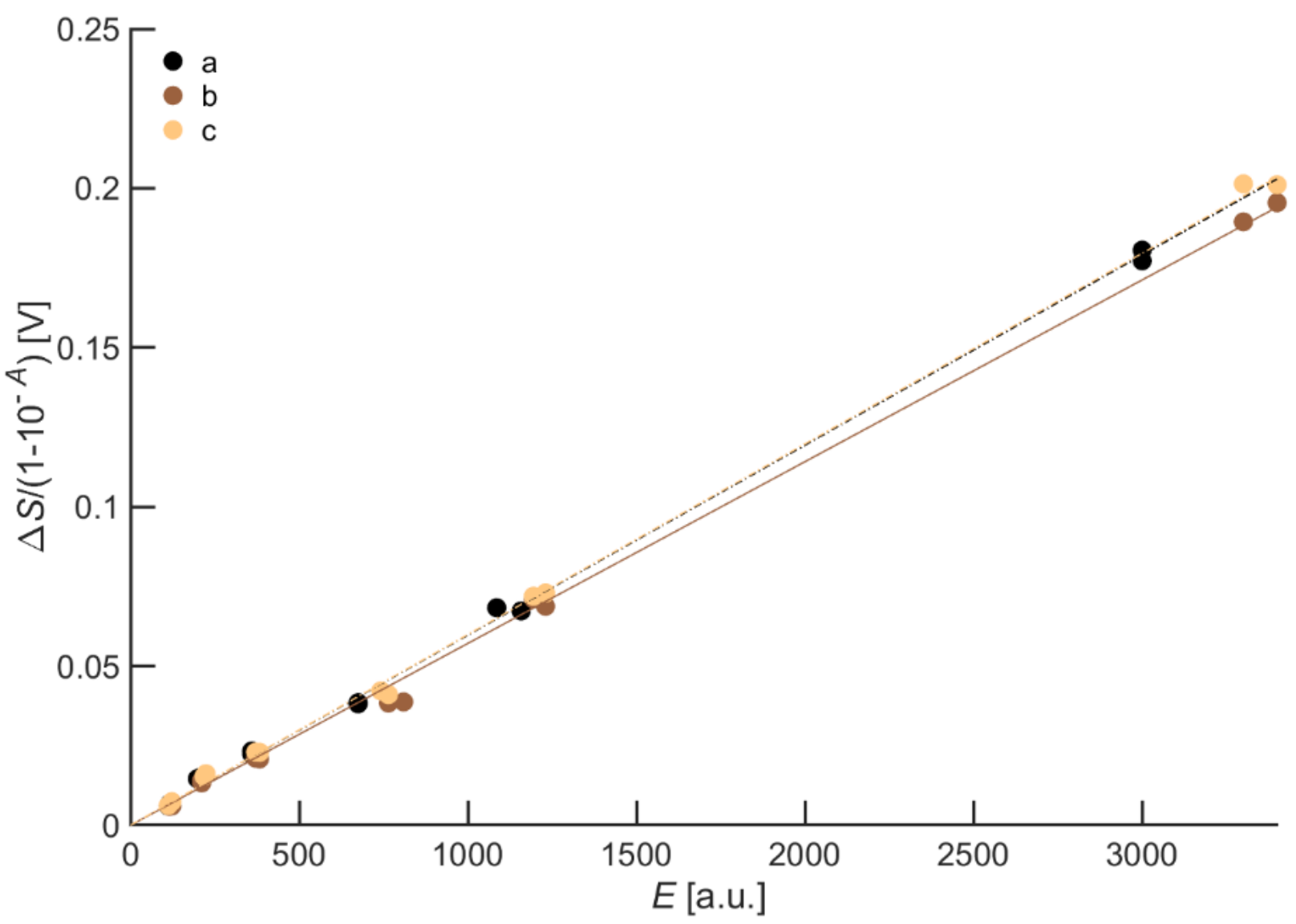

Figure S101: Normalized Δ*S* measured as a function of absorbed relative energy of NC before (a); **NiPc-3** (b) and NC after (c) in H_2O with a ND filter of OD 0.1. The solid and dashed lines are the least-squares linear fits through the data points of

(a), (b) and (c). The inset is the calculated α_S by dividing the slopes of the linear fits of (a) and (b).

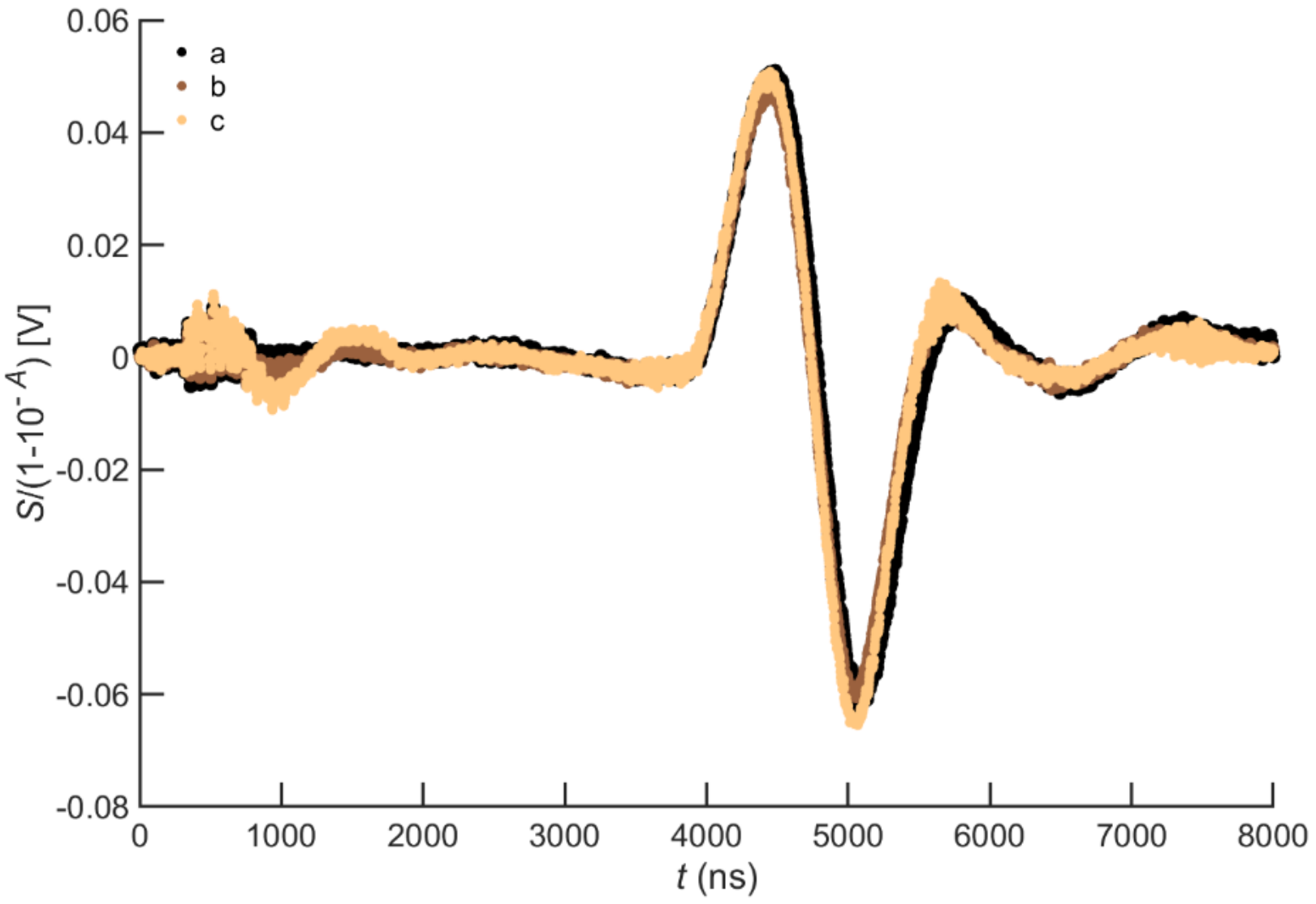

Figure S102: Normalized instrumentally averaged photoacoustic signal of (a) NC before sample measurement; (b) **NiPc-3** and (c) NC after sample measurement, in H_2O with a ND filter of OD 0.2 before the beam splitter. Laser pulse energy of the incident beam measured with the power sensor placed before the sample: (a) 1.9 mJ, (b) 2.0 mJ, and (c) 2.0 mJ. Laser pulse energy of the transmitted beam measured with the power sensor placed after the sample: below the experimental uncertainty (*ca.* 0.5 mJ).

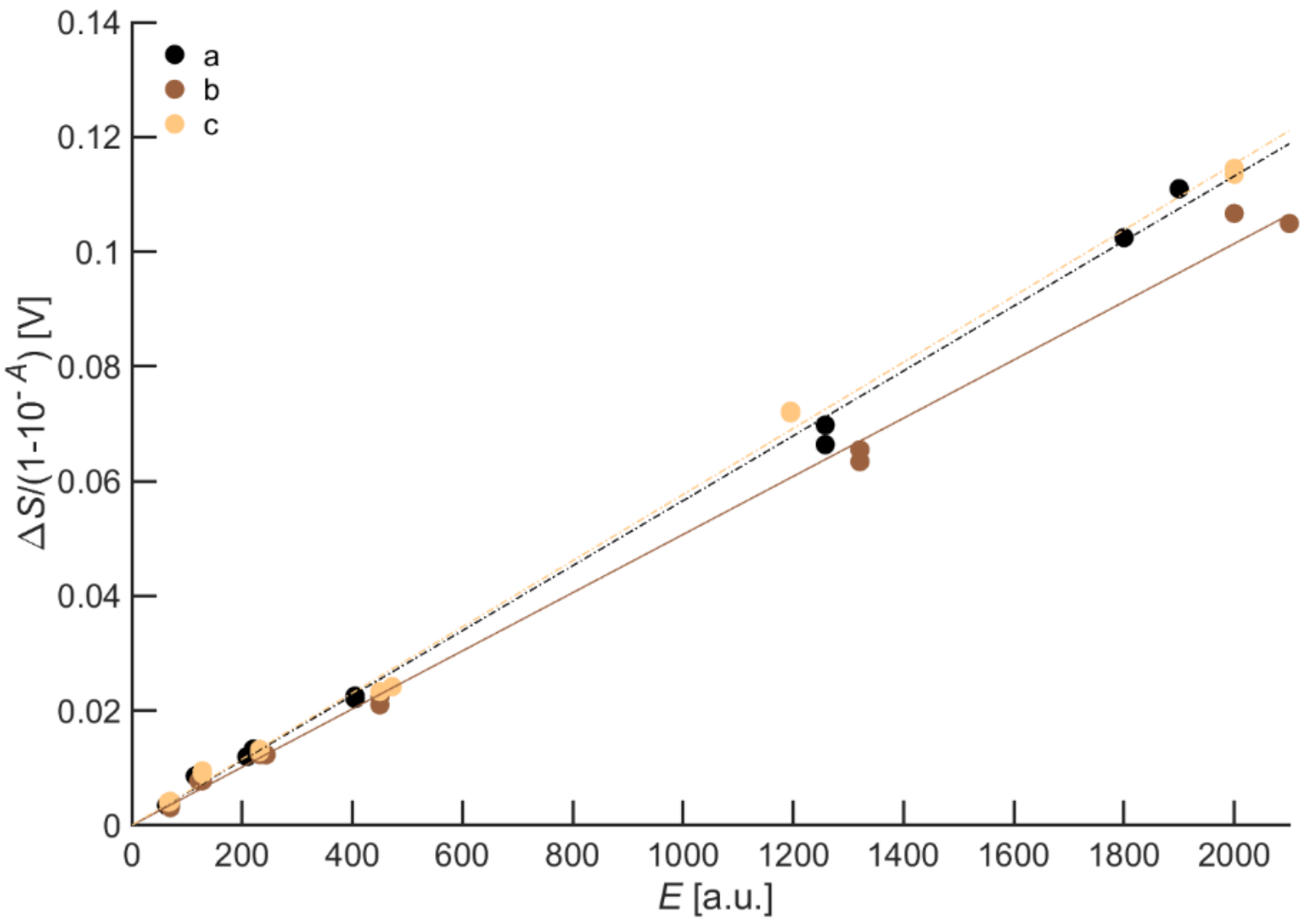

Figure S103: Normalized Δ*S* measured as a function of absorbed relative energy of NC before (a); **NiPc-3** (b) and NC after (c) in H_2O with a ND filter of OD 0.1. The solid and dashed lines are the least-squares linear fits through the data points of (a), (b) and (c). The inset is the calculated α_S by dividing the slopes of the linear fits of (a) and (b).

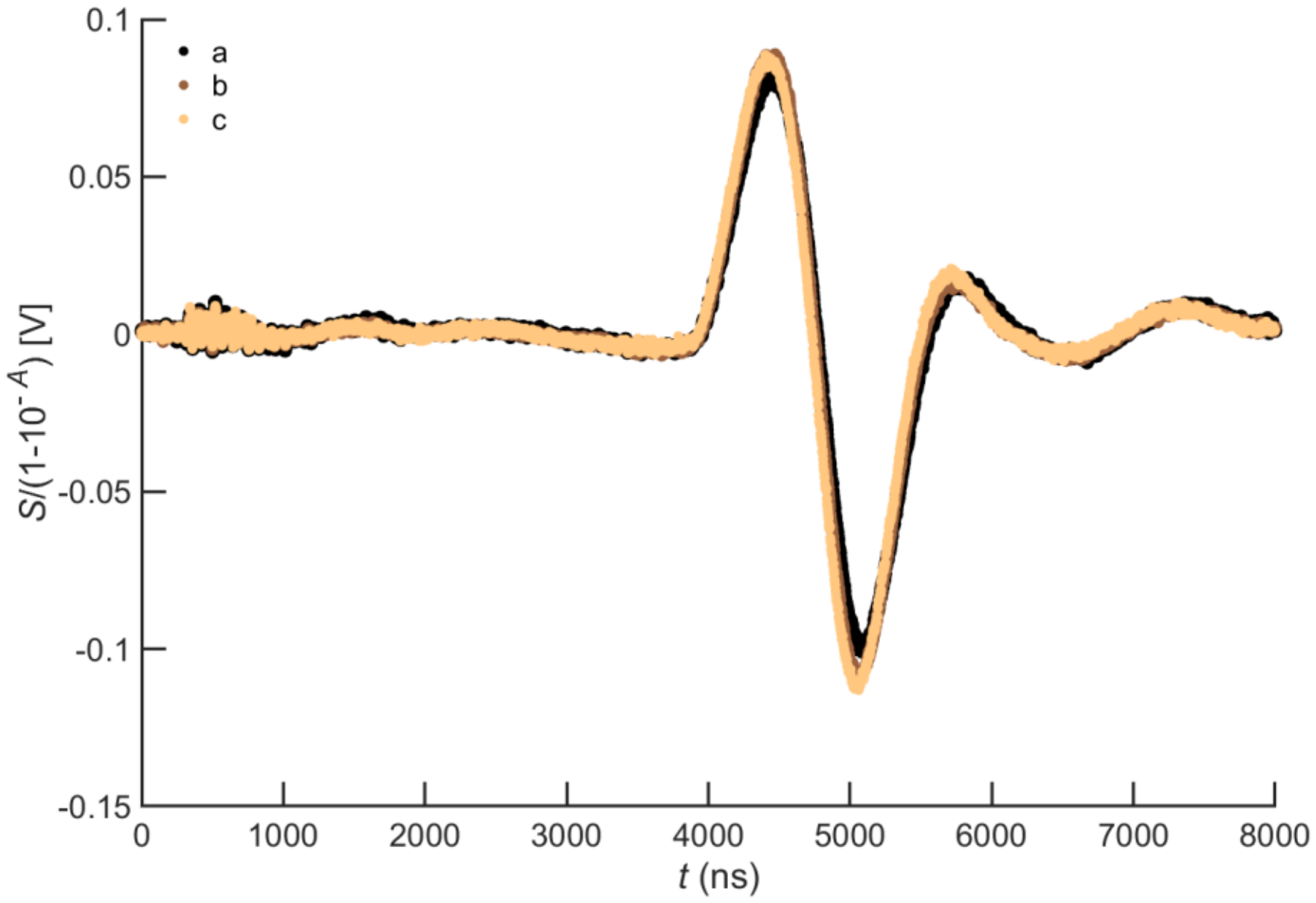

Figure S104: Normalized instrumentally averaged photoacoustic signal of (a) NC before sample measurement; (b) **NiPc-4** and (c) NC after sample measurement, in H_2O with a ND filter of OD 0.1 before the beam splitter. Laser pulse energy of the incident beam measured with the power sensor placed before the sample: (a) 3.3 mJ, (b) 3.5 mJ, and (c) 3.5 mJ. Laser pulse energy of the transmitted beam measured with the power sensor placed after the sample: below the experimental uncertainty (*ca.* 0.5 mJ).

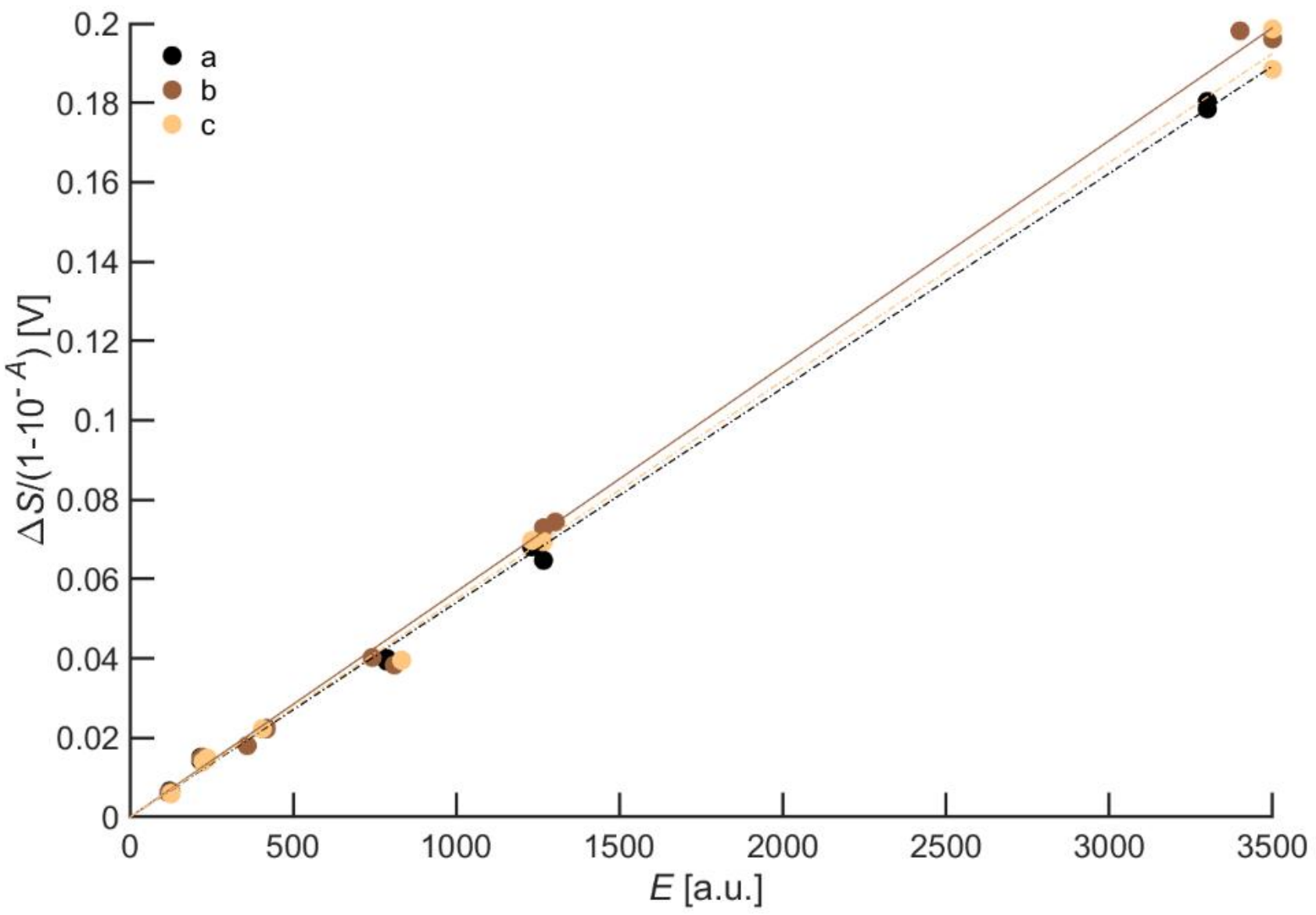

Figure S105: Normalized Δ*S* measured as a function of absorbed relative energy of NC before (a); **NiPc-4** (b) and NC after (c) in H_2O with a ND filter of OD 0.1. The solid and dashed lines are the least-squares linear fits through the data points of

(a), (b) and (c). The inset is the calculated α_S by dividing the slopes of the linear fits of (a) and (b).

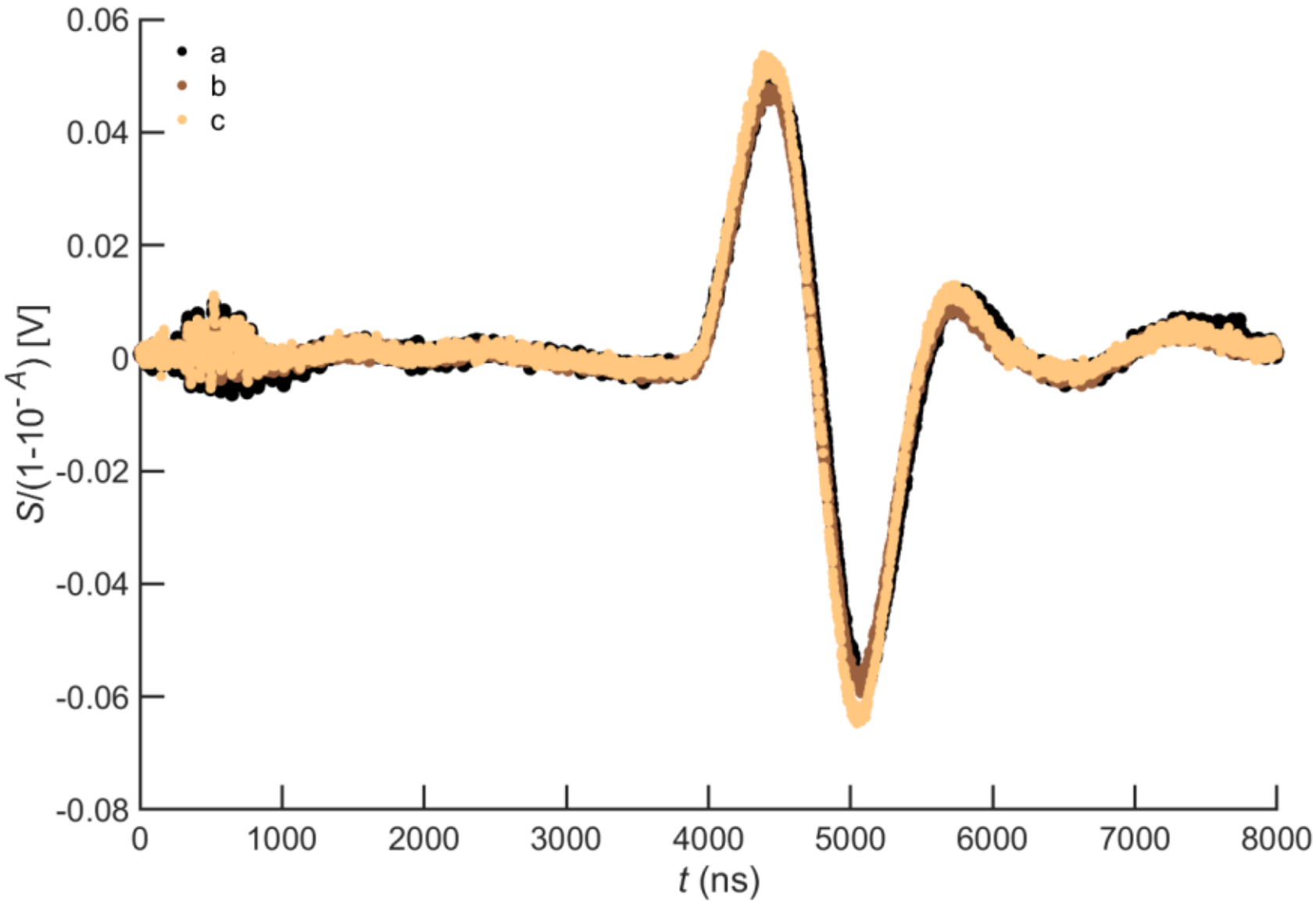

Figure S106: Normalized instrumentally averaged photoacoustic signal of (a) NC before sample measurement; (b) **NiPc-4** and (c) NC after sample measurement, in H_2O with a ND filter of OD 0.2 before the beam splitter. Laser pulse energy of the incident beam measured with the power sensor placed before the sample: (a) 2.0 mJ, (b) 2.0 mJ, and (c) 2.1 mJ. Laser pulse energy of the transmitted beam measured with the power sensor placed after the sample: below the experimental uncertainty (*ca.* 0.5 mJ).

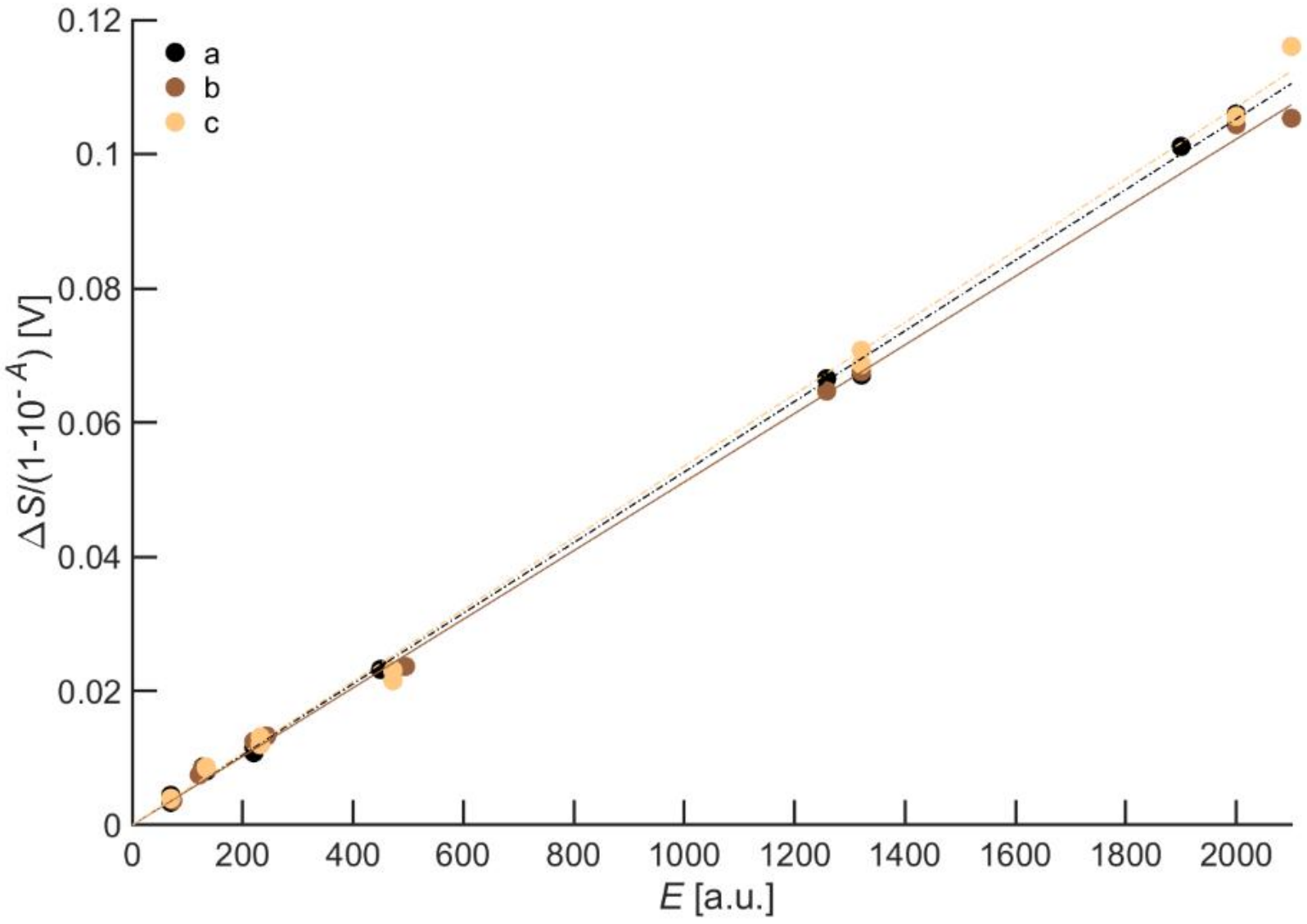

Figure S107: Normalized Δ*S* measured as a function of absorbed relative energy of NC before (a); **NiPc-4** (b) and NC after (c) in H_2O with a ND filter of OD 0.2. The solid and dashed lines are the least-squares linear fits through the data points of (a), (b) and (c). The inset is the calculated α_S by dividing the slopes of the linear fits of (a) and (b).

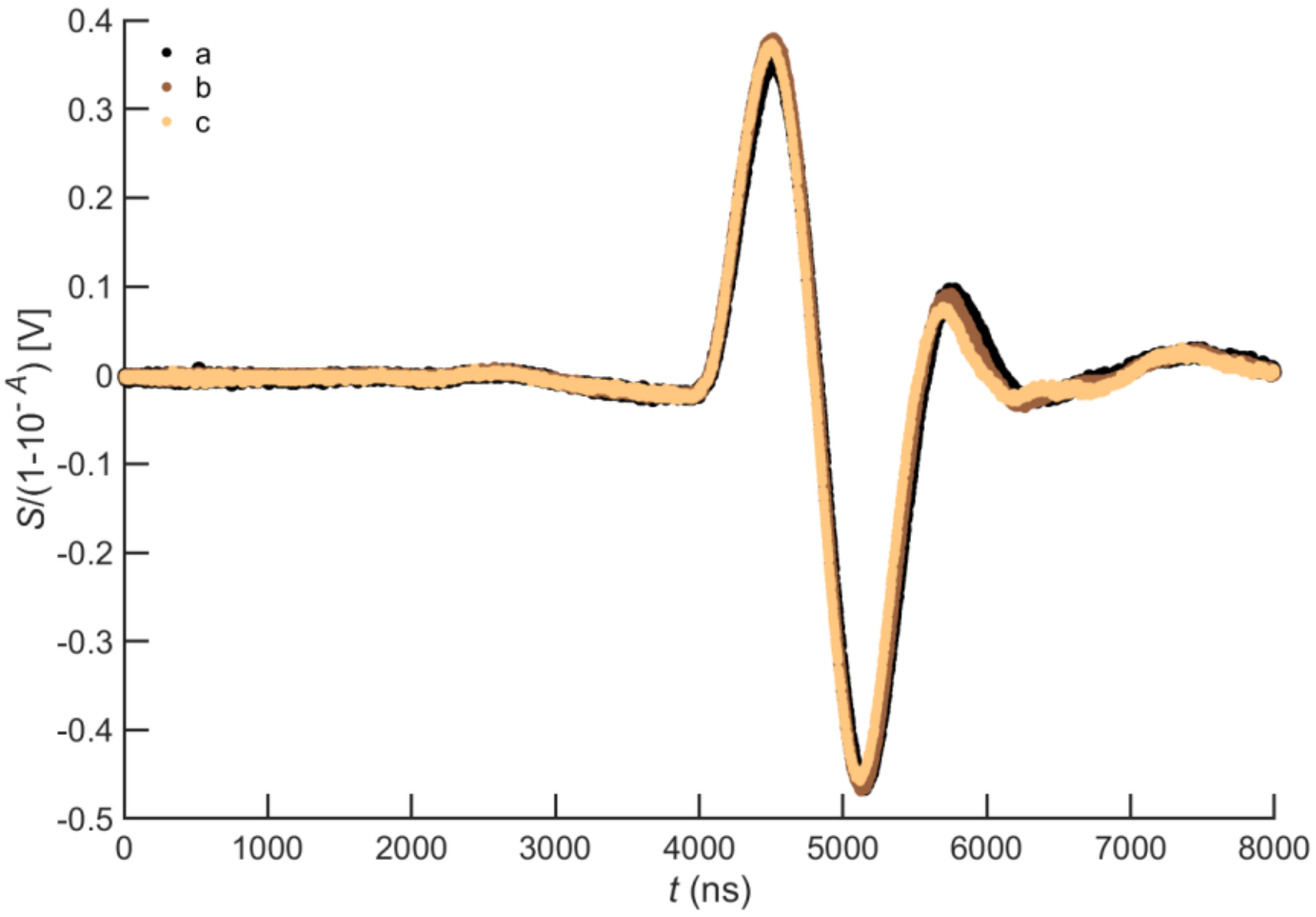

Figure S108: Normalized instrumentally averaged photoacoustic signal of (a) OHBP before sample measurement; (b) **NiPc-1** and (c) OHBP after sample measurement, in DMSO with a ND filter of OD 0.3 before the beam splitter. Laser pulse energy of the incident beam measured with the power sensor placed before the sample: (a) 1.5 mJ, (b) 1.5 mJ, and (c) 1.5 mJ. Laser pulse energy of the transmitted beam measured with the power sensor placed after the sample: below the experimental uncertainty (*ca.* 0.5 mJ).

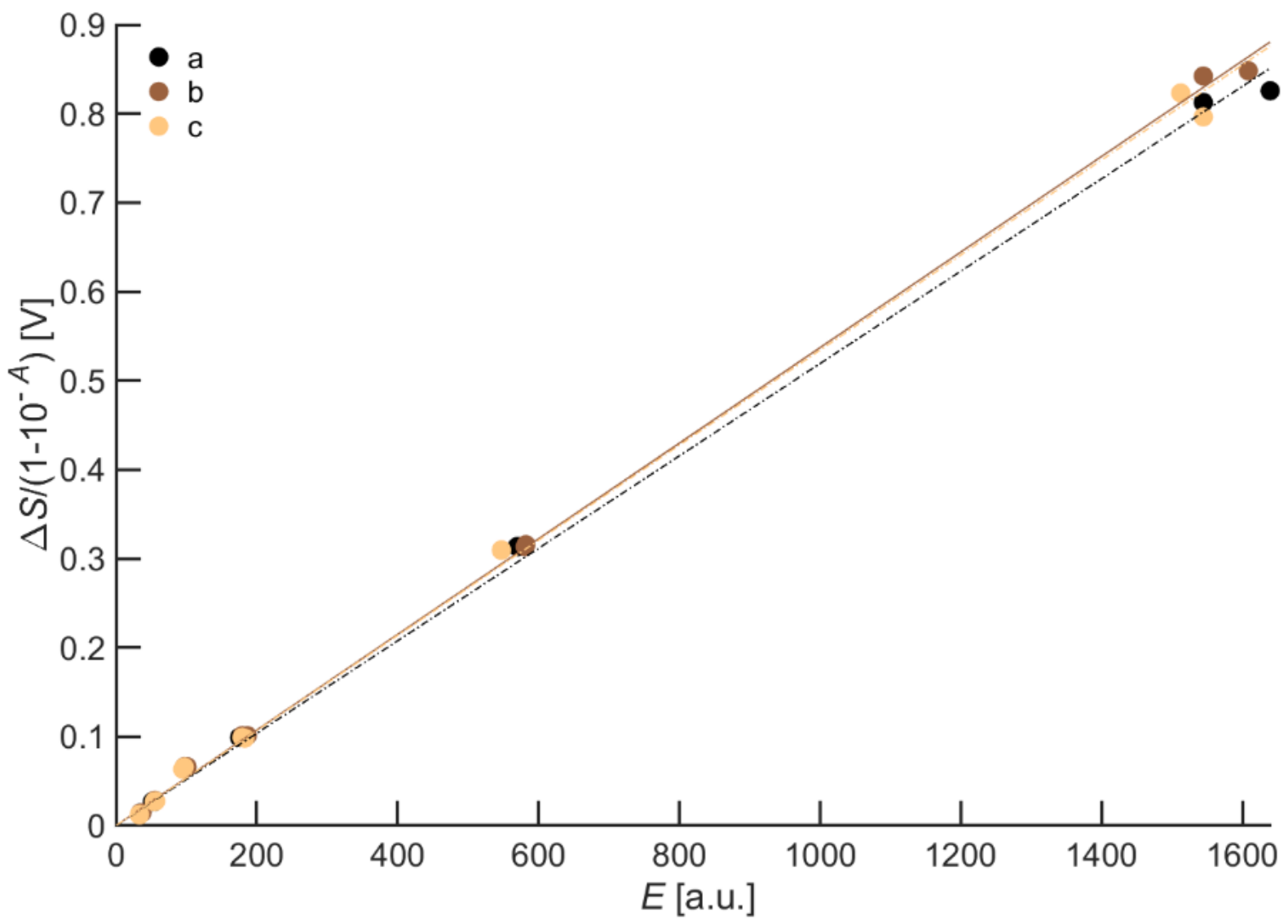

Figure S109: Normalized Δ*S* measured as a function of absorbed relative energy of OHBP before (a); **NiPc-1** (b) and OHBP after (c) in DMSO with a ND filter of OD 0.3. The solid and dashed lines are the least-squares linear fits through the data points of (a), (b) and (c). The inset is the calculated α_S by dividing the slopes of the linear fits of (a) and (b).

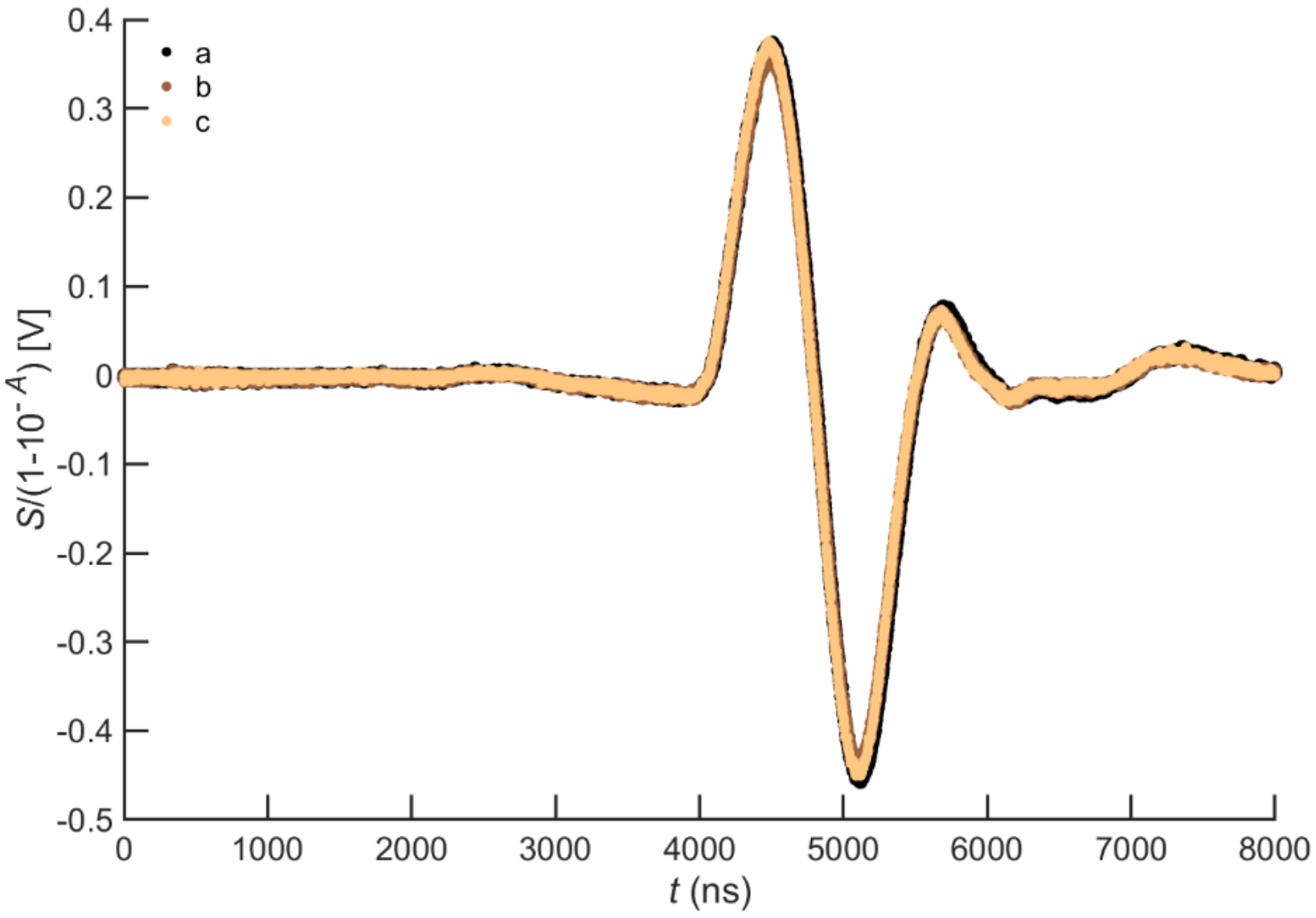

Figure S110: Normalized instrumentally averaged photoacoustic signal of (a) OHBP before sample measurement; (b) **NiPc-2** and (c) OHBP after sample measurement, in DMSO with a ND filter of OD 0.3 before the beam splitter. Laser pulse energy of the incident beam measured with the power sensor placed before the sample: (a) 1.5 mJ, (b) 1.4 mJ, and (c) 1.5 mJ. Laser pulse energy of the transmitted beam measured with the power sensor placed after the sample: below the experimental uncertainty (*ca.* 0.5 mJ).

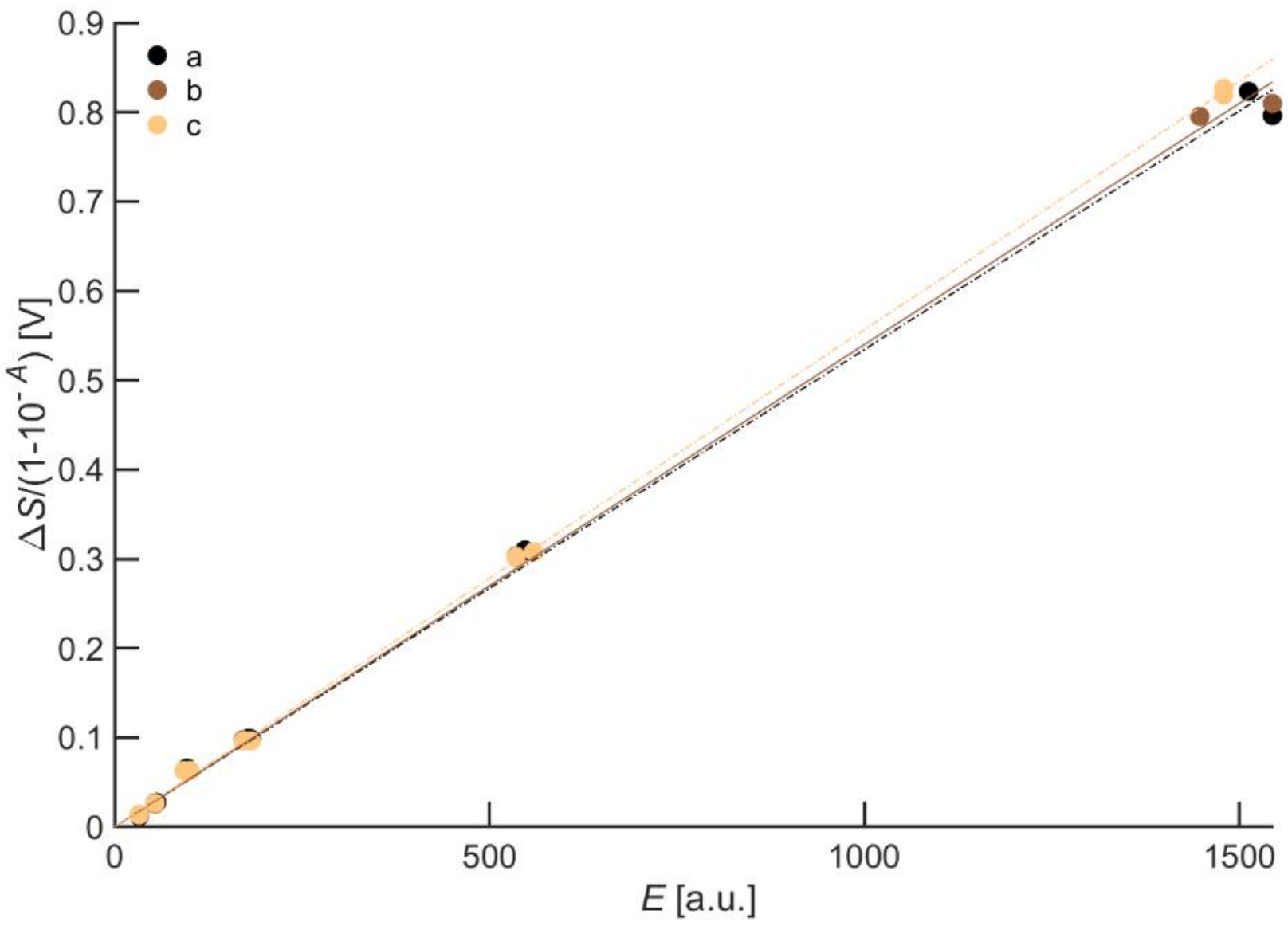

Figure S111: Normalized Δ*S* measured as a function of absorbed relative energy of OHBP before (a); **NiPc-2** (b) and OHBP after (c) in DMSO with a ND filter of OD 0.3. The solid and dashed lines are the least-squares linear fits through the data points of (a), (b) and (c). The inset is the calculated α_S by dividing the slopes of the linear fits of (a) and (b).

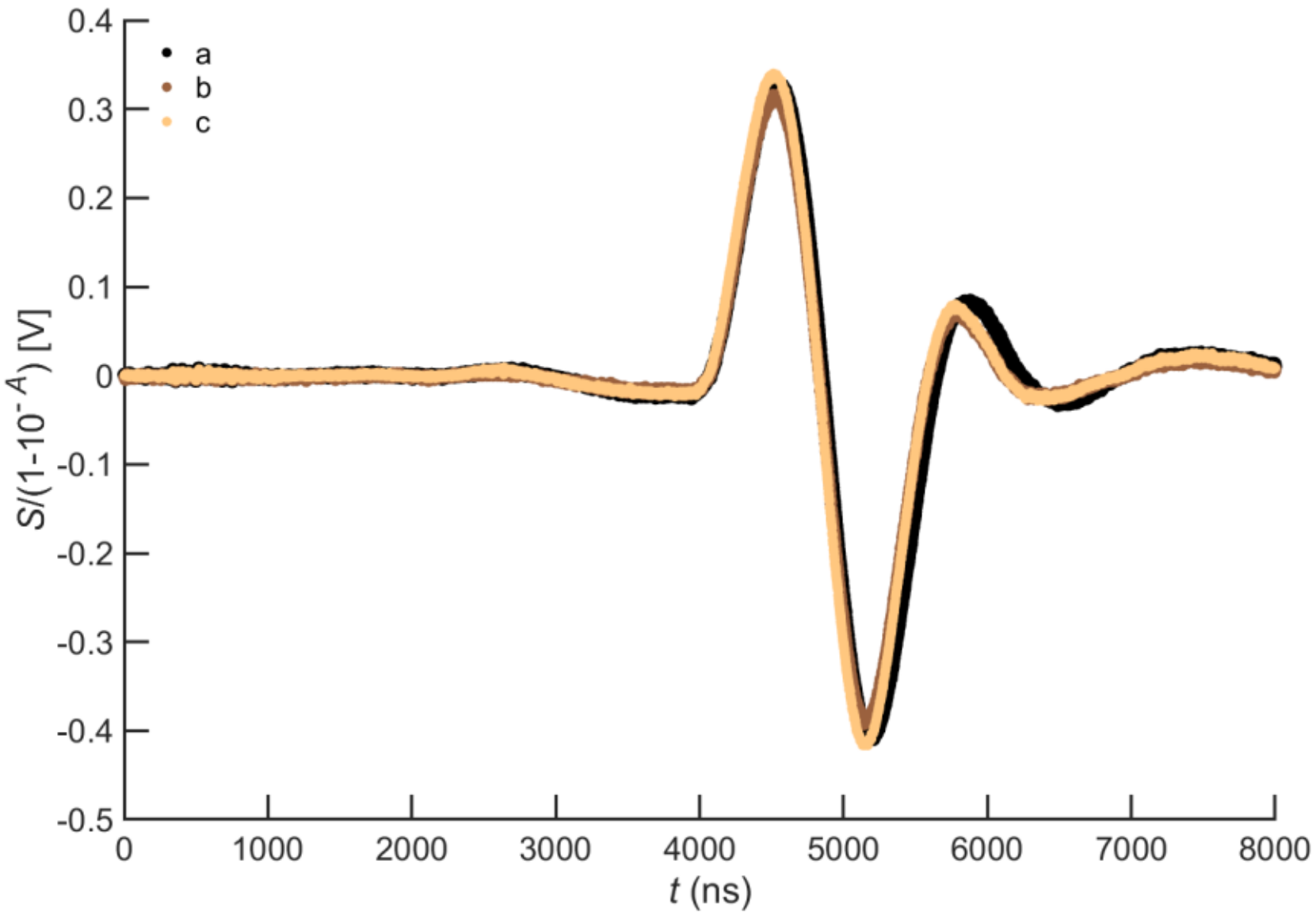

Figure S112: Normalized instrumentally averaged photoacoustic signal of (a) OHBP before sample measurement; (b) **NiPc-3** and (c) OHBP after sample measurement, in DMSO with a ND filter of OD 0.3 before the beam splitter. Laser pulse energy of the incident beam measured with the power sensor placed before the sample: (a) 1.5 mJ, (b) 1.5 mJ, and (c) 1.6 mJ. Laser pulse energy of the transmitted beam measured with the power sensor placed after the sample: below the experimental uncertainty (*ca.* 0.5 mJ).

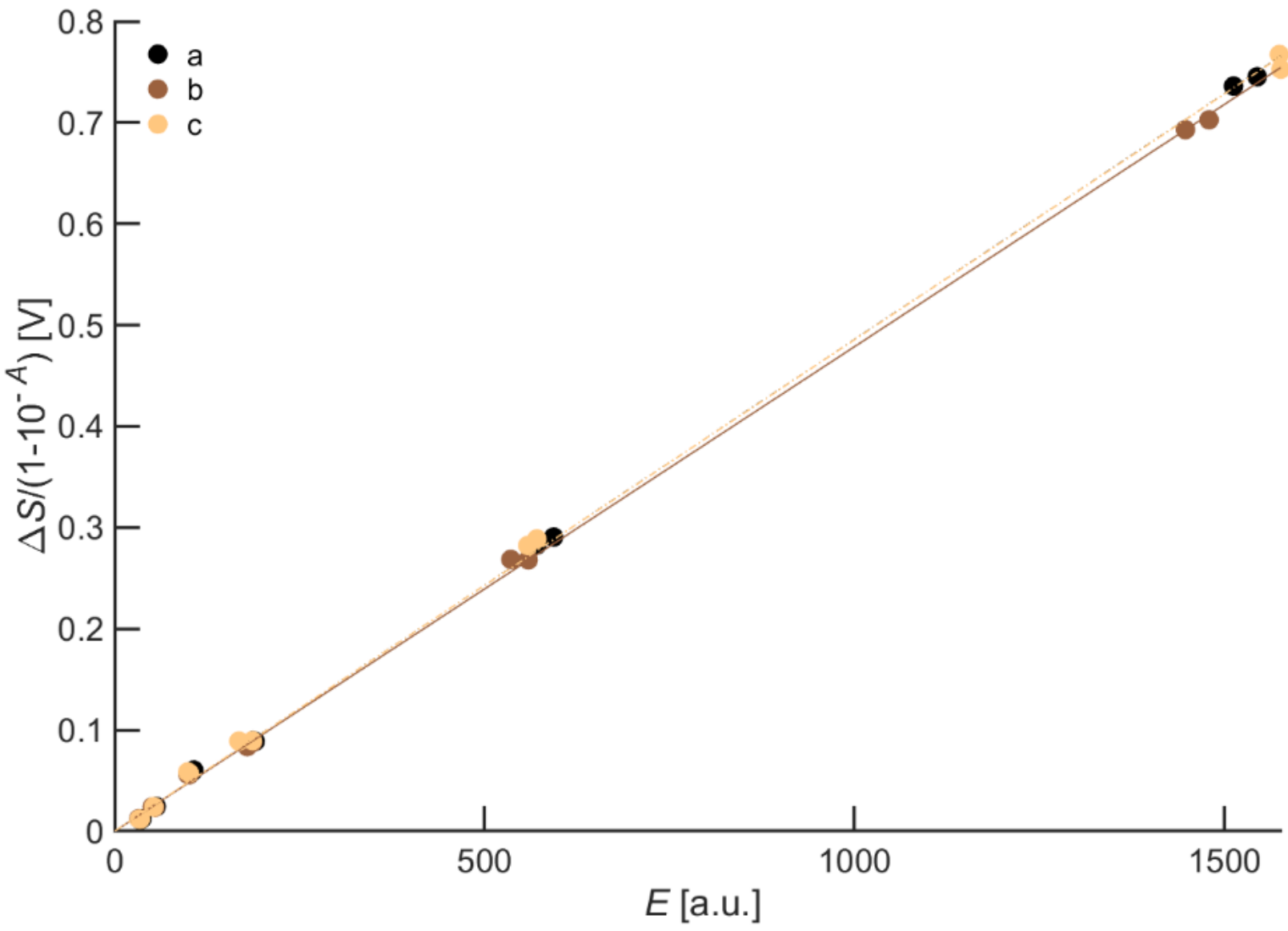

Figure S113: Normalized Δ*S* measured as a function of absorbed relative energy of OHBP before (a); **NiPc-3** (b) and OHBP after (c) in DMSO with a ND filter of OD 0.3. The solid and dashed lines are the least-squares linear fits through the data points of (a), (b) and (c). The inset is the calculated α_S by dividing the slopes of the linear fits of (a) and (b).

Section S8: Comparison between instrumental averaging and Savitsky-Golay smoothening

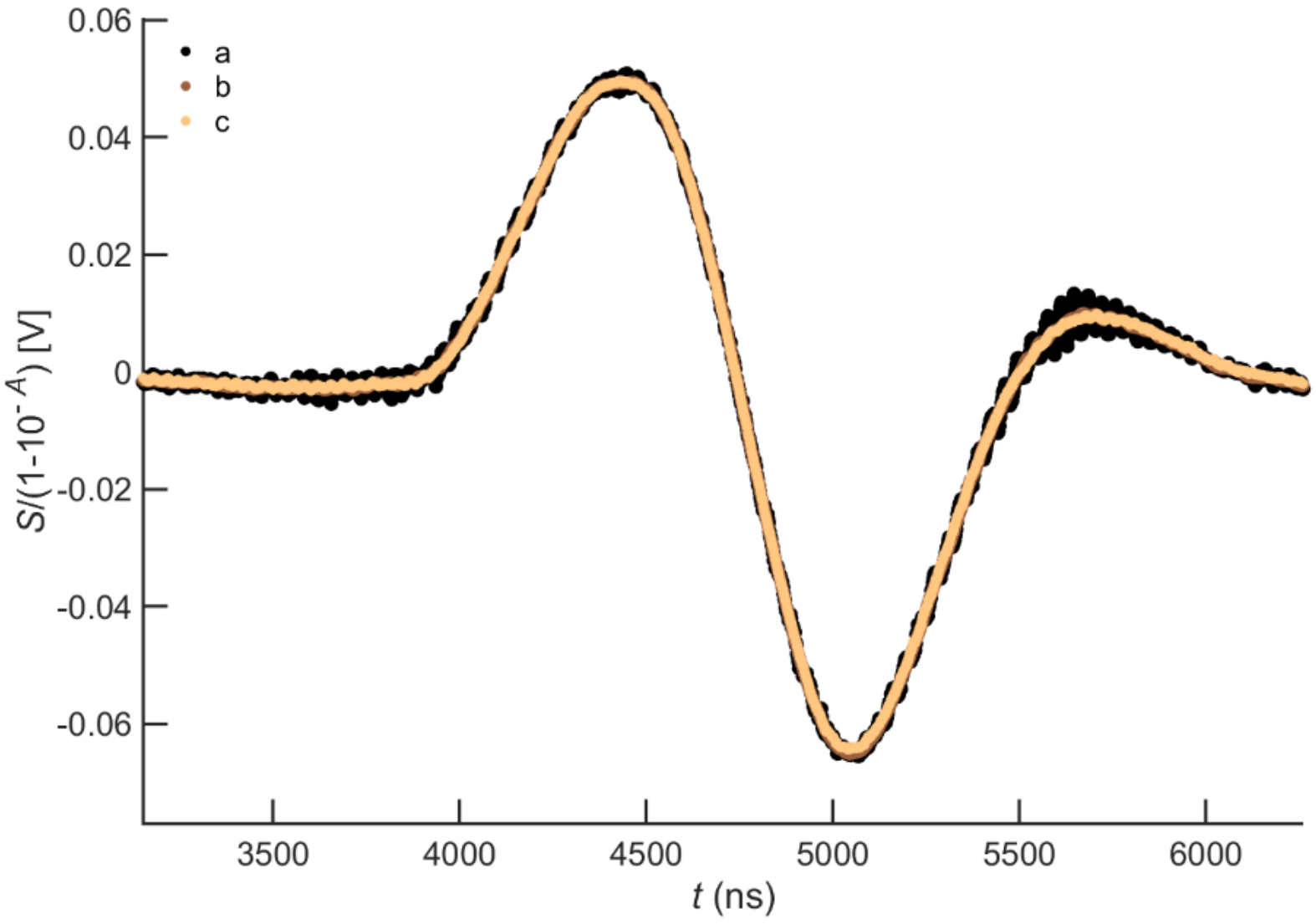

Figure S114: The normalized photoacoustic signal of NC in H_2O with a ND filter of OD 0.2 before the beam splitter at maximum laser intensity: (a) instrumentally averaged 5x; (b) the Savitsky-Golay smoothened curve of (a) with a window of 50 points and (c) the instrumentally averaged 60x. Laser pulse energy of the incident beam measured with the power sensor placed before the sample: (a) 1.9 mJ, (c) 1.9 mJ. Laser pulse energy of the transmitted beam measured with the power sensor placed after the sample: below the experimental uncertainty (*ca.* 0.5 mJ).

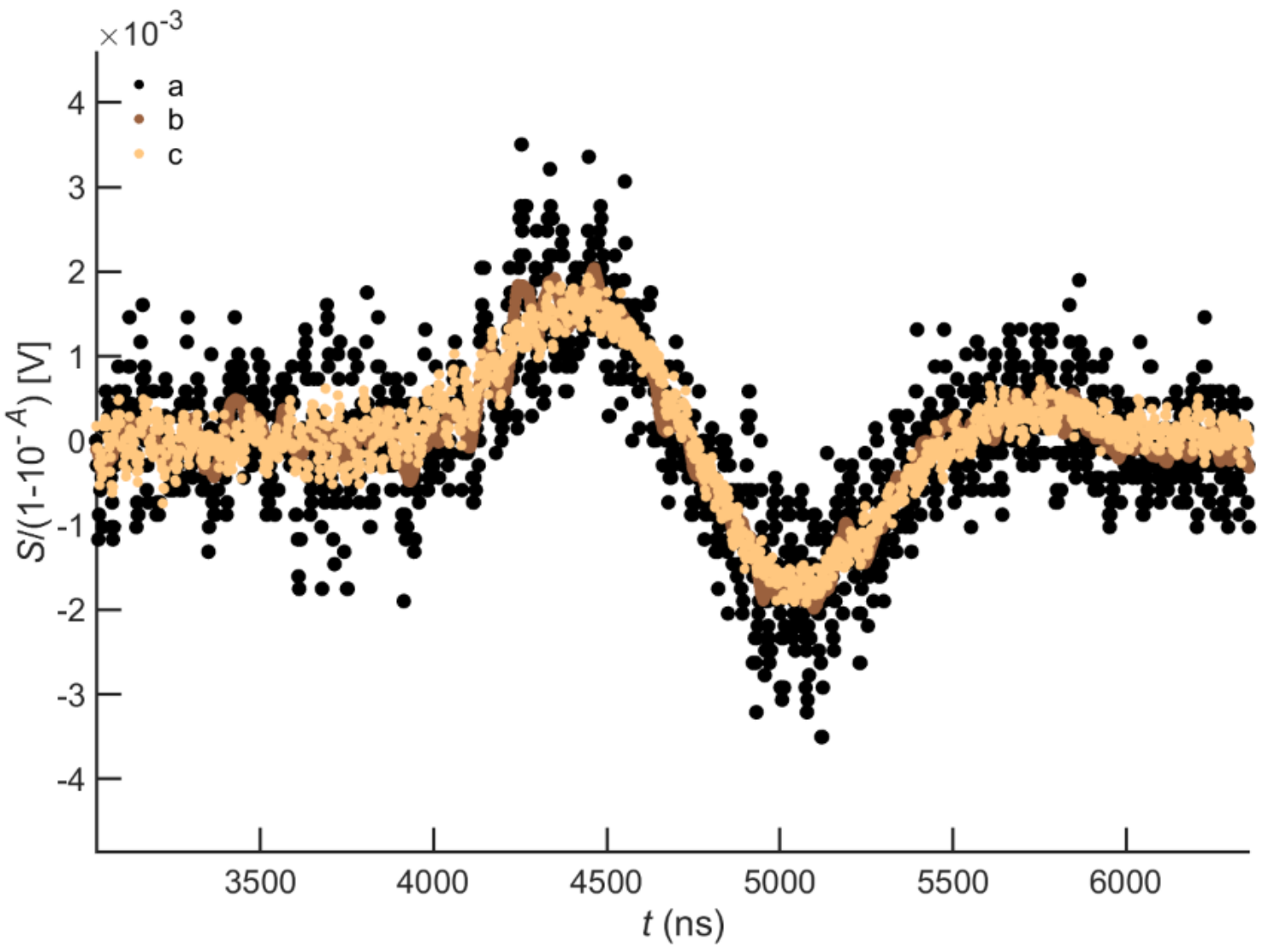

Figure S115: The normalized photoacoustic signal of NC in H_2O with a ND filter of OD 0.2 before the beam splitter at minimum laser intensity: (a) instrumentally averaged 5x; (b) the Savitsky-Golay smoothened curve of (a) with a window of 50 points and (c) the instrumentally averaged 60x. Laser pulse energy of the incident beam measured with the power sensor placed before the sample: (a) 0.25 mJ, (c)

0.25 mJ. Laser pulse energy of the transmitted beam measured with the power sensor placed after the sample: below the experimental uncertainty (*ca.* 0.5 mJ).

Section S9: Transient absorption spectroscopy data

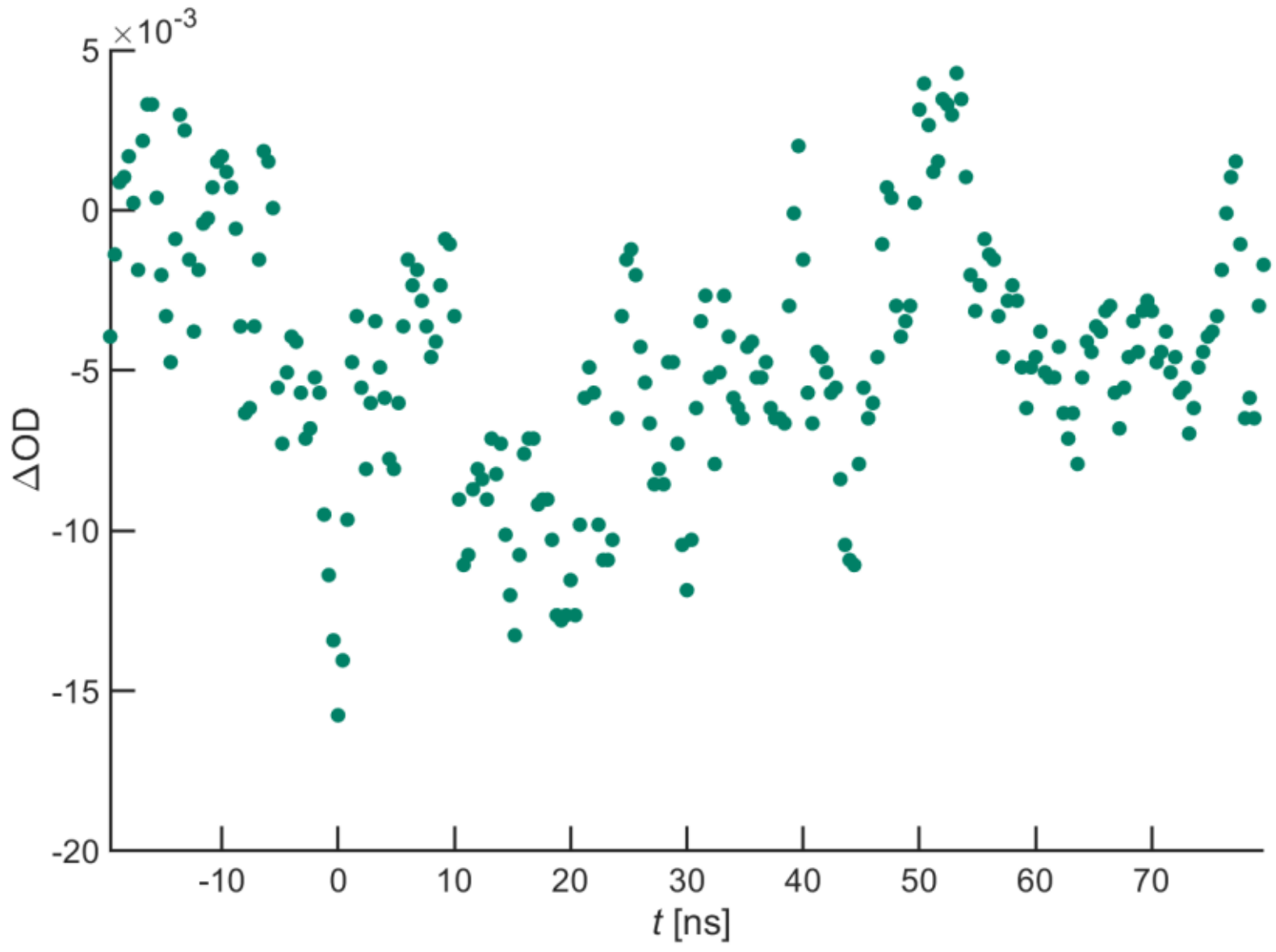

Figure S116: Oscillographic trace recorded of **NiPc-1** in DMSO with λ_{pump} = 640 nm (Q-band) and λ_{probe} = 355 nm (S-band) at the maximum laser excitation power, instrumentally averaged 20 times.

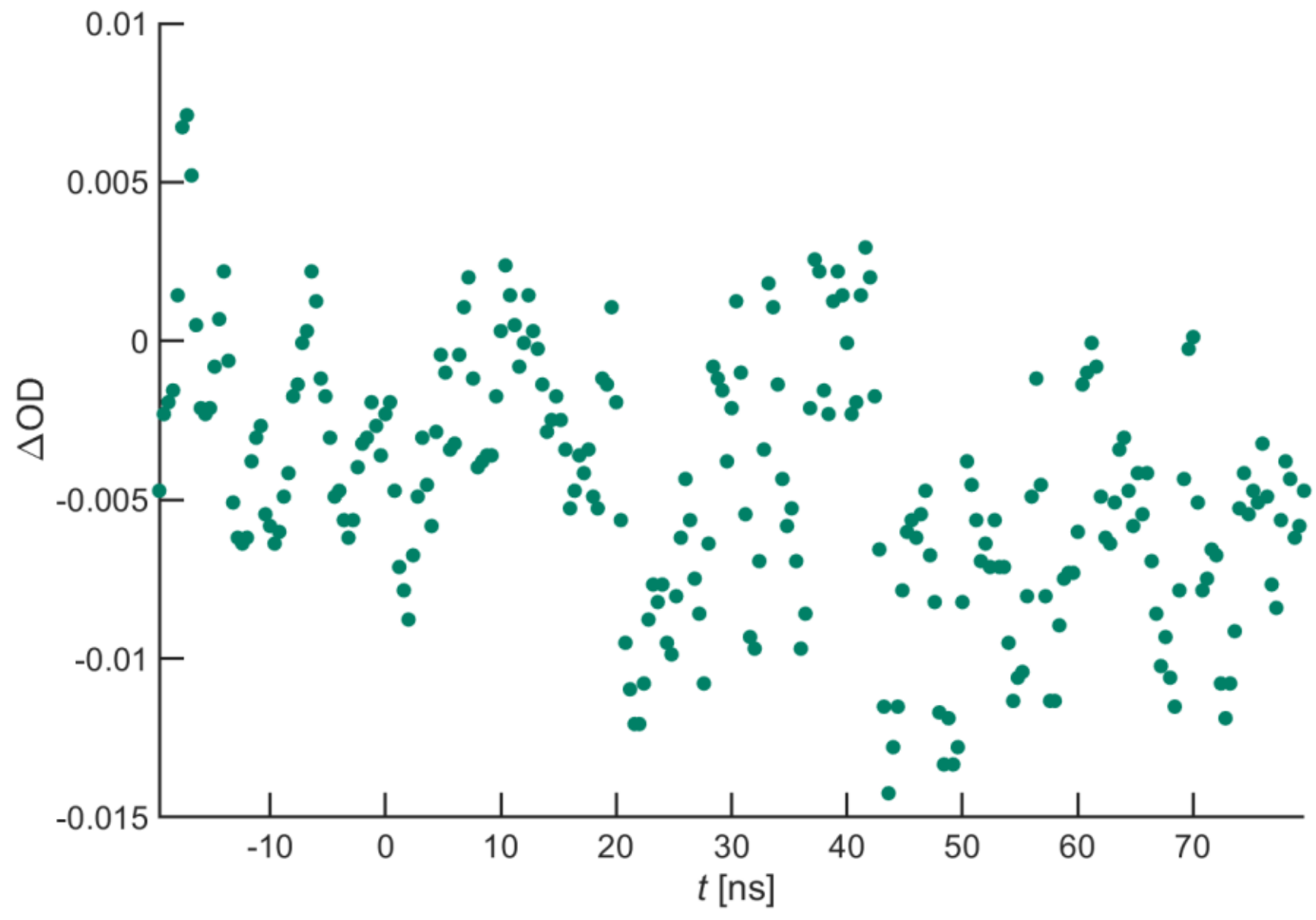

Figure S117: Oscillographic trace recorded of **NiPc-1** in DMSO with λ_{pump} = 640 nm (Q-band) and λ_{probe} = 670 nm (Q-band) at the maximum laser excitation power, instrumentally averaged 20 times.

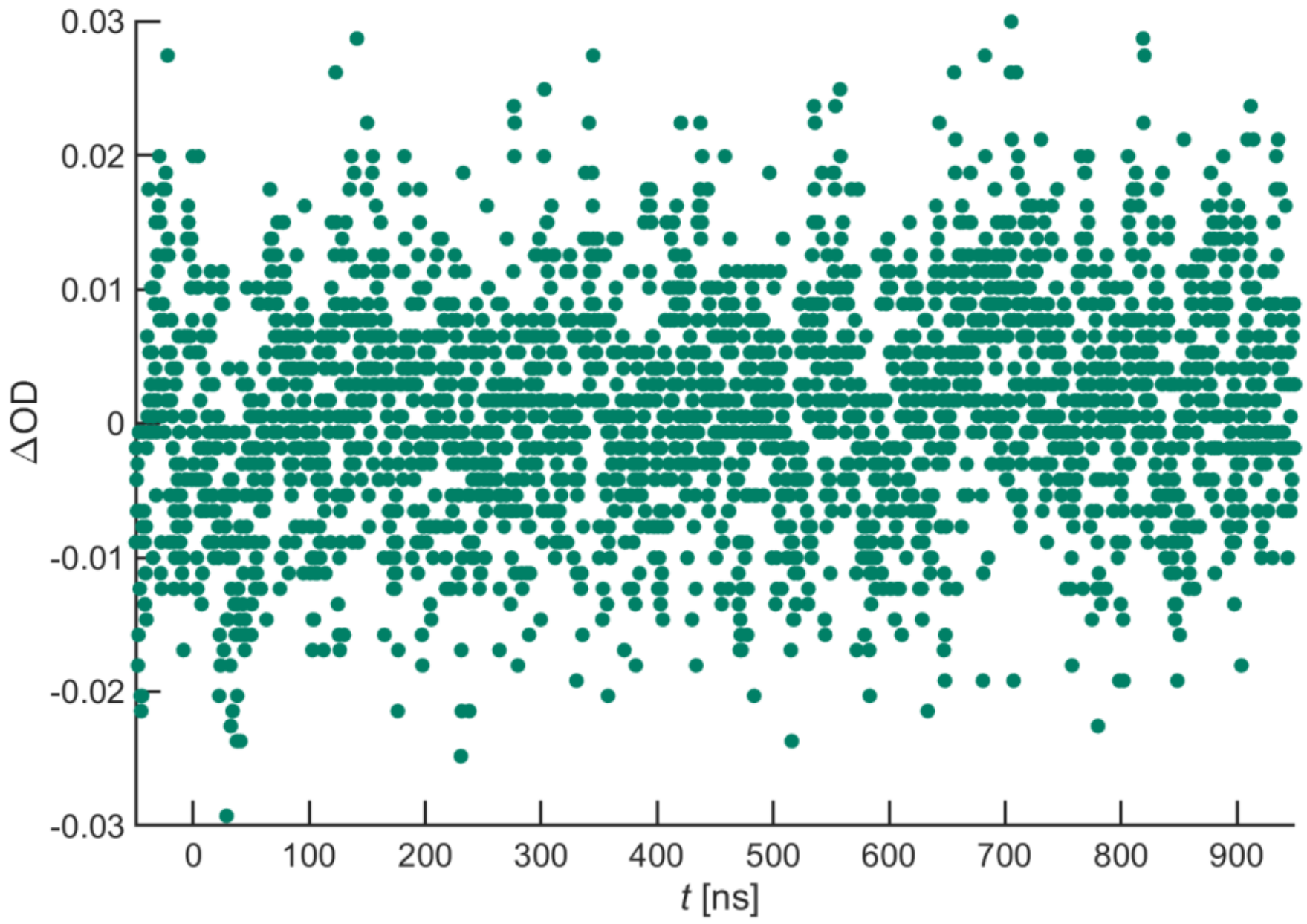

Figure S118: Oscillographic trace recorded of **NiPc-1** in DMSO with λ_{pump} = 355 nm (S-band) and λ_{probe} = 330 nm (S-band) at the maximum laser excitation power, instrumentally averaged 30 times.

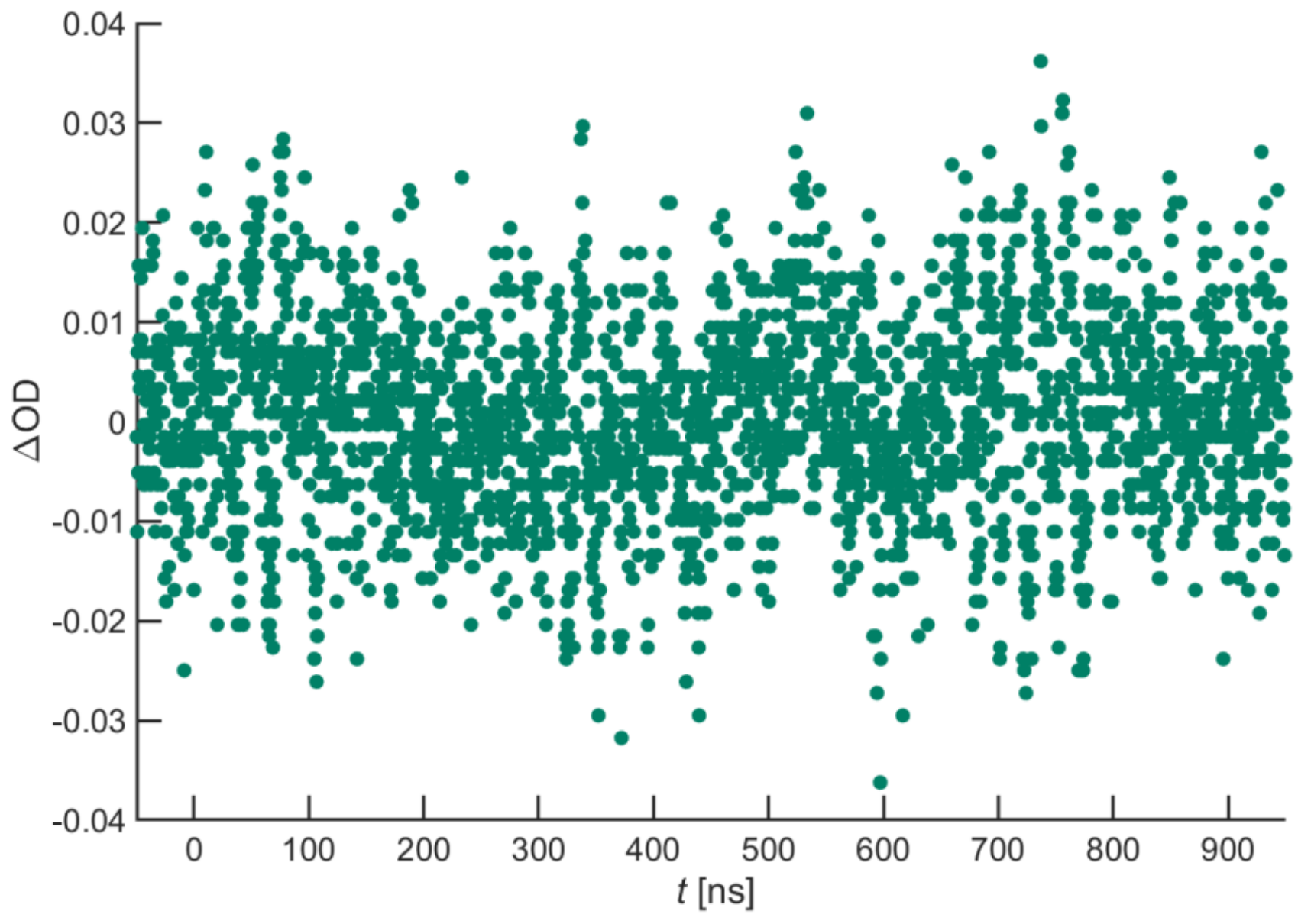

Figure S119: Oscillographic trace recorded of **NiPc-1** in DMSO with λ_{pump} = 355 nm (S-band) and λ_{probe} = 740 nm (Q-band) at the maximum laser excitation power, instrumentally averaged 30 times.

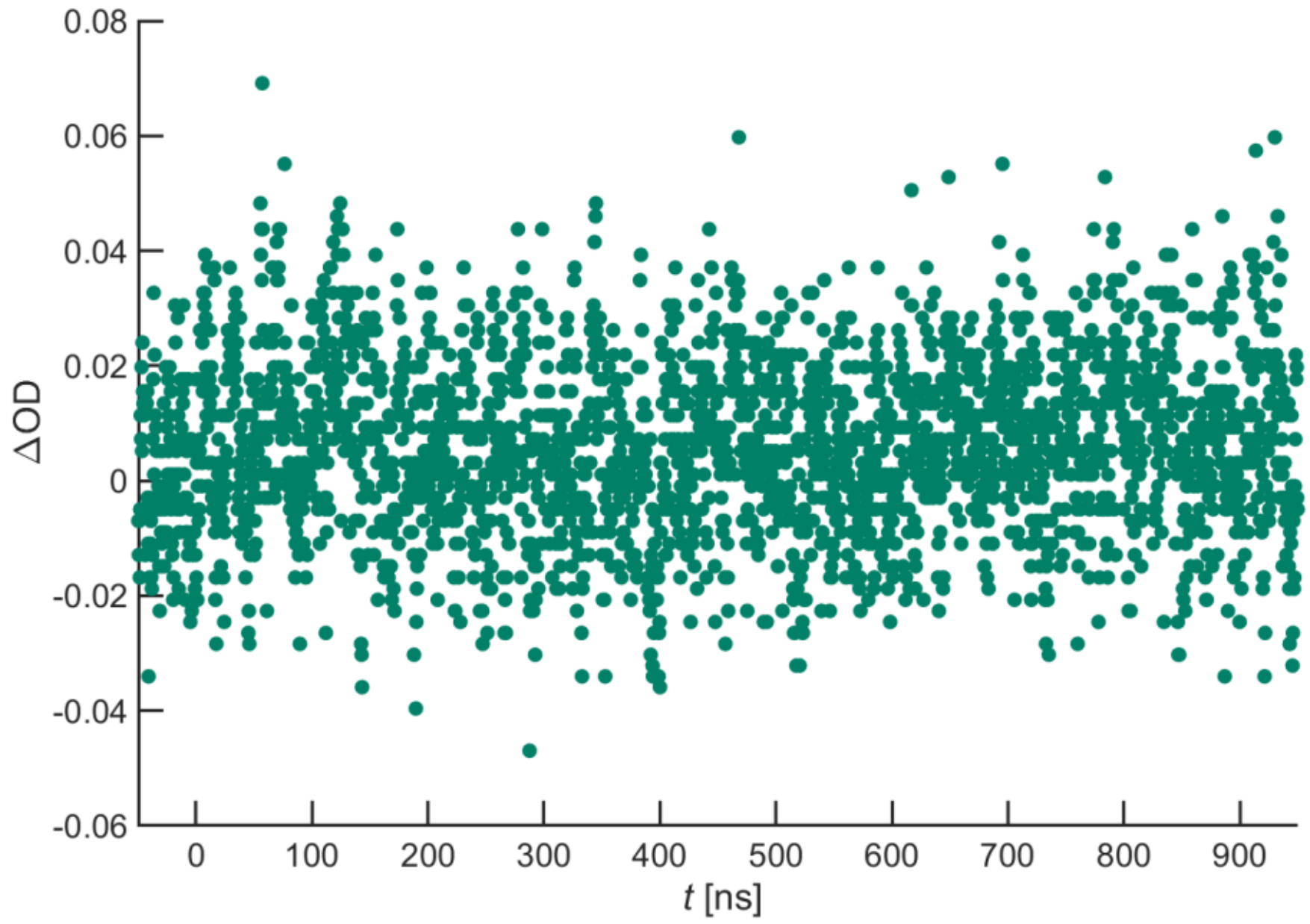

Figure S120: Oscillographic trace recorded of **NiPc-2** in DMSO with λ_{pump} = 355 nm (S-band) and λ_{probe} = 330 nm (S-band) at the maximum laser excitation power, instrumentally averaged 30 times.

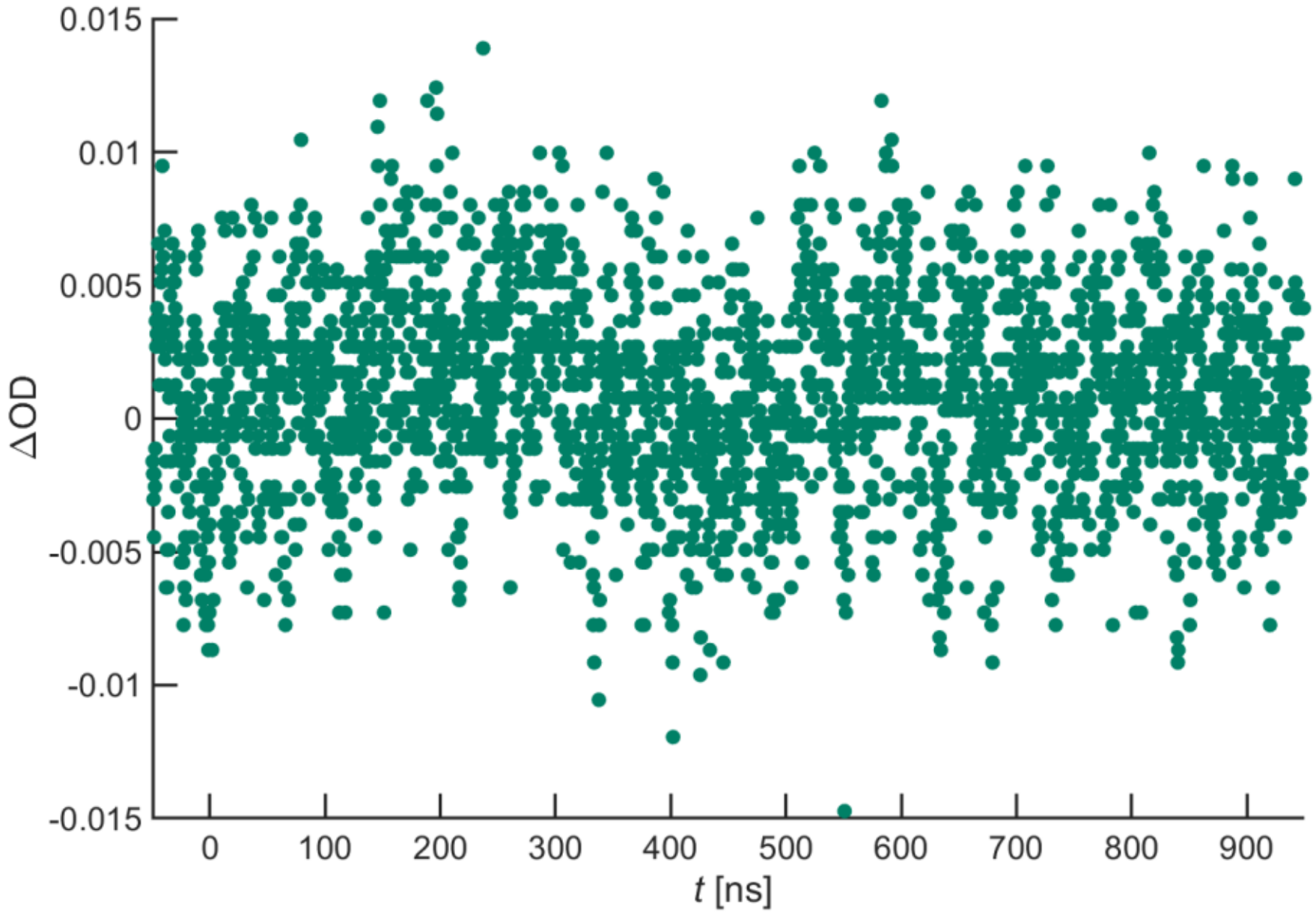

Figure S121: Oscillographic trace recorded of **NiPc-2** in DMSO with λ_{pump} = 355 nm (S-band) and λ_{probe} = 700 nm (Q-band) at the maximum laser excitation power, instrumentally averaged 30 times.

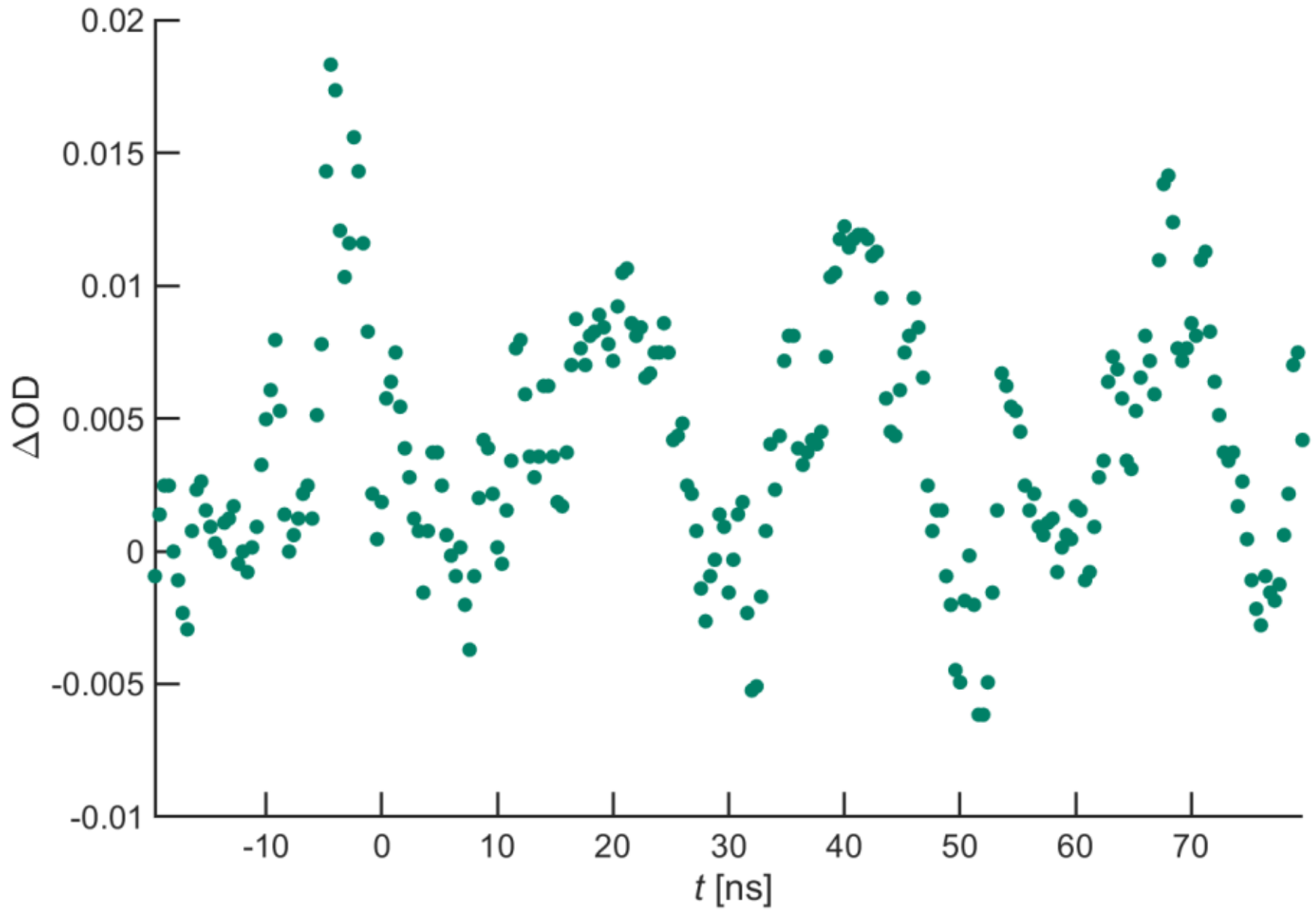

Figure S122: Oscillographic trace recorded of **NiPc-2** in DMSO with λ_{pump} = 590 nm (Q-band) and λ_{probe} = 355 nm (S-band) at the maximum laser excitation power, instrumentally averaged 20 times.

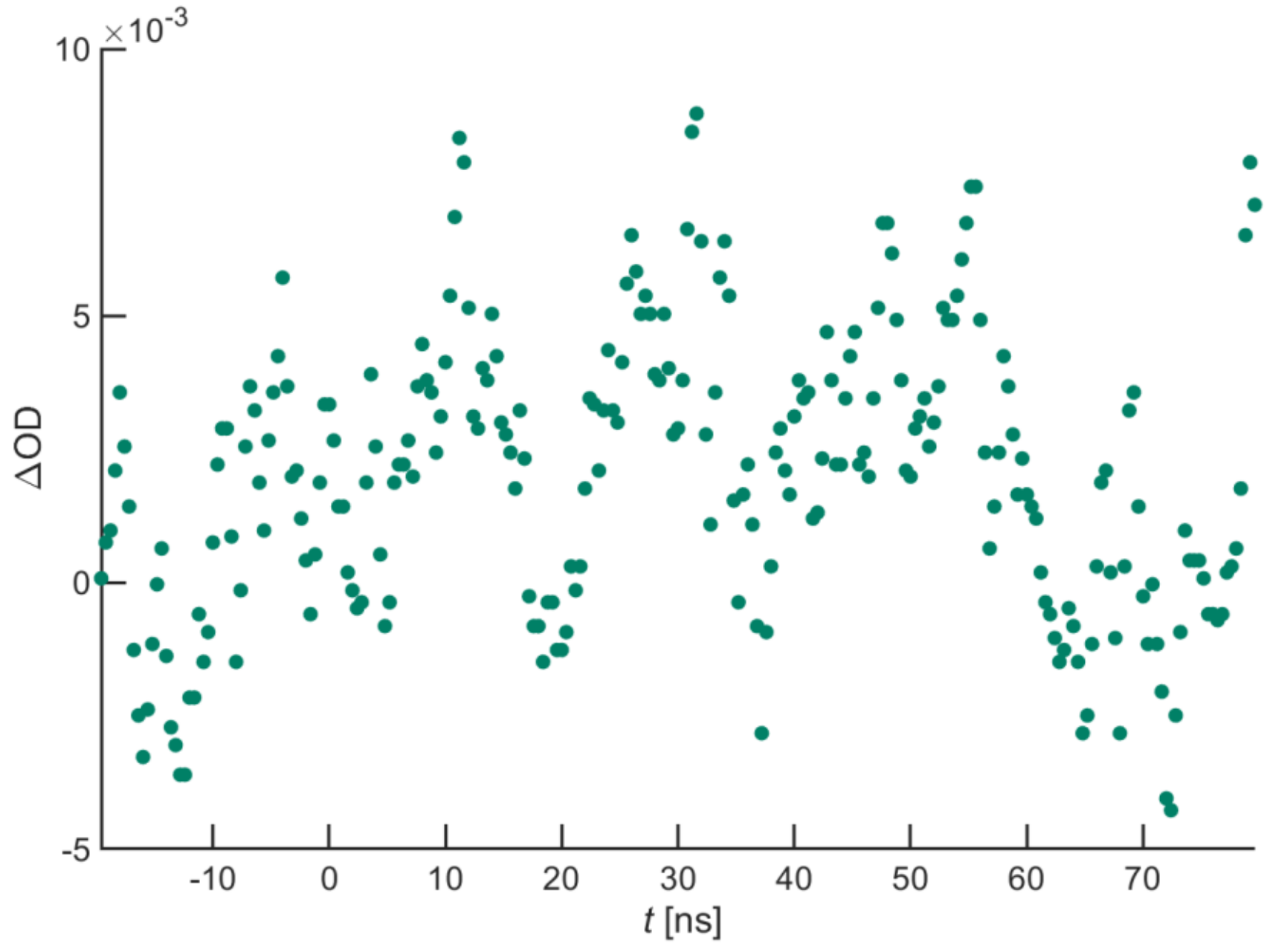

Figure S123: Oscillographic trace recorded of **NiPc-2** in DMSO with λ_{pump} = 590 nm (Q-band) and λ_{probe} = 620 nm (Q-band) at the maximum laser excitation power, instrumentally averaged 20 times.

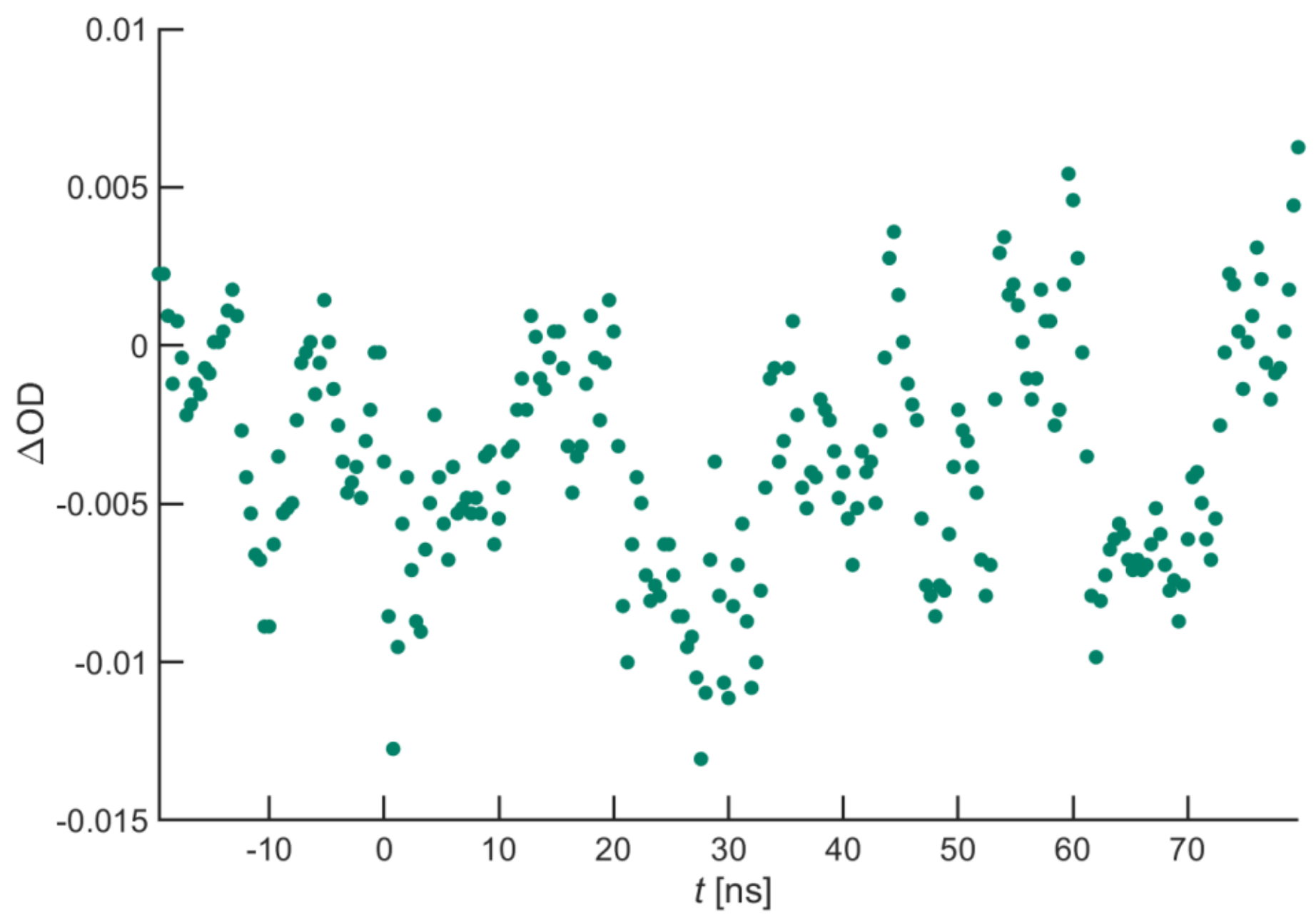

Figure S124: Oscillographic trace recorded of **NiPc-3** in DMSO with λ_{pump} = 690 nm (Q-band) and λ_{probe} = 355 nm (S-band) at the maximum laser excitation power, instrumentally averaged 20 times.

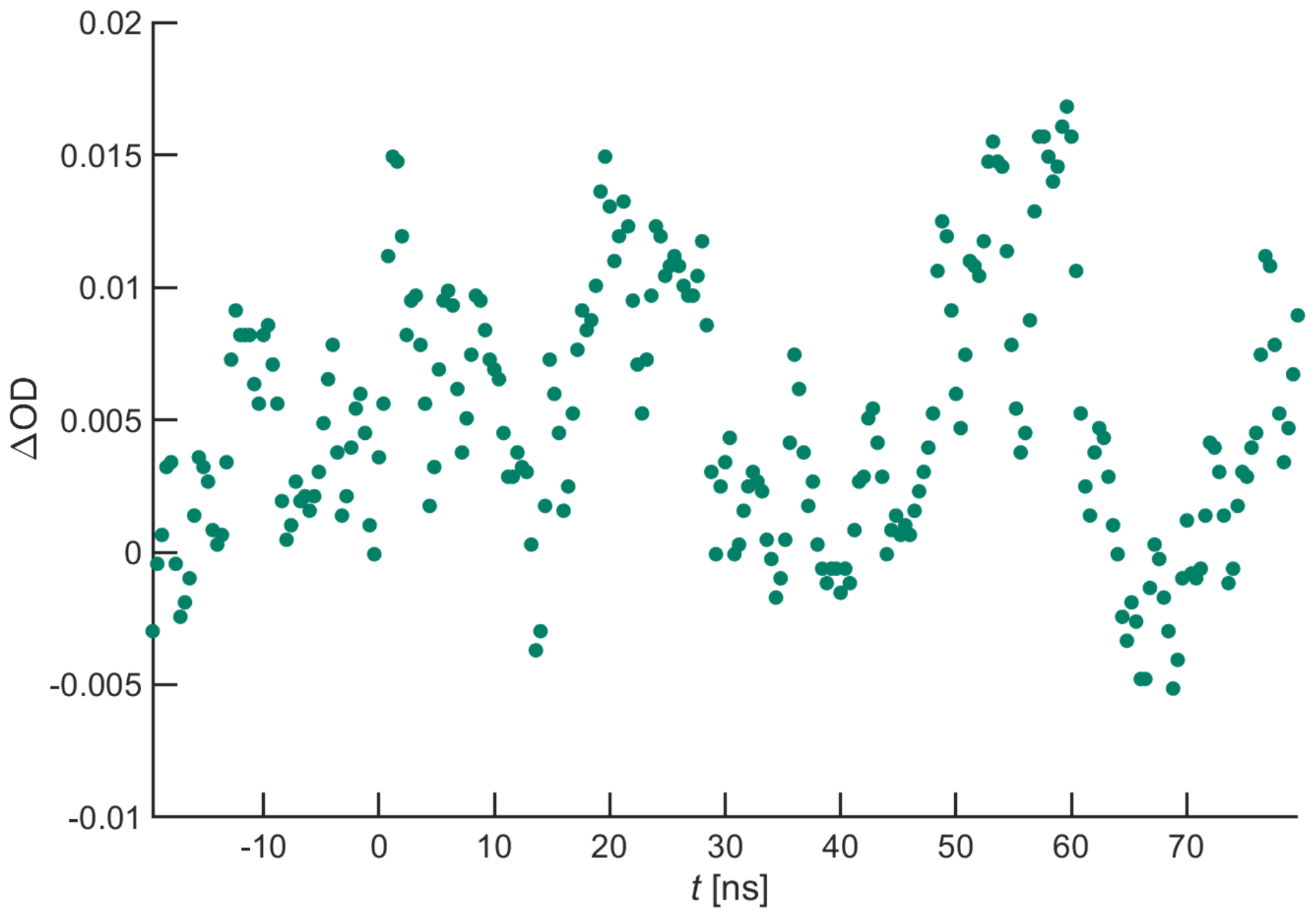

Figure S125: Oscillographic trace recorded of **NiPc-3** in DMSO with λ_{pump} = 690 nm (Q-band) and λ_{probe} = 700 nm (Q-band) at the maximum laser excitation power, instrumentally averaged 20 times.

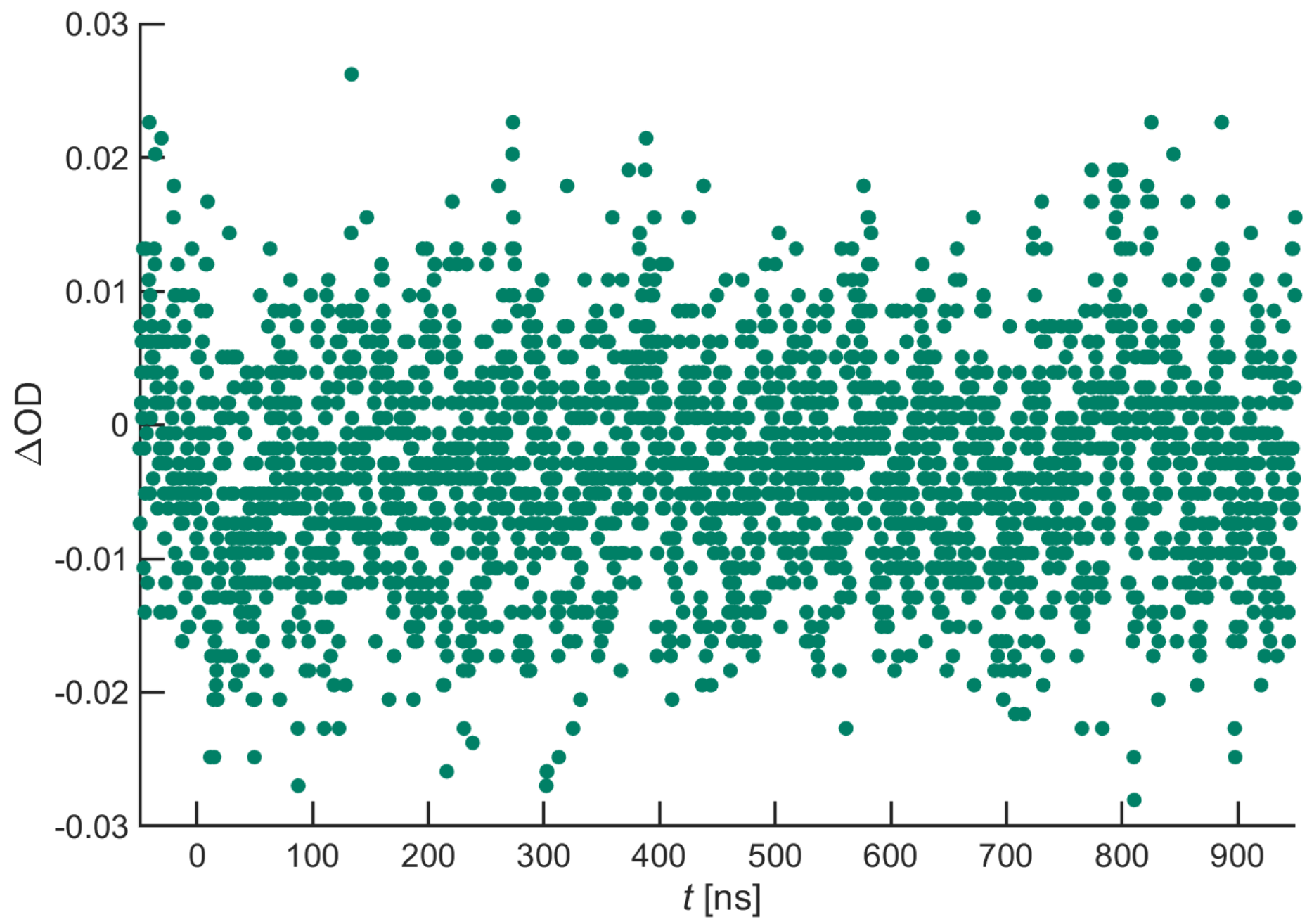

Figure S126: Oscillographic trace recorded of **NiPc-3** in DMSO with λ_{pump} = 355 nm (S-band) and λ_{probe} = 330 nm (S-band) at the maximum laser excitation power, instrumentally averaged 30 times.

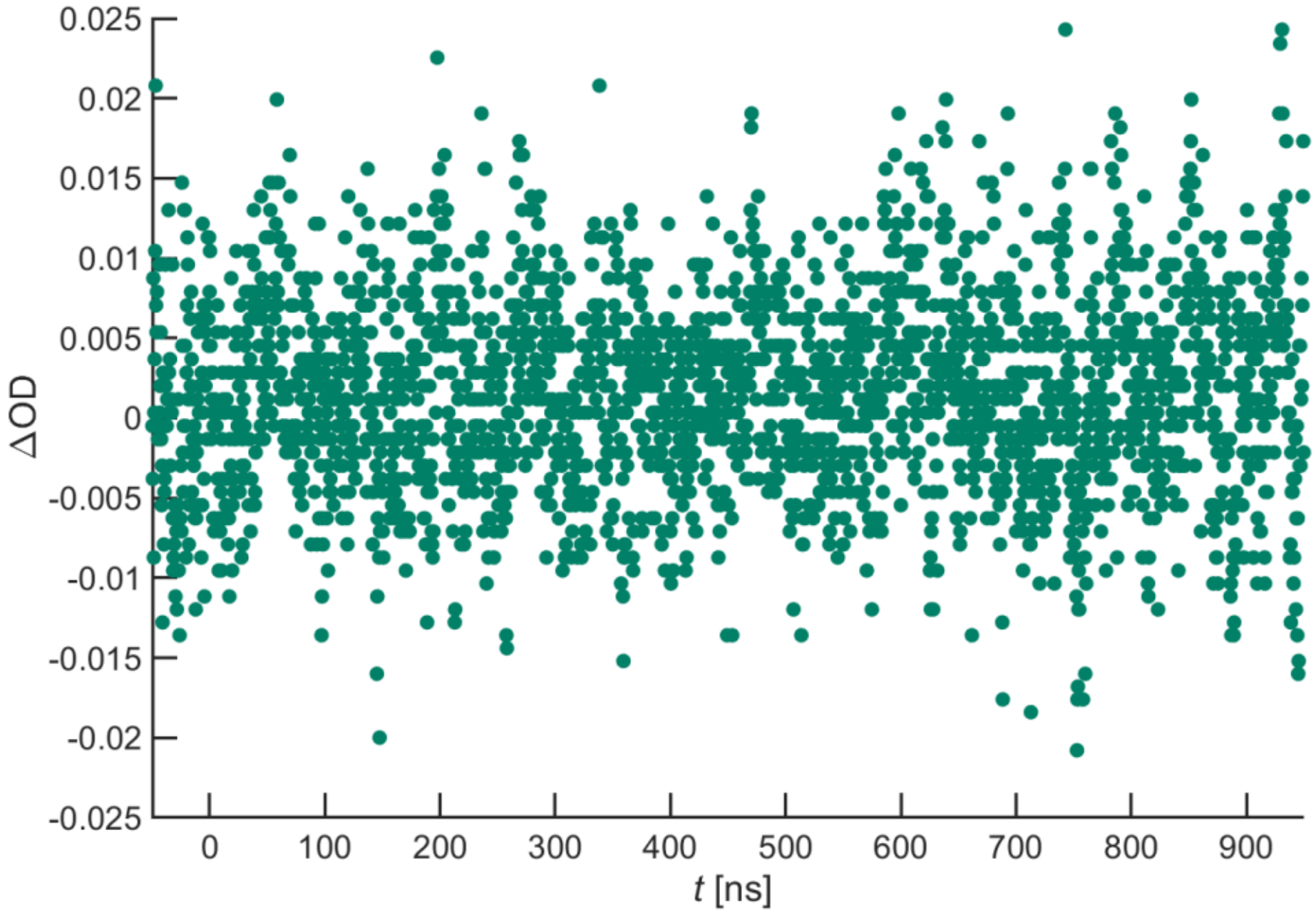

Figure S127: Oscillographic trace recorded of **NiPc-3** in DMSO with λ_{pump} = 355 nm (S-band) and λ_{probe} = 750 nm (Q-band) at the maximum laser excitation power, instrumentally averaged 30 times.

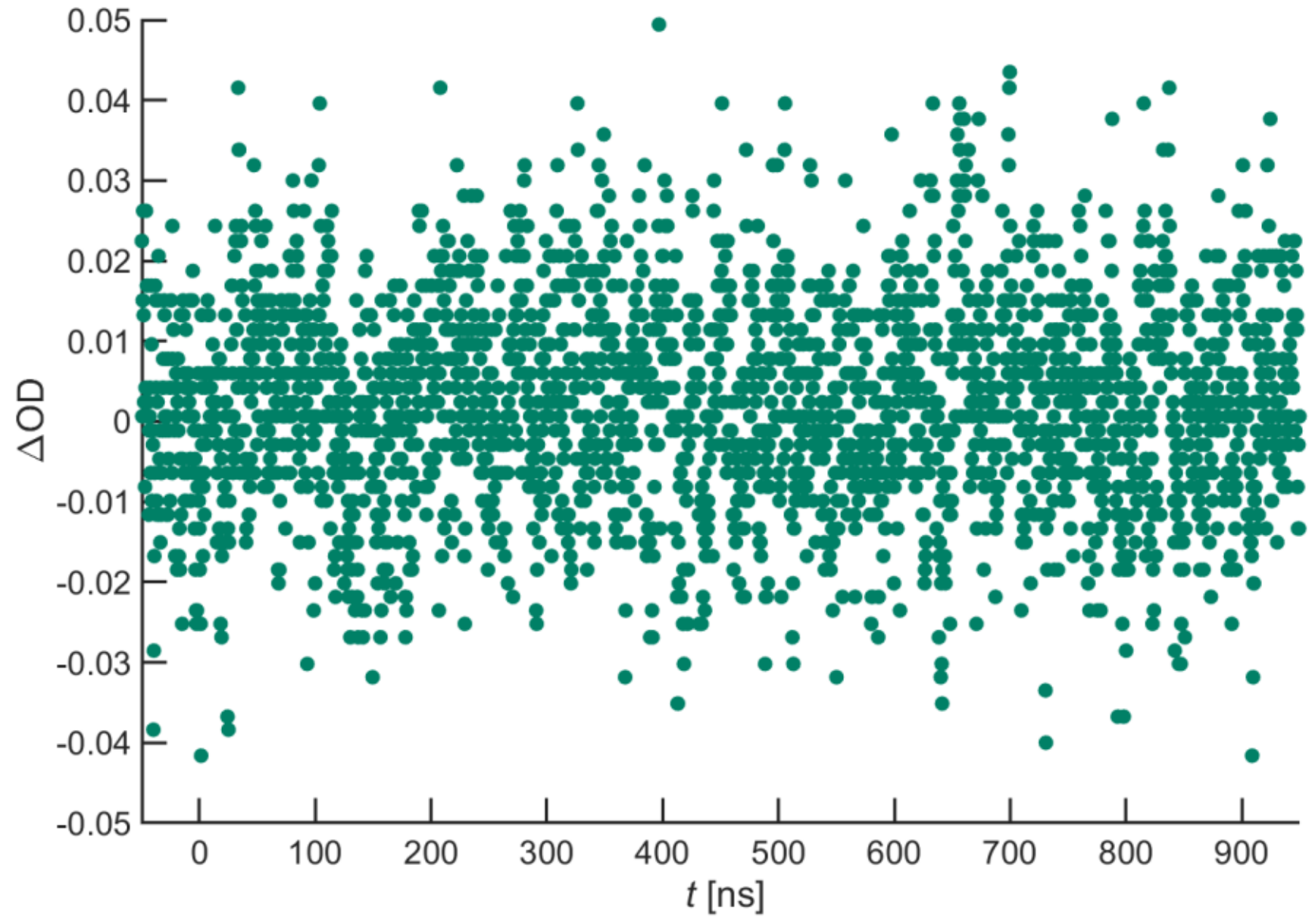

Figure S128: Oscillographic trace recorded of **NiPc-4** in DMSO with λ_{pump} = 355 nm (S-band) and λ_{probe} = 330 nm (S-band) at the maximum laser excitation power, instrumentally averaged 30 times.

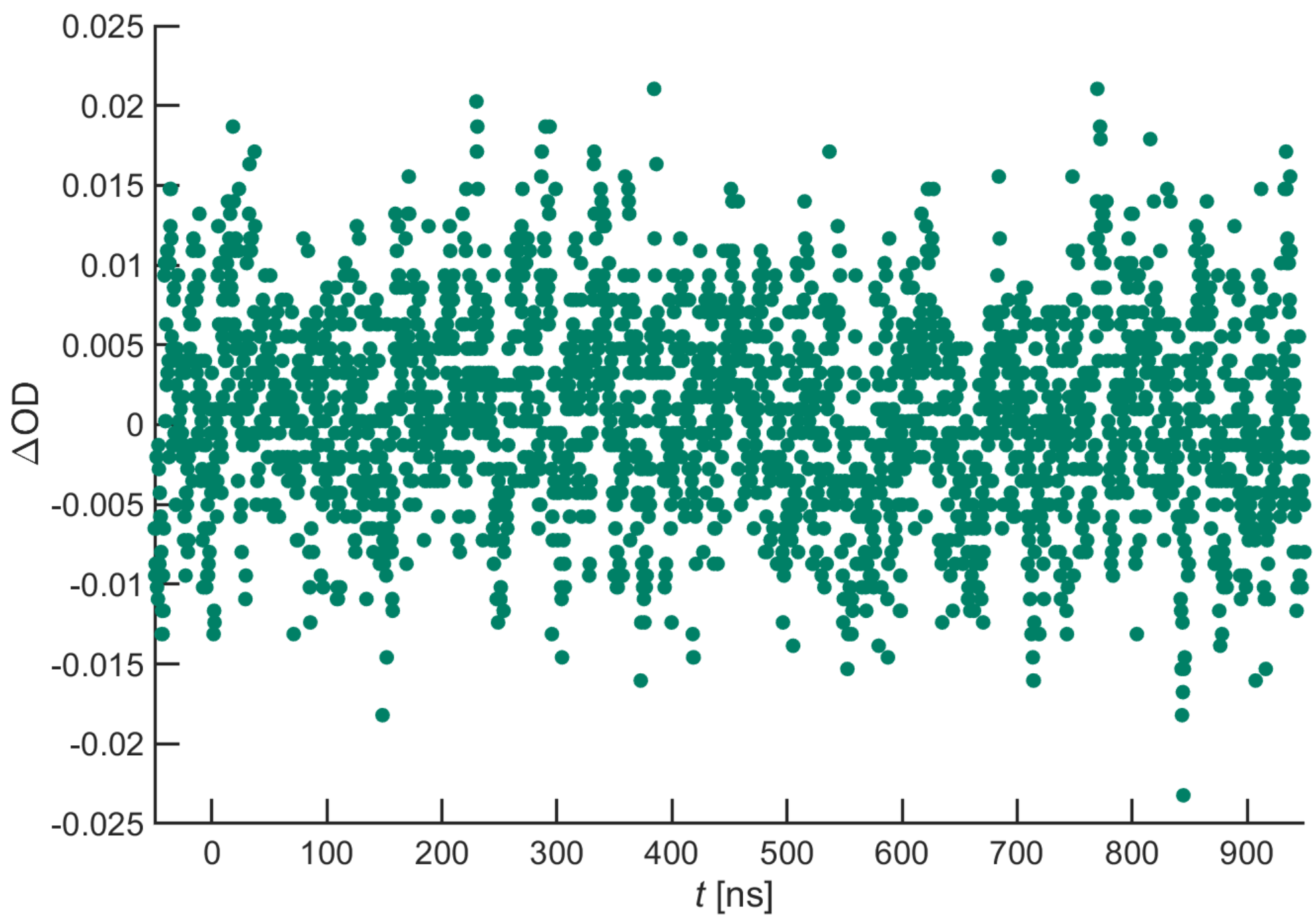

Figure S129: Oscillographic trace recorded of **NiPc-4** in DMSO with λ_{pump} = 355 nm (S-band) and λ_{probe} = 705 nm (Q-band) at the maximum laser excitation power, instrumentally averaged 30 times.

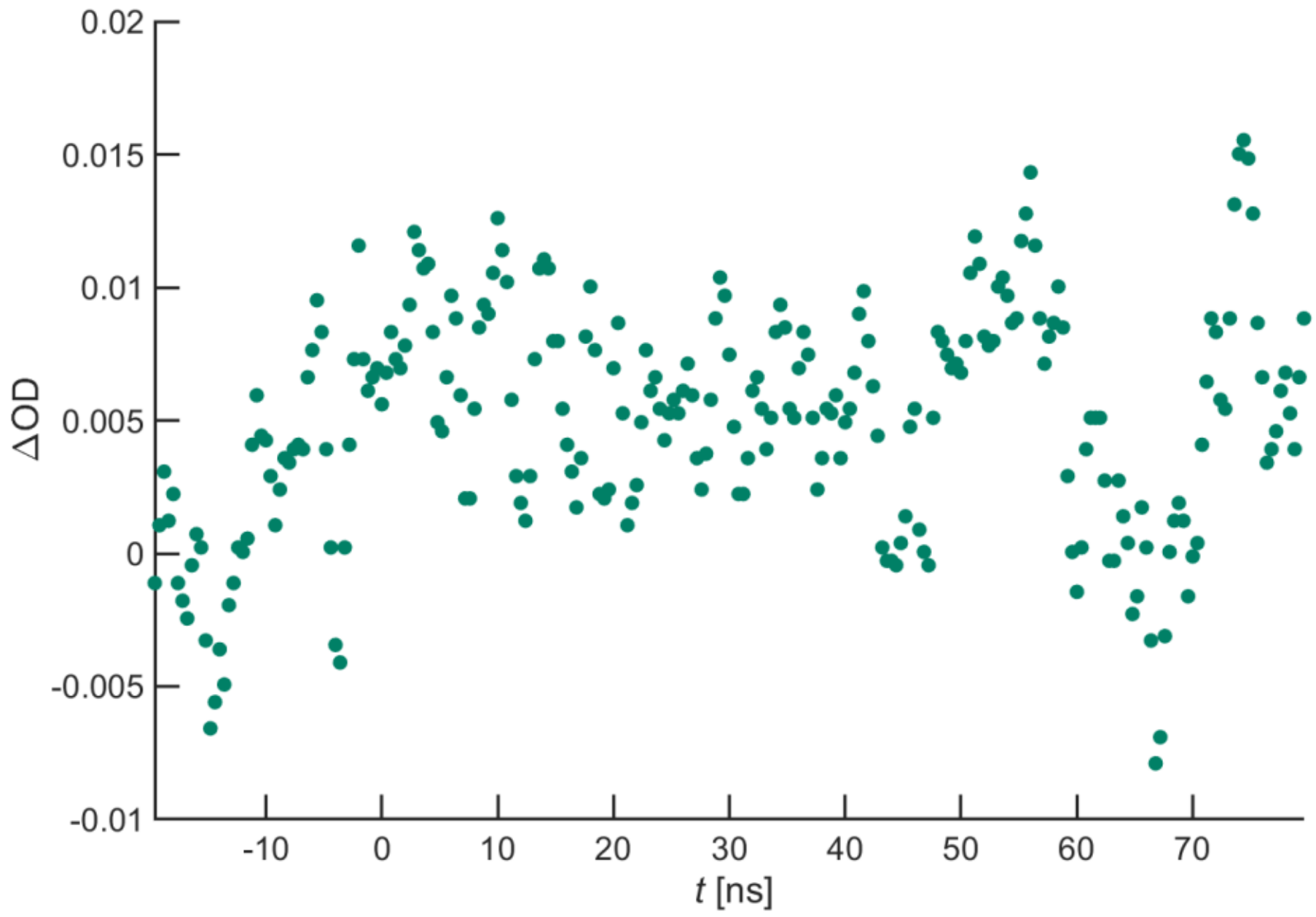

Figure S130: Oscillographic trace recorded of **NiPc-4** in DMSO with λ_{pump} = 620 nm (Q-band) and λ_{probe} = 355 nm (S-band) at the maximum laser excitation power, instrumentally averaged 20 times.

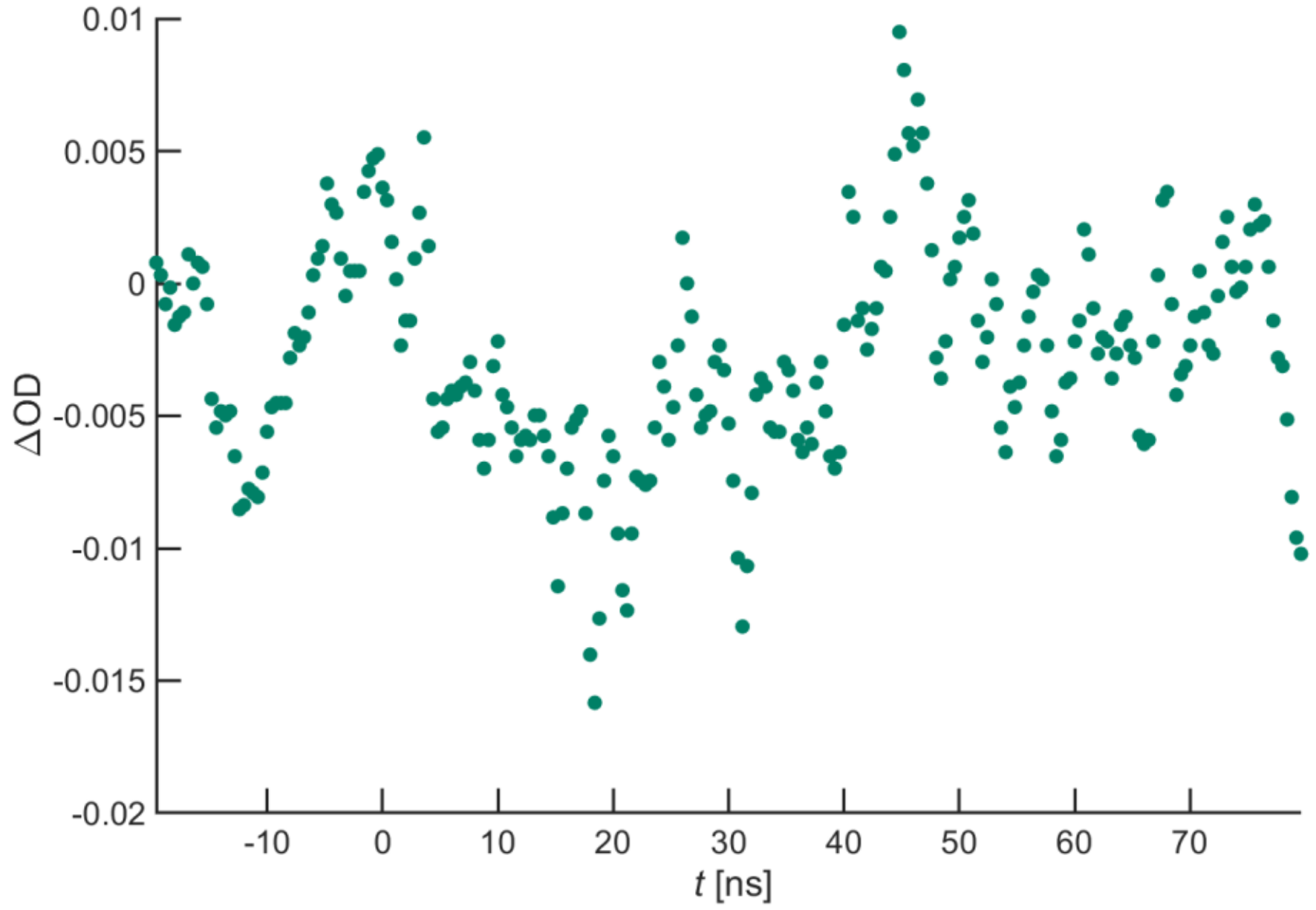

Figure S131: Oscillographic trace recorded of **NiPc-4** in DMSO with λ_{pump} = 620 nm (Q-band) and λ_{probe} = 650 nm (Q-band) at the maximum laser excitation power, instrumentally averaged 30 times.

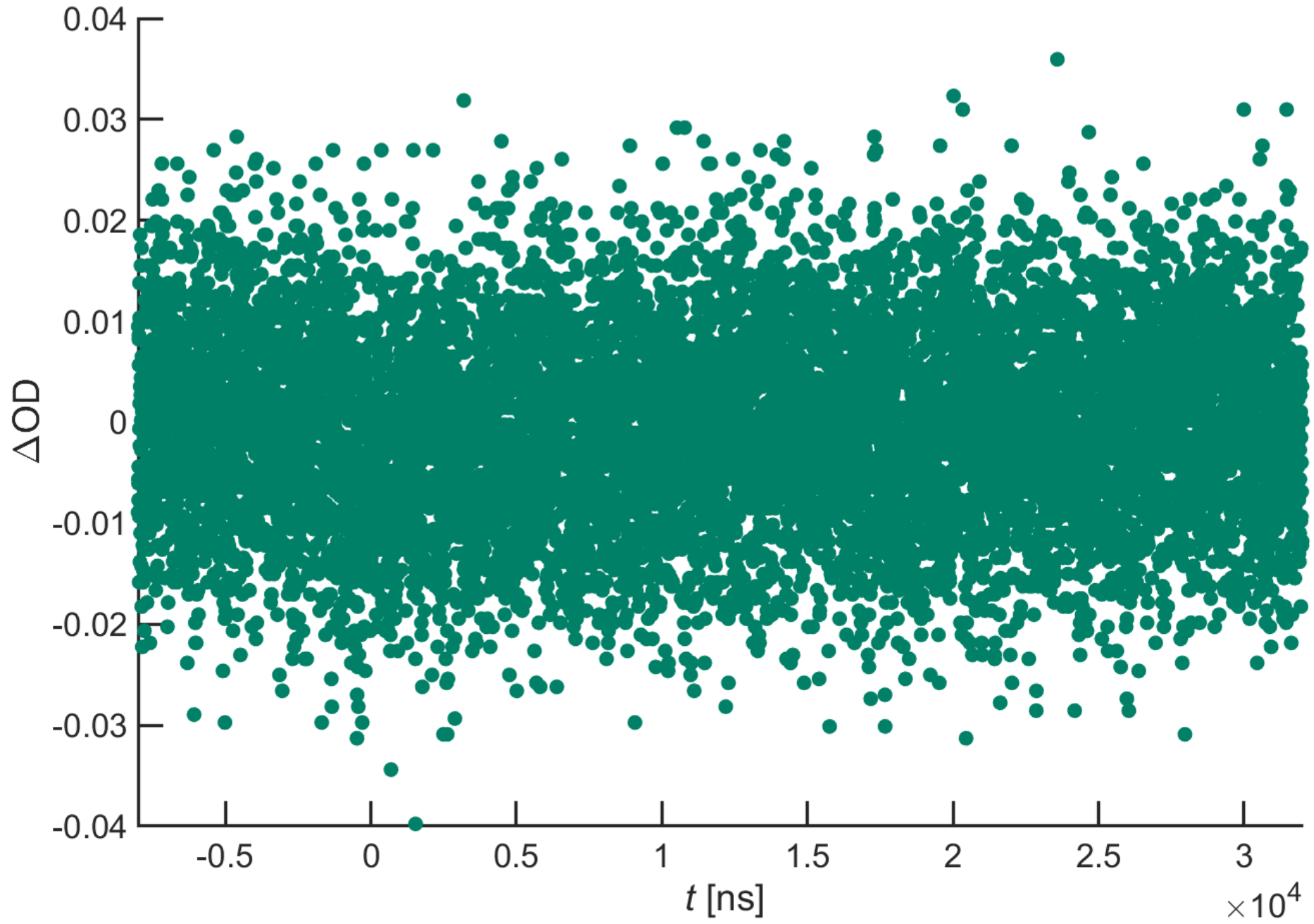

Figure S132: Oscillographic trace recorded of **NiPc-1** in H_2O with λ_{pump} = 355 nm (S-band) and λ_{probe} = 330 nm (S-band) at the maximum laser excitation power, instrumentally averaged 20 times.

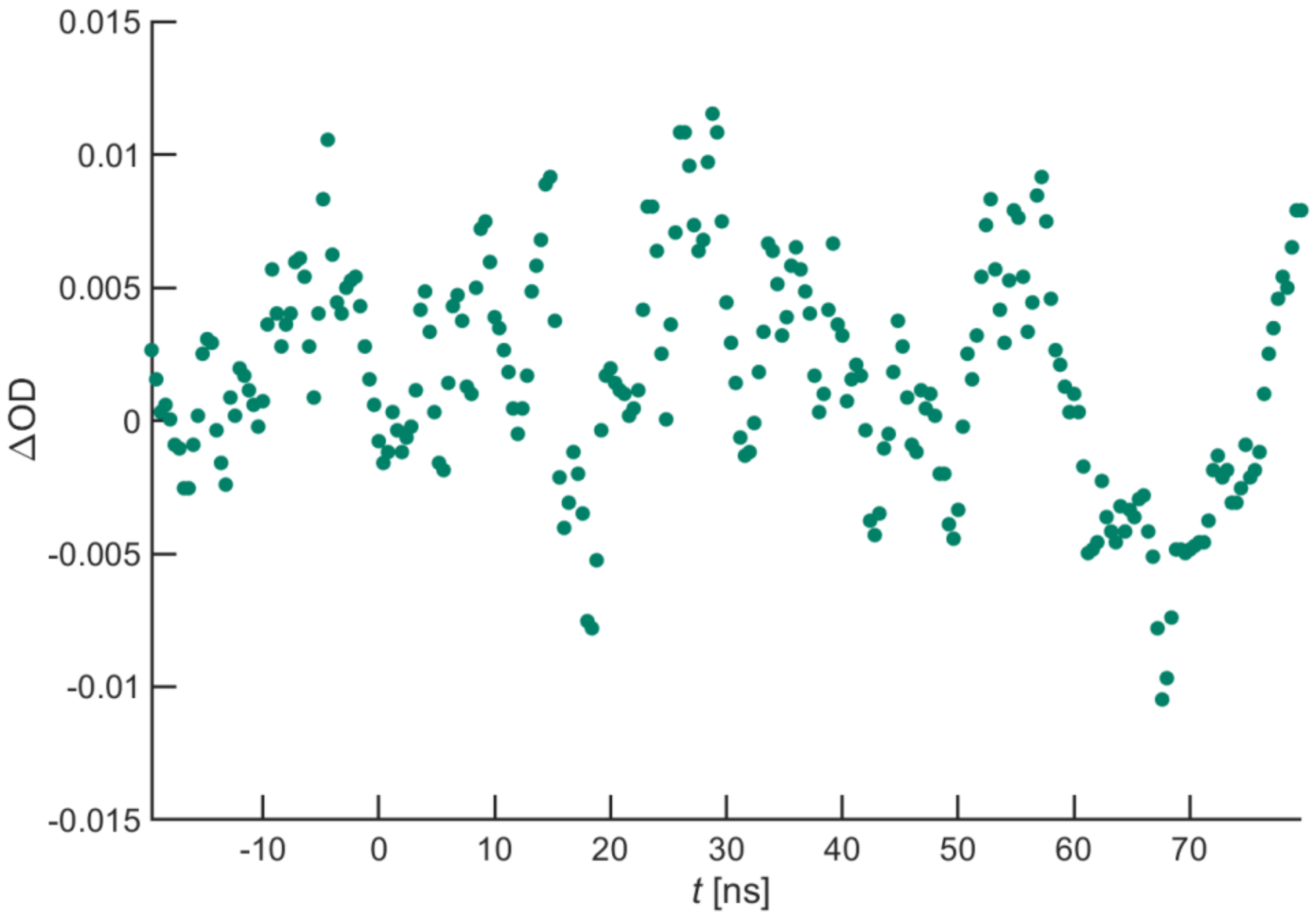

Figure S133: Oscillographic trace recorded of **NiPc-1** in H_2O with λ_{pump} = 355 nm (S-band) and λ_{probe} = 645 nm (Q-band) at the maximum laser excitation power, instrumentally averaged 20 times.

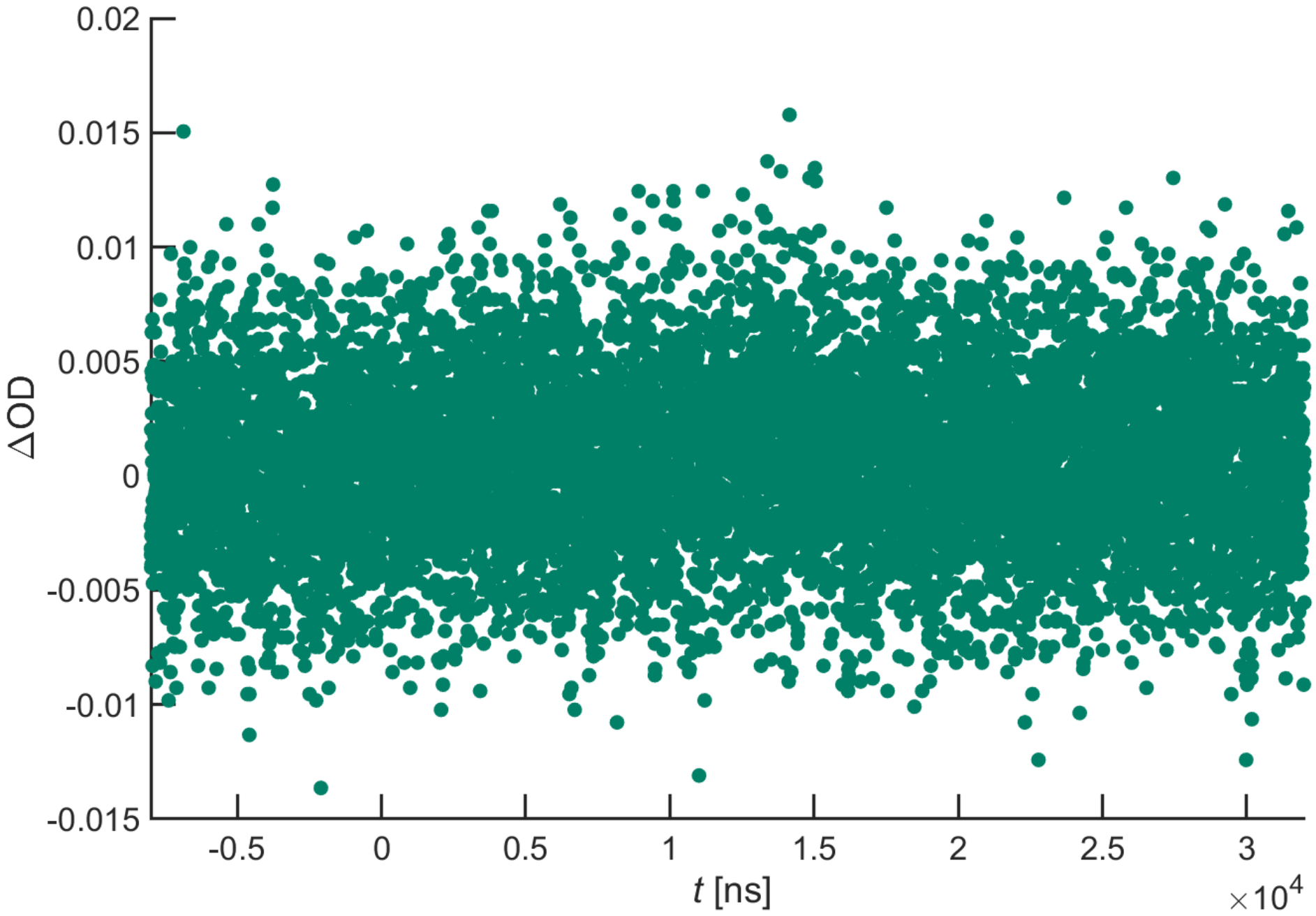

Figure S134: Oscillographic trace recorded of **NiPc-1** in H_2O with λ_{pump} = 645 nm (Q-band) and λ_{probe} = 355 nm (S-band) at the maximum laser excitation power, instrumentally averaged 20 times.

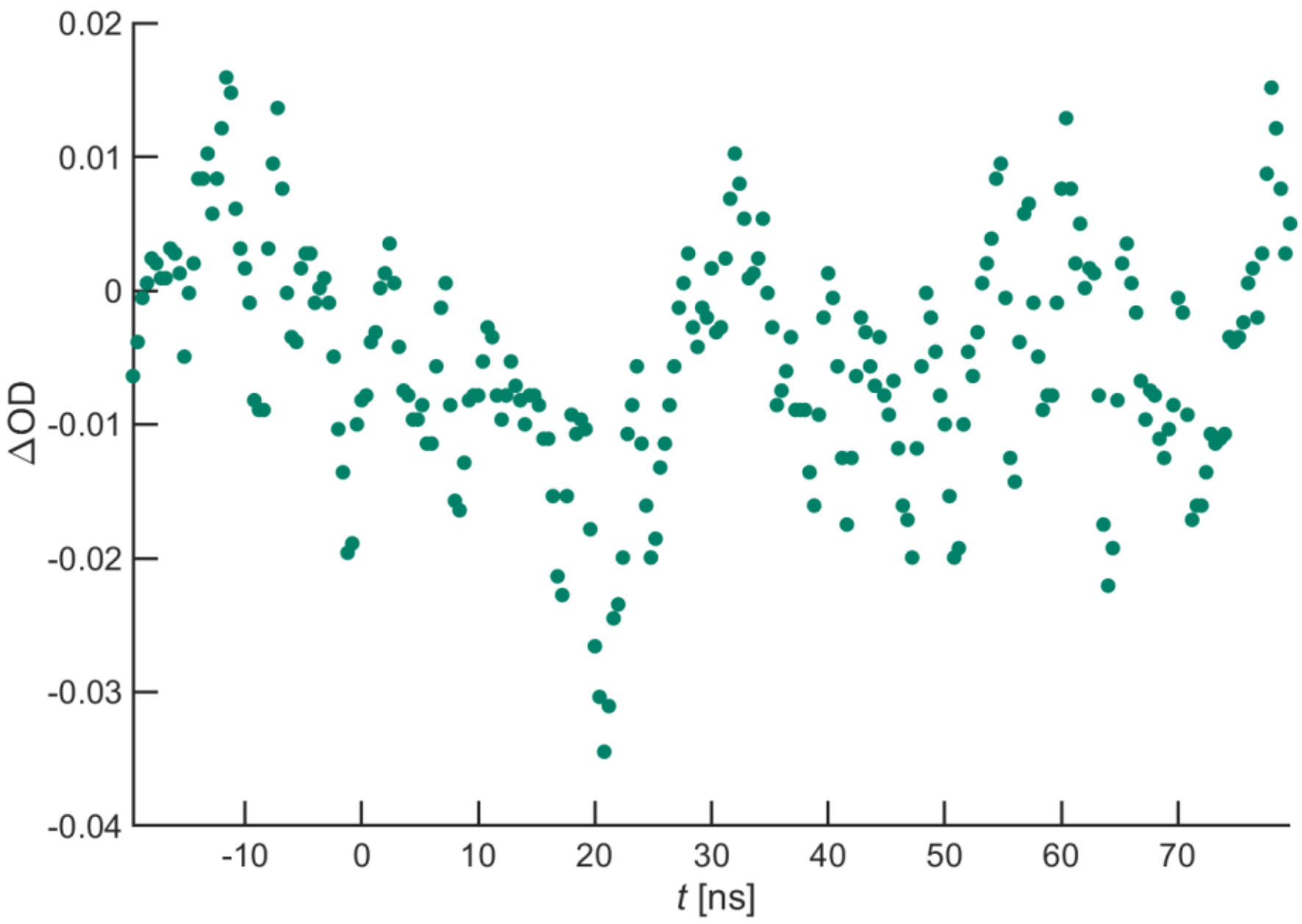

Figure S135: Oscillographic trace recorded of **NiPc-1** in H_2O with λ_{pump} = 645 nm (Q-band) and λ_{probe} = 720 nm (Q-band) at the maximum laser excitation power, instrumentally averaged 20 times.

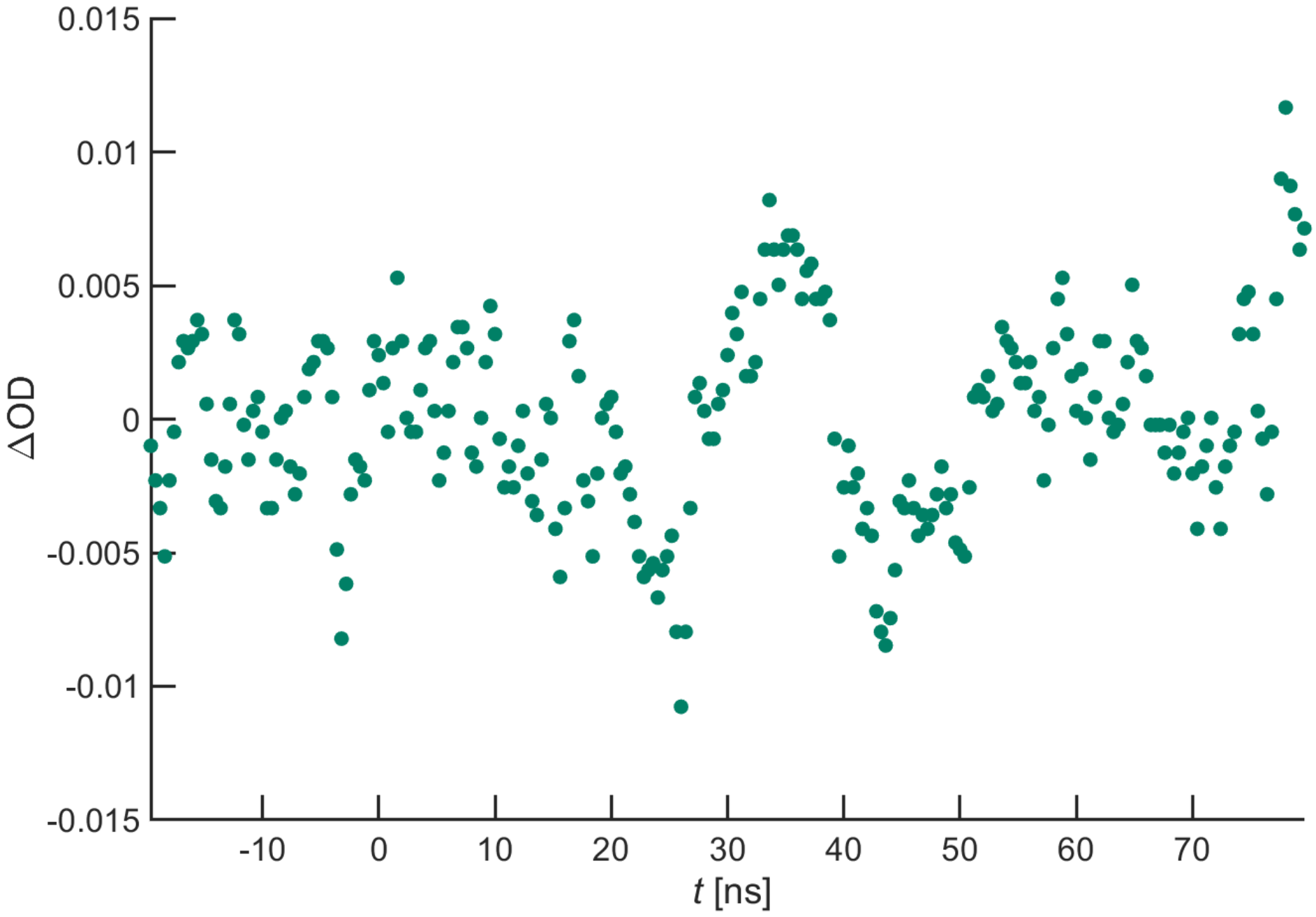

Figure S136: Oscillographic trace recorded of **NiPc-2** in H_2O with λ_{pump} = 650 nm (Q-band) and λ_{probe} = 355 nm (S-band) at the maximum laser excitation power, instrumentally averaged 20 times.

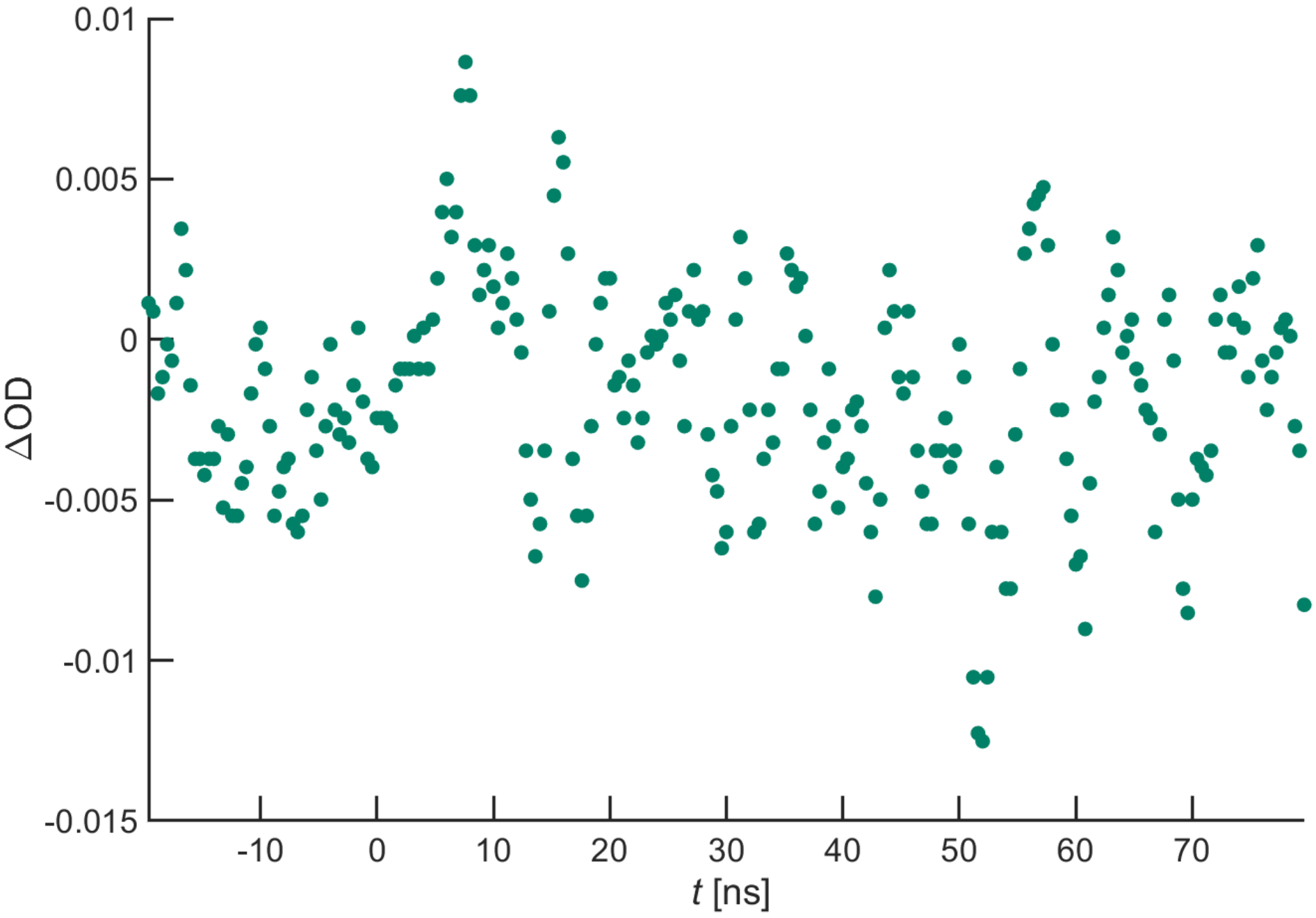

Figure S137: Oscillographic trace recorded of **NiPc-2** in H_2O with λ_{pump} = 650 nm (Q-band) and λ_{probe} = 600 nm (Q-band) at the maximum laser excitation power, instrumentally averaged 10 times.

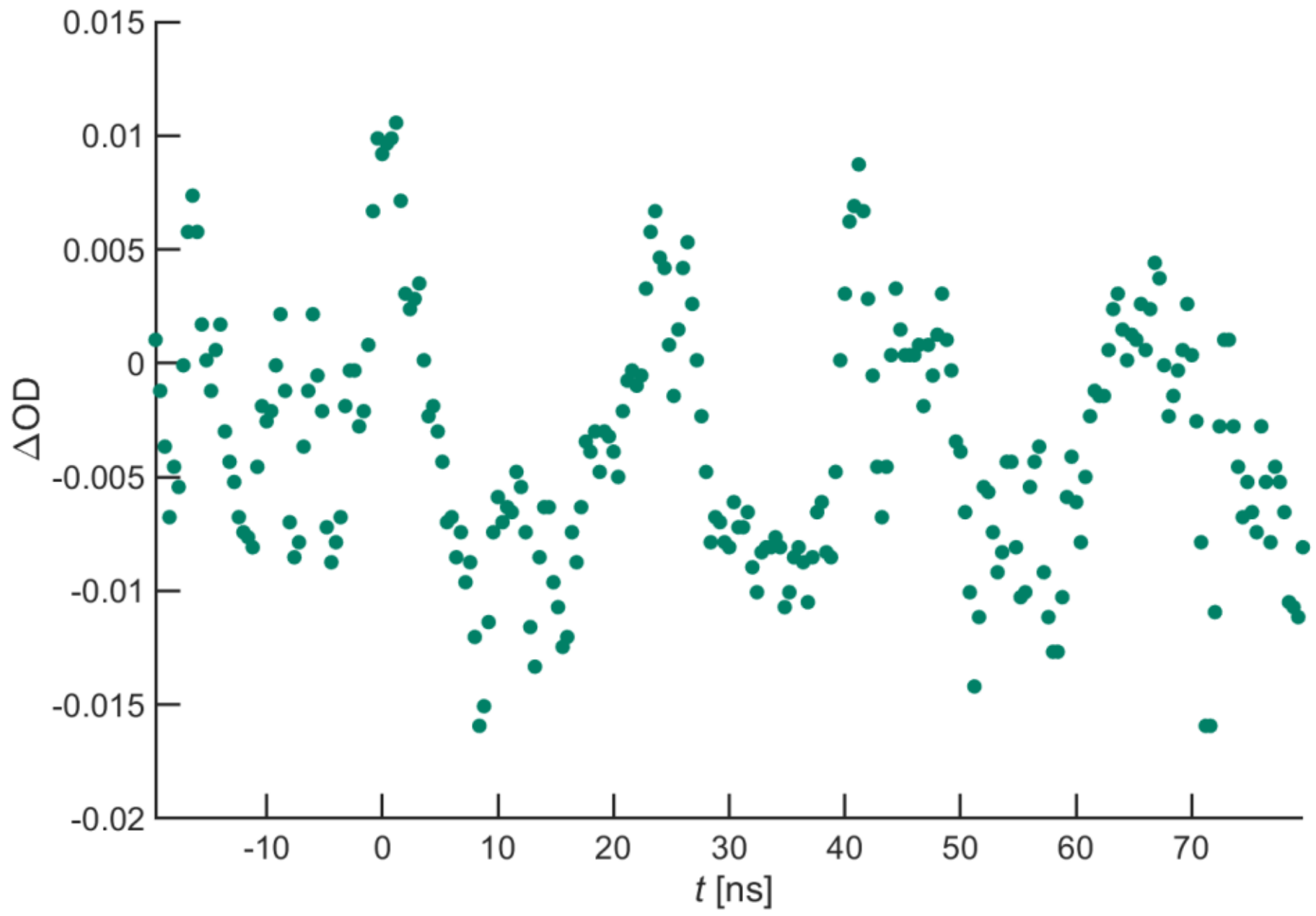

Figure S138: Oscillographic trace recorded of **NiPc-3** in H_2O with λ_{pump} = 355 nm (S-band) and λ_{probe} = 750 nm (Q-band) at the maximum laser excitation power, instrumentally averaged 20 times.

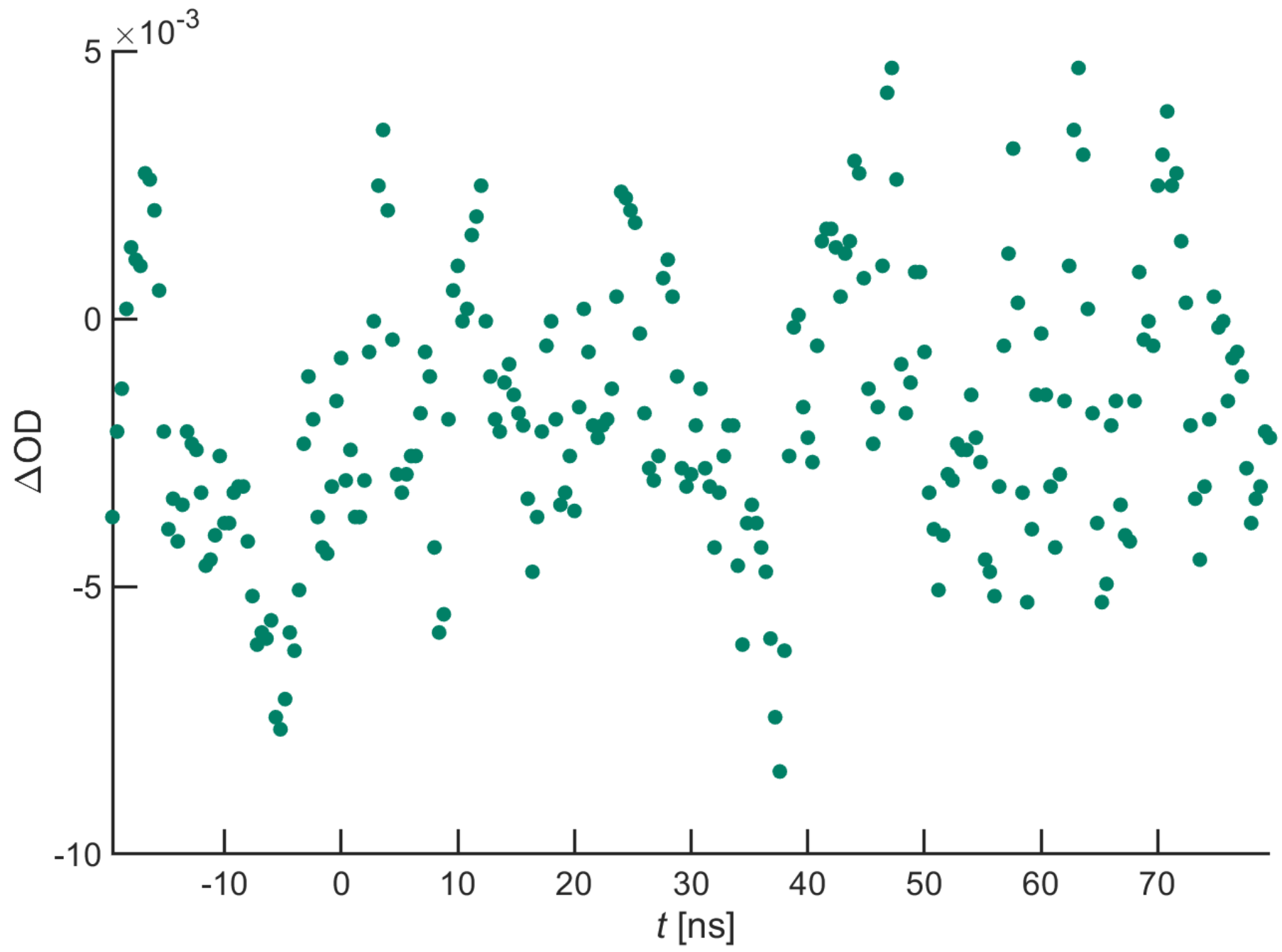

Figure S139: Oscillographic trace recorded of **NiPc-3** in H_2O with λ_{pump} = 705 nm (Q-band) and λ_{probe} = 355 nm (S-band) at the maximum laser excitation power, instrumentally averaged 20 times.

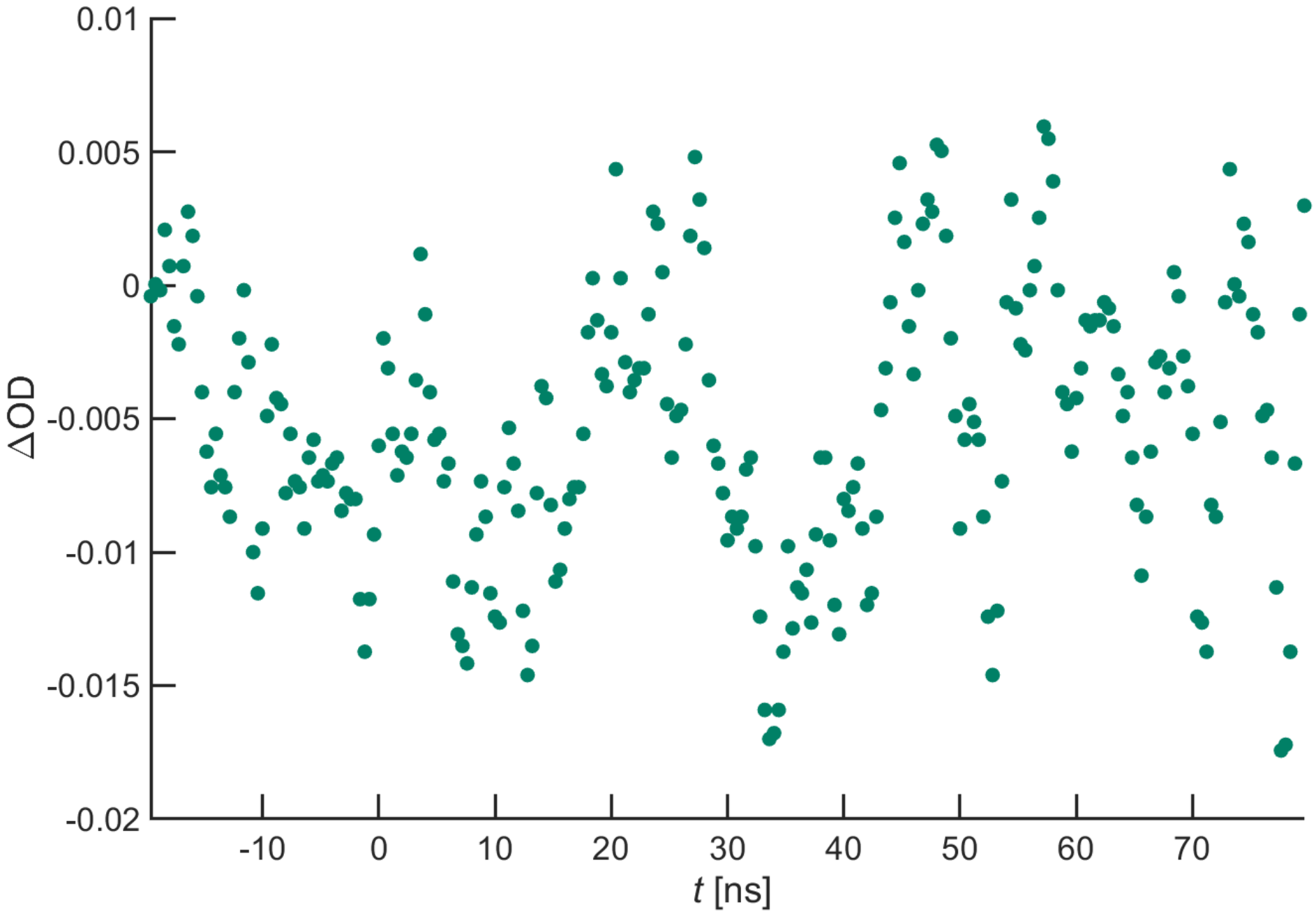

Figure S140: Oscillographic trace recorded of **NiPc-3** in H_2O with λ_{pump} = 705 nm (Q-band) and λ_{probe} = 750 nm (Q-band) at the maximum laser excitation power, instrumentally averaged 20 times.

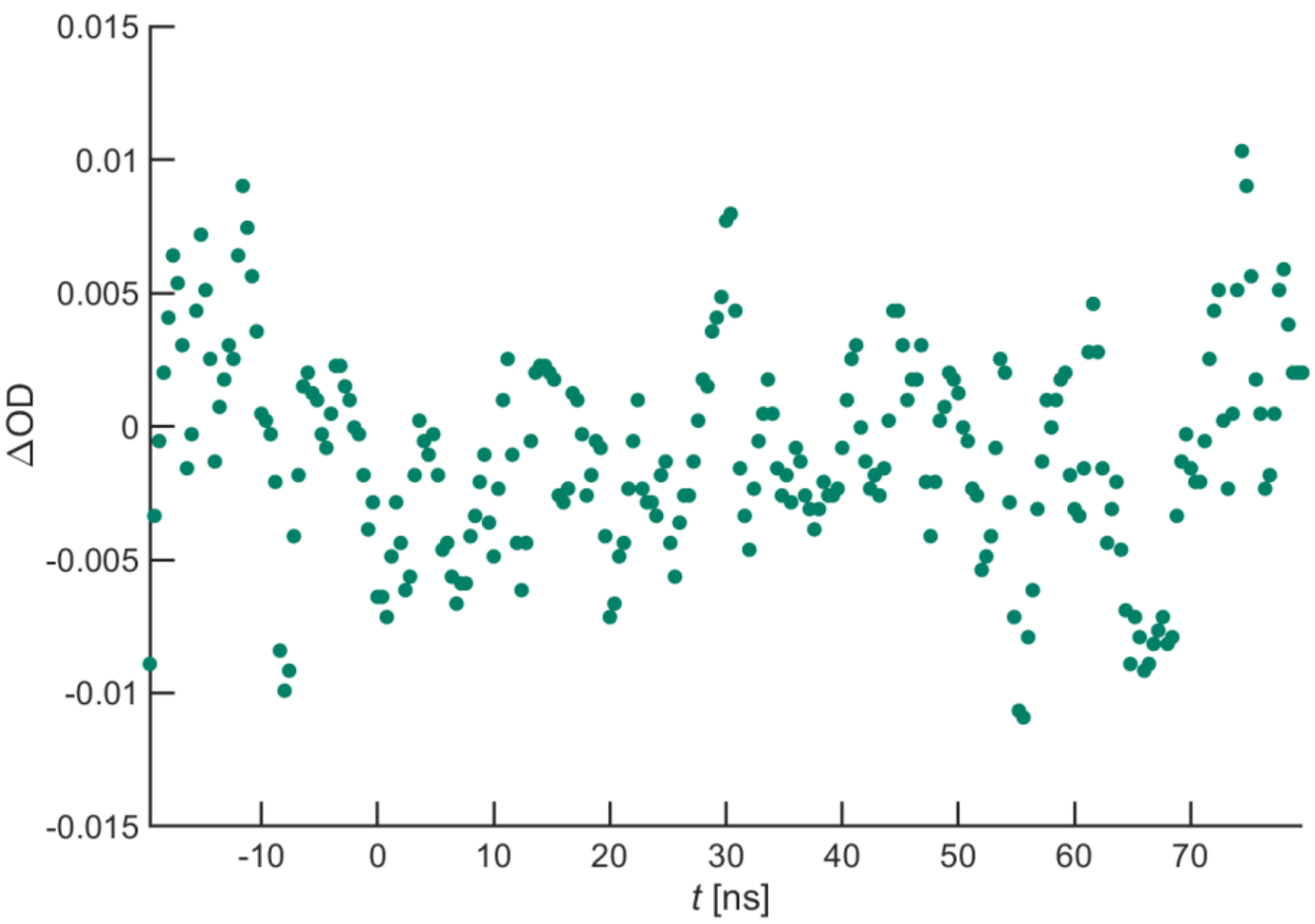

Figure S141: Oscillographic trace recorded of **NiPc-4** in H_2O with λ_{pump} = 355 nm (S-band) and λ_{probe} = 680 nm (Q-band) at the maximum laser excitation power, instrumentally averaged 20 times.

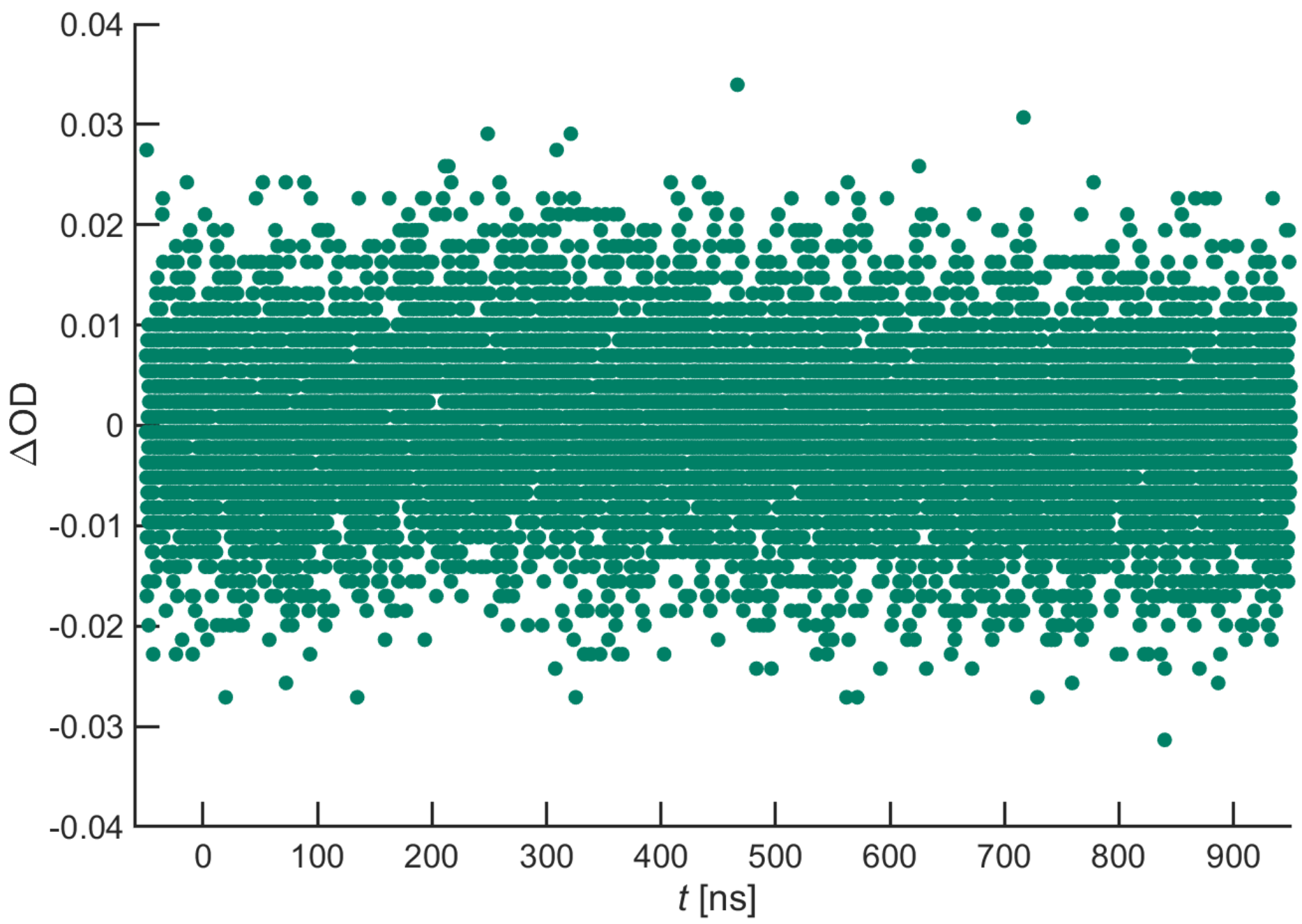

Figure S142: Oscillographic trace recorded of **NiPc-4** in H_2O with λ_{pump} = 700 nm (Q-band) and λ_{probe} = 330 nm (S-band) at the maximum laser excitation power, instrumentally averaged 20 times.

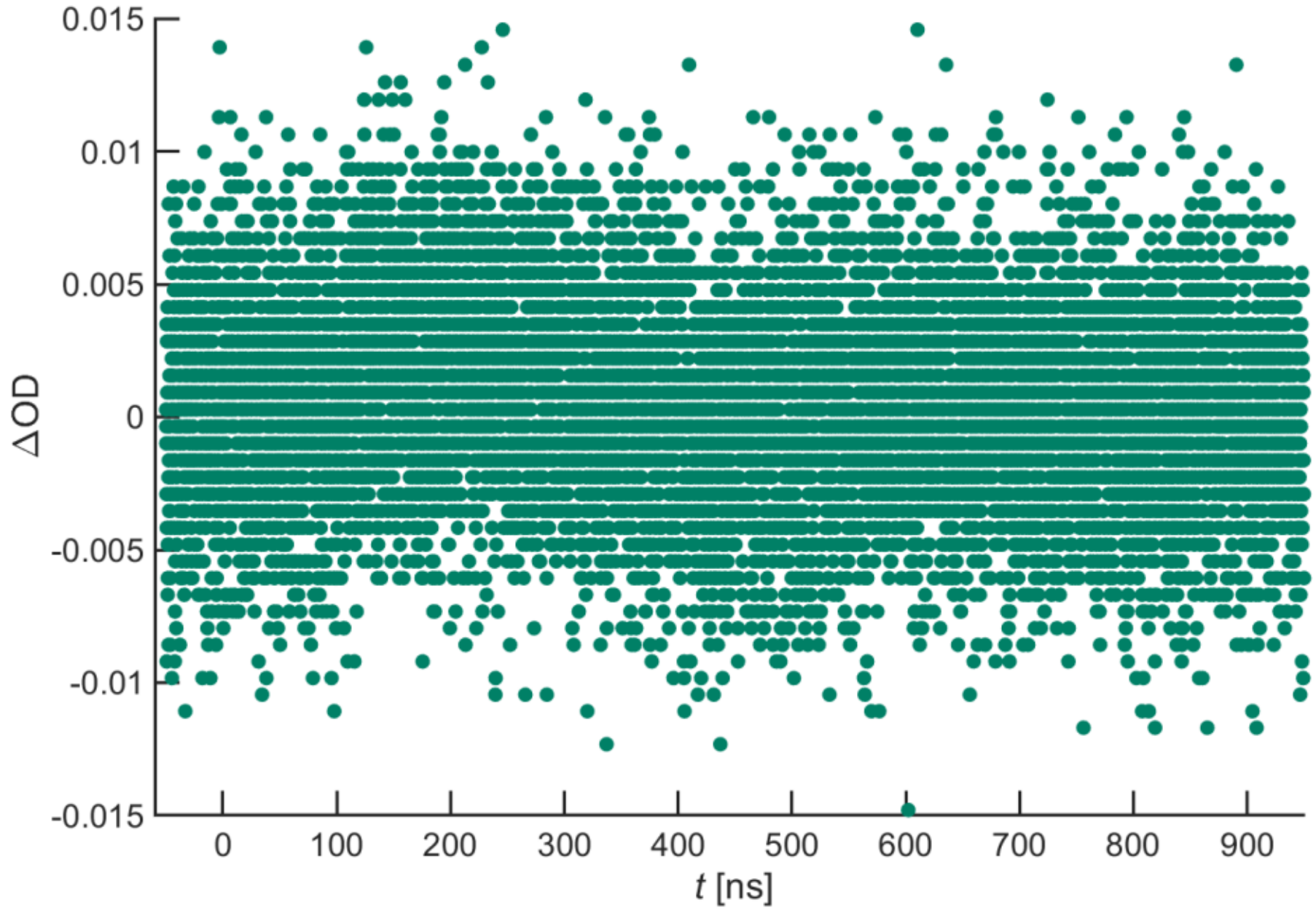

Figure S143: Oscillographic trace recorded of **NiPc-4** in H_2O with λ_{pump} = 700 nm (Q-band) and λ_{probe} = 680 nm (Q-band) at the maximum laser excitation power, instrumentally averaged 20 times.

Section S10: References

[1] J.-Y. Liu, X.-J. Jiang, W.-P. Fong, D. K. P. Ng, *Org. Biomol. Chem.* **2008**, *6*, 4560–4566.

[2] S. Tuncel, F. Dumoulin, J. Gailer, M. Sooriyaarachchi, D. Atilla, M. Durmuş, D. Bouchu, H. Savoie, R. W. Boyle, V. Ahsen, *Dalton Trans.* **2011**, *40*, 4067–4079.

[3] M. Surowiec, R. Custelcean, K. Surowiec, R. A. Bartsch, *Tetrahedron* **2009**, *65*, 7777–7783.

[4] A. G. Martynov, Y. G. Gorbunova, S. E. Nefedov, A. Y. Tsivadze, J.-P. Sauvage, *Eur. J. Org. Chem.* **2012**, *35*, 6888–6894.

[5] G. Mbambisa, P. Tau, E. Antunes, T. Nyokong, *Polyhedron* **2007**, *26*, 5355–5364.

[6] L. Sejdarasi, K. J. McAuliffe, B. A. Corbin, E. R. Trivedi, *ChemistrySelect* **2017**, *2*, 7417–7420.

[7] T. Furuyama, K. Satoh, T. Kushiya, N. Kobayashi, *J. Am. Chem. Soc.* **2014**, *136*, 765–776.

[8] H. Frisch, E.-C. Fritz, F. Stricker, L. Schmüser, D. Spitzer, T. Weidner, B. J. Ravoo, P. Besenius, *Angew. Chem., Int. Ed.* **2016**, *55*, 7242–7246.
